# Left corticospinal tract could be a biomarker to identify the dual prodromal LRRK2/GBA mutated Parkinson's disease

**DOI:** 10.1111/cns.14728

**Published:** 2024-06-04

**Authors:** Fabin Lin, Xinlin Ruan, Xinyang Zou, Huidan Weng, Yuqi Zeng, Jiayi Zheng, Qinyong Ye, Fangang Meng, Xiaochun Chen, Guoen Cai

**Affiliations:** ^1^ Department of Neurology, Center for Cognitive Neurology, Institute of Clinical Neurology Fujian Medical University Union Hospital Fuzhou China; ^2^ Fujian Institute of Geriatrics Fujian Medical University Union Hospital Fuzhou China; ^3^ Fujian Key Laboratory of Molecular Neurology Fujian Medical University Fuzhou China; ^4^ Department of Neurosurgery Fujian Medical University Union Hospital Fuzhou China; ^5^ Beijing Neurosurgical Institute, Beijing Tiantan Hospital Capital Medical University Beijing China

**Keywords:** diffusion tensor images, GBA, LRRK2, prodromal Parkinson's disease, white matter

## Abstract

**Introduction:**

Prodromal Parkinson's disease (PD) carriers of dual leucine‐rich repeat kinase 2 (LRRK2) and glucosylceramidase β (GBA) variants are rare, and their biomarkers are less well developed.

**Objective:**

This study aimed to investigate the biomarkers for diagnosing the prodromal phase of LRRK2‐GBA‐PD (LRRK2‐GBA‐prodromal).

**Methods:**

We assessed the clinical and whole‐brain white matter microstructural characteristics of 54 prodromal PD carriers of dual LRRK2 (100% M239T) and GBA (95% N409S) variants, along with 76 healthy controls (HCs) from the Parkinson's Progression Markers Initiative (PPMI) cohort.

**Results:**

By analyzing the four values of 100 nodes on 20 fiber bundles, totaling 8000 data points, we identified the smallest *p* value in the fractional anisotropy (FA) value of the 38th segment of left corticospinal tract (L‐CST) with differences between LRRK2‐GBA‐prodromal and HCs (*p* = 8.94 × 10^−9^). The FA value of the 38th node of the L‐CST was significantly lower in LRRK2‐GBA‐prodromal (FA value, 0.65) compared with HCs (FA value, 0.71). The receiver‐operating characteristic curve showed a cut‐off value of 0.218 for the FA value of L‐CST, providing sufficient sensitivity (79.2%) and specificity (72.2%) to distinguish double mutation prodromal PD from the healthy population.

**Conclusion:**

L‐CST, especially the 38th node, may potentially serve as a biomarker for distinguishing individuals with double mutation prodromal PD from the healthy population.

## Graphical abstract

Identification of a specific biomarker, the 38th node of the left corticospinal tract (L‐CST), for distinguishing double mutation prodromal Parkinson's disease (LRRK2‐GBA‐prodromal) from healthy individuals. This finding has potential implications for early diagnosis and personalized medical management.

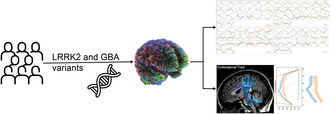

## 1 INTRODUCTION

Parkinson's disease (PD) is a progressive neurodegenerative disorder impacting approximately 1% of the population aged more than 60 years.^1^ Evidence suggests genotype–phenotype correlations in patients with PD who have specific genetic mutations.^2^, ^3^, ^4^ Compared with idiopathic PD (iPD), glucosylceramidase β PD (GBA‐PD) was associated with motor impairment at a younger age, higher incidence and earlier onset of cognitive impairment, and more psychiatric symptoms, and leucine‐rich repeat kinase 2 PD (LRRK2‐PD) exhibited better cognitive function and fewer psychiatric symptoms compared with iPD.^5^, ^6^ Notably, a small percentage of patients carried mutations in both genes.

Three studies investigating phenotypes consistently concluded that individuals with PD carrying mutations in both genes (LRRK2‐GBA‐PD) had a similar or milder phenotype compared with those with LRRK2‐PD^7^, ^8^, ^9^ alone. However, evidence on the pathological mechanisms in LRRK2‐GBA‐PD, especially during the prodromal phase of LRRK2‐GBA‐PD (LRRK2‐GBA‐prodromal), is insufficient. Also, early identification and accurate diagnosis of patients with PD with double mutations are neglected in most studies focusing on patients with PD with single mutations. Therefore, early identification, accurate diagnosis, and personalized medical management tailored for LRRK2‐GBA‐prodromal individuals are necessary.

Neuroimaging changes occur early in PD development, and changes in whole‐brain white matter microstructure have been observed in the prodromal phase of PD.^10^ Previous studies have described anisotropic changes in the substantia nigra, midbrain, and pontine fractions in patients with idiopathic rapid eye movement (REM) sleep behavior disorder to measure early neurodegeneration in the prodromal phase of PD.^11^ Therefore, identifying and monitoring patients with whole‐brain white matter microstructural damage are crucial. However, evidence for reliable neuroimaging biomarkers suggesting early neurodegenerative processes in the prodromal phase of PD is lacking.

Biomarkers that clearly distinguish between LRRK2‐GBA‐prodromal and healthy individuals are of great significance in early identification and diagnosis. Therefore, we examined the extent of whole‐brain white matter microstructural damage in individuals with LRRK2‐GBA‐prodromal to investigate whether white matter microstructure correlated with neurological involvement.

## 2 MATERIALS AND METHODS

### 2.1 Data sources

We downloaded data from the Parkinson's Progression Markers Initiative (PPMI) database (www.ppmi‐info.org/data). A prospective, longitudinal, and multicenter project (involving 16 US, 5 European, and 1 Australian sites) is underway to identify the biomarkers of PD progression, including serological and imaging markers, to improve the understanding of PD mechanisms.^12^ The present study was conducted in accordance with the principles outlined in the Declaration of Helsinki.^13^ Signed informed consent was obtained from all participants recruited in this study. The protocol and manuals for this study can be found at www.ppmi‐info.org/study‐design.

The study was approved by the ethics committee and has been registered with ClinicalTrials.gov (NCT01141023).

### 2.2 Study participants

**FIGURE 1:**
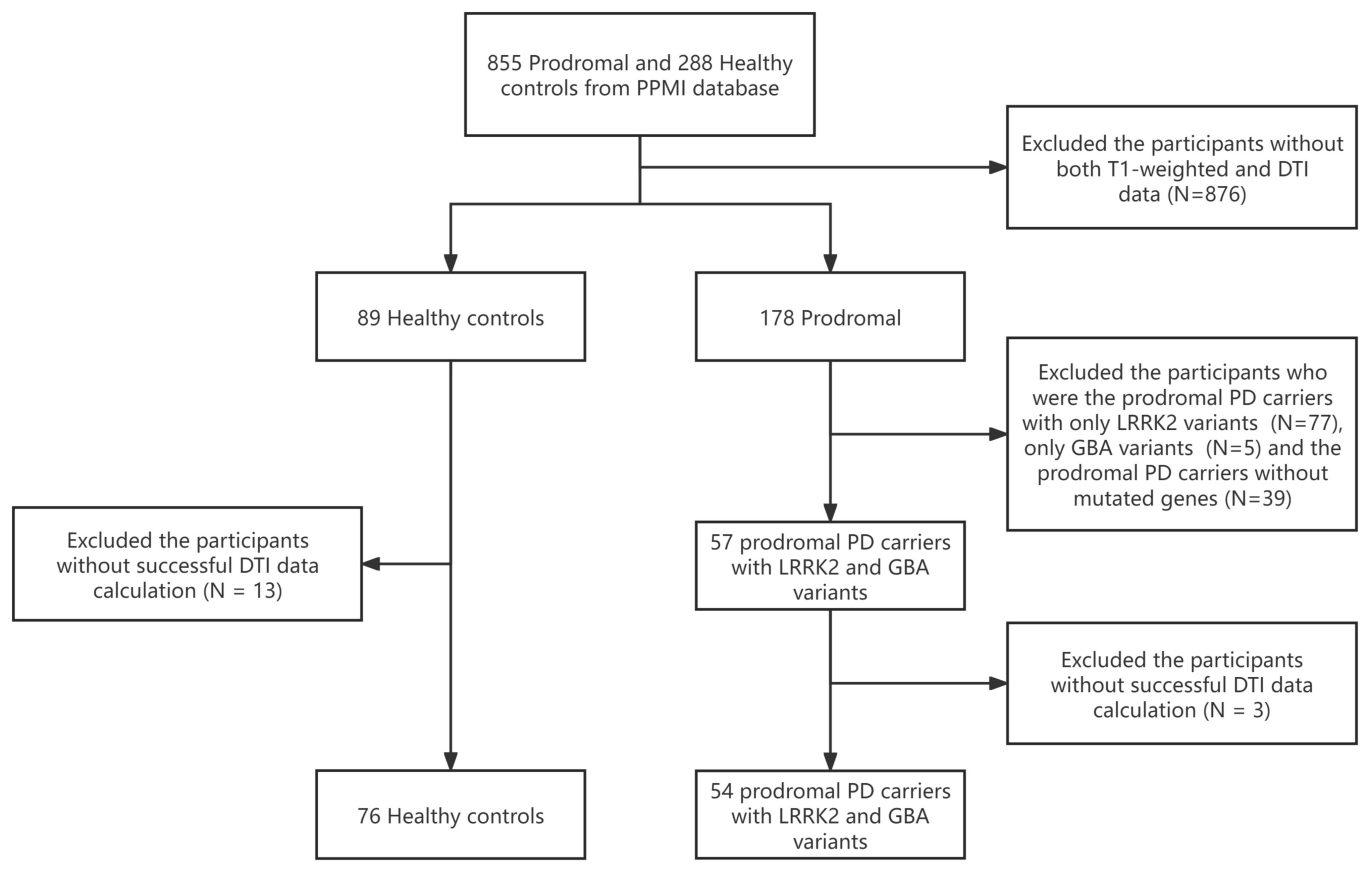
Flowchart of the study. DTI, Diffusion tensor imaging; PD, Parkinson's disease; PPMI, Parkinson's Progression Markers Initiative.

The study participants were recruited from the entire PPMI cohort. We retrieved 855 patients with prodromal PD enrolled in the prodromal cohorts, including patients with REM sleep behavior disorder (RBD), olfactory loss, genetic variants such as LRRK2, GBA, α‐synuclein (SNCA), Parkin, and Pink1, and/or other risk factors for PD with and without dopamine transporter (DAT) deficit, along with 288 healthy controls (HCs). We retrieved the prodromal and HCs with both T1‐weighted and diffusion tensor imaging (DTI) data, selecting 178 individuals with prodromal PD and 89 HCs. The data regarding GBA and LRRK2 variants in the PPMI were reviewed. We excluded 77 patients with prodromal PD with only LRRK2 variants, five patients with prodromal PD with only GBA variant, and 39 patients with prodromal PD without mutated genes. Thus, this study included 57 participants with LRRK2‐GBA‐prodromal. Of these, 13 were excluded from this study because of the unavailability of magnetic resonance imaging (MRI) data for analysis. Three patients with LRRK2‐GBA‐prodromal were excluded because of unsuccessful DTI data calculation. Finally, 76 participants were included in the HC group, and 54 patients with LRRK2‐GBA‐prodromal were included. The participant selection process is shown in Figure 1.

### 2.3 Clinical evaluation

The clinical and demographic features included age, sex, and education level of the patients. The Movement Disorders Society–sponsored Unified Parkinson's Disease Rating Scale (MDS‐UPDRS) I, MDS‐UPDRS II, and MDS‐UPDRS III were included in the movement rating scales.^14^ Global cognition was assessed using the Montreal Cognitive Assessment (MoCA).^15^ Screening for REM sleep behavior disorder was conducted using the RBD screening questionnaire (RBDSQ). The Geriatric Depression Scale–Short Form (GDS‐SF)^16^ was administered to assess depressive symptoms, whereas the State–Trait Anxiety Inventory (STAI)^17^ was used to evaluate the symptoms of anxiety. Motor and nonmotor symptoms were collected at baseline.

### 2.4 MRI acquisition and processing

Diffusion imaging data were downloaded from the PPMI website. The diffusion images were preprocessed using the FMRIB Software Library (FSL) 6.0.5.^18^ After preprocessing, fiber tracking and tract node delineation were conducted using the Automated Fiber Quantification (AFQ; https://github.jyeatman/AFQ) software, which was implemented in MATLAB R2018b (MathWorks, MA, USA).^19^ Eventually, we obtained metrics including fractional anisotropy (FA), mean diffusivity (MD), axial diffusivity (AD), and radial diffusivity (RD). For detailed experimental procedures and parameters, please refer to Figure S1 and Data S1.

### 2.5 Statistical analyses

The chi‐squared (*χ* ^2^) and *t* tests were used to compare the demographic and clinical characteristics of the two samples using SPSS software (Version 25.0). A *p* value < 0.05 indicated statistical significance. The between‐group DTI metrics were determined by averaging the diffusion values from 100 nodes along each white matter tract and using the general linear model (GLM) to analyze the correlation between FA, MD, AD, and RD metrics and the two groups of individuals based on the GLM packages in the R software. Age, education, and sex were included in the GLM as covariates. Next, pointwise analyses were performed using two‐tailed *t* tests based on the “rstatix” and “reshape2” packages in the R software. False discovery rate (FDR)^20^ correction was applied to the 2000 points (20 fibers × 100 points), and a corrected significant level was set at 0.05. Logistic regression analysis was used to eliminate potential confounding factors (sex, age, and years of education) affecting the FA value of white matter fiber tracts. The receiver‐operating characteristic (ROC) curve was used to assess the diagnostic sensitivity and specificity of detecting the FA value of white matter fiber tracts.

## 3 RESULTS

### 3.1 Demographic and clinical characteristics

The demographic and clinical characteristics of HC and individuals with LRRK2‐GBA‐prodromal are provided in Table 1. No significant difference in age was found, but differences were observed in the years of education and sex between the HC and LRRK2‐GBA‐prodromal (*p* < 0.05). LRRK2‐GBA‐prodromal displayed poorer performance on MDS‐UPDRS I (*p* < 0.05), MDS‐UPDRS II (*p* < 0.01), MDS‐UPDRS III (*p* < 0.01), R (*p* < 0.05), and MoCA (*p* < 0.05) compared with the HCs. No significant difference in GDS‐SF and STAI was noted between HCs and LRRK2‐GBA‐prodromal (*p* > 0.05).

**TABLE 1:** Demographic and clinical characteristics of HC and LRRK2‐GBA‐prodromal.

	Healthy controls (*n* = 76)	LRRK2‐GBA‐prodromal (*n* = 54)	*p* value
Age^a^	61.5 (52.6–67.2)	60.6 (56.6–67.6)	0.519
Sex (F/M)^b^	25/51	34/20	0.001**
Education^a^	16.0 (13.0–18.0)	18.0 (16.0–19.3)	<0.001***
MDS‐UPDRS I^a^	0.0 (0.0–1.0)	0.0 (0.0–1.3)	0.042*
MDS‐UPDRS II^a^	0.0 (0.0–0.0)	0.0 (0.0–2.0)	0.002**
MDS‐UPDRS III^a^	0.0 (0.0–1.0)	0.5 (0.0–3.0)	0.005**
RBDSQ^a^	3.0 (1.0–4.0)	3.0 (2.0–4.3)	0.027*
MoCA^a^	28.0 (27.0–29.0)	28.0 (26.0–29.0)	0.04*
GDS‐SF^a^	5.0 (5.0–6.0)	5.0 (4.0–6.0)	0.751
STAI^a^	94.5 (89.0–98.0)	94.0 (89.8–97.0)	0.46

*Note*: Unless otherwise stated, all values are expressed as mean ± SD.

Abbreviations: GDS‐SF, The Geriatric Depression Scale–Short Form; MoCA, Montreal Cognitive Assessment; MUPDRS, Movement Disorders Society–sponsored Unified Parkinson's disease rating scale; RBDSQ, REM sleep behavior disorder screening questionnaire; STAI, State–Trait Anxiety Inventory.

^a^ The *p* value was obtained using the Mann–Whitney test.

^b^ The *p* value was obtained using the *χ* ^2^ test.

* A *p* value < 0.05 indicates a statistical difference between groups.

** A *p* value < 0.01 indicates a statistical difference between groups.

*** A *p* value < 0.001 indicates a statistical difference between groups.

### 3.2 Group differences in the white matter tract and point‐wise levels

A between‐group difference in the white matter tract and point‐wise alterations was determined using mean diffusion metrics (FA, MD, AD, and RD) with AFQ. We successfully identified 20 fiber bundles in two groups using AFQ. Table S1 shows the unsuccessful identification rate for each of the 20 fiber tracts in HCs and LRRK2‐GBA‐prodromal.

#### 3.2.1 FA profiles

**FIGURE 2:**
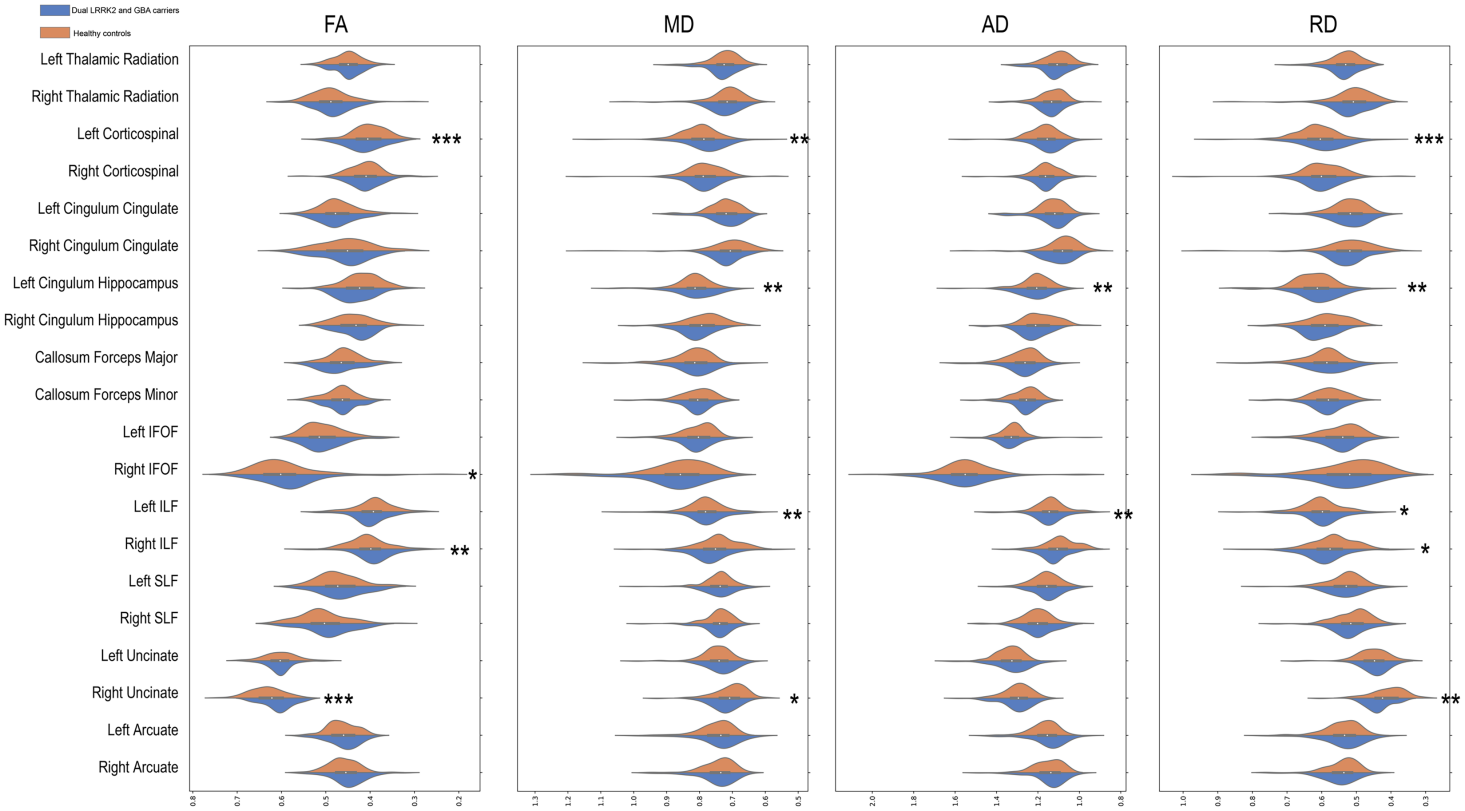
Violin plots showing distributions for the mean fractional anisotropy (FA), mean diffusivity (MD), axial diffusivity (AD), and radial diffusivity (RD) values for white matter tracts between HCs and patients with prodromal PD with dual LRRK2 and GBA variants. The *x*‐axis indicates the mean FA, MD, AD, and RD values for white matter tracts, whereas the *y*‐axis denotes 20 white matter tracts. The orange segments illustrate the mean FA, MD, AD, and RD values for white matter tracts in HCs, and the blue segments depict the mean values for patients with prodromal PD with dual LRRK2 and GBA variants. Statistical significance was determined using *p* values (*p < 0.05, **p < 0.01, ***p < 0.001) assessed using generalized linear models. AD, axial diffusivity; FA, fractional anisotropy; IFOF, inferior fronto‐occipital fasciculus; ILF, inferior longitudinal fasciculus; MD, mean diffusivity; RD, radial diffusivity; SLF, superior longitudinal fasciculus.

**FIGURE 3:**
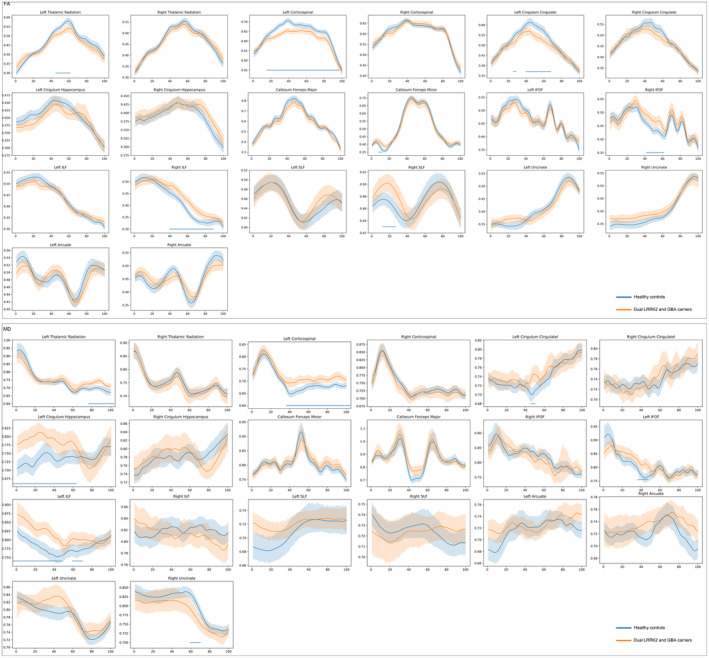
Pointwise comparison of FA and MD profiles between patients with prodromal PD with dual LRRK2 and GBA variants and HCs. The plots depict the profiles of FA and MD profiles across 20 identified fiber tracts in patients with prodromal PD with dual LRRK2 and GBA variants and HCs presented as mean (standard deviation, SD) values (solid lines represent the means, whereas shaded areas indicate the SDs). The blue bars under the profile indicate the regions of significant difference (FDR correction, *p* < 0.05) between patients with prodromal PD with dual LRRK2 and GBA variants and HCs. The *x*‐axis represents the spatial location between the beginning and termination waypoint regions of interest. FA, fractional anisotropy; IFOF, inferior fronto‐occipital fasciculus; ILF, inferior longitudinal fasciculus; MD, mean diffusivity; SLF, superior longitudinal fasciculus.

The mean FA values for the white matter tracts significantly changed between the groups with respect to the left corticospinal tract (L‐CST), right inferior fronto‐occipital fasciculus (IFOF), right inferior longitudinal fasciculus (ILF), and right uncinate (Figure 2 and Table S2). A point‐by‐point comparison of FA profiles confirmed the following significantly altered fiber bundle positions (FDR corrected, *p* < 0.05): the intermediate part of left thalamic radiation (*N* 45–60); the anterior, intermediate, and posterior parts of L‐CST (*N* 18–93); the anterior and intermediate parts of the left cingulum cingulate (*N* 27–30, 40–66); the anterior part of the callosum forceps minor (*N* 11–15); the intermediate part of the right IFOF (*N* 42–60); the intermediate part of the right ILF (*N* 40–85); and the anterior part of the right superior longitudinal fasciculus (SLF) (*N* 15–28; Figure 3 and Table S3).

#### 3.2.2 MD profiles

Compared with HCs, patients with prodromal PD carrying LRRK2 and GBA variants showed significant changes in MD values in the L‐CST, left cingulum hippocampus, left ILF, and right uncinate fasciculus (Figure 2 and Table S2). A detailed comparison of MD profiles revealed significant changes in the following fiber bundle segments (FDR corrected, *p* < 0.05): the anterior and intermediate regions of the superior segment of the left thalamic radiation (*N* 74–100); the intermediate and superior segments of the L‐CST (*N* 37–100); the intermediate segment of the left cingulum cingulate (*N* 46–50); the anterior and intermediate segments of the left cingulum hippocampus (*N* 1–62); the anterior and intermediate segments of the right cingulum hippocampus (*N* 1–62); the intermediate segment of the callosum forceps major (*N* 42–43); the intermediate segment of the left IFOF (*N* 37–47); the anterior and intermediate segments of the left ILF (*N* 1–68); the intermediate segment of the right uncinate fasciculus (*N* 59–68); and the intermediate and superior segments of the right arcuate fasciculus (*N* 45–50 and 92–100; Figure 3 and Table S4).

#### 3.2.3 AD profiles

**FIGURE 4:**
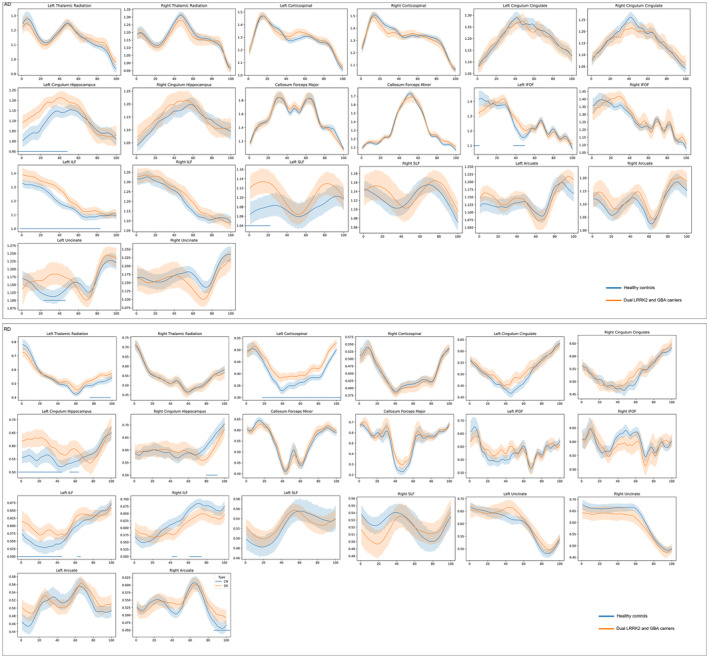
Pointwise comparison of AD and RD profiles between patients with prodromal PD with dual LRRK2 and GBA variants and HCs. The plots depicting the AD and RD profiles of 20 identified fiber tracts in patients with prodromal PD with dual LRRK2 and GBA variants and HCs, shown as mean (standard deviation, SD) values (solid lines represent the means, whereas shaded areas indicate SDs). The blue bars under the profile indicate the regions of significant difference (FDR correction, *p* < 0.05) between patients with prodromal PD with dual LRRK2 and GBA variants and HCs. The *x*‐axis represents the spatial location between the beginning and termination waypoint regions of interest. AD, axial diffusivity; IFOF, inferior fronto‐occipital fasciculus; ILF, inferior longitudinal fasciculus; RD, radial diffusivity; SLF, superior longitudinal fasciculus.

The mean AD values of the white matter tracts significantly changed between the groups with respect to the left cingulum hippocampus and left ILF (Figure 2 and Table S2). A detailed comparison of AD profiles confirmed the following significantly altered fiber bundle positions (FDR corrected, *p* < 0.05): the anterior segment of the left cingulum hippocampus (*N* 1–48); the anterior and intermediate segments of the callosum forceps major (*N* 1–5, 38–49); the superior segment of the callosum forceps minor (*N* 87–89); the anterior, intermediate, and superior segments of the left ILF (*N* 2–80); the anterior segment of the left SLF (*N* 1–24); and the intermediate segment of the left uncinate fasciculus (*N* 25–42; Figure 4 and Table S5).

#### 3.2.4 RD profiles

Compared with HCs, patients with prodromal PD carrying LRRK2 and GBA variants exhibited significant changes in RD values in the L‐CST, left cingulum hippocampus, left ILF, right ILF, and right uncinate fasciculus (Figure 2 and Table S2). A detailed comparison of RD profiles confirmed the following significantly altered fiber bundle positions (FDR corrected, *p* < 0.05): the intermediate and superior segments of the left thalamic radiation (*N* 51–94); the intermediate and superior segments of the L‐CST (*N* 20–100); the anterior and intermediate segments of the left cingulum cingulate (*N* 27–30, 42–64); the anterior and intermediate segments of the left cingulum hippocampus (*N* 1–62); the superior segment of the right cingulum hippocampus (*N* 77–88); the anterior and intermediate segments of the left IFOF (*N* 33–34 and 44–45); the anterior and intermediate segments of the left ILF (*N* 2–64); the intermediate and superior segments of the right ILF (*N* 42–47 and 60–72); and the intermediate and superior segments of the right arcuate fasciculus (*N* 40–47 and 83–99; Figure 4 and Table S6).

### 3.3 Regression analysis of LRRK2‐GBA‐prodromal of the 38th node of L‐CST

**FIGURE 5:**
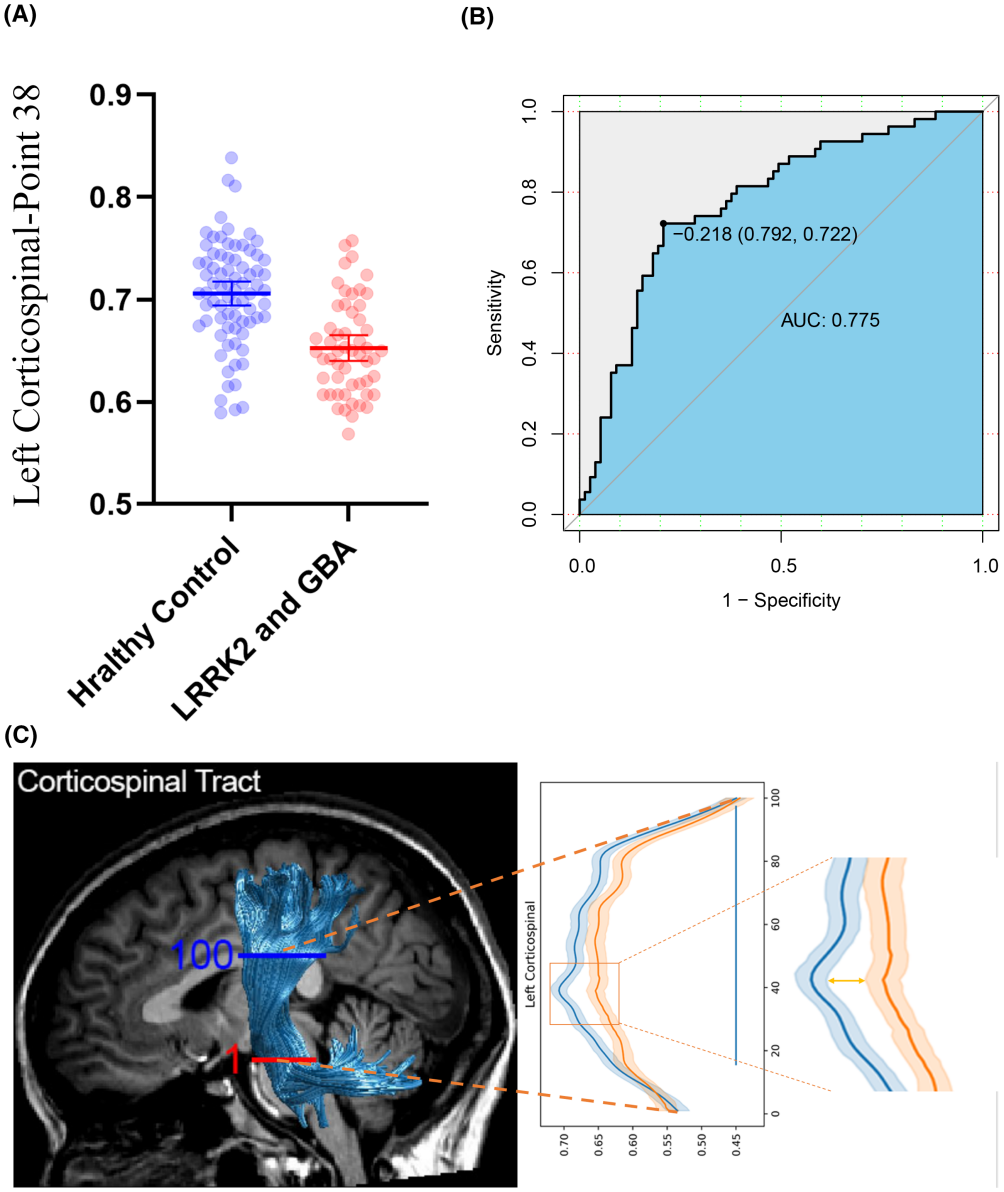
FA value of the 38th node of L‐CST in patients with LRRK2‐GBA‐prodromal and HCs. (A) Scatter plots with error bars representing the FA value of the 38th node of L‐CST in HCs and patients with LRRK2‐GBA‐prodromal. (B) Analysis of receiver‐operating characteristic curve distinguishing between HCs and patients with LRRK2‐GBA‐prodromal. (C) Graphical representation of the FA value of the 38th node of L‐CST. AUC, area under the curve.

By analyzing 100 nodes on 20 fiber bundles, we found that the FA value of the 38th node of L‐CST had the smallest *P* value for both groups compared (*p* = 8.94 × 10^−9^; Tables S3–S6). In individuals with LRRK2‐GBA‐prodromal, the FA value of the 38th node of L‐CST was lower than that of HCs (0.65 ± 0.006 vs. 0.71 ± 0.006; *p* < 0.001, *t* = −6.077; Figure 5 and Table S3). After adjusting for age, sex, and years of education using logistic regression analysis, a significant difference was found between patients with LRRK2‐GBA‐prodromal and HCs [odds ratio (OR) 95% confidence interval (CI), 20.61 (4.44–29.31); *p* < 0.001]. We performed ROC curve analysis to assess the ability of the FA value of the 38th node of L‐CST to discriminate between individuals with LRRK2‐GBA‐prodromal and HCs, which showed that the cut‐off value of the FA value of the 38th node of L‐CST was 0.218, with a sensitivity of 79.2% and specificity of 72.2%, and an area under the curve of 0.775 (Figure 5).

## 4 DISCUSSION

Previous studies indicated an association between whole‐brain white matter microstructural damage and PD. Based on these findings, our study aimed to explore whether such white matter microstructural damage could serve as a marker associated with LRRK2‐GBA‐prodromal. The results revealed that patients with LRRK2‐GBA‐prodromal exhibited significantly lower FA values in the L‐CST, especially at the 38th node, compared with HCs. The findings indicated that the L‐CST might be a sensitive diagnostic biomarker to distinguish HCs from patients with LRRK2‐GBA‐prodromal, indicating the presence of whole‐brain white matter microstructural damage in patients with prodromal PD. In addition, we found differences in clinical symptoms between patients with LRRK2‐GBA‐prodromal and HCs. Further, we explored the correlation between FA levels in the L‐CST and disease severity.

Our results were consistent with the findings of some other related studies demonstrating that the damage to the L‐CST was particularly pronounced in LRRK2‐GBA‐prodromal compared with other fiber bundles.

The neuroimaging evidence was found to support CST injury in PD.^21^ Taylor observed a significant increase in the white matter FA value in a broad anatomical network including the CST.^22^ A meta‐analysis showed that DTI could sensitively detect CST differences between patients with PD and HCs.^23^

The MDS‐UPDRS motor score of patients with prodromal PD carrying GBA and LRRK2 variants was higher than that of HCs.^9^ Significant differences were found in motor function, but no change in depression and anxiety was detected. Patients with prodromal PD experienced changes in motor function, but the degree of change did not affect the quality of life. For nonmotor features, cognition impairment and rapid eye movement sleep behavior disorder showed a higher prevalence in patients with prodromal PD with GBA and LRRK2 variants compared with HCs. However, a difference in depression and anxiety was not determined. None of the studies described the clinical and biological features of PD manifesting in double mutant genetic cohorts. Tanya Simuni found that the prevalence of both motor and nonmotor features of PD was higher in patients with prodromal PD with the GBA variant and patients with prodromal PD with the LRRK2 variant than in HCs.^24^ Pont‐Sunyer reported that patients with prodromal PD with the LRRK2 variant had higher MDS‐UPDRS motor scores than HCs.^25^ However, they found no difference in MoCA scores between patients with prodromal PD with the LRRK2 variant and HCs.

## 5 LIMITATIONS

This study had several limitations. The cohort of patients with prodromal PD with GBA and LRRK2 variants lacked the male predominance seen in the cohort of HCs. This was consistent with previously reported data in patients with LRRK2 and GBA mutations compared with HCs.^24^ We included sex as a covariate in the data analysis to minimize sex‐related differences. The LRRK2 (100% M239T) and GBA (95% N409S) variants represented specific mutations within these genes, thus improving our understanding of their individual effects. However, it is important to note that our conclusions are limited to these specific mutations and may not be applicable to other mutations within the genes. Given the lack of longitudinal data in this study, we could not determine the consistency and reliability of CST as a biomarker of disease activity. Further evaluation through large‐scale clinical trials will be conducted in the future. The results of this study should be interpreted with caution due to the low prevalence of double mutations, the small cohort size, and the relatively robust sensitivity and specificity observed in the ROC analysis.

## 6 CONCLUSION

L‐CST, especially at the 38th node, serves as a biomarker for distinguishing between patients with LRRK2‐GBA‐prodromal and HCs.

## AUTHOR CONTRIBUTIONS

F.B.L., X.Y.Z., X.L.R., Q.Y.Y., F.G.M., X.C.C., and G.E.C contributed to the concept and design of the study. F.B.L. and X.Y.Z. were involved in the acquisition of data, data generation, and data cleaning. F.B.L., X.Y.Z., and X.L.R. did the analysis and interpretation of data. F.B.L., X.Y.Z., Y.Q.Z., H.D.W., and J.Y.Z. contributed to the drafting of the article and revising it critically.

## CONFLICT OF INTEREST STATEMENT

All authors declare that they have not received any funding from any institution, including personal relationships, interests, grants, employment, affiliations, patents, inventions, honoraria, consultancies, royalties, stock options/ownership, or expert testimony for the last 12 months.

## Supporting information


Data S1.

## Data Availability

The data that support the findings of this study are available in Parkinson's Progression Markers Initiative at http://ppmi‐info.org. These data were derived from the following resources available in the public domain: ‐Parkinson's Progression Markers Initiative, https://www.ppmi‐info.org/.
