# Supplementary material for: Left corticospinal tract could be a biomarker to identify the dual prodromal LRRK2/GBA mutated Parkinson's disease
Source: CNS Neurosci Ther. 2024 Jun 4;30(6):e14728. doi: 10.1111/cns.14728 (PMC11150277; doi:10.1111/cns.14728)
Supplement: Supplementary file 1 — Data S1. [file CNS-30-e14728-s001.pdf]

Supplemental file 1

MRI acquisition and processing

The PPMI website was used to download diffusion imaging data (ppmi-info.org). We aimed to compare the DTI metrics between the prodromal PD carriers of dual LRRK2 and GBA variants and HC. The diffusion images were preprocessed using FSL 6.0.5. (FMRIB Software Library, FMRIB, Oxford, UK).

The preprocessing steps:

1. DCM2NII attempts to convert images from the Dicom format to the NIfTI format used by FSL
2. A non-DWI image (b0) was registered with a DWI image (b = 1000 s/mm2)
3. Head motion correction was performed using Eddy current correction
4. Exclusion of non-brain tissue.

After preprocessing, we used the Automated Fiber Quantification (AFQ; https://github.jyeatman/AFQ) software, implemented in MATLAB R2018b (Mathworks, Natick, MA), to perform fiber tracking and tract segmentation[1].

Step 1: Whole-brain tractography was performed to identify all voxels with fractional anisotropy (FA) > 0.3 in the WM mask. Terminate tracking when the FA value is less than 0.2 or the minimum angle between two consecutive fiber bundle analysis steps is greater than 30 degrees.

Step 2: Segment fiber bundles using regions of interest (ROI).

Step 3: Refinement of fiber bundles based on fiber bundle probability maps[2].

Step 4: Tow cleaning was performed to filter out fibers with an average fiber length greater than four standard deviations (SDs) or fibers that deviated from the core of the tow by more than five SDs.

Step 5: Fiber bundles were cut at the ROI of each waypoint to determine the core of fiber bundles.

Finally, divide 100 equally spaced nodes along the core of each fiber bundle and use spline interpolation to extract diffusivity metrics. Since AFQ uses strict region identification criteria, it was not successful in identifying all 20 WM regions in each participant. We filled in the missing values by the mean substitution square[3] (Table S1) .

1. Yeatman, J. D.; Dougherty, R. F.; Myall, N. J.; Wandell, B. A.; Feldman, H. M., Tract Profiles of White Matter Properties: Automating Fiber-Tract Quantification. *Plos One* **2012,** *7* (11).
2. Hua, K. G.; Oishi, K.; Zhang, J. Y.; Wakana, S.; Yoshioka, T.; Zhang, W. H.; Akhter, K. D.; Li, X.; Huang, H.; Jiang, H. Y.; van Zijl, P.; Mori, S., Mapping of Functional Areas in the Human Cortex Based on Connectivity through Association Fibers. *Cerebral Cortex* **2009,** *19* (8), 1889-1895.
3. Chen, H. F.; Huang, L. L.; Li, H. Y.; Qian, Y.; Yang, D.; Qing, Z.; Luo, C. M.; Li, M. C.; Zhang, B.; Xu, Y., Microstructural disruption of the right inferior fronto-occipital and inferior longitudinal fasciculus contributes to WMH-related cognitive impairment. *Cns Neuroscience & Therapeutics* **2020,** *26* (5), 576-588.

Figure S1： Flowchart of the DTI data analysis process.

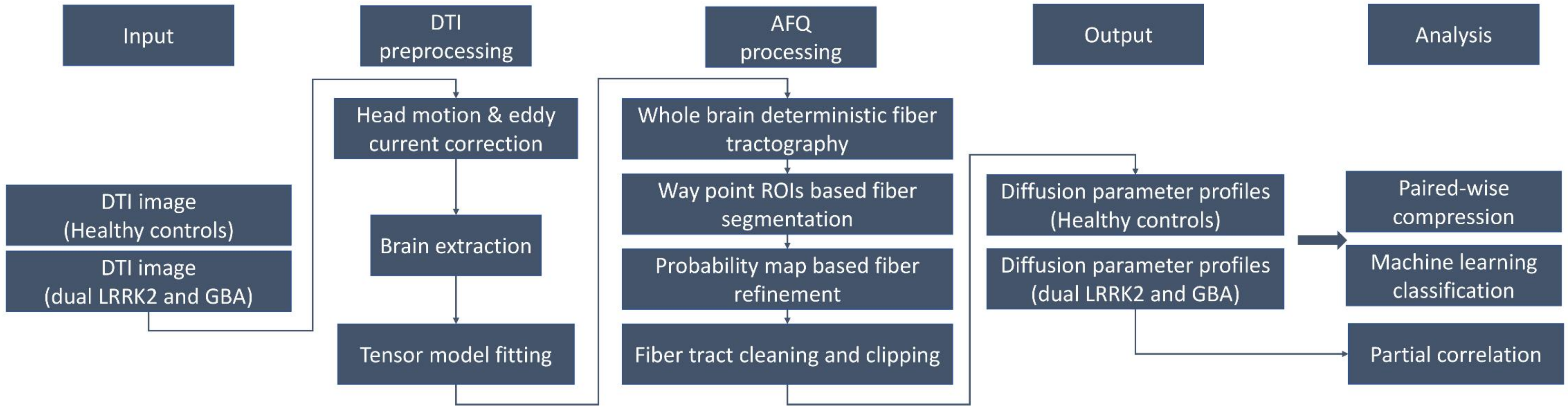

Table S1： Unsuccessful identification rate for each of 20 fiber tracts in Healthy controls and Dual LRRK2 and GBA carriers.

Fiber tract	Unsuccessful tract identification/total tract	
	Healthy controls	Dual LRRK2 and GBA carriers
Left Thalamic Radiation	1/76 (98.68%)	3/54 (94.44%)
Right Thalamic Radiation	0/76 (100%)	5/54 (90.74%)
Left Corticospinal	0/76 (100%)	5/54 (90.74%)
Right Corticospinal	0/76 (100%)	5/54 (90.74%)
Left Cingulum Cingulate	0/76 (100%)	5/54 (90.74%)
Right Cingulum Cingulate	2/76 (97.37%)	6/54 (88.89%)
Left Cingulum Hippocampus	2/76 (97.37%)	4/54 (92.59%)
Right Cingulum Hippocampus	14/76 (81.58%)	10/54 (81.48%)
Callosum Forceps Major	10/76 (86.84%)	9/54 (83.33%)
Callosum Forceps Minor	7/76 (90.79%)	4/54 (92.59%)
Left IFOF	0/76 (100%)	3/54 (94.44%)
Right IFOF	0/76 (100%)	4/54 (92.59%)
Left ILF	1/76 (98.68%)	4/54 (92.59%)
Right ILF	1/76 (98.68%)	2/54 (96.3%)
Left SLF	3/76 (96.05%)	0/54 (100%)
Right SLF	0/76 (100%)	0/54 (100%)
Left Uncinate	0/76 (100%)	0/54 (100%)
Right Uncinate	0/76 (100%)	4/54 (92.59%)
Left Arcuate	2/76 (97.37%)	4/54 (92.59%)
Right Arcuate	3/76 (96.05%)	3/54 (94.44%)

Table S2：The mean FA, MD, AD and RD values of WM tracts were compared between HC and prodromal PD carriers of LRRK2/GBA variant. Statistical analysis was performed using a generalized linear model, where * represents p < 0.05.

FA	Std.Error	t-value	*P*-value	Pr(>|t|)	
Left Thalamic Radiation	-0.012364	0.007163	-1.726	0.0868	
Right Thalamic Radiation	-3.84E-04	6.64E-03	-0.058	0.954	
Left Corticospinal	-0.0262454	0.006934	-3.785	0.000237	***
Right Corticospinal	0.0044946	0.006609	0.68	0.4977	
Left Cingulum Cingulate	-0.0154272	0.010248	-1.505	0.135	
Right Cingulum Cingulate	-0.0056402	0.00944	-0.597	0.5513	
Left Cingulum Hippocampus	-1.18E-02	8.65E-03	-1.366	0.175	
Right Cingulum Hippocampus	0.009415	0.008057	1.169	0.245	
Callosum Forceps Major	-0.0206791	0.013233	-1.563	0.121	
Callosum Forceps Minor	-0.0015478	0.007596	-0.204	0.839	
Left IFOF	-0.0050391	0.006633	-0.76	0.449	
Right IFOF	0.0150555	0.007467	2.016	0.04591	*
Left ILF	-5.59E-03	8.00E-03	-0.698	0.486	
Right ILF	0.0213234	0.008009	2.662	0.00878	**
Left SLF	7.35E-03	1.12E-02	0.655	0.514	
Right SLF	0.007857	0.008134	0.966	0.336	
Left Uncinate	1.24E-03	7.29E-03	0.17	0.865	
Right Uncinate	0.0260163	0.007194	3.617	0.000431	***
Left Arcuate	-2.91E-03	8.35E-03	-0.348	0.728	
Right Arcuate	0.0001418	0.006076	0.023	0.981	

MD	Std.Error	t	*P*-value	Pr(>|t|)	
Left Thalamic Radiation	0.0039229	0.010171	0.386	0.70036	
Right Thalamic Radiation	-0.0149312	0.011365	-1.314	0.1913	
Left Corticospinal	0.0274714	0.008976	3.061	0.0027	**
Right Corticospinal	-0.0093239	0.009043	-1.031	0.30451	
Left Cingulum Cingulate	0.0044917	0.008229	0.546	0.5861	
Right Cingulum Cingulate	-0.0034503	0.009117	-0.378	0.706	
Left Cingulum Hippocampus	0.0364948	0.011833	3.084	0.00251	**
Right Cingulum Hippocampus	-0.0024008	0.011092	-0.216	0.829	
Callosum Forceps Major	0.0215899	0.019572	1.103	0.272	
Callosum Forceps Minor	0.0044541	0.009562	0.466	0.64217	
Left IFOF	-0.0007146	0.009032	-0.079	0.93706	
Right IFOF	-0.0079765	0.011383	-0.701	0.48477	
Left ILF	0.0305015	0.010623	2.871	0.0048	**
Right ILF	-1.08E-02	1.12E-02	-0.963	0.3372	
Left SLF	0.0068416	0.0134	0.511	0.6105	
Right SLF	-0.0020756	0.00933	-0.222	0.8243	
Left Uncinate	8.58E-03	1.15E-02	0.744	0.458	

Right Uncinate	-0.0285451	0.011713	-2.437	0.0162	*
Left Arcuate	0.0064959	0.010511	0.618	0.5377	
Right Arcuate	0.0127436	0.008697	1.465	0.145	

AD	Std.Error	t	*P*-value	Pr(>|t|)	
Left Thalamic Radiation	-0.003289	0.014372	-0.229	0.819391	
Right Thalamic Radiation	-0.0235892	0.014833	-1.59	0.11429	
Left Corticospinal	0.0217089	0.013681	1.587	0.11509	
Right Corticospinal	-0.0113624	0.013911	-0.817	0.4156	
Left Cingulum Cingulate	-0.008138	0.014853	-0.548	0.585	
Right Cingulum Cingulate	-9.99E-03	1.36E-02	-0.736	0.463	
Left Cingulum Hippocampus	4.00E-02	1.43E-02	2.803	0.00587	**
Right Cingulum Hippocampus	0.0057329	0.014251	0.402	0.6882	
Callosum Forceps Major	0.0016326	0.026517	0.062	0.951	
Callosum Forceps Minor	0.0044567	0.012908	0.345	0.73	
Left IFOF	-6.29E-03	1.20E-02	-0.524	0.6012	
Right IFOF	0.006175	0.015324	0.403	0.6877	
Left ILF	0.045958	0.015018	3.06	0.00271	**
Right ILF	0.0146825	0.015061	0.975	0.331	
Left SLF	0.0281028	0.01642	1.712	0.0895	.
Right SLF	0.0154837	0.013855	1.118	0.2659	
Left Uncinate	0.0119799	0.01296	0.924	0.357	
Right Uncinate	-1.13E-02	1.43E-02	-0.792	0.43	
Left Arcuate	0.0153	0.012408	1.233	0.2199	
Right Arcuate	2.56E-02	1.31E-02	1.957	0.0526	.

RD	Std.Error	t	*P*-value	Pr(>|t|)	
Left Thalamic Radiation	0.0086248	0.009674	0.892	0.37433	
Right Thalamic Radiation	-0.0103049	0.010749	-0.959	0.33957	
Left Corticospinal	0.0314044	0.008877	3.538	0.000567	***
Right Corticospinal	-0.0068964	0.00894	-0.771	0.442	
Left Cingulum Cingulate	0.013256	0.009532	1.391	0.16679	
Right Cingulum Cingulate	0.0028129	0.010097	0.279	0.78101	
Left Cingulum Hippocampus	3.23E-02	1.22E-02	2.651	0.00906	**
Right Cingulum Hippocampus	-0.0056516	0.011371	-0.497	0.62	
Callosum Forceps Major	0.0306351	0.020538	1.492	0.138	
Callosum Forceps Minor	0.0032146	0.009831	0.327	0.7442	
Left IFOF	0.0014242	0.009091	0.157	0.87576	
Right IFOF	-0.0176312	0.01126	-1.566	0.12	
Left ILF	0.0247604	0.010811	2.29	0.0237	*
Right ILF	-0.0234379	0.011388	-2.058	0.04166	*
Left SLF	6.42E-05	1.42E-02	0.005	0.9964	
Right SLF	-0.0053973	0.009645	-0.56	0.577	
Left Uncinate	0.0066582	0.012025	0.554	0.581	

Right Uncinate	-0.035974	0.011641	-3.09	0.00247	**
Left Arcuate	0.0061602	0.011483	0.536	0.5926	
Right Arcuate	0.0079027	0.008327	0.949	0.344	

Table S3：Comparison of Mean FA Between prodromal PD carriers of dual LRRK2 and GBA variants and Healthy Controls Across 20 Fiber Tracts. Statistical significance was set at $p < 0.05$, corrected by FDR.

1	statistic	df	p	p.adj	p.adj.signif	2	statistic	df	p	p.adj	p.adj.signif
1	-2.582404	98.12315	0.0113	0.05538462	ns	1	-0.64573352	105.4156	0.52	0.6884615	ns
2	-2.5699523	95.84057	0.0117	0.05538462	ns	2	-0.55019699	106.9277	0.583	0.7197531	ns
3	-2.5553651	93.98041	0.0122	0.05538462	ns	3	-0.46404051	109.0017	0.644	0.7666667	ns
4	-2.5456908	92.60686	0.0126	0.05538462	ns	4	-0.37857166	111.2254	0.706	0.8022727	ns
5	-2.5324033	91.92768	0.013	0.05538462	ns	5	-0.28035807	113.4269	0.78	0.8478261	ns
6	-2.5298207	92.00909	0.0131	0.05538462	ns	6	-0.17355696	115.4503	0.863	0.8989583	ns
7	-2.5248124	92.68342	0.0133	0.05538462	ns	7	-0.05525045	117.3878	0.956	0.9656566	ns
8	-2.5166914	93.74357	0.0135	0.05538462	ns	8	0.06108857	119.0664	0.951	0.9656566	ns
9	-2.492647	95.29308	0.0144	0.05538462	ns	9	0.18397137	120.4409	0.854	0.8989474	ns
10	-2.4289931	97.41613	0.017	0.06071429	ns	10	0.30407073	121.3983	0.762	0.8373626	ns
11	-2.3295278	99.72515	0.0218	0.07517241	ns	11	0.4208118	121.8495	0.675	0.7848837	ns
12	-2.1898084	102.29215	0.0308	0.09625	ns	12	0.5225793	122.0589	0.602	0.7289157	ns
13	-1.988454	104.95296	0.0494	0.1235	ns	13	0.5955391	122.2647	0.553	0.69125	ns
14	-1.6963763	107.64338	0.0927	0.14449275	ns	14	0.64464886	122.4618	0.52	0.6884615	ns
15	-1.3285283	110.30196	0.187	0.22261905	ns	15	0.67914991	122.4576	0.498	0.6884615	ns
16	-0.9250508	112.45282	0.357	0.37978723	ns	16	0.71817783	122.285	0.474	0.6802817	ns
17	-0.5169182	113.51902	0.606	0.62474227	ns	17	0.76034144	121.8878	0.449	0.6602941	ns
18	-0.144053	113.47328	0.886	0.886	ns	18	0.81239839	121.0446	0.418	0.65625	ns
19	0.1758677	112.39047	0.861	0.86969697	ns	19	0.85446676	119.6499	0.395	0.6548387	ns
20	0.4370635	110.57284	0.663	0.67653061	ns	20	0.85558924	117.813	0.394	0.6548387	ns
21	0.6558706	108.4378	0.513	0.534375	ns	21	0.83418882	115.6969	0.406	0.6548387	ns

22	0.8435356	106.4876	0.401	0.42210526	ns	22	0.80927244	113.6474	0.42	0.65625	ns
23	1.0019698	104.69897	0.319	0.35054945	ns	23	0.76950088	112.0706	0.443	0.6602941	ns
24	1.1553805	103.14207	0.251	0.28522727	ns	24	0.70311323	111.0473	0.483	0.6802817	ns
25	1.3123397	102.00518	0.192	0.22588235	ns	25	0.62863187	110.3947	0.531	0.6884615	ns
26	1.4460661	101.1505	0.151	0.18414634	ns	26	0.59830855	110.4365	0.551	0.69125	ns
27	1.5496502	100.63273	0.124	0.155	ns	27	0.65572015	111.5144	0.513	0.6884615	ns
28	1.6491985	100.48315	0.102	0.14571429	ns	28	0.77942549	112.6096	0.437	0.6602941	ns
29	1.7251399	100.20575	0.0876	0.14129032	ns	29	0.92612686	112.8154	0.356	0.6245614	ns
30	1.76161	99.99523	0.0812	0.14129032	ns	30	1.07150255	111.9101	0.286	0.6057692	ns
31	1.7566297	100.55399	0.082	0.14129032	ns	31	1.21962649	110.3047	0.225	0.5846154	ns
32	1.7287613	101.95458	0.0869	0.14129032	ns	32	1.37925372	108.5603	0.171	0.534375	ns
33	1.6736625	103.64104	0.0972	0.14449275	ns	33	1.51699885	107.0366	0.132	0.4888889	ns
34	1.6260634	104.89231	0.107	0.14605263	ns	34	1.59695915	105.6563	0.113	0.4888889	ns
35	1.6078241	105.48854	0.111	0.14605263	ns	35	1.62196477	104.2682	0.108	0.4888889	ns
36	1.6079729	105.35104	0.111	0.14605263	ns	36	1.64405968	102.9103	0.103	0.4888889	ns
37	1.6290523	104.52344	0.106	0.14605263	ns	37	1.68317507	102.075	0.0954	0.4888889	ns
38	1.6751833	103.21394	0.0969	0.14449275	ns	38	1.77518766	102.2969	0.0788	0.4888889	ns
39	1.7263745	101.51206	0.0873	0.14129032	ns	39	1.88857166	103.4592	0.0618	0.4888889	ns
40	1.7855619	99.96089	0.0772	0.14129032	ns	40	1.97218816	104.9691	0.0512	0.4888889	ns
41	1.8710827	99.88803	0.0643	0.13530612	ns	41	1.98621261	106.4474	0.0496	0.4888889	ns
42	2.0329133	102.18979	0.0447	0.11763158	ns	42	1.9079623	107.6043	0.0591	0.4888889	ns
43	2.2520412	105.95829	0.0264	0.088	ns	43	1.75714784	108.7967	0.0817	0.4888889	ns
44	2.4881326	109.78644	0.0143	0.05538462	ns	44	1.58515998	110.0252	0.116	0.4888889	ns
45	2.7188743	112.51475	0.00759	0.0474375	*	45	1.42475086	111.0876	0.157	0.5233333	ns
46	2.9333655	114.41185	0.00405	0.02892857	*	46	1.28456062	111.4179	0.202	0.5771429	ns

47	3.109686	115.91909	0.00236	0.01966667	*	47	1.16883852	110.8254	0.245	0.6057692	ns
48	3.2317652	116.59093	0.0016	0.01454545	*	48	1.09195041	109.6469	0.277	0.6057692	ns
49	3.3103275	116.33237	0.00124	0.01377778	*	49	1.05099679	108.1964	0.296	0.6057692	ns
50	3.3810669	114.59948	0.000988	0.01235	*	50	1.03472447	107.0414	0.303	0.6057692	ns
51	3.464871	111.4351	0.000754	0.01122857	*	51	1.02302756	106.4167	0.309	0.6057692	ns
52	3.5654241	107.83025	0.000543	0.01086	*	52	0.99075921	106.3151	0.324	0.6089286	ns
53	3.6533306	104.69895	0.000406	0.01086	*	53	0.96428669	106.5111	0.337	0.6089286	ns
54	3.710942	102.91133	0.000335	0.01086	*	54	0.96807591	106.6847	0.335	0.6089286	ns
55	3.718057	102.53112	0.000328	0.01086	*	55	1.01080496	107.1559	0.314	0.6057692	ns
56	3.6283908	103.33236	0.000445	0.01086	*	56	1.09845798	108.2726	0.274	0.6057692	ns
57	3.4580816	105.43105	0.000786	0.01122857	*	57	1.21270304	110.0044	0.228	0.5846154	ns
58	3.2410429	108.74821	0.00158	0.01454545	*	58	1.32852869	111.7288	0.187	0.55	ns
59	2.9933911	112.01038	0.0034	0.02615385	*	59	1.44056469	112.8903	0.152	0.5233333	ns
60	2.7224224	114.64244	0.00749	0.0474375	*	60	1.5379852	113.4324	0.127	0.4888889	ns
61	2.4477573	116.73966	0.0159	0.05888889	ns	61	1.60007297	113.0953	0.112	0.4888889	ns
62	2.1952862	118.218	0.0301	0.09625	ns	62	1.62554712	111.8993	0.107	0.4888889	ns
63	1.995048	119.21723	0.0483	0.1235	ns	63	1.62018052	110.1877	0.108	0.4888889	ns
64	1.8633911	120.06234	0.0649	0.13530612	ns	64	1.59815794	108.4718	0.113	0.4888889	ns
65	1.7841292	120.42483	0.0769	0.14129032	ns	65	1.55312625	107.2448	0.123	0.4888889	ns
66	1.7250979	119.97441	0.0871	0.14129032	ns	66	1.47585058	106.8216	0.143	0.5107143	ns
67	1.6856061	118.74098	0.0945	0.14449275	ns	67	1.37798793	107.9077	0.171	0.534375	ns
68	1.6592756	117.35894	0.0997	0.14449275	ns	68	1.23868939	110.8694	0.218	0.5846154	ns
69	1.6310536	116.46619	0.106	0.14605263	ns	69	1.05398937	114.7173	0.294	0.6057692	ns
70	1.5932472	116.40788	0.114	0.14615385	ns	70	0.84457221	117.8973	0.4	0.6548387	ns
71	1.5747356	117.54828	0.118	0.14936709	ns	71	0.62713525	119.5666	0.532	0.6884615	ns

72	1.6054808	120.09015	0.111	0.14605263	ns	72	0.40103407	119.8279	0.689	0.791954	ns
73	1.6630899	123.00089	0.0988	0.14449275	ns	73	0.19870692	119.2734	0.843	0.8968085	ns
74	1.739383	125.30663	0.0844	0.14129032	ns	74	0.02225278	118.485	0.982	0.982	ns
75	1.8533714	126.65174	0.0662	0.13530612	ns	75	-0.12365671	117.7243	0.902	0.9298969	ns
76	1.9221	127.23038	0.0568	0.1352381	ns	76	-0.22933406	117.1375	0.819	0.8806452	ns
77	2.0298089	127.20591	0.0445	0.11763158	ns	77	-0.30334649	116.7688	0.762	0.8373626	ns
78	2.0390403	126.91638	0.0435	0.11763158	ns	78	-0.35392557	116.6173	0.724	0.8134831	ns
79	2.1429729	125.52695	0.034	0.1030303	ns	79	-0.42184771	116.3742	0.674	0.7848837	ns
80	2.1120167	123.75769	0.0367	0.10794118	ns	80	-0.51810826	116.0545	0.605	0.7289157	ns
81	2.036666	121.75205	0.0439	0.11763158	ns	81	-0.61853635	115.9285	0.537	0.6884615	ns
82	1.9515223	120.02203	0.0533	0.13	ns	82	-0.70415599	116.1372	0.483	0.6802817	ns
83	1.8724084	118.76929	0.0636	0.13530612	ns	83	-0.78679014	116.2589	0.433	0.6602941	ns
84	1.670606	119.55667	0.0974	0.14449275	ns	84	-0.87583754	115.9264	0.383	0.6548387	ns
85	1.8099183	118.18067	0.0728	0.14	ns	85	-0.95666176	115.2223	0.341	0.6089286	ns
86	1.828907	118.75959	0.0699	0.1398	ns	86	-1.00904057	114.0308	0.315	0.6057692	ns
87	1.8534862	119.79499	0.0663	0.13530612	ns	87	-1.03791592	112.411	0.302	0.6057692	ns
88	1.8704081	121.22943	0.0638	0.13530612	ns	88	-1.06790494	110.9011	0.288	0.6057692	ns
89	1.8662642	122.83491	0.0644	0.13530612	ns	89	-1.12108218	110.0284	0.265	0.6057692	ns
90	1.8189553	124.21268	0.0713	0.13980392	ns	90	-1.21793883	109.9641	0.226	0.5846154	ns
91	1.7293032	125.17827	0.0862	0.14129032	ns	91	-1.35387914	110.0981	0.179	0.5424242	ns
92	1.595905	125.78727	0.113	0.14615385	ns	92	-1.52871733	108.9994	0.129	0.4888889	ns
93	1.4642743	126.00925	0.146	0.18024691	ns	93	-1.70431015	106.6678	0.0912	0.4888889	ns
94	1.3660286	125.85607	0.174	0.20963855	ns	94	-1.82118586	104.5182	0.0714	0.4888889	ns
95	1.2849261	125.3615	0.201	0.23372093	ns	95	-1.84445784	103.7658	0.068	0.4888889	ns
96	1.2095299	124.51927	0.229	0.26321839	ns	96	-1.78951691	104.9185	0.0764	0.4888889	ns

97	1.1332991	123.16046	0.259	0.29101124	ns	97	-1.71100887	107.4738	0.09	0.4888889	ns
98	1.0397575	120.6878	0.301	0.33444444	ns	98	-1.65084466	110.7561	0.102	0.4888889	ns
99	0.9804517	117.36817	0.329	0.35376344	ns	99	-1.61711994	114.6622	0.109	0.4888889	ns
100	0.9923167	114.09322	0.323	0.35108696	ns	100	-1.60951211	118.0964	0.11	0.4888889	ns
3	statistic	df	p	p.adj	p.adj.signif	4	statistic	df	p	p.adj	p.adj.signif
1	-1.07114767	127.6648	2.86E-01	3.29E-01	ns	1	-1.09711347	119.62942	0.275	0.5745098	ns
2	-1.02599316	127.7779	3.07E-01	3.45E-01	ns	2	-0.98038363	119.32203	0.329	0.5981818	ns
3	-0.72001933	127.6672	4.73E-01	5.14E-01	ns	3	-0.92807748	118.61228	0.355	0.612069	ns
4	-0.59903772	127.5224	5.50E-01	5.91E-01	ns	4	-0.94430835	118.39425	0.347	0.612069	ns
5	-0.42316553	127.2782	6.73E-01	7.01E-01	ns	5	-1.00982835	119.9322	0.315	0.5943396	ns
6	-0.21061768	126.9602	8.34E-01	8.42E-01	ns	6	-1.06416157	122.47843	0.289	0.5745098	ns
7	0.04449981	127.0143	9.65E-01	9.65E-01	ns	7	-0.98979297	124.36899	0.324	0.5981818	ns
8	0.30688756	127.2232	7.59E-01	7.74E-01	ns	8	-0.89483783	125.6094	0.373	0.6216667	ns
9	0.59045875	127.4781	5.56E-01	5.91E-01	ns	9	-0.69453939	126.36204	0.489	0.7523077	ns
10	0.8478926	127.587	3.98E-01	4.42E-01	ns	10	-0.39183066	126.58854	0.696	0.9012821	ns
11	1.05627407	127.3177	2.93E-01	3.33E-01	ns	11	-0.08813806	126.73894	0.93	0.957	ns
12	1.27251438	126.7222	2.06E-01	2.42E-01	ns	12	0.18894454	126.72098	0.85	0.9223404	ns
13	1.46573028	125.541	1.45E-01	1.75E-01	ns	13	0.49205655	126.08945	0.624	0.8666667	ns
14	1.56790607	123.4408	1.19E-01	1.45E-01	ns	14	0.78304332	125.41199	0.435	0.6936508	ns
15	1.64857267	120.8524	1.02E-01	1.26E-01	ns	15	1.05497166	124.77222	0.293	0.5745098	ns
16	1.79802139	118.0706	7.47E-02	9.46E-02	ns	16	1.27461288	124.17458	0.205	0.5745098	ns
17	2.06087087	115.7402	4.16E-02	5.40E-02	ns	17	1.46627478	124.57646	0.145	0.5745098	ns
18	2.43046027	115.7444	1.66E-02	2.24E-02	*	18	1.68570178	125.52976	0.0943	0.5745098	ns
19	2.85982343	117.7275	5.02E-03	7.72E-03	**	19	1.97167117	124.94749	0.0509	0.5745098	ns
20	3.3044154	120.2042	1.25E-03	2.55E-03	**	20	2.1561711	123.73838	0.033	0.5745098	ns

21	3.7328801	122.2494	2.89E-04	9.90E-04	***	21	2.24369232	121.8588	0.0267	0.5745098	ns
22	4.09867021	123.5357	7.47E-05	3.11E-04	***	22	2.32235405	120.57838	0.0219	0.5745098	ns
23	4.38259701	124.168	2.47E-05	1.12E-04	***	23	2.3602326	121.23067	0.0199	0.5745098	ns
24	4.56062227	124.5164	1.20E-05	5.71E-05	****	24	2.26188828	121.82929	0.0255	0.5745098	ns
25	4.61017457	124.5074	9.82E-06	4.91E-05	****	25	2.01259649	120.04255	0.0464	0.5745098	ns
26	4.77682707	124.4367	4.92E-06	2.73E-05	****	26	1.71419237	118.38744	0.0891	0.5745098	ns
27	4.77993491	123.8145	4.88E-06	2.73E-05	****	27	1.48991854	118.06249	0.139	0.5745098	ns
28	4.93522314	123.2233	2.54E-06	1.59E-05	****	28	1.29661223	119.61864	0.197	0.5745098	ns
29	5.13939338	123.0667	1.05E-06	7.00E-06	****	29	1.14152683	121.85593	0.256	0.5745098	ns
30	5.34508372	123.2265	4.22E-07	3.25E-06	****	30	1.10568401	123.73006	0.271	0.5745098	ns
31	5.62471108	123.1415	1.18E-07	1.31E-06	****	31	1.19434681	125.12429	0.235	0.5745098	ns
32	5.55820609	122.5842	1.62E-07	1.39E-06	****	32	1.25788145	125.88415	0.211	0.5745098	ns
33	5.55407014	121.4632	1.67E-07	1.39E-06	****	33	1.21300197	125.22251	0.227	0.5745098	ns
34	5.66965453	120.0324	1.00E-07	1.25E-06	****	34	1.12022395	123.35457	0.265	0.5745098	ns
35	5.70358365	119.1091	8.70E-08	1.24E-06	****	35	1.0160598	121.21056	0.312	0.5943396	ns
36	5.8563701	119.2359	4.27E-08	8.45E-07	****	36	1.17563411	120.94992	0.242	0.5745098	ns
37	6.05523306	119.9619	1.65E-08	4.60E-07	****	37	0.76153848	119.73945	0.448	0.7	ns
38	6.18105388	120.6858	8.94E-09	4.60E-07	****	38	0.60238281	119.01819	0.548	0.8058824	ns
39	6.0283128	121.2968	1.84E-08	4.60E-07	****	39	0.4507883	118.25564	0.653	0.8824324	ns
40	6.04221846	120.648	1.74E-08	4.60E-07	****	40	0.5792269	119.47628	0.564	0.8173913	ns
41	5.81673938	120.1908	5.07E-08	8.45E-07	****	41	0.40840891	121.13014	0.684	0.9012821	ns
42	5.56196216	120.554	1.63E-07	1.39E-06	****	42	0.31384852	122.57201	0.754	0.9151163	ns
43	5.25041887	120.7446	6.60E-07	4.71E-06	****	43	0.27278436	123.89075	0.785	0.9151163	ns
44	4.68356887	120.0967	7.49E-06	3.94E-05	****	44	0.16895252	124.84336	0.866	0.9223404	ns
45	4.34015548	119.4687	3.00E-05	1.30E-04	***	45	-0.0732494	125.41578	0.942	0.957	ns

46	4.04697137	119.385	9.25E-05	3.70E-04	***	46	-0.3466608	125.20685	0.729	0.9123457	ns
47	3.80832042	119.421	2.22E-04	8.54E-04	***	47	-0.87624589	123.72692	0.383	0.6278689	ns
48	3.64807982	118.8045	3.94E-04	1.19E-03	**	48	-1.06215827	122.89004	0.29	0.5745098	ns
49	3.56359769	118.2078	5.29E-04	1.42E-03	**	49	-1.18561499	120.9038	0.238	0.5745098	ns
50	3.52372625	118.2369	6.06E-04	1.52E-03	**	50	-1.22902678	117.87845	0.222	0.5745098	ns
51	3.48067995	118.9006	7.00E-04	1.71E-03	**	51	-1.19877614	114.24525	0.233	0.5745098	ns
52	3.37915307	120.2384	9.81E-04	2.13E-03	**	52	-1.12686567	110.84517	0.262	0.5745098	ns
53	3.20990317	121.3498	1.70E-03	3.33E-03	**	53	-1.08987809	108.69049	0.278	0.5745098	ns
54	3.02360825	122.0121	3.05E-03	5.45E-03	**	54	-1.06326647	108.02092	0.29	0.5745098	ns
55	2.88155276	122.1232	4.68E-03	7.43E-03	**	55	-1.06513179	109.00545	0.289	0.5745098	ns
56	2.81333336	121.2046	5.72E-03	8.54E-03	**	56	-1.08871306	110.51264	0.279	0.5745098	ns
57	2.83405748	119.6357	5.40E-03	8.18E-03	**	57	-1.14011869	110.95376	0.257	0.5745098	ns
58	2.90619858	117.704	4.37E-03	7.16E-03	**	58	-1.2085264	110.30224	0.229	0.5745098	ns
59	2.98634693	115.6856	3.45E-03	5.95E-03	**	59	-1.26996452	108.65241	0.207	0.5745098	ns
60	3.03824738	114.3308	2.95E-03	5.36E-03	**	60	-1.34066274	105.64467	0.183	0.5745098	ns
61	3.05733339	113.8176	2.78E-03	5.15E-03	**	61	-1.36352591	102.78951	0.176	0.5745098	ns
62	3.01567817	112.8747	3.17E-03	5.56E-03	**	62	-1.31432081	100.69722	0.192	0.5745098	ns
63	2.89387318	110.7772	4.58E-03	7.39E-03	**	63	-1.21762819	99.50026	0.226	0.5745098	ns
64	2.75099245	108.1272	6.97E-03	1.01E-02	*	64	-1.08182048	99.74955	0.282	0.5745098	ns
65	2.64180026	106.0785	9.49E-03	1.34E-02	*	65	-0.92937369	100.75523	0.355	0.612069	ns
66	2.60655169	105.4231	1.05E-02	1.44E-02	*	66	-0.78046865	102.73055	0.437	0.6936508	ns
67	2.69201108	105.9629	8.26E-03	1.18E-02	*	67	-0.64074014	106.09315	0.523	0.7850746	ns
68	2.91505248	107.0979	4.33E-03	7.16E-03	**	68	-0.56041483	108.93975	0.576	0.8228571	ns
69	3.19027691	107.8396	1.86E-03	3.58E-03	**	69	-0.53383755	111.33779	0.595	0.8380282	ns
70	3.44437448	108.5324	8.15E-04	1.85E-03	**	70	-0.47948501	114.83906	0.633	0.8671233	ns

71	3.61471267	109.4795	4.56E-04	1.34E-03	**	71	-0.38220246	119.38529	0.703	0.9012821	ns
72	3.69857036	111.1479	3.39E-04	1.09E-03	**	72	-0.27143932	122.60387	0.787	0.9151163	ns
73	3.75545871	113.6333	2.75E-04	9.82E-04	***	73	-0.16774795	123.65801	0.867	0.9223404	ns
74	3.76191387	115.7787	2.66E-04	9.82E-04	***	74	-0.05898418	123.58922	0.953	0.957	ns
75	3.72977637	117.5206	2.97E-04	9.90E-04	***	75	0.08069475	123.95638	0.936	0.957	ns
76	3.66718283	118.8283	3.68E-04	1.15E-03	**	76	0.17879383	124.06973	0.858	0.9223404	ns
77	3.55998917	119.5105	5.33E-04	1.42E-03	**	77	0.21724682	123.69718	0.828	0.9223404	ns
78	3.43501538	119.786	8.15E-04	1.85E-03	**	78	0.24068176	123.06173	0.81	0.9223404	ns
79	3.35573071	119.996	1.06E-03	2.21E-03	**	79	0.28978236	122.70469	0.772	0.9151163	ns
80	3.37202984	120.5053	1.00E-03	2.13E-03	**	80	0.33400113	122.44529	0.739	0.9123457	ns
81	3.45304502	121.023	7.64E-04	1.82E-03	**	81	0.33625623	122.13783	0.737	0.9123457	ns
82	3.53984615	121.1623	5.69E-04	1.46E-03	**	82	0.27690369	122.03207	0.782	0.9151163	ns
83	3.59426891	121.222	4.71E-04	1.35E-03	**	83	0.1866603	122.44485	0.852	0.9223404	ns
84	3.55458137	121.3067	5.41E-04	1.42E-03	**	84	0.07755974	122.83268	0.938	0.957	ns
85	3.41006654	121.2791	8.83E-04	1.96E-03	**	85	-0.0540636	122.88755	0.957	0.957	ns
86	3.22245233	121.2185	1.63E-03	3.26E-03	**	86	-0.21074939	122.80448	0.833	0.9223404	ns
87	3.06117422	121.4111	2.71E-03	5.11E-03	**	87	-0.39771993	122.79897	0.692	0.9012821	ns
88	2.94339104	121.822	3.89E-03	6.59E-03	**	88	-0.63646346	122.63845	0.526	0.7850746	ns
89	2.8625705	121.9683	4.95E-03	7.72E-03	**	89	-0.91165309	122.08185	0.364	0.6169492	ns
90	2.77291807	121.4452	6.43E-03	9.46E-03	**	90	-1.14506011	121.09423	0.254	0.5745098	ns
91	2.6147204	120.2276	1.01E-02	1.40E-02	*	91	-1.28347282	120.01172	0.202	0.5745098	ns
92	2.39233733	118.5344	1.83E-02	2.44E-02	*	92	-1.33528037	118.63548	0.184	0.5745098	ns
93	2.14550868	116.3165	3.40E-02	4.47E-02	*	93	-1.33036478	116.76185	0.186	0.5745098	ns
94	1.89408549	113.3118	6.08E-02	7.79E-02	ns	94	-1.27999771	114.96874	0.203	0.5745098	ns
95	1.64691956	109.9663	1.02E-01	1.26E-01	ns	95	-1.22942131	113.5456	0.221	0.5745098	ns

96	1.38328251	107.0411	1.69E-01	2.01E-01	ns	96	-1.23510909	112.25995	0.219	0.5745098	ns
97	1.09087037	104.8983	2.78E-01	3.23E-01	ns	97	-1.3097772	111.05161	0.193	0.5745098	ns
98	0.80216705	103.2438	4.24E-01	4.66E-01	ns	98	-1.40272489	110.09728	0.164	0.5745098	ns
99	0.56063802	101.9985	5.76E-01	6.06E-01	ns	99	-1.51758566	108.94037	0.132	0.5745098	ns
100	0.39366453	101.1944	6.95E-01	7.16E-01	ns	100	-1.64477741	107.29337	0.103	0.5745098	ns
5	statistic	df	p	p.adj	p.adj.signif	6	statistic	df	p	p.adj	p.adj.signif
1	-0.38937051	93.27177	6.98E-01	0.935526316	ns	1	0.017094202	112.02808	0.986	0.994	ns
2	-0.12556525	92.92494	9.00E-01	0.999	ns	2	-0.20960845	113.07192	0.834	0.9863636	ns
3	0.425152631	93.16016	6.72E-01	0.933333333	ns	3	-0.51232238	110.25876	0.609	0.9863636	ns
4	0.855488399	94.29408	3.94E-01	0.703571429	ns	4	-0.59059444	110.00852	0.556	0.9863636	ns
5	0.917380838	95.98053	3.61E-01	0.668518519	ns	5	-0.55917333	106.3206	0.577	0.9863636	ns
6	0.819355569	99.44169	4.15E-01	0.728070175	ns	6	-0.38404793	100.07771	0.702	0.9863636	ns
7	0.703424159	104.50667	4.83E-01	0.78125	ns	7	-0.28182546	95.79073	0.779	0.9863636	ns
8	0.445686622	108.13617	6.57E-01	0.933333333	ns	8	-0.36284659	95.98889	0.718	0.9863636	ns
9	0.145019972	109.00244	8.85E-01	0.999	ns	9	-0.39300649	97.22918	0.695	0.9863636	ns
10	0.000903787	109.08806	9.99E-01	0.999	ns	10	-0.08670307	99.39272	0.931	0.9884211	ns
11	-0.07463227	108.63641	9.41E-01	0.999	ns	11	0.317436645	104.81699	0.752	0.9863636	ns
12	-0.21577776	106.92227	8.30E-01	0.999	ns	12	0.583658832	111.286	0.561	0.9863636	ns
13	-0.24498548	106.35382	8.07E-01	0.999	ns	13	0.673815306	115.09755	0.502	0.9863636	ns
14	-0.13087992	108.28965	8.96E-01	0.999	ns	14	0.550956323	117.0604	0.583	0.9863636	ns
15	-0.00884303	110.86165	9.93E-01	0.999	ns	15	0.322369127	117.83066	0.748	0.9863636	ns
16	0.057966542	112.26937	9.54E-01	0.999	ns	16	0.181807168	117.32599	0.856	0.9863636	ns
17	0.189390982	110.83215	8.50E-01	0.999	ns	17	-0.00692523	114.243	0.994	0.994	ns
18	0.372017798	107.33373	7.11E-01	0.935526316	ns	18	-0.30580377	110.20862	0.76	0.9863636	ns
19	0.585218843	104.22546	5.60E-01	0.848484848	ns	19	-0.65340026	106.63545	0.515	0.9863636	ns

20	0.788169295	103.98275	4.32E-01	0.744827586	ns	20	-0.88504864	102.52369	0.378	0.9863636	ns
21	1.007739294	106.92061	3.16E-01	0.609433962	ns	21	-0.95376756	102.97611	0.342	0.9863636	ns
22	1.179588228	111.5626	2.41E-01	0.502083333	ns	22	-0.83750075	105.89725	0.404	0.9863636	ns
23	1.347994836	114.68948	1.80E-01	0.382978723	ns	23	-0.43917747	107.97704	0.661	0.9863636	ns
24	1.648465851	115.49819	1.02E-01	0.242857143	ns	24	0.019422068	108.16838	0.985	0.994	ns
25	2.003167984	114.28498	4.75E-02	0.128378378	ns	25	0.402154064	107.98576	0.688	0.9863636	ns
26	2.326836181	113.94292	2.17E-02	0.065757576	ns	26	0.591295946	108.78833	0.556	0.9863636	ns
27	2.644187911	114.37646	9.34E-03	0.032206897	*	27	0.701379787	111.09056	0.485	0.9863636	ns
28	2.833920156	114.98116	5.43E-03	0.02172	*	28	0.762609937	115.05067	0.447	0.9863636	ns
29	2.81999396	115.5193	5.65E-03	0.021730769	*	29	0.701170029	117.35634	0.485	0.9863636	ns
30	2.55877063	114.86707	1.18E-02	0.039333333	*	30	0.572665899	116.99224	0.568	0.9863636	ns
31	2.225605159	110.67609	2.81E-02	0.082647059	ns	31	0.586883435	116.17052	0.558	0.9863636	ns
32	1.856398872	106.30018	6.62E-02	0.174210526	ns	32	0.732051336	117.38887	0.466	0.9863636	ns
33	1.656373632	104.28685	1.01E-01	0.242857143	ns	33	0.865312862	120.1269	0.389	0.9863636	ns
34	1.589524118	106.60295	1.15E-01	0.261363636	ns	34	0.860204376	122.12874	0.391	0.9863636	ns
35	1.561063649	111.62473	1.21E-01	0.268888889	ns	35	0.851167009	123.17925	0.396	0.9863636	ns
36	1.606621944	115.32278	1.11E-01	0.258139535	ns	36	0.992717094	124.16125	0.323	0.9863636	ns
37	1.767319816	117.9224	7.98E-02	0.1995	ns	37	1.35394332	124.52698	0.178	0.6137931	ns
38	2.075996407	119.64366	4.00E-02	0.111111111	ns	38	1.722768178	124.25406	0.0874	0.3237037	ns
39	2.393143675	119.99368	1.83E-02	0.0571875	ns	39	1.971492253	122.92843	0.0509	0.242381	ns
40	2.657434895	118.48756	8.96E-03	0.032	*	40	2.03235108	119.63267	0.0443	0.2215	ns
41	2.884840639	117.24573	4.66E-03	0.019416667	*	41	1.944474619	116.45242	0.0542	0.2463636	ns
42	3.085977041	118.24118	2.53E-03	0.012047619	*	42	1.883091182	115.43124	0.0622	0.2591667	ns
43	3.156616716	121.5855	2.01E-03	0.01025	*	43	1.856381412	116.3037	0.0659	0.2636	ns
44	3.247620928	124.06036	1.50E-03	0.008333333	**	44	1.900216205	117.3203	0.0599	0.2591667	ns

45	3.468292771	124.77012	7.19E-04	0.004229412	**	45	2.094074772	118.38114	0.0384	0.2021053	ns
46	3.753383287	123.80358	2.67E-04	0.002483333	**	46	2.340766675	119.0998	0.0209	0.1675	ns
47	4.013567271	120.57487	1.04E-04	0.00216	**	47	2.425195775	119.69828	0.0168	0.1675	ns
48	4.161333995	116.06459	6.10E-05	0.00216	**	48	2.438291384	120.95493	0.0162	0.1675	ns
49	4.150411204	114.34735	6.41E-05	0.00216	**	49	2.378097332	120.19623	0.019	0.1675	ns
50	4.03244215	114.71516	9.98E-05	0.00216	**	50	2.255264882	116.61816	0.026	0.1675	ns
51	3.922264571	115.56523	1.49E-04	0.002171429	**	51	2.215992874	113.53094	0.0287	0.1688235	ns
52	3.821225081	115.92307	2.15E-04	0.002433333	**	52	2.2434269	113.42538	0.0268	0.1675	ns
53	3.731448324	116.45386	2.96E-04	0.002483333	**	53	2.281572016	114.43817	0.0244	0.1675	ns
54	3.664228718	117.27303	3.74E-04	0.002876923	**	54	2.306575313	116.30842	0.0228	0.1675	ns
55	3.573227	117.90732	5.12E-04	0.003413333	**	55	2.341188651	118.26784	0.0209	0.1675	ns
56	3.586655978	118.5006	4.88E-04	0.003413333	**	56	2.389534452	119.59397	0.0184	0.1675	ns
57	3.726174831	119.83997	2.98E-04	0.002483333	**	57	2.479037221	120.36484	0.0146	0.1675	ns
58	3.910986383	121.24923	1.52E-04	0.002171429	**	58	2.597880798	120.48042	0.0105	0.1675	ns
59	4.00226708	122.93263	1.08E-04	0.00216	**	59	2.621532465	118.93606	0.0099	0.1675	ns
60	3.807908546	123.72813	2.19E-04	0.002433333	**	60	2.584079587	115.90948	0.011	0.1675	ns
61	3.472442025	122.85552	7.13E-04	0.004229412	**	61	2.547086065	112.1547	0.0122	0.1675	ns
62	3.152531346	119.8668	2.05E-03	0.01025	*	62	2.4352347	107.6072	0.0165	0.1675	ns
63	2.998906105	115.95605	3.32E-03	0.015090909	*	63	2.195474834	102.69437	0.0304	0.1688889	ns
64	2.915443994	111.9578	4.29E-03	0.018652174	*	64	1.84040345	100.00758	0.0687	0.2642308	ns
65	2.751527945	107.62088	6.96E-03	0.025777778	*	65	1.360897084	98.86439	0.177	0.6137931	ns
66	2.500735756	104.74449	1.39E-02	0.04483871	*	66	0.79637254	98.6503	0.428	0.9863636	ns
67	2.202730659	103.46297	2.98E-02	0.085142857	ns	67	0.271943479	99.37816	0.786	0.9863636	ns
68	1.813647376	104.83549	7.26E-02	0.186153846	ns	68	-0.05710774	101.60932	0.955	0.994	ns
69	1.425749455	107.79183	1.57E-01	0.341304348	ns	69	-0.19228439	103.61059	0.848	0.9863636	ns

70	1.151623023	109.91507	2.52E-01	0.514285714	ns	70	-0.19641178	104.6575	0.845	0.9863636	ns
71	1.021250258	109.8592	3.09E-01	0.609433962	ns	71	-0.1933971	104.8669	0.847	0.9863636	ns
72	1.000784024	107.34011	3.19E-01	0.609433962	ns	72	-0.08242757	105.91689	0.934	0.9884211	ns
73	0.993657372	103.68819	3.23E-01	0.609433962	ns	73	0.077303645	109.60005	0.939	0.9884211	ns
74	0.855935049	100.85768	3.94E-01	0.703571429	ns	74	0.166464684	112.71742	0.868	0.9863636	ns
75	0.5588411	99.60356	5.78E-01	0.857352941	ns	75	0.166584568	114.86264	0.868	0.9863636	ns
76	0.400153385	101.35158	6.90E-01	0.935526316	ns	76	0.187106289	116.62827	0.852	0.9863636	ns
77	0.431056062	104.5337	6.67E-01	0.933333333	ns	77	0.212612647	116.14711	0.832	0.9863636	ns
78	0.485205571	109.36077	6.29E-01	0.911594203	ns	78	0.16977913	113.40026	0.865	0.9863636	ns
79	0.37094495	113.45097	7.11E-01	0.935526316	ns	79	0.108826368	110.17424	0.914	0.9884211	ns
80	0.188303261	115.10191	8.51E-01	0.999	ns	80	-0.03716602	108.03626	0.97	0.994	ns
81	0.006573762	114.40648	9.95E-01	0.999	ns	81	-0.17014076	108.71556	0.865	0.9863636	ns
82	-0.15639932	112.52712	8.76E-01	0.999	ns	82	-0.24893773	109.7723	0.804	0.9863636	ns
83	-0.19242653	110.54203	8.48E-01	0.999	ns	83	-0.34589591	109.49735	0.73	0.9863636	ns
84	-0.18318674	108.60744	8.55E-01	0.999	ns	84	-0.49670558	108.2565	0.62	0.9863636	ns
85	-0.12784468	107.26636	8.99E-01	0.999	ns	85	-0.59199217	107.29189	0.555	0.9863636	ns
86	-0.04995173	107.00769	9.60E-01	0.999	ns	86	-0.58091424	107.45606	0.563	0.9863636	ns
87	-0.02079728	108.70688	9.83E-01	0.999	ns	87	-0.55090858	108.45782	0.583	0.9863636	ns
88	-0.03117007	110.89508	9.75E-01	0.999	ns	88	-0.48816248	108.70198	0.626	0.9863636	ns
89	-0.03454241	113.19032	9.73E-01	0.999	ns	89	-0.35140724	108.70799	0.726	0.9863636	ns
90	-0.04312687	115.62342	9.66E-01	0.999	ns	90	-0.22978718	109.17731	0.819	0.9863636	ns
91	-0.08374593	116.70759	9.33E-01	0.999	ns	91	-0.08647909	110.31827	0.931	0.9884211	ns
92	-0.13961255	116.03088	8.89E-01	0.999	ns	92	0.086207629	111.24624	0.931	0.9884211	ns
93	-0.31313032	114.12062	7.55E-01	0.980519481	ns	93	0.238913284	111.77298	0.812	0.9863636	ns
94	-0.54997871	112.9391	5.83E-01	0.857352941	ns	94	0.328807435	112.27058	0.743	0.9863636	ns

95	-0.68067651	114.4525	4.97E-01	0.78125	ns	95	0.414229398	111.52348	0.68	0.9863636	ns
96	-0.71383691	116.6571	4.77E-01	0.78125	ns	96	0.425769288	109.96087	0.671	0.9863636	ns
97	-0.71307074	117.8811	4.77E-01	0.78125	ns	97	0.352982794	109.20398	0.725	0.9863636	ns
98	-0.68521115	116.78207	4.95E-01	0.78125	ns	98	0.297402341	108.32814	0.767	0.9863636	ns
99	-0.67722726	114.24612	5.00E-01	0.78125	ns	99	0.206220018	108.70643	0.837	0.9863636	ns
100	-0.61220263	111.40767	5.42E-01	0.833846154	ns	100	0.092096066	109.99589	0.927	0.9884211	ns
7	statistic	df	p	p.adj	p.adj.signif	8	statistic	df	p	p.adj	p.adj.signif
1	1.92754242	118.04509	0.0563	0.1948276	ns	1	-0.79161249	117.4157	0.43	0.9345679	ns
2	1.79669844	117.09096	0.075	0.2272727	ns	2	-0.7603701	114.88605	0.449	0.9345679	ns
3	1.77158066	117.40342	0.0791	0.2326471	ns	3	-0.6092107	112.28698	0.544	0.9345679	ns
4	1.81682629	118.19054	0.0718	0.224375	ns	4	-0.41519371	109.71706	0.679	0.9345679	ns
5	1.8757898	119.46572	0.0631	0.2103333	ns	5	-0.31081588	107.35889	0.757	0.9345679	ns
6	1.92576292	121.59071	0.0565	0.1948276	ns	6	-0.32867459	105.74449	0.743	0.9345679	ns
7	1.97658643	123.52172	0.0503	0.1948276	ns	7	-0.43328784	103.98374	0.666	0.9345679	ns
8	1.97112829	124.65887	0.0509	0.1948276	ns	8	-0.60918021	102.21187	0.544	0.9345679	ns
9	1.93889424	124.51055	0.0548	0.1948276	ns	9	-0.80344591	100.45757	0.424	0.9345679	ns
10	1.93282314	123.50382	0.0555	0.1948276	ns	10	-0.92268978	99.18511	0.358	0.9345679	ns
11	2.00752177	122.78663	0.0469	0.1948276	ns	11	-0.94200307	98.6487	0.348	0.9345679	ns
12	2.14452981	122.15853	0.034	0.173	ns	12	-0.88832187	98.64528	0.377	0.9345679	ns
13	2.3410853	121.38187	0.0209	0.13625	ns	13	-0.78549477	100.6335	0.434	0.9345679	ns
14	2.53068811	119.89275	0.0127	0.1154545	ns	14	-0.65761928	103.92806	0.512	0.9345679	ns
15	2.65198196	117.99509	0.0091	0.115	ns	15	-0.55303767	106.84786	0.581	0.9345679	ns
16	2.713245	116.01797	0.00768	0.115	ns	16	-0.49317472	109.37438	0.623	0.9345679	ns
17	2.77907962	115.01558	0.00637	0.115	ns	17	-0.49426723	111.54358	0.622	0.9345679	ns
18	2.80617488	113.56413	0.0059	0.115	ns	18	-0.55088832	112.4682	0.583	0.9345679	ns

19	2.74711236	111.83043	0.00701	0.115	ns	19	-0.64210555	112.83728	0.522	0.9345679	ns
20	2.68209084	111.37482	0.00843	0.115	ns	20	-0.69570732	114.00951	0.488	0.9345679	ns
21	2.57789461	112.76732	0.0112	0.115	ns	21	-0.69650164	116.6066	0.488	0.9345679	ns
22	2.44693421	115.26914	0.0159	0.1171429	ns	22	-0.69081096	118.84406	0.491	0.9345679	ns
23	2.26915249	117.10603	0.0251	0.1476471	ns	23	-0.69343848	119.93201	0.489	0.9345679	ns
24	2.03548779	117.68898	0.044	0.1948276	ns	24	-0.67363747	120.66953	0.502	0.9345679	ns
25	1.82209382	118.54725	0.071	0.224375	ns	25	-0.66072069	120.12263	0.51	0.9345679	ns
26	1.68597455	120.47376	0.0944	0.2551351	ns	26	-0.67034744	118.46456	0.504	0.9345679	ns
27	1.60858417	122.7121	0.11	0.2894737	ns	27	-0.66355842	116.70215	0.508	0.9345679	ns
28	1.55947343	124.54313	0.121	0.3102564	ns	28	-0.57536509	116.88534	0.566	0.9345679	ns
29	1.52188133	125.60483	0.131	0.3119048	ns	29	-0.49796516	118.3679	0.619	0.9345679	ns
30	1.46324003	125.9882	0.146	0.3282609	ns	30	-0.47160083	119.57609	0.638	0.9345679	ns
31	1.40143204	125.5718	0.164	0.3346939	ns	31	-0.50236208	120.41007	0.616	0.9345679	ns
32	1.33591219	124.2769	0.184	0.3607843	ns	32	-0.49119895	121.36977	0.624	0.9345679	ns
33	1.30354935	122.42223	0.195	0.3679245	ns	33	-0.50352052	122.29959	0.616	0.9345679	ns
34	1.31159191	119.98356	0.192	0.3679245	ns	34	-0.52254661	122.46179	0.602	0.9345679	ns
35	1.36832791	117.35291	0.174	0.348	ns	35	-0.46237078	123.07136	0.645	0.9345679	ns
36	1.44706134	115.05247	0.151	0.3282609	ns	36	-0.38063936	124.05316	0.704	0.9345679	ns
37	1.50232867	112.12802	0.136	0.3162791	ns	37	-0.35971262	124.64034	0.72	0.9345679	ns
38	1.53537997	108.65806	0.128	0.3119048	ns	38	-0.36157565	124.21832	0.718	0.9345679	ns
39	1.52900082	105.06235	0.129	0.3119048	ns	39	-0.3600119	122.12134	0.719	0.9345679	ns
40	1.44619559	100.85488	0.151	0.3282609	ns	40	-0.31630432	119.11778	0.752	0.9345679	ns
41	1.26381899	95.54872	0.209	0.387037	ns	41	-0.22568834	115.88717	0.822	0.99036145	ns
42	1.08456974	90.67356	0.281	0.4622951	ns	42	-0.1279393	113.74912	0.898	0.997	ns
43	1.00347334	89.29447	0.318	0.4984375	ns	43	-0.06616619	112.72715	0.947	0.997	ns

44	0.95555395	89.93976	0.342	0.5164179	ns	44	-0.02258457	112.54793	0.982	0.997	ns
45	0.91386952	91.44577	0.363	0.526087	ns	45	0.028547952	113.0114	0.977	0.997	ns
46	0.85188905	92.88935	0.396	0.5424658	ns	46	0.090012617	113.43358	0.928	0.997	ns
47	0.82505297	94.92123	0.411	0.5533333	ns	47	0.117232602	112.92867	0.907	0.997	ns
48	0.85685359	97.39793	0.394	0.5424658	ns	48	0.080020201	112.01715	0.936	0.997	ns
49	0.91394882	99.33926	0.363	0.526087	ns	49	0.003451731	110.94981	0.997	0.997	ns
50	1.00071642	101.0276	0.319	0.4984375	ns	50	-0.0119207	112.44258	0.991	0.997	ns
51	1.15384801	102.61422	0.251	0.4403509	ns	51	0.018613924	114.03046	0.985	0.997	ns
52	1.40117601	104.40627	0.164	0.3346939	ns	52	0.05236879	113.41683	0.958	0.997	ns
53	1.74994179	108.30347	0.083	0.2371429	ns	53	0.07022885	111.23509	0.944	0.997	ns
54	2.13853036	112.86011	0.0346	0.173	ns	54	0.069700943	108.9056	0.945	0.997	ns
55	2.43538352	115.70861	0.0164	0.1171429	ns	55	0.041312402	107.502	0.967	0.997	ns
56	2.61054263	116.71898	0.0102	0.115	ns	56	0.020619536	107.67824	0.984	0.997	ns
57	2.63179219	116.59293	0.00964	0.115	ns	57	-0.03577809	108.56867	0.972	0.997	ns
58	2.56682236	115.87496	0.0115	0.115	ns	58	-0.14455303	109.16713	0.885	0.997	ns
59	2.4697514	115.25098	0.015	0.1171429	ns	59	-0.28215141	109.63292	0.778	0.94878049	ns
60	2.32618516	114.75744	0.0218	0.13625	ns	60	-0.41862575	109.86908	0.676	0.9345679	ns
61	2.15995329	113.91264	0.0329	0.173	ns	61	-0.50296442	110.25428	0.616	0.9345679	ns
62	1.95405565	111.94292	0.0532	0.1948276	ns	62	-0.51985253	111.7645	0.604	0.9345679	ns
63	1.71800615	108.415	0.0887	0.2463889	ns	63	-0.47926984	113.75484	0.633	0.9345679	ns
64	1.42335631	103.98759	0.158	0.3346939	ns	64	-0.40753055	115.9597	0.684	0.9345679	ns
65	1.08116284	99.51332	0.282	0.4622951	ns	65	-0.36962837	118.65351	0.712	0.9345679	ns
66	0.71451204	95.32441	0.477	0.6194805	ns	66	-0.34326646	121.49601	0.732	0.9345679	ns
67	0.35111489	90.37726	0.726	0.8642857	ns	67	-0.36975336	123.46875	0.712	0.9345679	ns
68	0.030975	84.75196	0.975	0.9919192	ns	68	-0.51785508	124.39803	0.605	0.9345679	ns

69	-0.22787776	80.1949	0.82	0.9534884	ns	69	-0.781732	124.72948	0.436	0.9345679	ns
70	-0.42873252	77.661	0.669	0.8060241	ns	70	-1.08338902	124.49125	0.281	0.90645161	ns
71	-0.55802105	77.42899	0.578	0.7225	ns	71	-1.34805756	123.64333	0.18	0.6	ns
72	-0.68617235	78.89761	0.495	0.6346154	ns	72	-1.58578906	122.6431	0.115	0.39655172	ns
73	-0.81856648	80.00083	0.415	0.5533333	ns	73	-1.82348596	121.56788	0.0707	0.27	ns
74	-0.97201916	79.50828	0.334	0.5138462	ns	74	-2.04298733	120.06536	0.0432	0.18782609	ns
75	-1.10399917	77.53485	0.273	0.4622951	ns	75	-2.17066576	118.31112	0.032	0.15238095	ns
76	-1.16284521	75.1984	0.249	0.4403509	ns	76	-2.23529222	116.86054	0.0273	0.14	ns
77	-1.16520962	74.02319	0.248	0.4403509	ns	77	-2.30004033	116.64617	0.0232	0.12888889	ns
78	-1.09966448	74.29559	0.275	0.4622951	ns	78	-2.38869052	117.42173	0.0185	0.115625	ns
79	-1.0096648	76.04061	0.316	0.4984375	ns	79	-2.52300582	117.73173	0.013	0.09285714	ns
80	-0.94895473	78.90067	0.346	0.5164179	ns	80	-2.68589526	118.05424	0.00828	0.069	ns
81	-0.90392706	83.46471	0.369	0.5271429	ns	81	-2.82268292	119.24662	0.00558	0.054	ns
82	-0.86426155	89.32129	0.39	0.5424658	ns	82	-2.88703208	120.67638	0.00461	0.054	ns
83	-0.79669342	95.45508	0.428	0.5631579	ns	83	-2.8922588	122.30342	0.00453	0.054	ns
84	-0.66605628	103.92952	0.507	0.6417722	ns	84	-2.85829432	123.34424	0.005	0.054	ns
85	-0.53963032	113.05222	0.591	0.7296296	ns	85	-2.83894127	124.45049	0.00529	0.054	ns
86	-0.43142365	119.85127	0.667	0.8060241	ns	86	-2.8955934	125.03904	0.00447	0.054	ns
87	-0.31058278	124.06008	0.757	0.8905882	ns	87	-2.99647929	125.13693	0.00329	0.054	ns
88	-0.1827218	125.74587	0.855	0.9715909	ns	88	-3.05126751	125.07227	0.00278	0.054	ns
89	-0.06876585	125.88695	0.945	0.9919192	ns	89	-3.04163996	124.33775	0.00287	0.054	ns
90	-0.05092978	123.72673	0.959	0.9919192	ns	90	-2.96201437	122.57869	0.00367	0.054	ns
91	-0.02271944	121.54442	0.982	0.9919192	ns	91	-2.80095267	119.72078	0.00594	0.054	ns
92	0.08241742	121.09497	0.934	0.9919192	ns	92	-2.63589734	116.26801	0.00954	0.07338462	ns
93	0.16150431	120.02921	0.872	0.9758242	ns	93	-2.49343926	113.07835	0.0141	0.094	ns

94	0.18467417	117.6522	0.854	0.9715909	ns	94	-2.36033582	110.8436	0.02	0.11764706	ns
95	0.14099882	114.44587	0.888	0.9758242	ns	95	-2.22650645	109.59416	0.028	0.14	ns
96	0.07615961	111.7373	0.939	0.9919192	ns	96	-2.11053036	108.95229	0.0371	0.16863636	ns
97	0.01043406	109.6406	0.992	0.992	ns	97	-1.99469091	108.87064	0.0486	0.2025	ns
98	-0.04672216	108.38921	0.963	0.9919192	ns	98	-1.88835492	109.79336	0.0616	0.2464	ns
99	-0.09801049	108.0546	0.922	0.9919192	ns	99	-1.81032806	111.34299	0.0729	0.27	ns
100	-0.14887948	108.54904	0.882	0.9758242	ns	100	-1.73008645	112.99574	0.0863	0.30821429	ns
9	statistic	df	p	p.adj	p.adj.signif	10	statistic	df	p	p.adj	p.adj.signif
1	0.53353851	113.0587	0.595	0.6611111	ns	1	-0.41537275	88.41937	0.679	0.76853933	ns
2	0.90761622	109.9003	0.366	0.4717949	ns	2	-0.4088805	87.91024	0.684	0.76853933	ns
3	1.67081948	111.6406	0.0976	0.2711111	ns	3	-0.55877561	88.83753	0.578	0.70487805	ns
4	1.9364689	115.398	0.0553	0.2126923	ns	4	-0.82886736	90.85178	0.409	0.64647887	ns
5	2.29602869	117.2581	0.0235	0.1566667	ns	5	-1.11554247	92.78695	0.267	0.58043478	ns
6	2.37270321	117.1698	0.0193	0.1471429	ns	6	-1.39106661	94.00886	0.167	0.46216216	ns
7	2.34833191	115.0137	0.0206	0.1471429	ns	7	-1.7587955	93.35039	0.0819	0.36608696	ns
8	2.404299	113.4415	0.0178	0.1471429	ns	8	-2.18612107	92.07225	0.0313	0.20866667	ns
9	2.50069685	110.3727	0.0139	0.1471429	ns	9	-2.61465338	91.63652	0.0104	0.10727273	ns
10	2.18608959	111.8359	0.0309	0.1609524	ns	10	-2.97707819	91.38217	0.00372	0.05585714	ns
11	2.14865334	113.8422	0.0338	0.1609524	ns	11	-3.28942579	91.3803	0.00143	0.0286	*
12	1.91840647	116.2716	0.0575	0.212963	ns	12	-3.55706831	91.75263	0.000596	0.01986667	*
13	1.3713394	117.7409	0.173	0.3089286	ns	13	-3.66198094	92.14515	0.000417	0.01986667	*
14	0.79997885	119.1743	0.425	0.5182927	ns	14	-3.57210592	92.99177	0.000563	0.01986667	*
15	0.17867935	119.317	0.858	0.8845361	ns	15	-3.32380772	95.3462	0.00126	0.0286	*
16	-0.15254349	122.3031	0.879	0.8969388	ns	16	-2.95492406	99.36595	0.00391	0.05585714	ns
17	-0.57047376	124.2539	0.569	0.6393258	ns	17	-2.56586811	103.95533	0.0117	0.10727273	ns

18	-1.0511213	126.3641	0.295	0.4180556	ns	18	-2.18722369	107.55175	0.0309	0.20866667	ns
19	-1.30167382	127.499	0.195	0.3362069	ns	19	-1.78686092	109.41999	0.0767	0.36608696	ns
20	-1.26270361	127.5237	0.209	0.35	ns	20	-1.35996286	110.65293	0.177	0.46578947	ns
21	-0.72284013	125.4069	0.471	0.5541176	ns	21	-0.96385544	111.86788	0.337	0.60178571	ns
22	-0.25880985	121.3722	0.796	0.8468085	ns	22	-0.62988896	113.15621	0.53	0.69615385	ns
23	0.41032802	119.1456	0.682	0.7413043	ns	23	-0.34404879	114.63594	0.731	0.79680851	ns
24	0.90954994	118.6491	0.365	0.4717949	ns	24	-0.10782568	115.77822	0.914	0.93265306	ns
25	1.45518505	118.3796	0.148	0.3055556	ns	25	0.08484398	115.96482	0.933	0.94242424	ns
26	1.55056829	117.1925	0.124	0.297619	ns	26	0.28472126	115.03203	0.776	0.81354167	ns
27	1.7202386	115.1275	0.0881	0.2591176	ns	27	0.45411001	114.91062	0.651	0.76588235	ns
28	1.44996283	116.3383	0.15	0.3055556	ns	28	0.61552813	116.65617	0.539	0.69615385	ns
29	1.19569277	118.2431	0.234	0.3714286	ns	29	0.92509785	118.89623	0.357	0.60508475	ns
30	1.40479299	118.4065	0.163	0.3055556	ns	30	1.37602952	119.1546	0.171	0.46216216	ns
31	1.21370381	115.7579	0.227	0.366129	ns	31	1.74158141	117.21928	0.0842	0.36608696	ns
32	1.32893177	109.3528	0.187	0.3280702	ns	32	1.93225937	113.83245	0.0558	0.31	ns
33	1.37940619	107.962	0.171	0.3089286	ns	33	2.09005241	110.19227	0.0389	0.243125	ns
34	1.80050479	104.0486	0.0747	0.240625	ns	34	2.45138564	107.79419	0.0158	0.13166667	ns
35	1.84083705	101.5248	0.0686	0.2286667	ns	35	2.69944593	104.36043	0.0081	0.10125	ns
36	2.17664436	106.0944	0.0317	0.1609524	ns	36	2.56458911	101.13208	0.0118	0.10727273	ns
37	2.1507366	107.3824	0.0337	0.1609524	ns	37	2.20755318	98.35258	0.0296	0.20866667	ns
38	2.23775719	107.4996	0.0273	0.1609524	ns	38	2.00641997	94.78953	0.0477	0.28058824	ns
39	2.52354124	107.9145	0.0131	0.1471429	ns	39	1.81943184	91.21977	0.0721	0.36608696	ns
40	2.55963802	107.1221	0.0119	0.1471429	ns	40	1.52348439	89.05277	0.131	0.42580645	ns
41	2.55692036	109.0054	0.0119	0.1471429	ns	41	1.32835784	87.57462	0.188	0.4804878	ns
42	2.47495746	109.1958	0.0149	0.1471429	ns	42	1.18445172	86.11202	0.239	0.55581395	ns

43	2.38807307	108.1235	0.0187	0.1471429	ns	43	1.04524286	85.93553	0.299	0.60178571	ns
44	2.07571768	110.0137	0.0403	0.1752174	ns	44	0.97028239	84.35575	0.335	0.60178571	ns
45	1.84347885	109.1614	0.068	0.2286667	ns	45	0.90788735	82.8444	0.367	0.60806452	ns
46	1.78559624	107.3933	0.077	0.240625	ns	46	0.77764722	83.26562	0.439	0.64647887	ns
47	1.68185676	104.8591	0.0956	0.2711111	ns	47	0.61078179	83.99218	0.543	0.69615385	ns
48	1.57107245	105.5553	0.119	0.297619	ns	48	0.41611524	85.11504	0.678	0.76853933	ns
49	1.74675496	109.0326	0.0835	0.2530303	ns	49	0.2785892	86.84575	0.781	0.81354167	ns
50	1.97897953	112.389	0.0503	0.2012	ns	50	0.33391099	87.73489	0.739	0.79680851	ns
51	2.16682731	112.8825	0.0323	0.1609524	ns	51	0.41858219	88.57555	0.677	0.76853933	ns
52	1.89483309	112.0511	0.0607	0.2167857	ns	52	0.4937446	89.78167	0.623	0.75060241	ns
53	1.54647681	112.4025	0.125	0.297619	ns	53	0.5609295	91.05728	0.576	0.70487805	ns
54	1.47665586	112.3842	0.143	0.3055556	ns	54	0.57828186	92.80898	0.564	0.70487805	ns
55	1.26147189	109.5492	0.21	0.35	ns	55	0.6320009	94.09669	0.529	0.69615385	ns
56	1.46332919	108.0093	0.146	0.3055556	ns	56	0.7441791	95.48559	0.459	0.64647887	ns
57	1.47450073	108.4415	0.143	0.3055556	ns	57	0.7854101	96.19012	0.434	0.64647887	ns
58	1.43840098	108.4984	0.153	0.3055556	ns	58	0.74782832	94.6194	0.456	0.64647887	ns
59	1.40439226	111.1937	0.163	0.3055556	ns	59	0.72570702	91.20343	0.47	0.64657534	ns
60	1.39607695	112.586	0.165	0.3055556	ns	60	0.76988601	88.3492	0.443	0.64647887	ns
61	1.24104381	110.96	0.217	0.3557377	ns	61	0.89454091	87.80721	0.373	0.60806452	ns
62	1.04566922	108.4175	0.298	0.4180556	ns	62	0.96447545	88.50084	0.337	0.60178571	ns
63	0.81927603	106.0637	0.414	0.5111111	ns	63	1.00016157	89.9795	0.32	0.60178571	ns
64	0.86979165	105.9497	0.386	0.48375	ns	64	1.02218095	91.16113	0.309	0.60178571	ns
65	1.05412844	110.894	0.294	0.4180556	ns	65	1.12305446	91.93984	0.264	0.58043478	ns
66	0.92230517	112.9646	0.358	0.4717949	ns	66	1.30046833	93.44779	0.197	0.4804878	ns
67	0.90360235	117.4066	0.368	0.4717949	ns	67	1.45363737	97.30758	0.149	0.45151515	ns

68	1.05642651	119.7929	0.293	0.4180556	ns	68	1.4845012	101.67012	0.141	0.440625	ns
69	1.07179816	120.2991	0.286	0.4180556	ns	69	1.51823342	105.57162	0.132	0.42580645	ns
70	1.40313945	118.4796	0.163	0.3055556	ns	70	1.59727868	108.38712	0.113	0.40357143	ns
71	1.55191405	118.5087	0.123	0.297619	ns	71	1.65724438	111.79254	0.1	0.38461538	ns
72	1.3967509	118.9942	0.165	0.3055556	ns	72	1.5947777	115.93742	0.113	0.40357143	ns
73	1.40118917	119.5316	0.164	0.3055556	ns	73	1.42175635	118.60353	0.158	0.46	ns
74	0.9572363	120.6152	0.34	0.4608108	ns	74	1.24075039	118.87247	0.217	0.51666667	ns
75	0.57281666	121.7673	0.568	0.6393258	ns	75	1.02168833	118.78179	0.309	0.60178571	ns
76	0.01443665	122.5948	0.989	0.989	ns	76	0.75585228	118.80517	0.451	0.64647887	ns
77	-0.61003174	121.8465	0.543	0.6241379	ns	77	0.56203998	118.39597	0.575	0.70487805	ns
78	-0.95536188	121.4653	0.341	0.4608108	ns	78	0.4597554	118.21166	0.647	0.76588235	ns
79	-1.03950352	120.9483	0.301	0.4180556	ns	79	0.39282266	118.9708	0.695	0.77222222	ns
80	-1.03869742	122.0337	0.301	0.4180556	ns	80	0.32023335	119.75506	0.749	0.79680851	ns
81	-0.78532379	124.3669	0.434	0.5228916	ns	81	0.19338578	120.09275	0.847	0.87319588	ns
82	-0.36954385	125.6719	0.712	0.7655914	ns	82	-0.02722893	119.8801	0.978	0.978	ns
83	-0.06416599	127.116	0.949	0.9585859	ns	83	-0.32509198	118.67491	0.746	0.79680851	ns
84	0.1982889	127.7735	0.843	0.878125	ns	84	-0.66962541	116.55481	0.504	0.68108108	ns
85	0.43068745	126.8645	0.667	0.732967	ns	85	-1.00686742	113.89181	0.316	0.60178571	ns
86	0.76502514	126.0037	0.446	0.5309524	ns	86	-1.30483535	111.45731	0.195	0.4804878	ns
87	1.1239533	126.0175	0.263	0.4046154	ns	87	-1.54288659	110.11383	0.126	0.42580645	ns
88	1.54417277	125.4903	0.125	0.297619	ns	88	-1.70272165	110.68444	0.0914	0.38083333	ns
89	2.12357216	125.545	0.0357	0.1622727	ns	89	-1.75217772	113.25195	0.0824	0.36608696	ns
90	2.72162736	126.2235	0.00741	0.1471429	ns	90	-1.65791173	116.8695	0.1	0.38461538	ns
91	3.10473758	125.8882	0.00235	0.1345	ns	91	-1.40979341	120.30385	0.161	0.46	ns
92	3.06229818	124.3669	0.00269	0.1345	ns	92	-1.12931684	122.77037	0.261	0.58043478	ns

93	2.90844168	123.0238	0.00431	0.1436667	ns	93	-0.93188647	123.91528	0.353	0.60508475	ns
94	2.46348041	122.0416	0.0152	0.1471429	ns	94	-0.79889909	123.56988	0.426	0.64647887	ns
95	1.99400683	123.4484	0.0484	0.2012	ns	95	-0.72138712	121.95728	0.472	0.64657534	ns
96	1.59109062	124.4723	0.114	0.297619	ns	96	-0.76452308	119.24727	0.446	0.64647887	ns
97	1.13162606	125.1787	0.26	0.4046154	ns	97	-0.88736571	116.023	0.377	0.60806452	ns
98	0.70565313	126.0809	0.482	0.5604651	ns	98	-0.94384577	113.16986	0.347	0.60508475	ns
99	0.24390955	121.1932	0.808	0.8505263	ns	99	-0.9656737	111.13626	0.336	0.60178571	ns
100	0.86784594	114.8802	0.387	0.48375	ns	100	-0.98343339	110.06142	0.328	0.60178571	ns

11	statistic	df	p	p.adj	p.adj.signif	12	statistic	df	p	p.adj	p.adj.signif
1	0.4549323	121.14076	0.65	0.9013333	ns	1	1.40624828	115.1662	0.162	0.3	ns
2	0.41940925	119.58446	0.676	0.9013333	ns	2	1.03448282	115.21465	0.303	0.43913043	ns
3	0.33380512	119.15994	0.739	0.9474359	ns	3	0.65745666	117.14098	0.512	0.58850575	ns
4	0.09678862	116.46386	0.923	0.9597938	ns	4	0.55276183	116.82189	0.581	0.63846154	ns
5	-0.19419905	115.76208	0.846	0.9597938	ns	5	0.58170437	116.34242	0.562	0.62444444	ns
6	-0.38570805	117.09797	0.7	0.9210526	ns	6	0.7560713	113.53477	0.451	0.56097561	ns
7	-0.20135503	116.24392	0.841	0.9597938	ns	7	0.77131229	109.85195	0.442	0.55949367	ns
8	0.09060759	116.42638	0.928	0.9597938	ns	8	0.62316361	111.03062	0.534	0.60681818	ns
9	0.44868231	116.76787	0.654	0.9013333	ns	9	0.66283044	115.36767	0.509	0.58850575	ns
10	0.65885042	114.77606	0.511	0.8396825	ns	10	0.84966295	116.87051	0.397	0.51558442	ns
11	0.77813209	116.00797	0.438	0.8396825	ns	11	1.13766829	116.59252	0.258	0.41612903	ns
12	1.06998363	117.30683	0.287	0.7121951	ns	12	1.24637047	115.73875	0.215	0.37413793	ns
13	1.24944607	118.76785	0.214	0.7076923	ns	13	1.11582849	115.46746	0.267	0.4171875	ns
14	1.33260476	117.60459	0.185	0.6851852	ns	14	0.92090799	114.60187	0.359	0.49178082	ns
15	1.10778822	118.63911	0.27	0.7076923	ns	15	0.92551269	113.01339	0.357	0.49178082	ns

16	1.01839967	120.64991	0.311	0.7372093	ns	16	1.07102917	112.97609	0.286	0.42686567	ns
17	1.1537229	123.31735	0.251	0.7076923	ns	17	1.05667417	110.89111	0.293	0.43088235	ns
18	1.4767541	124.50066	0.142	0.6173913	ns	18	0.95580792	108.76093	0.341	0.48028169	ns
19	1.89655568	124.79626	0.0602	0.3584211	ns	19	0.86515989	106.61766	0.389	0.51558442	ns
20	2.25387378	125.1379	0.0259	0.2354545	ns	20	0.80118345	106.34896	0.425	0.54487179	ns
21	2.33937849	124.82164	0.0209	0.227	ns	21	0.74263952	109.37037	0.459	0.56097561	ns
22	2.1192531	123.53892	0.0361	0.2846154	ns	22	0.61156236	112.97116	0.542	0.60898876	ns
23	1.74793858	122.33777	0.083	0.415	ns	23	0.23934881	115.73365	0.811	0.82755102	ns
24	1.36643643	121.79372	0.174	0.6851852	ns	24	-0.26493616	116.46566	0.792	0.81649485	ns
25	1.0591503	122.94372	0.292	0.7121951	ns	25	-0.74173986	117.43119	0.46	0.56097561	ns
26	0.98642241	122.85292	0.326	0.7409091	ns	26	-1.17601124	118.98721	0.242	0.40333333	ns
27	1.13135362	123.31678	0.26	0.7076923	ns	27	-1.55920157	120.69323	0.122	0.23018868	ns
28	1.42943753	123.10666	0.155	0.6458333	ns	28	-1.9406683	120.25185	0.0546	0.16878788	ns
29	1.8403025	122.90601	0.0681	0.3584211	ns	29	-2.13390254	118.45373	0.0349	0.12925926	ns
30	2.30859288	121.87307	0.0227	0.227	ns	30	-2.01304265	115.82788	0.0464	0.16	ns
31	2.68797684	118.58891	0.00822	0.137	ns	31	-1.81740377	113.55767	0.0718	0.19648649	ns
32	3.12439674	115.20494	0.00225	0.073	ns	32	-1.74771286	112.91953	0.0832	0.20634146	ns
33	3.50504905	112.89341	0.000656	0.0401	*	33	-1.85583584	113.00069	0.0661	0.19441176	ns
34	3.44717606	110.68704	0.000802	0.0401	*	34	-1.98595235	115.52778	0.0494	0.16466667	ns
35	3.04451316	109.23723	0.00292	0.073	ns	35	-2.05153422	117.52853	0.0424	0.15142857	ns
36	2.54571549	110.74388	0.0123	0.15375	ns	36	-2.17720832	114.88744	0.0315	0.12115385	ns
37	1.98130173	113.1926	0.05	0.3571429	ns	37	-2.40059817	108.75695	0.0181	0.07541667	ns
38	1.56357668	115.08774	0.121	0.5761905	ns	38	-2.51877898	99.84397	0.0134	0.067	ns
39	1.28274365	114.47854	0.202	0.7076923	ns	39	-2.44090959	92.17652	0.0166	0.07217391	ns
40	1.11662233	116.07445	0.266	0.7076923	ns	40	-2.36907275	91.5537	0.0199	0.0796	ns

41	1.20283676	120.77225	0.231	0.7076923	ns	41	-2.45791705	92.1497	0.0158	0.07181818	ns
42	1.48942798	122.51724	0.139	0.6173913	ns	42	-2.77681534	91.27222	0.00666	0.037	*
43	1.85275585	120.23698	0.0664	0.3584211	ns	43	-3.01499309	90.38074	0.00334	0.02385714	*
44	2.10975332	115.6857	0.037	0.2846154	ns	44	-3.10741505	92.54504	0.00251	0.02133333	*
45	1.85280656	108.57262	0.0666	0.3584211	ns	45	-3.11172764	94.04479	0.00246	0.02133333	*
46	1.34198182	102.69871	0.183	0.6851852	ns	46	-3.09890514	96.91673	0.00254	0.02133333	*
47	1.00535905	104.80966	0.317	0.7372093	ns	47	-3.0939035	99.97321	0.00256	0.02133333	*
48	0.64502296	110.46034	0.52	0.8396825	ns	48	-3.17472537	100.96805	0.00199	0.02133333	*
49	0.2503672	110.63067	0.803	0.9597938	ns	49	-3.29392173	100.8084	0.00136	0.02133333	*
50	-0.08661036	107.32118	0.931	0.9597938	ns	50	-3.28673147	98.85431	0.0014	0.02133333	*
51	-0.49764451	101.70226	0.62	0.9013333	ns	51	-3.02312031	96.39153	0.00321	0.02385714	*
52	-0.69609406	98.30816	0.488	0.8396825	ns	52	-2.81620567	94.10271	0.00592	0.03482353	*
53	-0.63240111	97.0945	0.529	0.8396825	ns	53	-2.74181431	92.96655	0.00733	0.03857895	*
54	-0.47287949	98.43306	0.637	0.9013333	ns	54	-2.93914409	93.01673	0.00415	0.0259375	*
55	-0.22211544	103.58171	0.825	0.9597938	ns	55	-3.33176264	92.7681	0.00124	0.02133333	*
56	-0.03655701	108.13257	0.971	0.979	ns	56	-3.70342622	94.93682	0.000357	0.01785	*
57	0.13813317	112.71296	0.89	0.9597938	ns	57	-3.7315846	98.23146	0.000319	0.01785	*
58	0.02662225	115.56419	0.979	0.979	ns	58	-3.52384619	98.38825	0.000647	0.02133333	*
59	-0.31754014	115.43543	0.751	0.947561	ns	59	-3.22259894	99.83753	0.00172	0.02133333	*
60	-0.64693391	115.85591	0.519	0.8396825	ns	60	-2.93284277	103.25893	0.00414	0.0259375	*
61	-0.69330834	118.47818	0.489	0.8396825	ns	61	-2.48937426	108.79065	0.0143	0.06809524	ns
62	-0.43193002	117.6546	0.667	0.9013333	ns	62	-1.9712292	112.90025	0.0511	0.16483871	ns
63	0.10849947	120.3277	0.914	0.9597938	ns	63	-1.58456945	113.81938	0.116	0.22745098	ns
64	0.71777001	124.28922	0.474	0.8396825	ns	64	-1.16028228	110.30086	0.248	0.40655738	ns
65	1.16090678	125.54413	0.248	0.7076923	ns	65	-1.08953759	109.09603	0.278	0.42686567	ns

66	1.09338402	123.41727	0.276	0.7076923	ns	66	-1.35929212	110.55998	0.177	0.32181818	ns
67	0.86766653	120.65642	0.387	0.8396825	ns	67	-1.55896858	112.0445	0.122	0.23018868	ns
68	0.71715767	116.97949	0.475	0.8396825	ns	68	-1.69528035	113.83517	0.0928	0.214	ns
69	0.63570024	113.4193	0.526	0.8396825	ns	69	-1.63489728	113.80909	0.105	0.214	ns
70	0.71621404	112.76896	0.475	0.8396825	ns	70	-1.65156495	110.19645	0.101	0.214	ns
71	0.68375288	118.01601	0.495	0.8396825	ns	71	-1.66782955	104.99036	0.0983	0.214	ns
72	0.52596582	121.91536	0.6	0.9013333	ns	72	-1.67545519	99.11899	0.097	0.214	ns
73	0.1366009	123.06203	0.892	0.9597938	ns	73	-1.83587135	97.11089	0.0694	0.19648649	ns
74	-0.18173564	123.14304	0.856	0.9597938	ns	74	-1.9364388	97.34083	0.0557	0.16878788	ns
75	0.11614195	118.59681	0.908	0.9597938	ns	75	-1.62759445	96.42463	0.107	0.214	ns
76	0.28605078	114.17722	0.775	0.947561	ns	76	-1.6597569	89.81174	0.1	0.214	ns
77	0.41967415	112.13242	0.676	0.9013333	ns	77	-1.80137879	84.56442	0.0752	0.19789474	ns
78	0.54252711	115.73783	0.588	0.9013333	ns	78	-1.78201939	85.1309	0.0783	0.20076923	ns
79	0.35720646	118.25052	0.722	0.9376623	ns	79	-1.81599083	91.06706	0.0727	0.19648649	ns
80	0.2837842	120.49942	0.777	0.947561	ns	80	-1.74291906	94.54645	0.0846	0.20634146	ns
81	-0.02929364	122.23006	0.977	0.979	ns	81	-1.67390196	94.99152	0.0974	0.214	ns
82	-0.7448725	122.97926	0.458	0.8396825	ns	82	-1.63596204	98.45856	0.105	0.214	ns
83	-1.191011	126.19599	0.236	0.7076923	ns	83	-1.24098064	105.32776	0.217	0.37413793	ns
84	-1.21232969	126.48199	0.228	0.7076923	ns	84	-1.08110651	110.68399	0.282	0.42686567	ns
85	-0.95621928	122.20193	0.341	0.7577778	ns	85	-0.97256353	112.6499	0.333	0.47571429	ns
86	-0.55105002	117.83741	0.583	0.9013333	ns	86	-0.6962967	113.69488	0.488	0.58095238	ns
87	-0.20385802	116.57609	0.839	0.9597938	ns	87	-0.85958067	107.1941	0.392	0.51558442	ns
88	0.1510293	117.10577	0.88	0.9597938	ns	88	-0.90591274	98.05122	0.367	0.49594595	ns
89	0.44043002	119.34199	0.66	0.9013333	ns	89	-0.68283935	92.96323	0.496	0.58352941	ns
90	0.7252353	122.79572	0.47	0.8396825	ns	90	-0.39118852	95.58701	0.697	0.74946237	ns

91	0.80372203	126.07473	0.423	0.8396825	ns	91	-0.01173376	98.25415	0.991	0.991	ns
92	0.76212026	126.74346	0.447	0.8396825	ns	92	0.36883068	103.678	0.713	0.75851064	ns
93	0.63406877	125.8854	0.527	0.8396825	ns	93	0.35854027	109.15382	0.721	0.75894737	ns
94	0.3003014	123.99007	0.764	0.947561	ns	94	0.27708559	114.85981	0.782	0.81458333	ns
95	-0.09861961	119.96488	0.922	0.9597938	ns	95	-0.02725523	117.16792	0.978	0.98787879	ns
96	-0.49231999	118.54846	0.623	0.9013333	ns	96	-0.42831648	113.16509	0.669	0.72717391	ns
97	-1.23180836	118.17872	0.22	0.7076923	ns	97	-0.7270779	109.47411	0.469	0.56506024	ns
98	-1.91211978	115.47309	0.0583	0.3584211	ns	98	-1.11610347	109.0337	0.267	0.4171875	ns
99	-2.54593152	109.55152	0.0123	0.15375	ns	99	-1.29757629	107.24204	0.197	0.35178571	ns
100	-2.85129966	105.40158	0.00524	0.1048	ns	100	-1.20433626	102.92866	0.231	0.39152542	ns
13	statistic	df	p	p.adj	p.adj.signif	14	statistic	df	p	p.adj	p.adj.signif
1	1.02278131	120.498	0.308	0.5216667	ns	1	1.01913189	117.90126	3.10E-01	0.361627907	ns
2	1.12161586	122.028	0.264	0.4732143	ns	2	1.02161831	120.0361	3.09E-01	0.361627907	ns
3	1.2965448	123.2498	0.197	0.402	ns	3	1.02754398	122.54912	3.06E-01	0.361627907	ns
4	1.46180013	123.6434	0.146	0.352381	ns	4	1.041946	124.62509	2.99E-01	0.361627907	ns
5	1.6312703	123.5934	0.105	0.2763158	ns	5	1.06369545	126.30384	2.89E-01	0.36125	ns
6	1.76932544	123.0817	0.0793	0.273871	ns	6	1.1294615	127.25055	2.61E-01	0.344155844	ns
7	1.84844587	122.703	0.0669	0.273871	ns	7	1.20016937	127.62366	2.32E-01	0.336231884	ns
8	1.88039775	122.9215	0.0624	0.273871	ns	8	1.19006769	127.6798	2.36E-01	0.337142857	ns
9	1.86263456	123.8017	0.0649	0.273871	ns	9	1.16966688	127.50244	2.44E-01	0.343661972	ns
10	1.80541439	124.6868	0.0734	0.273871	ns	10	1.15978697	126.67928	2.48E-01	0.344155844	ns
11	1.75383083	125.235	0.0819	0.273871	ns	11	1.14510532	124.59728	2.54E-01	0.344155844	ns
12	1.73687004	125.6589	0.0849	0.273871	ns	12	1.12431448	121.79295	2.63E-01	0.344155844	ns
13	1.74615448	126.0748	0.0832	0.273871	ns	13	1.10556344	119.34958	2.71E-01	0.347435897	ns
14	1.7616401	126.3839	0.0805	0.273871	ns	14	1.0283772	117.58474	3.06E-01	0.361627907	ns

15	1.78531942	126.438	0.0766	0.273871	ns	15	0.89347919	115.9777	3.73E-01	0.414444444	ns
16	1.80682727	126.1583	0.0732	0.273871	ns	16	0.70007991	114.75314	4.85E-01	0.521505376	ns
17	1.82893214	125.7963	0.0698	0.273871	ns	17	0.4836381	114.70302	6.30E-01	0.663157895	ns
18	1.82170952	125.5111	0.0709	0.273871	ns	18	0.25821048	115.66162	7.97E-01	0.813265306	ns
19	1.77026645	125.2194	0.0791	0.273871	ns	19	0.01877226	116.70663	9.85E-01	0.985	ns
20	1.69222853	124.7106	0.0931	0.2755556	ns	20	-0.19338707	117.78879	8.47E-01	0.855555556	ns
21	1.66428241	124.217	0.0986	0.2755556	ns	21	-0.34130349	118.79786	7.33E-01	0.755670103	ns
22	1.67570031	123.6794	0.0963	0.2755556	ns	22	-0.46569506	120.04353	6.42E-01	0.66875	ns
23	1.77872357	123.0644	0.0778	0.273871	ns	23	-0.59124094	121.36889	5.55E-01	0.590425532	ns
24	1.95355556	122.8671	0.053	0.273871	ns	24	-0.71097128	122.20048	4.78E-01	0.519565217	ns
25	2.1446796	122.8332	0.0339	0.273871	ns	25	-0.86008176	122.61194	3.91E-01	0.42967033	ns
26	2.35564839	122.999	0.0201	0.273871	ns	26	-0.96095669	122.85258	3.38E-01	0.388505747	ns
27	2.55756293	123.3614	0.0118	0.273871	ns	27	-1.01733837	122.46065	3.11E-01	0.361627907	ns
28	2.62269977	123.5435	0.00982	0.273871	ns	28	-1.11881828	122.01869	2.65E-01	0.344155844	ns
29	2.58196554	123.6662	0.011	0.273871	ns	29	-1.24347686	122.02418	2.16E-01	0.317647059	ns
30	2.43330278	124.1626	0.0164	0.273871	ns	30	-1.33726939	122.26573	1.84E-01	0.278787879	ns
31	2.26178727	124.7977	0.0254	0.273871	ns	31	-1.40874601	122.20073	1.61E-01	0.2515625	ns
32	2.06102915	125.8457	0.0414	0.273871	ns	32	-1.4428635	121.88119	1.52E-01	0.2515625	ns
33	1.85206174	126.7585	0.0663	0.273871	ns	33	-1.42032803	122.07652	1.58E-01	0.2515625	ns
34	1.60191357	127.2947	0.112	0.2871795	ns	34	-1.41949481	122.67238	1.58E-01	0.2515625	ns
35	1.29485144	127.722	0.198	0.402	ns	35	-1.46450883	123.15513	1.46E-01	0.247457627	ns
36	0.96683031	127.9424	0.335	0.5234375	ns	36	-1.57641734	123.30976	1.17E-01	0.205263158	ns
37	0.69724126	127.9835	0.487	0.6901408	ns	37	-1.74723038	123.25178	8.31E-02	0.156792453	ns
38	0.4719147	127.9838	0.638	0.8621622	ns	38	-1.96177716	122.96934	5.20E-02	0.104	ns
39	0.30212726	127.9562	0.763	0.9192771	ns	39	-2.1990653	122.06997	2.98E-02	0.062083333	ns

40	0.22062629	127.8298	0.826	0.9386364	ns	40	-2.43033708	120.89187	1.66E-02	0.037727273	*
41	0.23553292	127.5125	0.814	0.9386364	ns	41	-2.65002206	119.72325	9.14E-03	0.022292683	*
42	0.30208898	126.925	0.763	0.9192771	ns	42	-2.85478318	118.44769	5.09E-03	0.013756757	*
43	0.33887798	126.104	0.735	0.9192771	ns	43	-3.04836283	117.42013	2.84E-03	0.008352941	**
44	0.36722119	124.6991	0.714	0.9179487	ns	44	-3.21453639	115.80489	1.69E-03	0.00637037	**
45	0.32807369	123.0395	0.743	0.9192771	ns	45	-3.26630606	115.26667	1.44E-03	0.00626087	**
46	0.19891389	121.7257	0.843	0.947191	ns	46	-3.27305385	115.35314	1.40E-03	0.00626087	**
47	0.04255917	119.8072	0.966	0.9868687	ns	47	-3.28195357	115.1449	1.36E-03	0.00626087	**
48	-0.12908796	115.7633	0.898	0.9553191	ns	48	-3.24878399	115.53995	1.52E-03	0.006333333	**
49	-0.25399543	109.4119	0.8	0.9386364	ns	49	-3.1888842	118.08437	1.83E-03	0.006535714	**
50	-0.2253372	109.5743	0.822	0.9386364	ns	50	-3.14790351	120.43799	2.07E-03	0.006545455	**
51	-0.18224546	112.4026	0.856	0.9511111	ns	51	-3.13376974	121.83743	2.16E-03	0.006545455	**
52	-0.13617052	115.2827	0.892	0.9553191	ns	52	-3.14473801	122.40521	2.09E-03	0.006545455	**
53	-0.03965515	118.0663	0.968	0.9868687	ns	53	-3.13837238	121.76382	2.13E-03	0.006545455	**
54	0.10922918	120.2234	0.913	0.9610526	ns	54	-3.15302493	119.63107	2.04E-03	0.006545455	**
55	0.23343324	121.2983	0.816	0.9386364	ns	55	-3.21528047	116.98653	1.68E-03	0.00637037	**
56	0.32280756	121.7655	0.747	0.9192771	ns	56	-3.33915431	114.79997	1.13E-03	0.00565	**
57	0.42498432	121.7683	0.672	0.896	ns	57	-3.50577156	113.68233	6.53E-04	0.003627778	**
58	0.55500906	121.5493	0.58	0.7945205	ns	58	-3.7133414	112.35805	3.20E-04	0.002	**
59	0.6929581	121.4223	0.49	0.6901408	ns	59	-3.91599505	109.7397	1.57E-04	0.001207692	**
60	0.83843272	121.0009	0.403	0.6014925	ns	60	-4.08889517	105.54467	8.48E-05	0.000828182	***
61	0.99515228	119.8745	0.322	0.5234375	ns	61	-4.23154386	100.86639	5.12E-05	0.00064	***
62	1.13205999	118.4039	0.26	0.4732143	ns	62	-4.34902613	97.13811	3.37E-05	0.000635	***
63	1.20151141	117.4256	0.232	0.4461538	ns	63	-4.41314978	94.53797	2.70E-05	0.000635	***
64	1.12057559	117.5371	0.265	0.4732143	ns	64	-4.40026432	92.41083	2.89E-05	0.000635	***

65	0.9788267	117.416	0.33	0.5234375	ns	65	-4.36241628	90.58957	3.40E-05	0.000635	***
66	0.85881519	116.2303	0.392	0.5939394	ns	66	-4.364902	89.6961	3.40E-05	0.000635	***
67	0.7427937	114.7382	0.459	0.675	ns	67	-4.33612596	88.89268	3.81E-05	0.000635	***
68	0.57923665	114.0311	0.564	0.7833333	ns	68	-4.29030666	87.91714	4.57E-05	0.00064	***
69	0.36407457	113.7069	0.716	0.9179487	ns	69	-4.21670873	86.88066	6.06E-05	0.000673333	***
70	0.16056132	113.8175	0.873	0.9553191	ns	70	-4.10550152	86.52067	9.11E-05	0.000828182	***
71	0.05598291	113.8144	0.955	0.9868687	ns	71	-3.99001478	86.50428	1.38E-04	0.00115	**
72	0.02878523	114.2568	0.977	0.9868687	ns	72	-3.8854931	86.67753	1.99E-04	0.001421429	**
73	-0.01553218	114.9292	0.988	0.988	ns	73	-3.77039132	86.92809	2.97E-04	0.00198	**
74	-0.14764674	115.5206	0.883	0.9553191	ns	74	-3.62337074	88.25812	4.85E-04	0.002852941	**
75	-0.41098181	116.1707	0.682	0.8973684	ns	75	-3.43079414	90.78152	9.08E-04	0.004778947	**
76	-0.69354994	116.7582	0.489	0.6901408	ns	76	-3.2273275	94.06424	1.72E-03	0.00637037	**
77	-0.87480181	118.6323	0.383	0.5892308	ns	77	-3.0402408	98.31887	3.03E-03	0.008657143	**
78	-0.97338849	120.8082	0.332	0.5234375	ns	78	-2.91414904	103.14025	4.37E-03	0.012138889	*
79	-1.01218041	122.3302	0.313	0.5216667	ns	79	-2.82244891	107.32664	5.68E-03	0.014947368	*
80	-1.03917483	123.3136	0.301	0.5189655	ns	80	-2.77556518	109.01502	6.49E-03	0.016641026	*
81	-1.10122333	123.1743	0.273	0.4789474	ns	81	-2.72623643	109.2457	7.46E-03	0.01865	*
82	-1.17172397	122.7058	0.244	0.4603774	ns	82	-2.62622262	109.62098	9.87E-03	0.0235	*
83	-1.20926111	122.8205	0.229	0.4461538	ns	83	-2.4979667	110.57052	1.40E-02	0.03255814	*
84	-1.29579796	122.4382	0.197	0.402	ns	84	-2.40751373	112.76551	1.77E-02	0.039333333	*
85	-1.45483091	121.2032	0.148	0.352381	ns	85	-2.33214134	115.30872	2.14E-02	0.046521739	*
86	-1.67490895	119.437	0.0966	0.2755556	ns	86	-2.22257958	117.65002	2.82E-02	0.06	ns
87	-1.85786101	118.0774	0.0657	0.273871	ns	87	-2.04945028	119.23749	4.26E-02	0.086938776	ns
88	-2.00108403	116.685	0.0477	0.273871	ns	88	-1.87761628	120.11339	6.29E-02	0.123333333	ns
89	-2.01522861	114.4513	0.0462	0.273871	ns	89	-1.72720211	120.93108	8.67E-02	0.160555556	ns

90	-1.9052183	111.5608	0.0593	0.273871	ns	90	-1.59704967	122.37996	1.13E-01	0.201785714	ns
91	-1.66288891	108.7585	0.0992	0.2755556	ns	91	-1.48399612	123.38746	1.40E-01	0.24137931	ns
92	-1.40440715	107.9884	0.163	0.3790698	ns	92	-1.37134764	123.7888	1.73E-01	0.266153846	ns
93	-1.28615168	109.601	0.201	0.402	ns	93	-1.24595886	123.95179	2.15E-01	0.317647059	ns
94	-1.30611976	111.2849	0.194	0.402	ns	94	-1.07456776	123.701	2.85E-01	0.360759494	ns
95	-1.34662316	111.4225	0.181	0.402	ns	95	-0.92771986	123.00021	3.55E-01	0.4	ns
96	-1.34389617	110.147	0.182	0.402	ns	96	-0.92590176	123.10218	3.56E-01	0.4	ns
97	-1.47693032	108.6044	0.143	0.352381	ns	97	-1.12256754	124.64042	2.64E-01	0.344155844	ns
98	-1.63612011	108.6959	0.105	0.2763158	ns	98	-1.41595597	126.18983	1.59E-01	0.2515625	ns
99	-1.87748668	107.268	0.0632	0.273871	ns	99	-1.65585118	127.51429	1.00E-01	0.181818182	ns
100	-1.97043281	107.4639	0.0514	0.273871	ns	100	-1.80799331	127.98345	7.30E-02	0.140384615	ns
15	statistic	df	p	p.adj	p.adj.signif	16	statistic	df	p	p.adj	p.adj.signif
1	0.664837175	118.238	0.507	0.999	ns	1	-1.46688776	109.08903	0.145	0.40277778	ns
2	0.555751954	117.4342	0.579	0.999	ns	2	-1.5650618	108.1443	0.12	0.34285714	ns
3	0.485690697	116.4891	0.628	0.999	ns	3	-1.67227154	106.9949	0.0974	0.29515152	ns
4	0.438100034	115.3489	0.662	0.999	ns	4	-1.7745359	105.81352	0.0789	0.2465625	ns
5	0.378896788	114.0781	0.705	0.999	ns	5	-1.87533626	104.54202	0.0635	0.21166667	ns
6	0.310991678	112.8032	0.756	0.999	ns	6	-1.97151852	103.33453	0.0513	0.17689655	ns
7	0.241159536	111.5841	0.81	0.999	ns	7	-2.05812545	102.29801	0.0421	0.15592593	ns
8	0.181613875	110.4581	0.856	0.999	ns	8	-2.13499387	101.45197	0.0352	0.13538462	ns
9	0.130688473	109.4862	0.896	0.999	ns	9	-2.21406119	100.66642	0.0291	0.12125	ns
10	0.082946536	108.7479	0.934	0.999	ns	10	-2.30237021	100.00219	0.0234	0.10173913	ns
11	0.047050073	108.3264	0.963	0.999	ns	11	-2.39335576	99.57862	0.0186	0.08857143	ns
12	0.027043435	108.3198	0.978	0.999	ns	12	-2.48331863	99.45889	0.0147	0.07736842	ns
13	0.015051719	108.6137	0.988	0.999	ns	13	-2.57271774	99.599	0.0116	0.06444444	ns

14	-0.00101698	109.0355	0.999	0.999	ns	14	-2.66552686	99.97235	0.00896	0.056	ns
15	-0.018942	109.5641	0.985	0.999	ns	15	-2.75827287	100.69264	0.0069	0.04928571	*
16	-0.02769996	110.2311	0.978	0.999	ns	16	-2.84746199	101.73182	0.00533	0.04441667	*
17	-0.02186206	111.0639	0.983	0.999	ns	17	-2.9242578	102.94278	0.00425	0.0425	*
18	-0.01870799	111.9504	0.985	0.999	ns	18	-2.9871292	104.09908	0.00351	0.0425	*
19	-0.02099295	112.8976	0.983	0.999	ns	19	-3.03226052	105.0874	0.00306	0.0425	*
20	-0.01863209	113.9686	0.985	0.999	ns	20	-3.05405995	105.87416	0.00286	0.0425	*
21	-0.00907014	115.2208	0.993	0.999	ns	21	-3.05428609	106.42224	0.00285	0.0425	*
22	0.007983681	116.5233	0.994	0.999	ns	22	-3.04614386	106.76471	0.00292	0.0425	*
23	0.033530071	117.6521	0.973	0.999	ns	23	-3.03754453	107.00124	0.003	0.0425	*
24	0.069278972	118.5391	0.945	0.999	ns	24	-3.02209493	107.20382	0.00314	0.0425	*
25	0.112097121	119.27	0.911	0.999	ns	25	-2.99096345	107.34492	0.00345	0.0425	*
26	0.159195597	119.8553	0.874	0.999	ns	26	-2.93983536	107.32078	0.00402	0.0425	*
27	0.20300278	120.3193	0.839	0.999	ns	27	-2.86806265	107.03762	0.00498	0.04441667	*
28	0.243519744	120.6316	0.808	0.999	ns	28	-2.78170888	106.50745	0.0064	0.04923077	*
29	0.28002149	120.8638	0.78	0.999	ns	29	-2.68243074	105.73696	0.00848	0.056	ns
30	0.308069852	121.0412	0.759	0.999	ns	30	-2.57620476	104.76508	0.0114	0.06444444	ns
31	0.321939501	121.1624	0.748	0.999	ns	31	-2.4619913	103.65678	0.0155	0.0775	ns
32	0.327887295	121.2241	0.744	0.999	ns	32	-2.33230697	102.51761	0.0216	0.09818182	ns
33	0.331136116	121.2495	0.741	0.999	ns	33	-2.17749831	101.42055	0.0318	0.1272	ns
34	0.338109444	121.3224	0.736	0.999	ns	34	-1.99745738	100.38124	0.0485	0.17321429	ns
35	0.346375107	121.4356	0.73	0.999	ns	35	-1.79577158	99.39188	0.0756	0.24387097	ns
36	0.353279512	121.5072	0.724	0.999	ns	36	-1.5807218	98.5043	0.117	0.34285714	ns
37	0.354312203	121.5549	0.724	0.999	ns	37	-1.35721319	97.8247	0.178	0.48108108	ns
38	0.347239351	121.5611	0.729	0.999	ns	38	-1.12991294	97.4035	0.261	0.53877551	ns

39	0.32937652	121.5256	0.742	0.999	ns	39	-0.90338634	97.24224	0.369	0.58461538	ns
40	0.297493859	121.4781	0.767	0.999	ns	40	-0.68193749	97.34982	0.497	0.67162162	ns
41	0.258573654	121.4735	0.796	0.999	ns	41	-0.46631384	97.75561	0.642	0.8025	ns
42	0.228469534	121.6078	0.82	0.999	ns	42	-0.26284107	98.44899	0.793	0.93294118	ns
43	0.216166947	121.976	0.829	0.999	ns	43	-0.07620875	99.40197	0.939	0.99693878	ns
44	0.213295772	122.5172	0.831	0.999	ns	44	0.090997237	100.55365	0.928	0.99693878	ns
45	0.210156062	123.1045	0.834	0.999	ns	45	0.240576743	101.94112	0.81	0.94186047	ns
46	0.203220751	123.6671	0.839	0.999	ns	46	0.372098117	103.53984	0.711	0.86707317	ns
47	0.192818504	124.1722	0.847	0.999	ns	47	0.481910997	105.34225	0.631	0.79873418	ns
48	0.1792851	124.5983	0.858	0.999	ns	48	0.571846745	107.35995	0.569	0.73896104	ns
49	0.15690058	124.9654	0.876	0.999	ns	49	0.644033658	109.60504	0.521	0.69466667	ns
50	0.127050929	125.2731	0.899	0.999	ns	50	0.704035442	111.99186	0.483	0.66849315	ns
51	0.097710978	125.5318	0.922	0.999	ns	51	0.754832108	114.41705	0.452	0.63661972	ns
52	0.06871654	125.7115	0.945	0.999	ns	52	0.797571013	116.77959	0.427	0.61884058	ns
53	0.036769081	125.7895	0.971	0.999	ns	53	0.833135684	118.96079	0.406	0.59705882	ns
54	-0.00089574	125.8023	0.999	0.999	ns	54	0.861429853	120.87638	0.391	0.59242424	ns
55	-0.05173847	125.7916	0.959	0.999	ns	55	0.881195914	122.47514	0.38	0.58461538	ns
56	-0.11880243	125.7094	0.906	0.999	ns	56	0.89529483	123.76586	0.372	0.58461538	ns
57	-0.20133863	125.4922	0.841	0.999	ns	57	0.910748207	124.8045	0.364	0.58461538	ns
58	-0.295258	125.1257	0.768	0.999	ns	58	0.930201357	125.60116	0.354	0.58461538	ns
59	-0.39510916	124.5908	0.693	0.999	ns	59	0.952848473	126.16422	0.342	0.58461538	ns
60	-0.4996678	123.9031	0.618	0.999	ns	60	0.981256055	126.5423	0.328	0.58461538	ns
61	-0.60735386	123.1046	0.545	0.999	ns	61	1.017004112	126.78073	0.311	0.57592593	ns
62	-0.71804082	122.2362	0.474	0.999	ns	62	1.057584339	126.90981	0.292	0.56153846	ns
63	-0.82774225	121.3397	0.409	0.999	ns	63	1.097443443	126.93802	0.275	0.55	ns

64	-0.9390891	120.4654	0.35	0.999	ns	64	1.134819712	126.85434	0.259	0.53877551	ns
65	-1.05485751	119.6752	0.294	0.999	ns	65	1.171488882	126.66369	0.244	0.5326087	ns
66	-1.17372714	118.9741	0.243	0.999	ns	66	1.205582497	126.38727	0.23	0.52272727	ns
67	-1.29287771	118.3352	0.199	0.999	ns	67	1.233361206	126.04496	0.22	0.52272727	ns
68	-1.41104268	117.7266	0.161	0.9388889	ns	68	1.251024854	125.61084	0.213	0.52272727	ns
69	-1.5230858	117.1007	0.13	0.85625	ns	69	1.254838822	125.11017	0.212	0.52272727	ns
70	-1.62553138	116.4369	0.107	0.7785714	ns	70	1.248143656	124.59653	0.214	0.52272727	ns
71	-1.71962026	115.8243	0.0882	0.7408333	ns	71	1.233205506	124.13622	0.22	0.52272727	ns
72	-1.80514509	115.2637	0.0737	0.739	ns	72	1.207692452	123.70483	0.229	0.52272727	ns
73	-1.87801924	114.7257	0.0629	0.739	ns	73	1.169007254	123.23858	0.245	0.5326087	ns
74	-1.93451666	114.1724	0.0555	0.739	ns	74	1.123033688	122.69896	0.264	0.53877551	ns
75	-1.97385786	113.6157	0.0508	0.739	ns	75	1.078021447	122.05294	0.283	0.55490196	ns
76	-1.99535899	113.1132	0.0484	0.739	ns	76	1.038449909	121.28219	0.301	0.56792453	ns
77	-1.99450565	112.7282	0.0485	0.739	ns	77	1.000352421	120.39235	0.319	0.58	ns
78	-1.9698551	112.4931	0.0513	0.739	ns	78	0.96609589	119.41663	0.336	0.58461538	ns
79	-1.93027816	112.4896	0.0561	0.739	ns	79	0.931407733	118.36961	0.354	0.58461538	ns
80	-1.87540506	112.7231	0.0633	0.739	ns	80	0.891230764	117.2916	0.375	0.58461538	ns
81	-1.80426019	113.1177	0.0739	0.739	ns	81	0.841318968	116.2415	0.402	0.59705882	ns
82	-1.71581257	113.6262	0.0889	0.7408333	ns	82	0.776502338	115.26767	0.439	0.62714286	ns
83	-1.61312001	114.2808	0.109	0.7785714	ns	83	0.695822923	114.33063	0.488	0.66849315	ns
84	-1.49907278	115.0352	0.137	0.85625	ns	84	0.604248717	113.52086	0.547	0.71973684	ns
85	-1.38472932	115.8428	0.169	0.9388889	ns	85	0.50952303	112.91023	0.611	0.78333333	ns
86	-1.27318019	116.7622	0.205	0.999	ns	86	0.418484147	112.64782	0.676	0.8345679	ns
87	-1.15518005	117.8491	0.25	0.999	ns	87	0.338458918	112.84552	0.736	0.88674699	ns
88	-1.02221714	119.1034	0.309	0.999	ns	88	0.275939228	113.53266	0.783	0.93214286	ns

89	-0.87636688	120.3994	0.383	0.999	ns	89	0.225344595	114.5444	0.822	0.94482759	ns
90	-0.72794506	121.6216	0.468	0.999	ns	90	0.173311574	115.64762	0.863	0.98068182	ns
91	-0.58219572	122.7406	0.562	0.999	ns	91	0.111647632	116.62617	0.911	0.99693878	ns
92	-0.43843367	123.752	0.662	0.999	ns	92	0.04505816	117.45795	0.964	0.99693878	ns
93	-0.29504767	124.6203	0.768	0.999	ns	93	-0.0087493	118.29509	0.993	1	ns
94	-0.15927462	125.3421	0.874	0.999	ns	94	-0.03872912	119.18299	0.969	0.99693878	ns
95	-0.04579195	125.9065	0.964	0.999	ns	95	-0.04851659	119.98664	0.961	0.99693878	ns
96	0.040986735	126.2967	0.967	0.999	ns	96	-0.0462826	120.51501	0.963	0.99693878	ns
97	0.102363948	126.571	0.919	0.999	ns	97	-0.02945191	120.76402	0.977	0.99693878	ns
98	0.135816328	126.7391	0.892	0.999	ns	98	0.000452221	120.72465	1	1	ns
99	0.136625781	126.8253	0.892	0.999	ns	99	0.043627807	120.38171	0.965	0.99693878	ns
100	0.114110643	126.8396	0.909	0.999	ns	100	0.097642166	119.63532	0.922	0.99693878	ns
17	statistic	df	p	p.adj	p.adj.signif	18	statistic	df	p	p.adj	p.adj.signif
1	0.17028648	96.80945	0.865	0.87373737	ns	1	-2.76136736	127.7464	0.0066	0.090625	ns
2	-0.18481507	93.32556	0.854	0.87373737	ns	2	-2.59394176	127.78622	0.0106	0.090625	ns
3	-0.25638618	97.20647	0.798	0.83125	ns	3	-2.43011991	127.78157	0.0165	0.090625	ns
4	-0.29050453	101.41428	0.772	0.81263158	ns	4	-2.28149116	127.71496	0.0242	0.090625	ns
5	-0.36875828	106.05534	0.713	0.77234043	ns	5	-2.14061123	127.54484	0.0342	0.09351351	ns
6	-0.46558226	109.76951	0.642	0.71333333	ns	6	-2.02336172	127.22194	0.0451	0.09931034	ns
7	-0.59877908	111.91433	0.551	0.61910112	ns	7	-1.95137265	126.57555	0.0532	0.09931034	ns
8	-0.75039347	112.93486	0.455	0.53529412	ns	8	-1.92237409	125.40427	0.0568	0.09931034	ns
9	-0.88010258	113.63962	0.381	0.45903614	ns	9	-1.92834451	123.82328	0.0561	0.09931034	ns
10	-0.9965401	114.27644	0.321	0.3962963	ns	10	-1.95571422	122.19736	0.0528	0.09931034	ns
11	-1.13513104	114.64648	0.259	0.38382353	ns	11	-1.99782734	120.73173	0.048	0.09931034	ns
12	-1.34388223	114.69392	0.182	0.31896552	ns	12	-2.05145856	119.54414	0.0424	0.09931034	ns

13	-1.62964431	114.49508	0.106	0.22083333	ns	13	-2.10562365	118.79338	0.0373	0.09564103	ns
14	-1.94057844	114.00512	0.0548	0.14421053	ns	14	-2.14966884	118.50627	0.0336	0.09351351	ns
15	-2.25580198	113.50856	0.026	0.14027027	ns	15	-2.18537681	118.50718	0.0308	0.09333333	ns
16	-2.53422314	113.21512	0.0126	0.07875	ns	16	-2.21005864	118.70718	0.029	0.090625	ns
17	-2.74124567	113.48125	0.00711	0.06253846	ns	17	-2.22438711	119.01641	0.028	0.090625	ns
18	-2.87507705	114.32331	0.00482	0.06253846	ns	18	-2.23206565	119.37116	0.0275	0.090625	ns
19	-2.94241074	115.6736	0.00394	0.06253846	ns	19	-2.23792012	119.82251	0.0271	0.090625	ns
20	-2.9564956	117.10839	0.00376	0.06253846	ns	20	-2.24522443	120.44683	0.0266	0.090625	ns
21	-2.9362142	118.33977	0.00399	0.06253846	ns	21	-2.25365072	121.25386	0.026	0.090625	ns
22	-2.90424565	119.32785	0.00439	0.06253846	ns	22	-2.2696626	122.20454	0.025	0.090625	ns
23	-2.85891119	120.32419	0.00501	0.06253846	ns	23	-2.29778941	123.16519	0.0233	0.090625	ns
24	-2.80643662	121.55974	0.00584	0.06253846	ns	24	-2.33710317	123.99707	0.021	0.090625	ns
25	-2.75137343	122.95341	0.00683	0.06253846	ns	25	-2.38602017	124.64279	0.0185	0.090625	ns
26	-2.7084281	124.38938	0.00771	0.06253846	ns	26	-2.43655437	125.06376	0.0162	0.090625	ns
27	-2.69367377	125.64815	0.00803	0.06253846	ns	27	-2.47440755	125.29739	0.0147	0.090625	ns
28	-2.69757544	126.57749	0.00794	0.06253846	ns	28	-2.49892717	125.40194	0.0137	0.090625	ns
29	-2.68889737	127.19563	0.00813	0.06253846	ns	29	-2.50733756	125.41017	0.0134	0.090625	ns
30	-2.6373157	127.55205	0.0094	0.06714286	ns	30	-2.5044136	125.32414	0.0135	0.090625	ns
31	-2.53460743	127.74448	0.0125	0.07875	ns	31	-2.4965357	125.06064	0.0138	0.090625	ns
32	-2.38996157	127.81236	0.0183	0.10764706	ns	32	-2.49481688	124.5458	0.0139	0.090625	ns
33	-2.21355345	127.78771	0.0286	0.14027027	ns	33	-2.50513176	123.69248	0.0135	0.090625	ns
34	-1.99376865	127.66288	0.0483	0.14027027	ns	34	-2.51456053	122.81314	0.0132	0.090625	ns
35	-1.72568087	127.39594	0.0868	0.19577778	ns	35	-2.52259754	121.84147	0.0129	0.090625	ns
36	-1.41337592	126.91669	0.16	0.2962963	ns	36	-2.5281111	120.60258	0.0128	0.090625	ns
37	-1.0716207	126.1506	0.286	0.384	ns	37	-2.52934024	119.07392	0.0127	0.090625	ns

38	-0.71399119	124.99349	0.477	0.55465116	ns	38	-2.51696251	117.30011	0.0132	0.090625	ns
39	-0.35102386	123.42698	0.726	0.77234043	ns	39	-2.48286671	115.41964	0.0145	0.090625	ns
40	0.01000733	121.67815	0.992	0.992	ns	40	-2.42951439	113.77137	0.0167	0.090625	ns
41	0.35953678	120.12633	0.72	0.77234043	ns	41	-2.35969205	112.73631	0.02	0.090625	ns
42	0.69690115	118.98082	0.487	0.55977011	ns	42	-2.28767569	112.12617	0.024	0.090625	ns
43	1.02570387	118.26469	0.307	0.38987342	ns	43	-2.22329466	111.77479	0.0282	0.090625	ns
44	1.33199889	117.87743	0.185	0.31896552	ns	44	-2.17358032	111.55876	0.0319	0.09351351	ns
45	1.59579526	117.63658	0.113	0.228	ns	45	-2.1390346	111.38234	0.0346	0.09351351	ns
46	1.81001491	117.27084	0.0729	0.16953488	ns	46	-2.11511975	111.051	0.0367	0.09564103	ns
47	1.96393265	116.7771	0.0519	0.14027027	ns	47	-2.08651825	110.5103	0.0392	0.098	ns
48	2.05045241	116.036	0.0426	0.14027027	ns	48	-2.05388446	109.58986	0.0424	0.09931034	ns
49	2.08013358	114.93987	0.0397	0.14027027	ns	49	-2.01027175	108.43052	0.0469	0.09931034	ns
50	2.06111739	113.84848	0.0416	0.14027027	ns	50	-1.97152088	107.23898	0.0512	0.09931034	ns
51	2.02267298	113.06011	0.0455	0.14027027	ns	51	-1.94734697	105.95974	0.0541	0.09931034	ns
52	1.97864586	112.50185	0.0503	0.14027027	ns	52	-1.9405372	104.63101	0.055	0.09931034	ns
53	1.91917885	111.87657	0.0575	0.1474359	ns	53	-1.92050327	103.20976	0.0576	0.09931034	ns
54	1.83196056	111.10819	0.0696	0.16571429	ns	54	-1.88915105	101.48925	0.0617	0.10114754	ns
55	1.72101146	109.91448	0.0881	0.19577778	ns	55	-1.86196763	99.67037	0.0656	0.10412698	ns
56	1.5926606	108.38153	0.114	0.228	ns	56	-1.8653306	98.18716	0.0651	0.10412698	ns
57	1.46031649	106.53235	0.147	0.27735849	ns	57	-1.90007484	97.19354	0.0604	0.10114754	ns
58	1.34146366	104.79334	0.183	0.31896552	ns	58	-1.93910289	96.78661	0.0554	0.09931034	ns
59	1.23551465	103.81471	0.219	0.35901639	ns	59	-1.96690174	96.73888	0.0521	0.09931034	ns
60	1.14713145	103.86273	0.254	0.38382353	ns	60	-1.98060399	96.95917	0.0505	0.09931034	ns
61	1.10201319	104.77607	0.273	0.384	ns	61	-1.97983215	97.42274	0.0505	0.09931034	ns
62	1.10816831	106.37892	0.27	0.384	ns	62	-1.95087125	98.12408	0.0539	0.09931034	ns

63	1.11903343	107.69612	0.266	0.384	ns	63	-1.88971077	98.92109	0.0617	0.10114754	ns
64	1.12940833	108.13488	0.261	0.38382353	ns	64	-1.81696512	99.5276	0.0722	0.1128125	ns
65	1.15536318	108.2829	0.25	0.38382353	ns	65	-1.74936438	99.57056	0.0833	0.12815385	ns
66	1.18316244	108.46808	0.239	0.37936508	ns	66	-1.68383845	99.02499	0.0954	0.14454545	ns
67	1.22559362	108.41842	0.223	0.35967742	ns	67	-1.59332644	98.07638	0.114	0.17014925	ns
68	1.29777423	108.16338	0.197	0.33389831	ns	68	-1.48267771	97.16841	0.141	0.20735294	ns
69	1.40176123	108.13603	0.164	0.29818182	ns	69	-1.36190056	96.481	0.176	0.25507246	ns
70	1.5271183	108.19273	0.13	0.25490196	ns	70	-1.23289181	96.07178	0.221	0.31571429	ns
71	1.68387183	108.61369	0.0951	0.20673913	ns	71	-1.10703861	95.85324	0.271	0.38169014	ns
72	1.84693712	109.91373	0.0674	0.16439024	ns	72	-0.98055031	95.7074	0.329	0.45068493	ns
73	1.99273698	112.02962	0.0487	0.14027027	ns	73	-0.84539363	95.50217	0.4	0.51948052	ns
74	2.11835654	114.42949	0.0363	0.14027027	ns	74	-0.71797638	95.11556	0.475	0.60126582	ns
75	2.19981329	116.38027	0.0298	0.14027027	ns	75	-0.60043345	94.35695	0.55	0.67901235	ns
76	2.2112961	117.4771	0.029	0.14027027	ns	76	-0.48811764	93.57689	0.627	0.76385542	ns
77	2.17962413	118.14963	0.0313	0.14027027	ns	77	-0.38334219	93.2581	0.702	0.83571429	ns
78	2.12574236	118.91894	0.0356	0.14027027	ns	78	-0.28894085	93.44222	0.773	0.89883721	ns
79	2.0692466	119.65642	0.0407	0.14027027	ns	79	-0.20109778	94.05988	0.841	0.94270833	ns
80	2.02289586	120.14463	0.0453	0.14027027	ns	80	-0.12051815	94.85218	0.904	0.94270833	ns
81	2.00927201	120.46934	0.0467	0.14027027	ns	81	-0.0449676	95.62984	0.964	0.97373737	ns
82	2.01272561	120.62852	0.0464	0.14027027	ns	82	0.02436077	96.30284	0.981	0.981	ns
83	1.96577959	120.3884	0.0516	0.14027027	ns	83	0.07419612	96.51311	0.941	0.96020408	ns
84	1.84571073	119.58646	0.0674	0.16439024	ns	84	0.10610703	96.19983	0.916	0.9443299	ns
85	1.67206908	118.43271	0.0972	0.20680851	ns	85	0.11981114	95.56623	0.905	0.94270833	ns
86	1.47412548	117.17249	0.143	0.275	ns	86	0.12403321	94.83355	0.902	0.94270833	ns
87	1.28572985	116.01241	0.201	0.335	ns	87	0.13246042	94.08509	0.895	0.94270833	ns

88	1.13534315	115.27414	0.259	0.38382353	ns	88	0.14979756	93.32217	0.881	0.94270833	ns
89	1.03736645	115.18111	0.302	0.38987342	ns	89	0.16906114	92.42431	0.866	0.94270833	ns
90	1.00528522	115.83296	0.317	0.39625	ns	90	0.18935815	91.29396	0.85	0.94270833	ns
91	1.03223618	116.94311	0.304	0.38987342	ns	91	0.21908687	89.96247	0.827	0.93977273	ns
92	1.07168412	117.65113	0.286	0.384	ns	92	0.2662222	88.51849	0.791	0.9091954	ns
93	1.08652827	117.93569	0.279	0.384	ns	93	0.35200152	87.58995	0.726	0.85411765	ns
94	1.06704721	117.93082	0.288	0.384	ns	94	0.47794291	87.53874	0.634	0.76385542	ns
95	1.02390055	117.61392	0.308	0.38987342	ns	95	0.65295039	88.36892	0.515	0.64375	ns
96	0.9593629	116.98713	0.339	0.41341463	ns	96	0.82189492	88.53993	0.413	0.52948718	ns
97	0.82596559	115.80035	0.411	0.48928571	ns	97	0.93890666	87.41252	0.35	0.46666667	ns
98	0.64333389	115.16323	0.521	0.59204545	ns	98	0.98264917	85.1343	0.329	0.45068493	ns
99	0.43055595	115.57483	0.668	0.73406593	ns	99	0.949569	82.36523	0.345	0.46621622	ns
100	0.17284379	116.69946	0.863	0.87373737	ns	100	0.86362845	79.89008	0.39	0.51315789	ns
19	statistic	df	p	p.adj	p.adj.signif	20	statistic	df	p	p.adj	p.adj.signif
1	2.50230095	120.4088	0.0137	0.2928571	ns	1	2.50230095	120.4088	0.0137	0.2928571	ns
2	2.46143236	118.9996	0.0153	0.2928571	ns	2	2.46143236	118.9996	0.0153	0.2928571	ns
3	2.41043909	117.8508	0.0175	0.2928571	ns	3	2.41043909	117.8508	0.0175	0.2928571	ns
4	2.34162329	117.1876	0.0209	0.2928571	ns	4	2.34162329	117.1876	0.0209	0.2928571	ns
5	2.29236535	117.477	0.0237	0.2928571	ns	5	2.29236535	117.477	0.0237	0.2928571	ns
6	2.23727909	118.0497	0.0271	0.2928571	ns	6	2.23727909	118.0497	0.0271	0.2928571	ns
7	2.17857609	118.7058	0.0313	0.2928571	ns	7	2.17857609	118.7058	0.0313	0.2928571	ns
8	2.09474138	119.2794	0.0383	0.2928571	ns	8	2.09474138	119.2794	0.0383	0.2928571	ns
9	1.9993233	119.6902	0.0478	0.2928571	ns	9	1.9993233	119.6902	0.0478	0.2928571	ns
10	1.8882755	119.7254	0.0614	0.2928571	ns	10	1.8882755	119.7254	0.0614	0.2928571	ns
11	1.72175597	119.2938	0.0877	0.3373077	ns	11	1.72175597	119.2938	0.0877	0.3373077	ns

12	1.50071045	118.5199	0.136	0.4387097	ns	12	1.50071045	118.5199	0.136	0.4387097	ns
13	1.25367332	117.4322	0.212	0.572973	ns	13	1.25367332	117.4322	0.212	0.572973	ns
14	0.99152431	116.3756	0.323	0.6588235	ns	14	0.99152431	116.3756	0.323	0.6588235	ns
15	0.74408611	115.7635	0.458	0.7365079	ns	15	0.74408611	115.7635	0.458	0.7365079	ns
16	0.54199653	115.5827	0.589	0.7959459	ns	16	0.54199653	115.5827	0.589	0.7959459	ns
17	0.38842303	115.8019	0.698	0.8512195	ns	17	0.38842303	115.8019	0.698	0.8512195	ns
18	0.28504014	116.4124	0.776	0.9046512	ns	18	0.28504014	116.4124	0.776	0.9046512	ns
19	0.24969801	116.8483	0.803	0.9055556	ns	19	0.24969801	116.8483	0.803	0.9055556	ns
20	0.28210469	117.0357	0.778	0.9046512	ns	20	0.28210469	117.0357	0.778	0.9046512	ns
21	0.35453446	117.0391	0.724	0.8722892	ns	21	0.35453446	117.0391	0.724	0.8722892	ns
22	0.46492859	117.1821	0.643	0.824359	ns	22	0.46492859	117.1821	0.643	0.824359	ns
23	0.62759807	117.754	0.531	0.7685714	ns	23	0.62759807	117.754	0.531	0.7685714	ns
24	0.80225025	118.5463	0.424	0.7365079	ns	24	0.80225025	118.5463	0.424	0.7365079	ns
25	0.93344391	119.3923	0.352	0.6641509	ns	25	0.93344391	119.3923	0.352	0.6641509	ns
26	0.97458327	120.335	0.332	0.6588235	ns	26	0.97458327	120.335	0.332	0.6588235	ns
27	0.90808347	121.4313	0.366	0.6777778	ns	27	0.90808347	121.4313	0.366	0.6777778	ns
28	0.74233304	122.6588	0.459	0.7365079	ns	28	0.74233304	122.6588	0.459	0.7365079	ns
29	0.47750099	123.986	0.634	0.824359	ns	29	0.47750099	123.986	0.634	0.824359	ns
30	0.13756197	125.2881	0.891	0.9580645	ns	30	0.13756197	125.2881	0.891	0.9580645	ns
31	-0.240774	126.2744	0.81	0.9055556	ns	31	-0.240774	126.2744	0.81	0.9055556	ns
32	-0.62650973	126.8113	0.532	0.7685714	ns	32	-0.62650973	126.8113	0.532	0.7685714	ns
33	-0.99547655	126.9006	0.321	0.6588235	ns	33	-0.99547655	126.9006	0.321	0.6588235	ns
34	-1.32828892	126.7327	0.186	0.5314286	ns	34	-1.32828892	126.7327	0.186	0.5314286	ns
35	-1.61029119	126.4758	0.11	0.3793103	ns	35	-1.61029119	126.4758	0.11	0.3793103	ns
36	-1.81455461	126.049	0.072	0.3044	ns	36	-1.81455461	126.049	0.072	0.3044	ns

37	-1.93063584	125.298	0.0558	0.2928571	ns	37	-1.93063584	125.298	0.0558	0.2928571	ns
38	-1.97450821	124.3288	0.0505	0.2928571	ns	38	-1.97450821	124.3288	0.0505	0.2928571	ns
39	-1.95888626	123.4481	0.0524	0.2928571	ns	39	-1.95888626	123.4481	0.0524	0.2928571	ns
40	-1.88726494	122.8239	0.0615	0.2928571	ns	40	-1.88726494	122.8239	0.0615	0.2928571	ns
41	-1.79441671	122.1512	0.0752	0.3044	ns	41	-1.79441671	122.1512	0.0752	0.3044	ns
42	-1.65335515	121.4815	0.101	0.3740741	ns	42	-1.65335515	121.4815	0.101	0.3740741	ns
43	-1.46618341	121.2177	0.145	0.453125	ns	43	-1.46618341	121.2177	0.145	0.453125	ns
44	-1.28387611	120.9208	0.202	0.5611111	ns	44	-1.28387611	120.9208	0.202	0.5611111	ns
45	-1.13689127	119.9623	0.258	0.645	ns	45	-1.13689127	119.9623	0.258	0.645	ns
46	-1.06668924	118.1651	0.288	0.6586957	ns	46	-1.06668924	118.1651	0.288	0.6586957	ns
47	-1.05001596	115.7487	0.296	0.6586957	ns	47	-1.05001596	115.7487	0.296	0.6586957	ns
48	-1.0556749	113.1095	0.293	0.6586957	ns	48	-1.0556749	113.1095	0.293	0.6586957	ns
49	-1.0644613	110.2673	0.289	0.6586957	ns	49	-1.0644613	110.2673	0.289	0.6586957	ns
50	-1.04805239	107.7329	0.297	0.6586957	ns	50	-1.04805239	107.7329	0.297	0.6586957	ns
51	-1.03490887	106.0157	0.303	0.6586957	ns	51	-1.03490887	106.0157	0.303	0.6586957	ns
52	-0.98481856	105.6867	0.327	0.6588235	ns	52	-0.98481856	105.6867	0.327	0.6588235	ns
53	-0.87980541	106.4339	0.381	0.6927273	ns	53	-0.87980541	106.4339	0.381	0.6927273	ns
54	-0.78907521	107.9555	0.432	0.7365079	ns	54	-0.78907521	107.9555	0.432	0.7365079	ns
55	-0.73433377	110.5131	0.464	0.7365079	ns	55	-0.73433377	110.5131	0.464	0.7365079	ns
56	-0.68867729	113.7743	0.492	0.7621212	ns	56	-0.68867729	113.7743	0.492	0.7621212	ns
57	-0.57387074	116.9211	0.567	0.7875	ns	57	-0.57387074	116.9211	0.567	0.7875	ns
58	-0.38852982	119.4292	0.698	0.8512195	ns	58	-0.38852982	119.4292	0.698	0.8512195	ns
59	-0.20767405	121.4759	0.836	0.9186813	ns	59	-0.20767405	121.4759	0.836	0.9186813	ns
60	-0.09990219	123.2723	0.921	0.9656566	ns	60	-0.09990219	123.2723	0.921	0.9656566	ns
61	-0.06330164	124.8973	0.95	0.9656566	ns	61	-0.06330164	124.8973	0.95	0.9656566	ns

62	-0.05470466	126.3141	0.956	0.9656566	ns	62	-0.05470466	126.3141	0.956	0.9656566	ns
63	-0.00438743	127.2863	0.997	0.997	ns	63	-0.00438743	127.2863	0.997	0.997	ns
64	0.08123046	127.9097	0.935	0.9656566	ns	64	0.08123046	127.9097	0.935	0.9656566	ns
65	0.23454125	127.8502	0.815	0.9055556	ns	65	0.23454125	127.8502	0.815	0.9055556	ns
66	0.4349266	126.7024	0.664	0.8405063	ns	66	0.4349266	126.7024	0.664	0.8405063	ns
67	0.59314672	124.3508	0.554	0.7802817	ns	67	0.59314672	124.3508	0.554	0.7802817	ns
68	0.65162759	121.2197	0.516	0.7685714	ns	68	0.65162759	121.2197	0.516	0.7685714	ns
69	0.67169482	118.6102	0.503	0.7621212	ns	69	0.67169482	118.6102	0.503	0.7621212	ns
70	0.77327856	118.2753	0.441	0.7365079	ns	70	0.77327856	118.2753	0.441	0.7365079	ns
71	0.94708276	119.6555	0.346	0.6641509	ns	71	0.94708276	119.6555	0.346	0.6641509	ns
72	1.1568634	120.785	0.25	0.6410256	ns	72	1.1568634	120.785	0.25	0.6410256	ns
73	1.36812233	121.8981	0.174	0.5117647	ns	73	1.36812233	121.8981	0.174	0.5117647	ns
74	1.57851336	123.9826	0.117	0.39	ns	74	1.57851336	123.9826	0.117	0.39	ns
75	1.78866673	126.1013	0.0761	0.3044	ns	75	1.78866673	126.1013	0.0761	0.3044	ns
76	1.94558201	127.3497	0.0539	0.2928571	ns	76	1.94558201	127.3497	0.0539	0.2928571	ns
77	2.02014293	127.9339	0.0455	0.2928571	ns	77	2.02014293	127.9339	0.0455	0.2928571	ns
78	2.05452035	127.8828	0.042	0.2928571	ns	78	2.05452035	127.8828	0.042	0.2928571	ns
79	2.07454774	127.1495	0.04	0.2928571	ns	79	2.07454774	127.1495	0.04	0.2928571	ns
80	2.09172933	125.9317	0.0385	0.2928571	ns	80	2.09172933	125.9317	0.0385	0.2928571	ns
81	2.06750108	124.0718	0.0408	0.2928571	ns	81	2.06750108	124.0718	0.0408	0.2928571	ns
82	1.96526353	121.4848	0.0517	0.2928571	ns	82	1.96526353	121.4848	0.0517	0.2928571	ns
83	1.81011914	119.1566	0.0728	0.3044	ns	83	1.81011914	119.1566	0.0728	0.3044	ns
84	1.63134156	117.9576	0.105	0.375	ns	84	1.63134156	117.9576	0.105	0.375	ns
85	1.41842632	117.5809	0.159	0.4818182	ns	85	1.41842632	117.5809	0.159	0.4818182	ns
86	1.17469206	116.9015	0.243	0.6394737	ns	86	1.17469206	116.9015	0.243	0.6394737	ns

87	0.96596337	115.6531	0.336	0.6588235	ns	87	0.96596337	115.6531	0.336	0.6588235	ns
88	0.82628454	114.7278	0.41	0.7321429	ns	88	0.82628454	114.7278	0.41	0.7321429	ns
89	0.74164102	115.0164	0.46	0.7365079	ns	89	0.74164102	115.0164	0.46	0.7365079	ns
90	0.68057587	116.9953	0.497	0.7621212	ns	90	0.68057587	116.9953	0.497	0.7621212	ns
91	0.61802689	119.7505	0.538	0.7685714	ns	91	0.61802689	119.7505	0.538	0.7685714	ns
92	0.56169226	121.9886	0.575	0.7876712	ns	92	0.56169226	121.9886	0.575	0.7876712	ns
93	0.51693061	123.7264	0.606	0.808	ns	93	0.51693061	123.7264	0.606	0.808	ns
94	0.46753599	124.3987	0.641	0.824359	ns	94	0.46753599	124.3987	0.641	0.824359	ns
95	0.4153442	124.088	0.679	0.84875	ns	95	0.4153442	124.088	0.679	0.84875	ns
96	0.33483683	122.7776	0.738	0.8785714	ns	96	0.33483683	122.7776	0.738	0.8785714	ns
97	0.2438977	121.4798	0.808	0.9055556	ns	97	0.2438977	121.4798	0.808	0.9055556	ns
98	0.15395487	120.9887	0.878	0.9543478	ns	98	0.15395487	120.9887	0.878	0.9543478	ns
99	0.11013592	121.7152	0.912	0.9656566	ns	99	0.11013592	121.7152	0.912	0.9656566	ns
100	0.06403194	122.84	0.949	0.9656566	ns	100	0.06403194	122.84	0.949	0.9656566	ns

Table S4： Comparison of Mean MD Between prodromal PD carriers of dual LRRK2 and GBA variants and Healthy Controls Across 20 Fiber Tracts. Statistical significance was set at $p < 0.05$, corrected by FDR.

1	statistic	df	p	p.adj	p.adj.signif	2	statistic	df	p	p.adj	p.adj.signif
1	1.742443803	127.16912	8.40E-02	0.168	ns	1	0.316920384	127.77557	0.752	0.998	ns
2	1.782534949	126.93678	7.70E-02	0.166666667	ns	2	0.382275561	127.94356	0.703	0.998	ns
3	1.818100897	126.64406	7.10E-02	0.157777778	ns	3	0.444210675	127.99998	0.658	0.998	ns
4	1.846654587	126.2349	6.70E-02	0.152272727	ns	4	0.482475164	127.98034	0.63	0.998	ns
5	1.887017312	125.78374	6.20E-02	0.152272727	ns	5	0.497964216	127.96885	0.619	0.998	ns
6	1.928029299	125.38158	5.60E-02	0.146153846	ns	6	0.481711873	127.98045	0.631	0.998	ns
7	1.978170657	125.23509	5.00E-02	0.135135135	ns	7	0.449311775	127.98935	0.654	0.998	ns
8	2.028566573	125.27076	4.50E-02	0.125	ns	8	0.421251876	127.99175	0.674	0.998	ns
9	2.066939716	125.36312	4.10E-02	0.122857143	ns	9	0.393959672	127.98572	0.694	0.998	ns
10	2.094346602	125.4797	3.80E-02	0.11875	ns	10	0.368702657	127.98116	0.713	0.998	ns
11	2.10878523	125.62947	3.70E-02	0.11875	ns	11	0.341257369	127.9873	0.733	0.998	ns
12	2.099228306	125.83618	3.80E-02	0.11875	ns	12	0.323418967	127.9964	0.747	0.998	ns
13	2.042608055	125.958	4.30E-02	0.122857143	ns	13	0.326044082	127.99771	0.745	0.998	ns
14	1.924005723	125.80321	5.70E-02	0.146153846	ns	14	0.339897981	127.94486	0.734	0.998	ns
15	1.75108707	125.05984	8.20E-02	0.167346939	ns	15	0.341705984	127.69856	0.733	0.998	ns
16	1.544725748	123.37122	1.25E-01	0.231481481	ns	16	0.316969446	126.9202	0.752	0.998	ns
17	1.326178801	120.69941	1.87E-01	0.316949153	ns	17	0.274819589	125.30601	0.784	0.998	ns
18	1.121353684	117.11152	2.64E-01	0.4	ns	18	0.206348907	122.7788	0.837	0.998	ns
19	0.953736272	113.02409	3.42E-01	0.466216216	ns	19	0.121384863	119.50807	0.904	0.998	ns
20	0.820033951	108.86177	4.14E-01	0.544736842	ns	20	0.05332149	115.9975	0.958	0.998	ns
21	0.696643993	104.88025	4.88E-01	0.633333333	ns	21	0.006586707	112.641	0.995	0.998	ns
22	0.583097436	101.56243	5.61E-01	0.710126582	ns	22	0.00208688	109.83996	0.998	0.998	ns
23	0.481921173	99.13459	6.31E-01	0.779012346	ns	23	0.054346436	107.54113	0.957	0.998	ns

24	0.38073095	97.91938	7.04E-01	0.848192771	ns	24	0.163836613	105.66865	0.87	0.998	ns
25	0.274782199	97.95217	7.84E-01	0.922352941	ns	25	0.298698512	104.08704	0.766	0.998	ns
26	0.180174873	98.97245	8.57E-01	0.984090909	ns	26	0.411597068	103.08151	0.681	0.998	ns
27	0.104840824	100.26208	9.17E-01	0.994	ns	27	0.468962369	102.88356	0.64	0.998	ns
28	0.0302789	101.28316	9.76E-01	0.994	ns	28	0.477473417	103.09523	0.634	0.998	ns
29	-0.026849423	101.80188	9.79E-01	0.994	ns	29	0.452436283	103.02693	0.652	0.998	ns
30	-0.055002217	101.93648	9.56E-01	0.994	ns	30	0.41379565	102.52595	0.68	0.998	ns
31	-0.04423342	101.95269	9.65E-01	0.994	ns	31	0.376652807	101.5787	0.707	0.998	ns
32	-0.016111208	101.55428	9.87E-01	0.994	ns	32	0.356744333	100.23879	0.722	0.998	ns
33	0.013679466	100.57406	9.89E-01	0.994	ns	33	0.369045062	98.72847	0.713	0.998	ns
34	0.021790578	99.35059	9.83E-01	0.994	ns	34	0.425532712	97.44705	0.671	0.998	ns
35	0.008056045	98.67609	9.94E-01	0.994	ns	35	0.534046042	96.75535	0.595	0.998	ns
36	-0.011311588	98.81813	9.91E-01	0.994	ns	36	0.668857818	96.53461	0.505	0.998	ns
37	-0.046854093	99.27269	9.63E-01	0.994	ns	37	0.788286125	96.72642	0.432	0.998	ns
38	-0.10789767	99.93187	9.14E-01	0.994	ns	38	0.842387765	97.24071	0.402	0.998	ns
39	-0.169367882	101.07734	8.66E-01	0.984090909	ns	39	0.82831743	97.5826	0.41	0.998	ns
40	-0.232569524	102.75105	8.17E-01	0.95	ns	40	0.786135372	97.31281	0.434	0.998	ns
41	-0.308035791	105.14953	7.59E-01	0.903571429	ns	41	0.754906902	96.20756	0.452	0.998	ns
42	-0.411694831	108.02696	6.81E-01	0.830487805	ns	42	0.776209066	94.64867	0.44	0.998	ns
43	-0.540107585	110.18686	5.90E-01	0.7375	ns	43	0.824194504	93.39785	0.412	0.998	ns
44	-0.685636884	110.41437	4.94E-01	0.633333333	ns	44	0.881488438	92.56685	0.38	0.998	ns
45	-0.82793107	109.0339	4.10E-01	0.544736842	ns	45	0.949053637	91.48837	0.345	0.998	ns
46	-0.948501961	106.87685	3.45E-01	0.466216216	ns	46	1.007357448	89.80543	0.316	0.998	ns
47	-1.02984422	104.82816	3.05E-01	0.423611111	ns	47	1.023791269	87.59242	0.309	0.998	ns
48	-1.082648477	102.50468	2.82E-01	0.413043478	ns	48	0.984219098	85.4499	0.328	0.998	ns

49	-1.123512328	99.03517	2.64E-01	0.4	ns	49	0.912192336	83.90541	0.364	0.998	ns
50	-1.189004562	93.94385	2.37E-01	0.376190476	ns	50	0.842748217	83.05502	0.402	0.998	ns
51	-1.304142743	87.52813	1.96E-01	0.326666667	ns	51	0.801221025	82.93141	0.425	0.998	ns
52	-1.464823261	81.66955	1.47E-01	0.257894737	ns	52	0.80952101	83.66509	0.421	0.998	ns
53	-1.631624339	77.43347	1.07E-01	0.201886792	ns	53	0.844481961	85.00935	0.401	0.998	ns
54	-1.774124798	74.96786	8.00E-02	0.166666667	ns	54	0.872245267	86.56903	0.385	0.998	ns
55	-1.864976133	73.96097	6.60E-02	0.152272727	ns	55	0.878663676	88.3635	0.382	0.998	ns
56	-1.861851282	73.78533	6.70E-02	0.152272727	ns	56	0.854998572	90.08048	0.395	0.998	ns
57	-1.775517661	74.30336	8.00E-02	0.166666667	ns	57	0.807064794	91.25884	0.422	0.998	ns
58	-1.650921165	75.29411	1.03E-01	0.198076923	ns	58	0.733339961	91.55761	0.465	0.998	ns
59	-1.505882399	76.32068	1.36E-01	0.247272727	ns	59	0.620930895	91.31569	0.536	0.998	ns
60	-1.359012277	77.31817	1.78E-01	0.306896552	ns	60	0.481630261	91.05261	0.631	0.998	ns
61	-1.22403647	78.65892	2.25E-01	0.362903226	ns	61	0.343077789	91.35412	0.732	0.998	ns
62	-1.113355625	80.49988	2.69E-01	0.401492537	ns	62	0.220180667	92.61775	0.826	0.998	ns
63	-1.048604129	82.97622	2.97E-01	0.423611111	ns	63	0.118854673	94.9643	0.906	0.998	ns
64	-1.038760535	85.72073	3.02E-01	0.423611111	ns	64	0.040931715	97.63902	0.967	0.998	ns
65	-1.075184919	87.99767	2.85E-01	0.413043478	ns	65	-0.014149226	99.29954	0.989	0.998	ns
66	-1.155630531	89.52754	2.51E-01	0.3921875	ns	66	-0.052694111	99.05432	0.958	0.998	ns
67	-1.28517566	90.4051	2.02E-01	0.331147541	ns	67	-0.0797965	97.01962	0.937	0.998	ns
68	-1.462518947	91.20282	1.47E-01	0.257894737	ns	68	-0.076835001	93.90091	0.939	0.998	ns
69	-1.667860568	92.39702	9.90E-02	0.194117647	ns	69	-0.034776028	90.65552	0.972	0.998	ns
70	-1.865504321	93.92283	6.50E-02	0.152272727	ns	70	0.039476223	87.88512	0.969	0.998	ns
71	-2.052467786	95.99145	4.30E-02	0.122857143	ns	71	0.118606412	85.65319	0.906	0.998	ns
72	-2.242022336	98.97651	2.70E-02	0.093103448	ns	72	0.20347665	83.8959	0.839	0.998	ns
73	-2.411602587	102.36823	1.80E-02	0.064285714	ns	73	0.284832395	82.61205	0.776	0.998	ns

74	-2.548201455	105.52473	1.20E-02	0.044444444	*	74	0.362435802	81.90073	0.718	0.998	ns
75	-2.647266097	107.77422	9.00E-03	0.034615385	*	75	0.4244844	81.85097	0.672	0.998	ns
76	-2.767385386	109.57244	7.00E-03	0.033333333	*	76	0.45080336	82.64079	0.653	0.998	ns
77	-2.921728235	110.88612	4.00E-03	0.023529412	*	77	0.434310209	83.97621	0.665	0.998	ns
78	-3.075475145	111.13043	3.00E-03	0.02	*	78	0.379293411	85.28012	0.705	0.998	ns
79	-3.206477829	110.31828	2.00E-03	0.014285714	*	79	0.304905388	86.29922	0.761	0.998	ns
80	-3.315848406	109.17682	1.00E-03	0.007692308	**	80	0.231655341	86.93039	0.817	0.998	ns
81	-3.384759507	108.14609	9.93E-04	0.007692308	**	81	0.155544817	87.03675	0.877	0.998	ns
82	-3.449176947	107.46859	8.04E-04	0.007692308	**	82	0.060293131	86.58567	0.952	0.998	ns
83	-3.530498966	107.49604	6.12E-04	0.0068	**	83	-0.05723977	85.91501	0.954	0.998	ns
84	-3.628565509	108.31792	4.37E-04	0.0054625	**	84	-0.178968776	85.44541	0.858	0.998	ns
85	-3.753709341	110.18472	2.80E-04	0.004666667	**	85	-0.289098196	85.60797	0.773	0.998	ns
86	-3.894182112	112.89899	1.67E-04	0.00334	**	86	-0.410654085	86.50739	0.682	0.998	ns
87	-4.02448122	116.73538	1.02E-04	0.00334	**	87	-0.535467924	87.87698	0.594	0.998	ns
88	-4.099722892	121.2626	7.52E-05	0.00334	**	88	-0.606111292	89.63642	0.546	0.998	ns
89	-4.069278854	125.04477	8.30E-05	0.00334	**	89	-0.577779148	91.55061	0.565	0.998	ns
90	-3.902969933	127.22505	1.53E-04	0.00334	**	90	-0.425264825	93.4099	0.672	0.998	ns
91	-3.613560921	127.96281	4.33E-04	0.0054625	**	91	-0.139092291	95.31657	0.89	0.998	ns
92	-3.259679381	127.90895	1.00E-03	0.007692308	**	92	0.221650972	96.07306	0.825	0.998	ns
93	-2.954849434	127.6296	4.00E-03	0.023529412	*	93	0.558084919	95.89643	0.578	0.998	ns
94	-2.768636019	127.49278	6.00E-03	0.031578947	*	94	0.776704425	96.64681	0.439	0.998	ns
95	-2.697512616	127.55141	8.00E-03	0.033333333	*	95	0.829845957	98.02914	0.409	0.998	ns
96	-2.68904309	127.70566	8.00E-03	0.033333333	*	96	0.766837255	99.16882	0.445	0.998	ns
97	-2.690960664	127.91106	8.00E-03	0.033333333	*	97	0.64153271	99.54394	0.523	0.998	ns
98	-2.663510421	127.9734	9.00E-03	0.034615385	*	98	0.475703874	100.27861	0.635	0.998	ns

99	-2.68463784	127.32702	8.00E-03	0.033333333	*	99	0.318360326	101.71669	0.751	0.998	ns
100	-2.79321721	125.83874	6.00E-03	0.031578947	*	100	0.271083784	102.91018	0.787	0.998	ns
3	statistic	df	p	p.adj	p.adj.signif	4	statistic	df	p	p.adj	p.adj.signif
1	0.21136517	123.88997	8.33E-01	0.867708333	ns	1	0.3477473	127.91961	0.729	0.9346154	ns
2	0.26328252	125.59538	7.93E-01	0.856989247	ns	2	0.20549157	127.75625	0.838	0.9536842	ns
3	0.25774465	126.41039	7.97E-01	0.856989247	ns	3	0.19228654	127.59076	0.848	0.9536842	ns
4	0.22033218	127.02649	8.26E-01	0.867708333	ns	4	0.32325166	127.67055	0.747	0.9444444	ns
5	0.14335345	127.26667	8.86E-01	0.904081633	ns	5	0.57458153	127.96487	0.567	0.9283784	ns
6	0.03157717	127.27771	9.75E-01	0.975	ns	6	0.78671565	127.98158	0.433	0.9283784	ns
7	-0.07640798	127.33914	9.39E-01	0.948484849	ns	7	0.88044319	127.97078	0.38	0.9283784	ns
8	-0.16679405	127.20993	8.68E-01	0.894845361	ns	8	0.92587309	127.84504	0.356	0.9283784	ns
9	-0.22751128	126.60101	8.20E-01	0.867708333	ns	9	0.91158287	126.08893	0.364	0.9283784	ns
10	-0.27943361	125.54669	7.80E-01	0.856989247	ns	10	0.87329215	121.68815	0.384	0.9283784	ns
11	-0.33824295	123.83122	7.36E-01	0.845454546	ns	11	0.80679574	115.88403	0.421	0.9283784	ns
12	-0.40550889	121.92467	6.86E-01	0.807058824	ns	12	0.75491575	110.46516	0.452	0.9283784	ns
13	-0.41547698	120.18051	6.79E-01	0.807058824	ns	13	0.77619472	106.38988	0.439	0.9283784	ns
14	-0.34913479	118.64588	7.28E-01	0.845454546	ns	14	0.82195538	104.02833	0.413	0.9283784	ns
15	-0.27726952	117.28771	7.82E-01	0.856989247	ns	15	0.83419991	103.15951	0.406	0.9283784	ns
16	-0.25877861	115.96627	7.96E-01	0.856989247	ns	16	0.85335745	103.38704	0.395	0.9283784	ns
17	-0.32718232	115.799	7.44E-01	0.845454546	ns	17	0.90393282	104.38498	0.368	0.9283784	ns
18	-0.50776187	116.98643	6.13E-01	0.738554217	ns	18	0.95194891	105.92933	0.343	0.9283784	ns
19	-0.81414126	118.74796	4.17E-01	0.508536585	ns	19	0.92446931	107.83786	0.357	0.9283784	ns
20	-1.15755401	120.56468	2.49E-01	0.307407407	ns	20	0.88834464	108.58103	0.376	0.9283784	ns
21	-1.41760439	122.46173	1.59E-01	0.227142857	ns	21	0.84350767	108.89778	0.401	0.9283784	ns
22	-1.51733349	123.86191	1.32E-01	0.194117647	ns	22	0.777463	109.50962	0.439	0.9283784	ns

23	-1.52472666	124.4559	1.30E-01	0.194029851	ns	23	0.76498039	110.00803	0.446	0.9283784	ns
24	-1.47530982	124.16296	1.43E-01	0.207246377	ns	24	0.88715236	110.55227	0.377	0.9283784	ns
25	-1.34276779	122.91144	1.82E-01	0.252777778	ns	25	1.14083911	110.10552	0.256	0.9283784	ns
26	-1.21517336	120.90697	2.27E-01	0.294805195	ns	26	1.44833302	108.50857	0.15	0.9283784	ns
27	-1.17181744	118.29243	2.44E-01	0.305	ns	27	1.71533111	105.88375	0.0892	0.9283784	ns
28	-1.19973732	114.35753	2.33E-01	0.294936709	ns	28	1.98494607	103.01243	0.0498	0.9283784	ns
29	-1.24638921	109.43405	2.15E-01	0.288	ns	29	2.07413835	98.31447	0.0407	0.9283784	ns
30	-1.26299059	104.62353	2.09E-01	0.28630137	ns	30	1.91545525	93.54119	0.0585	0.9283784	ns
31	-1.24422718	100.43701	2.16E-01	0.288	ns	31	1.60672704	89.78694	0.112	0.9283784	ns
32	-1.20566032	96.94008	2.31E-01	0.294936709	ns	32	1.27723308	86.07256	0.205	0.9283784	ns
33	-1.22625795	93.45496	2.23E-01	0.293421053	ns	33	1.03260925	82.79888	0.305	0.9283784	ns
34	-1.37204206	90.48992	1.73E-01	0.243661972	ns	34	0.88247781	82.08053	0.38	0.9283784	ns
35	-1.64220614	89.24794	1.04E-01	0.157575758	ns	35	0.75354862	84.03903	0.453	0.9283784	ns
36	-2.02953649	90.05639	4.54E-02	0.069846154	ns	36	0.6784513	86.96729	0.499	0.9283784	ns
37	-2.51090044	92.90887	1.38E-02	0.023389831	*	37	0.64929739	89.26493	0.518	0.9283784	ns
38	-3.00772397	97.45312	3.35E-03	0.006320755	**	38	0.61971805	90.03645	0.537	0.9283784	ns
39	-3.44965005	101.4197	8.19E-04	0.0021	**	39	0.53599831	88.8626	0.593	0.9283784	ns
40	-3.78575739	104.19633	2.56E-04	0.0011125	**	40	0.41609265	87.71695	0.678	0.9283784	ns
41	-4.01283553	107.89071	1.11E-04	0.000792857	***	41	0.25026204	87.42287	0.803	0.9536842	ns
42	-4.18685738	113.87678	5.60E-05	0.000622222	***	42	0.04785413	87.94868	0.962	0.9727273	ns
43	-4.26381444	119.30218	4.04E-05	0.0006125	***	43	-0.15025626	89.43647	0.881	0.9536842	ns
44	-4.21722367	122.34861	4.77E-05	0.0006125	***	44	-0.29990655	91.08391	0.765	0.9444444	ns
45	-4.13724328	123.1885	6.46E-05	0.000627692	***	45	-0.36900166	92.02529	0.713	0.9346154	ns
46	-4.09162167	122.7989	7.70E-05	0.000627692	***	46	-0.40988629	91.70658	0.683	0.9283784	ns
47	-4.07754603	121.51143	8.16E-05	0.000627692	***	47	-0.4107874	90.53479	0.682	0.9283784	ns

48	-4.11638166	119.83386	7.10E-05	0.000627692	***	48	-0.35394708	88.61034	0.724	0.9346154	ns
49	-4.2203942	118.8256	4.79E-05	0.0006125	***	49	-0.27004653	86.09707	0.788	0.9536842	ns
50	-4.39812034	119.72246	2.38E-05	0.000595	***	50	-0.18720096	84.32614	0.852	0.9536842	ns
51	-4.55674779	121.68472	1.24E-05	0.000595	***	51	-0.14279501	83.95173	0.887	0.9536842	ns
52	-4.58438291	123.89108	1.10E-05	0.000595	***	52	-0.1200757	84.76853	0.905	0.9536842	ns
53	-4.45709778	125.5043	1.82E-05	0.000595	***	53	-0.1245018	86.39165	0.901	0.9536842	ns
54	-4.20552347	126.10382	4.90E-05	0.0006125	***	54	-0.14743894	88.19741	0.883	0.9536842	ns
55	-3.9306363	126.00317	1.39E-04	0.00086875	***	55	-0.14392665	90.85307	0.886	0.9536842	ns
56	-3.67943646	125.01086	3.46E-04	0.001173333	**	56	-0.09136049	94.44586	0.927	0.965625	ns
57	-3.48098664	123.17716	6.92E-04	0.001821053	**	57	-0.01659025	98.68978	0.987	0.987	ns
58	-3.32747644	120.71232	1.16E-03	0.002577778	**	58	0.05919308	101.89161	0.953	0.9727273	ns
59	-3.21362402	118.32774	1.69E-03	0.003673913	**	59	0.11862269	102.16466	0.906	0.9536842	ns
60	-3.11681078	117.26121	2.30E-03	0.0046	**	60	0.20957791	99.34765	0.834	0.9536842	ns
61	-3.02421729	118.01025	3.06E-03	0.005884615	**	61	0.31064371	95.95319	0.757	0.9444444	ns
62	-2.88967158	119.45076	4.58E-03	0.008327273	**	62	0.40394861	93.45678	0.687	0.9283784	ns
63	-2.67664671	120.40385	8.47E-03	0.014859649	*	63	0.48471029	92.34894	0.629	0.9283784	ns
64	-2.43899567	120.63571	1.62E-02	0.027	*	64	0.57598393	92.65868	0.566	0.9283784	ns
65	-2.2385084	120.60548	2.70E-02	0.043548387	*	65	0.63204647	93.47957	0.529	0.9283784	ns
66	-2.14064961	120.81611	3.43E-02	0.05359375	ns	66	0.66261214	94.02431	0.509	0.9283784	ns
67	-2.19077223	121.15478	3.04E-02	0.048253968	*	67	0.67891369	94.13585	0.499	0.9283784	ns
68	-2.38115554	120.92857	1.88E-02	0.030819672	*	68	0.73875318	93.49008	0.462	0.9283784	ns
69	-2.60396529	119.28989	1.04E-02	0.017931035	*	69	0.82118737	92.74091	0.414	0.9283784	ns
70	-2.79704852	116.75476	6.03E-03	0.010767857	*	70	0.86059549	92.61138	0.392	0.9283784	ns
71	-2.92521759	114.08871	4.15E-03	0.007685185	**	71	0.84170551	93.15771	0.402	0.9283784	ns
72	-3.0348206	112.14154	2.99E-03	0.005862745	**	72	0.77673935	92.58565	0.439	0.9283784	ns

73	-3.20550574	110.892	1.76E-03	0.003744681	**	73	0.70206215	90.37104	0.484	0.9283784	ns
74	-3.40645398	108.95205	9.23E-04	0.002197619	**	74	0.6452018	87.62394	0.52	0.9283784	ns
75	-3.61323358	106.57631	4.63E-04	0.001322857	**	75	0.59279012	85.4873	0.555	0.9283784	ns
76	-3.77329753	105.03267	2.67E-04	0.0011125	**	76	0.59313593	84.38916	0.555	0.9283784	ns
77	-3.81935524	105.55344	2.26E-04	0.001109091	**	77	0.64197109	84.43272	0.523	0.9283784	ns
78	-3.75802375	107.68386	2.79E-04	0.001116	**	78	0.66948924	85.22392	0.505	0.9283784	ns
79	-3.66553386	110.2719	3.81E-04	0.0012	**	79	0.64309564	86.28889	0.522	0.9283784	ns
80	-3.65057275	112.32334	3.99E-04	0.001209091	**	80	0.5831103	86.83715	0.561	0.9283784	ns
81	-3.72383366	112.53013	3.08E-04	0.001166667	**	81	0.52359368	86.44702	0.602	0.9283784	ns
82	-3.79345826	109.71096	2.44E-04	0.001109091	**	82	0.4962856	85.8608	0.621	0.9283784	ns
83	-3.79904897	105.16299	2.44E-04	0.001109091	**	83	0.47794572	85.33386	0.634	0.9283784	ns
84	-3.71327885	102.80448	3.33E-04	0.001173333	**	84	0.45312171	84.41942	0.652	0.9283784	ns
85	-3.56020329	102.90815	5.63E-04	0.001563889	**	85	0.43871012	82.74233	0.662	0.9283784	ns
86	-3.41959574	103.6518	8.98E-04	0.002190244	**	86	0.44960139	81.14468	0.654	0.9283784	ns
87	-3.37420817	104.06083	1.04E-03	0.002363636	**	87	0.49495798	80.45376	0.622	0.9283784	ns
88	-3.4318773	104.1609	8.61E-04	0.0021525	**	88	0.58539691	81.23275	0.56	0.9283784	ns
89	-3.54688018	103.25077	5.88E-04	0.001589189	**	89	0.71673076	83.71252	0.476	0.9283784	ns
90	-3.63304458	100.91763	4.42E-04	0.0013	**	90	0.80259984	87.36928	0.424	0.9283784	ns
91	-3.67701019	98.5009	3.84E-04	0.0012	**	91	0.78311739	92.02641	0.436	0.9283784	ns
92	-3.73561156	97.85704	3.15E-04	0.001166667	**	92	0.63296674	96.63913	0.528	0.9283784	ns
93	-3.82357524	99.32099	2.30E-04	0.001109091	**	93	0.35548605	98.98996	0.723	0.9346154	ns
94	-3.9218435	102.67751	1.59E-04	0.000883333	***	94	0.046067	98.70556	0.963	0.9727273	ns
95	-3.98026863	107.16936	1.26E-04	0.00084	***	95	-0.2252501	96.85434	0.822	0.9536842	ns
96	-3.91463106	111.74672	1.56E-04	0.000883333	***	96	-0.42246092	95.04495	0.674	0.9283784	ns
97	-3.68296695	115.54392	3.52E-04	0.001173333	**	97	-0.53548755	95.64326	0.594	0.9283784	ns

98	-3.37590345	118.4346	9.96E-04	0.002316279	**	98	-0.61837356	99.27181	0.538	0.9283784	ns
99	-3.15746825	120.29971	2.01E-03	0.0041875	**	99	-0.65256978	104.52639	0.515	0.9283784	ns
100	-3.13200179	121.77277	2.17E-03	0.004428571	**	100	-0.59822866	108.13177	0.551	0.9283784	ns
5	statistic	df	p	p.adj	p.adj.signif	6	statistic	df	p	p.adj	p.adj.signif
1	0.16577096	107.6945	0.869	0.90520833	ns	1	-0.52177355	103.81626	0.603	0.8987805	ns
2	0.3269549	103.90108	0.744	0.90105263	ns	2	-0.17774324	104.58026	0.859	0.9410526	ns
3	0.09851686	100.7591	0.922	0.95051546	ns	3	0.27300383	102.89631	0.785	0.9325581	ns
4	-0.2256533	99.67869	0.822	0.90105263	ns	4	0.61042429	105.65913	0.543	0.8987805	ns
5	-0.54077824	96.57366	0.59	0.84246575	ns	5	0.62276049	106.0045	0.535	0.8987805	ns
6	-0.82295784	94.08146	0.413	0.75090909	ns	6	0.3624147	105.93055	0.718	0.8987805	ns
7	-0.96712176	93.78517	0.336	0.71489362	ns	7	0.18074971	107.87827	0.857	0.9410526	ns
8	-1.04131826	96.95489	0.3	0.71489362	ns	8	0.13548279	110.42591	0.892	0.9410526	ns
9	-1.10584989	102.51969	0.271	0.71315789	ns	9	0.16171432	110.55577	0.872	0.9410526	ns
10	-1.13419033	106.55597	0.259	0.7	ns	10	-0.03967157	110.00382	0.968	0.9808081	ns
11	-1.14917811	106.37445	0.253	0.7	ns	11	-0.33621918	107.78159	0.737	0.8987805	ns
12	-1.0228863	104.05912	0.309	0.71489362	ns	12	-0.62935678	104.99227	0.53	0.8987805	ns
13	-0.91905661	103.61458	0.36	0.71960784	ns	13	-0.86028282	104.4192	0.392	0.8987805	ns
14	-0.92464036	104.80816	0.357	0.71960784	ns	14	-0.85978206	105.21257	0.392	0.8987805	ns
15	-0.98106933	105.64014	0.329	0.71489362	ns	15	-0.68494375	106.52548	0.495	0.8987805	ns
16	-0.99964343	104.54144	0.32	0.71489362	ns	16	-0.39344044	107.47281	0.695	0.8987805	ns
17	-0.96713038	101.7812	0.336	0.71489362	ns	17	-0.036337	106.79817	0.971	0.9808081	ns
18	-0.92290004	97.28371	0.358	0.71960784	ns	18	0.42671414	103.90864	0.67	0.8987805	ns
19	-0.90625521	92.57029	0.367	0.71960784	ns	19	0.9083625	100.40118	0.366	0.8987805	ns
20	-0.83051607	90.83277	0.408	0.75090909	ns	20	1.26301596	96.85671	0.21	0.8987805	ns
21	-0.77452437	90.38121	0.441	0.75166667	ns	21	1.38731581	99.06094	0.168	0.8987805	ns

22	-0.82723984	93.07788	0.41	0.75090909	ns	22	1.29470731	103.60741	0.198	0.8987805	ns
23	-1.06571348	96.70025	0.289	0.71489362	ns	23	1.00087146	107.85608	0.319	0.8987805	ns
24	-1.31921988	100.86481	0.19	0.61212121	ns	24	0.68581279	110.01386	0.494	0.8987805	ns
25	-1.53595781	105.48066	0.128	0.52692308	ns	25	0.42910355	110.59914	0.669	0.8987805	ns
26	-1.65911384	107.9038	0.1	0.52631579	ns	26	0.39053901	111.69383	0.697	0.8987805	ns
27	-1.81685596	107.70852	0.072	0.48	ns	27	0.46588458	112.01792	0.642	0.8987805	ns
28	-1.97718463	107.29163	0.0506	0.42166667	ns	28	0.62423198	111.88763	0.534	0.8987805	ns
29	-2.08489847	107.5647	0.0394	0.394	ns	29	0.89686064	112.83067	0.372	0.8987805	ns
30	-1.97876154	110.00129	0.0503	0.42166667	ns	30	1.24036713	112.89012	0.217	0.8987805	ns
31	-1.76068654	114.05879	0.081	0.50625	ns	31	1.43149881	112.2443	0.155	0.8987805	ns
32	-1.41798832	117.40515	0.159	0.53	ns	32	1.32159221	110.43225	0.189	0.8987805	ns
33	-1.06204803	118.91176	0.29	0.71489362	ns	33	1.03031541	108.13191	0.305	0.8987805	ns
34	-0.61168939	121.39024	0.542	0.80895522	ns	34	0.7982401	107.44139	0.426	0.8987805	ns
35	-0.23556487	123.75603	0.814	0.90105263	ns	35	0.73703103	108.07862	0.463	0.8987805	ns
36	-0.04686089	124.68953	0.963	0.97272727	ns	36	0.76041705	107.76924	0.449	0.8987805	ns
37	-0.07938305	124.42538	0.937	0.95612245	ns	37	0.73231185	104.85336	0.466	0.8987805	ns
38	-0.26326968	123.13338	0.793	0.90105263	ns	38	0.67604971	102.48142	0.501	0.8987805	ns
39	-0.50835735	120.71291	0.612	0.84246575	ns	39	0.67220815	102.01278	0.503	0.8987805	ns
40	-0.75651645	118.16561	0.451	0.75166667	ns	40	0.70304862	101.75003	0.484	0.8987805	ns
41	-0.98720657	118.43219	0.326	0.71489362	ns	41	0.76747582	102.32847	0.445	0.8987805	ns
42	-1.18740521	121.71686	0.237	0.67714286	ns	42	0.75395106	107.71807	0.453	0.8987805	ns
43	-1.44731218	123.69397	0.15	0.53	ns	43	0.59710189	115.51733	0.552	0.8987805	ns
44	-1.8188892	125.52833	0.0713	0.48	ns	44	0.32396686	120.72321	0.747	0.9	ns
45	-2.42608683	126.73745	0.0167	0.20875	ns	45	0.01238721	124.15851	0.99	0.99	ns
46	-3.13858937	126.23374	0.00211	0.0422	*	46	-0.25660529	126.41383	0.798	0.9325581	ns

47	-3.69163631	121.53993	0.000335	0.01446667	*	47	-0.44631367	126.16836	0.656	0.8987805	ns
48	-3.83958212	114.13105	0.000203	0.01446667	*	48	-0.55560542	123.5898	0.579	0.8987805	ns
49	-3.62908305	109.23173	0.000434	0.01446667	*	49	-0.5787018	117.9742	0.564	0.8987805	ns
50	-3.2711975	108.10302	0.00144	0.036	*	50	-0.62498932	109.57144	0.533	0.8987805	ns
51	-2.86190142	107.47009	0.00506	0.08433333	ns	51	-0.77781325	100.23834	0.439	0.8987805	ns
52	-2.50025524	106.80782	0.0139	0.19857143	ns	52	-0.93306691	94.47154	0.353	0.8987805	ns
53	-2.19900877	108.72465	0.03	0.33333333	ns	53	-1.02586702	94.32276	0.308	0.8987805	ns
54	-1.87819826	113.40005	0.0629	0.48	ns	54	-1.04963168	96.52547	0.297	0.8987805	ns
55	-1.52931559	117.79896	0.129	0.52692308	ns	55	-1.10341649	100.04248	0.272	0.8987805	ns
56	-1.30009514	120.33245	0.196	0.61212121	ns	56	-1.26519195	102.40515	0.209	0.8987805	ns
57	-1.2814927	121.75298	0.202	0.61212121	ns	57	-1.50811587	103.7509	0.135	0.8987805	ns
58	-1.42575565	121.88325	0.156	0.53	ns	58	-1.76956471	102.63983	0.0798	0.8987805	ns
59	-1.54451927	120.3231	0.125	0.52692308	ns	59	-1.91426656	98.70567	0.0585	0.8987805	ns
60	-1.50549102	117.09786	0.135	0.52692308	ns	60	-1.92914856	93.97568	0.0567	0.8987805	ns
61	-1.46099361	113.55397	0.147	0.53	ns	61	-1.87727761	90.52619	0.0637	0.8987805	ns
62	-1.49678193	111.10695	0.137	0.52692308	ns	62	-1.74729528	89.40966	0.084	0.8987805	ns
63	-1.61242676	109.12825	0.11	0.52692308	ns	63	-1.53564668	88.75109	0.128	0.8987805	ns
64	-1.69367561	106.94565	0.0932	0.51777778	ns	64	-1.24828534	88.23189	0.215	0.8987805	ns
65	-1.69609844	102.82643	0.0929	0.51777778	ns	65	-0.91851994	87.43217	0.361	0.8987805	ns
66	-1.52495748	97.39097	0.131	0.52692308	ns	66	-0.55150333	87.16642	0.583	0.8987805	ns
67	-1.19984633	94.86493	0.233	0.67714286	ns	67	-0.15815912	86.95344	0.875	0.9410526	ns
68	-0.73496933	96.54778	0.464	0.76065574	ns	68	0.14318409	87.72985	0.886	0.9410526	ns
69	-0.29483889	99.56824	0.769	0.90105263	ns	69	0.37958894	89.32504	0.705	0.8987805	ns
70	0.02602588	102.48863	0.979	0.979	ns	70	0.52367904	89.17606	0.602	0.8987805	ns
71	0.18869291	105.49832	0.851	0.90105263	ns	71	0.60336281	86.54331	0.548	0.8987805	ns

72	0.25009184	105.7572	0.803	0.90105263	ns	72	0.47762326	84.49974	0.634	0.8987805	ns
73	0.23236807	100.02834	0.817	0.90105263	ns	73	0.1664025	83.8415	0.868	0.9410526	ns
74	0.22884527	92.52848	0.819	0.90105263	ns	74	-0.15267714	83.68136	0.879	0.9410526	ns
75	0.27309915	87.26154	0.785	0.90105263	ns	75	-0.37086652	85.36776	0.712	0.8987805	ns
76	0.25250417	85.00143	0.801	0.90105263	ns	76	-0.56462893	89.58842	0.574	0.8987805	ns
77	0.20859673	84.96494	0.835	0.90105263	ns	77	-0.76158544	93.70645	0.448	0.8987805	ns
78	0.18216163	88.59148	0.856	0.90105263	ns	78	-0.8117372	95.46908	0.419	0.8987805	ns
79	0.34835338	95.47488	0.728	0.90105263	ns	79	-0.61572845	96.84242	0.54	0.8987805	ns
80	0.58581373	104.25678	0.559	0.81014493	ns	80	-0.25183443	98.16266	0.802	0.9325581	ns
81	0.80284999	107.55882	0.424	0.75166667	ns	81	0.1111353	102.32961	0.912	0.942268	ns
82	0.85870321	104.67703	0.392	0.75090909	ns	82	0.35060571	105.69466	0.727	0.8987805	ns
83	0.76455379	100.04553	0.446	0.75166667	ns	83	0.49300396	108.07412	0.623	0.8987805	ns
84	0.67704897	96.46372	0.5	0.77692308	ns	84	0.50769905	110.74187	0.613	0.8987805	ns
85	0.52477528	93.06315	0.601	0.84246575	ns	85	0.3552007	110.0927	0.723	0.8987805	ns
86	0.30705808	92.05587	0.759	0.90105263	ns	86	0.13414979	106.87061	0.894	0.9410526	ns
87	0.19252827	93.4604	0.848	0.90105263	ns	87	-0.10839162	101.05374	0.914	0.942268	ns
88	0.22876563	95.01477	0.82	0.90105263	ns	88	-0.33814426	95.14528	0.736	0.8987805	ns
89	0.30688695	96.7493	0.76	0.90105263	ns	89	-0.57551119	90.66586	0.566	0.8987805	ns
90	0.32990882	96.85681	0.742	0.90105263	ns	90	-0.73451791	90.03496	0.465	0.8987805	ns
91	0.32834269	94.75281	0.743	0.90105263	ns	91	-0.83078778	92.92929	0.408	0.8987805	ns
92	0.31704147	94.7204	0.752	0.90105263	ns	92	-0.94148489	97.22	0.349	0.8987805	ns
93	0.34649504	97.51873	0.73	0.90105263	ns	93	-1.08075468	99.91234	0.282	0.8987805	ns
94	0.50504339	103.26407	0.615	0.84246575	ns	94	-1.17863778	102.99762	0.241	0.8987805	ns
95	0.69669916	109.67418	0.487	0.77301587	ns	95	-1.2451227	107.3059	0.216	0.8987805	ns
96	0.70594277	111.40091	0.482	0.77301587	ns	96	-1.27164586	111.22868	0.206	0.8987805	ns

97	0.62422982	108.52403	0.534	0.80895522	ns	97	-1.3371663	112.23267	0.184	0.8987805	ns
98	0.58871271	105.01823	0.557	0.81014493	ns	98	-1.41186215	110.06085	0.161	0.8987805	ns
99	0.66966478	103.11133	0.505	0.77692308	ns	99	-1.47621072	106.08324	0.143	0.8987805	ns
100	0.77618059	103.25635	0.439	0.75166667	ns	100	-1.47341093	101.9151	0.144	0.8987805	ns
7	statistic	df	p	p.adj	p.adj.signif	8	statistic	df	p	p.adj	p.adj.signif
						1	-0.975897321	99.37862402	0.331	0.651923077	ns
1	-4.3605714	118.15396	2.79E-05	0.000227692	***	2	-0.885095484	97.44959245	0.378	0.675	ns
2	-4.30731896	117.75974	3.44E-05	0.000229333	***	3	-0.960645159	96.01286564	0.339	0.651923077	ns
3	-4.22772975	117.98231	4.68E-05	0.000277647	***	4	-1.106121086	94.78191342	0.271	0.651923077	ns
4	-4.14740387	118.60251	6.35E-05	0.000334211	***	5	-1.194788203	94.29679639	0.235	0.651923077	ns
5	-4.08422306	119.76469	8.02E-05	0.000359259	***	6	-1.19157044	93.77418205	0.236	0.651923077	ns
6	-4.06801121	121.47205	8.47E-05	0.000359259	***	7	-1.119024172	93.02491886	0.266	0.651923077	ns
7	-4.07995779	122.97033	8.04E-05	0.000359259	***	8	-0.991027434	92.43335436	0.324	0.651923077	ns
8	-4.05349925	124.10438	8.84E-05	0.000359259	***	9	-0.841121707	92.93694786	0.402	0.675757576	ns
9	-4.02908196	124.46719	9.68E-05	0.000359259	***	10	-0.728682462	95.05253774	0.468	0.688235294	ns
10	-4.02887267	124.15343	9.70E-05	0.000359259	***	11	-0.672587817	98.04455364	0.503	0.698611111	ns
11	-4.08183934	123.09179	7.98E-05	0.000359259	***	12	-0.656431294	100.8499034	0.513	0.698666667	ns
12	-4.17695936	121.59448	5.59E-05	0.000310556	***	13	-0.696634086	104.4817569	0.488	0.698591549	ns
13	-4.34165238	120.7187	2.96E-05	0.000227692	***	14	-0.772720206	108.2749185	0.441	0.675757576	ns
14	-4.50212352	120.19096	1.57E-05	0.000192	***	15	-0.813190064	110.4712157	0.418	0.675757576	ns
15	-4.59529405	119.49778	1.08E-05	0.000192	***	16	-0.799368055	111.3081442	0.426	0.675757576	ns
16	-4.60177617	118.57055	1.06E-05	0.000192	***	17	-0.73646329	112.0495354	0.463	0.688235294	ns
17	-4.5830778	118.34962	1.14E-05	0.000192	***	18	-0.683224771	113.1810036	0.496	0.698591549	ns
18	-4.55070239	118.21685	1.31E-05	0.000192	***	19	-0.622058337	113.5443257	0.535	0.703947368	ns
19	-4.47214163	117.51186	1.80E-05	0.000192	***	20	-0.550615796	113.5196805	0.583	0.737974684	ns

20	-4.45677555	116.72358	1.92E-05	0.000192	***	21	-0.443104188	112.7999744	0.659	0.79047619	ns
21	-4.47081208	116.26001	1.82E-05	0.000192	***	22	-0.308277852	111.9798652	0.758	0.847777778	ns
22	-4.47128639	116.66243	1.81E-05	0.000192	***	23	-0.192335786	111.015964	0.848	0.90212766	ns
23	-4.46262984	117.20824	1.87E-05	0.000192	***	24	-0.153171254	110.2524738	0.879	0.906185567	ns
24	-4.40297592	118.37553	2.35E-05	0.000213636	***	25	-0.174840542	109.6768469	0.862	0.906185567	ns
25	-4.31551449	120.73165	3.28E-05	0.000229333	***	26	-0.243659223	110.7051821	0.808	0.87826087	ns
26	-4.21786137	124.00827	4.72E-05	0.000277647	***	27	-0.357732489	112.0831406	0.721	0.838372093	ns
27	-4.04024038	126.43096	9.21E-05	0.000359259	***	28	-0.56188749	112.5770105	0.575	0.737974684	ns
28	-3.77267739	127.64856	2.46E-04	0.000878571	***	29	-0.764610187	111.740205	0.446	0.675757576	ns
29	-3.48469788	127.94159	6.75E-04	0.00225	**	30	-0.921898421	108.5930264	0.359	0.652727273	ns
30	-3.22005392	127.94999	1.62E-03	0.004628571	**	31	-1.001764668	103.9870026	0.319	0.651923077	ns
31	-2.97325798	127.8147	3.52E-03	0.0088	**	32	-1.064058971	99.29533334	0.29	0.651923077	ns
32	-2.77124094	127.3089	6.42E-03	0.013102041	*	33	-1.056711026	95.79781674	0.293	0.651923077	ns
33	-2.66995063	126.11906	8.59E-03	0.01675	*	34	-1.049216556	93.29881258	0.297	0.651923077	ns
34	-2.6941872	123.91863	8.03E-03	0.01606	*	35	-1.08356289	92.20282116	0.281	0.651923077	ns
35	-2.81775837	121.33555	5.65E-03	0.012282609	*	36	-1.070995449	91.73979175	0.287	0.651923077	ns
36	-2.99104605	119.10712	3.38E-03	0.008666667	**	37	-0.974113083	91.23930351	0.333	0.651923077	ns
37	-3.17211633	116.11751	1.94E-03	0.005243243	**	38	-0.851693251	89.85022427	0.397	0.675757576	ns
38	-3.3507346	112.12391	1.10E-03	0.003333333	**	39	-0.76619383	87.83231527	0.446	0.675757576	ns
39	-3.48338777	108.09578	7.16E-04	0.002309677	**	40	-0.806340299	86.84682096	0.422	0.675757576	ns
40	-3.54281063	103.97488	5.94E-04	0.002048276	**	41	-0.944149511	87.87061983	0.348	0.652727273	ns
41	-3.45811575	99.62136	8.02E-04	0.00250625	**	42	-1.133276422	90.27964609	0.26	0.651923077	ns
42	-3.2999632	95.73473	1.36E-03	0.004	**	43	-1.318968369	92.33076568	0.19	0.651923077	ns
43	-3.19170187	93.95999	1.92E-03	0.005243243	**	44	-1.438445282	93.71208257	0.154	0.651923077	ns
44	-3.11512947	93.30728	2.44E-03	0.006421053	**	45	-1.477341322	95.37290558	0.143	0.651923077	ns

45	-2.98315145	92.26117	3.65E-03	0.008902439	**	46	-1.476042907	96.89553872	0.143	0.651923077	ns
46	-2.81680162	91.06155	5.95E-03	0.012659575	*	47	-1.454126929	96.9017789	0.149	0.651923077	ns
47	-2.62828639	90.49139	1.01E-02	0.018703704	*	48	-1.347657954	95.51377058	0.181	0.651923077	ns
48	-2.46716636	90.1908	1.55E-02	0.027192983	*	49	-1.193684773	93.18227798	0.236	0.651923077	ns
49	-2.31129922	90.12222	2.31E-02	0.039827586	*	50	-1.143792188	92.34894832	0.256	0.651923077	ns
50	-2.20509354	89.83213	3.00E-02	0.049180328	*	51	-1.164182253	92.1551214	0.247	0.651923077	ns
51	-2.18448997	89.05079	3.16E-02	0.050967742	ns	52	-1.155615215	91.20670865	0.251	0.651923077	ns
52	-2.27485203	88.61181	2.53E-02	0.042166667	*	53	-1.086772182	90.38600737	0.28	0.651923077	ns
53	-2.46992659	89.93943	1.54E-02	0.027192983	*	54	-0.976396176	89.86339207	0.331	0.651923077	ns
54	-2.6800479	92.58282	8.71E-03	0.01675	*	55	-0.834340226	90.6272001	0.406	0.675757576	ns
55	-2.84579607	95.24251	5.42E-03	0.012044444	*	56	-0.693482564	93.20139784	0.49	0.698591549	ns
56	-2.95491314	97.66216	3.92E-03	0.009116279	**	57	-0.552418839	96.67262882	0.582	0.737974684	ns
57	-2.95919754	99.5438	3.85E-03	0.009116279	**	58	-0.397796129	99.79129428	0.692	0.814117647	ns
58	-2.88916989	100.83786	4.73E-03	0.01075	*	59	-0.255264118	102.7852538	0.799	0.878021978	ns
59	-2.7884045	101.68733	6.32E-03	0.013102041	*	60	-0.158160232	105.5131794	0.875	0.906185567	ns
60	-2.63960626	101.77819	9.61E-03	0.018132076	*	61	-0.114457393	108.509085	0.909	0.918181818	ns
61	-2.47853547	100.77994	1.49E-02	0.027090909	*	62	-0.138623363	111.6133612	0.89	0.908163265	ns
62	-2.29971951	98.65877	2.36E-02	0.04	*	63	-0.227858414	114.1406659	0.82	0.88172043	ns
63	-2.11149279	95.68006	3.73E-02	0.059206349	ns	64	-0.336778569	115.5398344	0.737	0.847126437	ns
64	-1.89405429	92.48975	6.13E-02	0.09578125	ns	65	-0.435871155	116.1594963	0.664	0.79047619	ns
65	-1.61108025	89.3967	1.11E-01	0.170769231	ns	66	-0.490464179	116.6153805	0.625	0.771604938	ns
66	-1.28655675	86.76291	2.02E-01	0.306060606	ns	67	-0.459811281	117.0047184	0.647	0.78902439	ns
67	-0.92990403	84.42719	3.55E-01	0.514492754	ns	68	-0.309150988	116.7602749	0.758	0.847777778	ns
68	-0.58717313	81.99477	5.59E-01	0.70125	ns	69	-0.035950682	116.6270938	0.971	0.971	ns
69	-0.3084939	79.56413	7.59E-01	0.807446809	ns	70	0.302213314	116.578174	0.763	0.847777778	ns

70	-0.09345962	78.04152	9.26E-01	0.944897959	ns	71	0.647414067	115.9577738	0.519	0.698666667	ns
71	0.06160277	77.69757	9.51E-01	0.951	ns	72	0.993512235	115.2005824	0.323	0.651923077	ns
72	0.21476708	77.97572	8.31E-01	0.865625	ns	73	1.347538097	114.7780314	0.18	0.651923077	ns
73	0.39674694	78.23803	6.93E-01	0.785714286	ns	74	1.707230158	114.9670764	0.0905	0.502777778	ns
74	0.59415571	78.2339	5.54E-01	0.70125	ns	75	2.022134467	115.78361	0.0455	0.315333333	ns
75	0.76077075	77.98487	4.49E-01	0.598666667	ns	76	2.299932225	117.6433418	0.0232	0.210909091	ns
76	0.85844371	77.86909	3.93E-01	0.551388889	ns	77	2.527390502	120.3730144	0.0128	0.155555556	ns
77	0.88618342	78.46975	3.78E-01	0.54	ns	78	2.712621609	122.8783886	0.00763	0.127166667	ns
78	0.85203335	79.87588	3.97E-01	0.551388889	ns	79	2.860253675	124.2093628	0.00497	0.12425	ns
79	0.79299409	81.87205	4.30E-01	0.589041096	ns	80	2.947573842	124.6876802	0.00382	0.12425	ns
80	0.7313801	85.49237	4.67E-01	0.614473684	ns	81	2.962780065	124.8128622	0.00365	0.12425	ns
81	0.66192913	90.09635	5.10E-01	0.662337662	ns	82	2.899014122	124.8034218	0.00442	0.12425	ns
82	0.56910195	94.75618	5.71E-01	0.704938272	ns	83	2.779230727	124.7109462	0.00629	0.1258	ns
83	0.47568629	99.2834	6.35E-01	0.774390244	ns	84	2.64089259	124.312414	0.00933	0.133285714	ns
84	0.40551585	104.71507	6.86E-01	0.785714286	ns	85	2.492929522	123.4010354	0.014	0.155555556	ns
85	0.37655496	109.84356	7.07E-01	0.785714286	ns	86	2.367463722	121.7192208	0.0195	0.195	ns
86	0.37570267	113.03076	7.08E-01	0.785714286	ns	87	2.253684439	119.5733264	0.026	0.216666667	ns
87	0.37922933	114.8723	7.05E-01	0.785714286	ns	88	2.131647929	117.4180525	0.0351	0.27	ns
88	0.3752179	115.50103	7.08E-01	0.785714286	ns	89	2.005457643	115.4246836	0.0473	0.315333333	ns
89	0.36577553	114.15068	7.15E-01	0.785714286	ns	90	1.869178969	114.4117768	0.0642	0.40125	ns
90	0.39005834	109.60385	6.97E-01	0.785714286	ns	91	1.70782317	114.283729	0.0904	0.502777778	ns
91	0.39946185	106.05878	6.90E-01	0.785714286	ns	92	1.560549322	114.2028284	0.121	0.636842105	ns
92	0.33676357	105.44056	7.37E-01	0.792473118	ns	93	1.420682431	113.9601262	0.158	0.651923077	ns
93	0.21901854	105.53834	8.27E-01	0.865625	ns	94	1.280071614	113.994871	0.203	0.651923077	ns
94	0.06369064	104.38568	9.49E-01	0.951	ns	95	1.151634255	114.3869156	0.252	0.651923077	ns

95	-0.12507852	102.10212	9.01E-01	0.928865979	ns	96	1.044623781	115.2440164	0.298	0.651923077	ns
96	-0.3477161	99.1943	7.29E-01	0.792391304	ns	97	0.923847086	116.6870108	0.357	0.652727273	ns
97	-0.58263655	96.38014	5.61E-01	0.70125	ns	98	0.78248909	118.4378745	0.435	0.675757576	ns
98	-0.78062104	94.43091	4.37E-01	0.590540541	ns	99	0.638875136	120.0713513	0.524	0.698666667	ns
99	-0.92872299	93.80983	3.55E-01	0.514492754	ns	100	0.49629681	121.094798	0.621	0.771604938	ns
100	-1.04099668	94.36816	3.01E-01	0.449253731	ns						
9	statistic	df	p	p.adj	p.adj.signif	10	statistic	df	p	p.adj	p.adj.signif
1	0.15528474	104.25249	0.877	0.975	ns	1	0.05514166	108.70324	0.956	0.9656566	ns
2	0.3714712	115.718	0.711	0.9285714	ns	2	0.12244743	113.22521	0.903	0.940625	ns
3	0.18056134	115.619	0.857	0.975	ns	3	0.28367338	120.52488	0.777	0.9210526	ns
4	-0.18063929	109.47079	0.857	0.975	ns	4	0.41044594	125.91258	0.682	0.9	ns
5	-0.91058258	95.75863	0.365	0.8148936	ns	5	0.36216767	127.36748	0.718	0.9	ns
6	-1.35190602	87.12009	0.18	0.6851852	ns	6	0.18761261	127.15254	0.851	0.9210526	ns
7	-1.57085339	82.17319	0.12	0.5952381	ns	7	0.21519816	125.35382	0.83	0.9210526	ns
8	-1.47162893	82.83427	0.145	0.6304348	ns	8	0.47080851	122.41804	0.639	0.9	ns
9	-1.3364491	82.37115	0.185	0.6851852	ns	9	0.75450573	120.62248	0.452	0.7409836	ns
10	-0.89620415	86.25316	0.373	0.8148936	ns	10	0.87187657	118.49501	0.385	0.6964286	ns
11	-0.63796299	90.59572	0.525	0.8597015	ns	11	0.86248364	114.97215	0.39	0.6964286	ns
12	-0.59825987	90.73897	0.551	0.8597015	ns	12	0.82093087	111.60318	0.413	0.6983333	ns
13	-0.4778618	91.57581	0.634	0.8929577	ns	13	0.72726471	108.5354	0.469	0.7564516	ns
14	-0.18590613	94.14158	0.853	0.975	ns	14	0.57443915	105.82894	0.567	0.8784615	ns
15	0.29280389	97.18294	0.77	0.9506173	ns	15	0.40150015	104.40248	0.689	0.9	ns
16	0.63103058	100.14842	0.529	0.8597015	ns	16	0.25995453	105.39394	0.795	0.9210526	ns
17	0.96258368	107.73765	0.338	0.8148936	ns	17	0.20283763	107.04478	0.84	0.9210526	ns
18	1.12525867	111.64101	0.263	0.8148936	ns	18	0.25252121	107.23034	0.801	0.9210526	ns

19	1.18888688	115.08024	0.237	0.8148936	ns	19	0.37115297	105.72187	0.711	0.9	ns
20	1.09299482	116.08836	0.277	0.8148936	ns	20	0.4547302	102.97231	0.65	0.9	ns
21	0.60703067	116.66814	0.545	0.8597015	ns	21	0.4552559	99.48366	0.65	0.9	ns
22	0.26026066	120.2747	0.795	0.9695122	ns	22	0.35897158	96.0125	0.72	0.9	ns
23	-0.18539039	117.06069	0.853	0.975	ns	23	0.17106322	94.21386	0.865	0.9210526	ns
24	-0.41749529	113.6767	0.677	0.9148649	ns	24	-0.06883228	94.46058	0.945	0.9656566	ns
25	-0.68307069	111.00764	0.496	0.8597015	ns	25	-0.27561757	97.01135	0.783	0.9210526	ns
26	-0.78484162	110.20787	0.434	0.8509804	ns	26	-0.40125373	101.90165	0.689	0.9	ns
27	-0.99037789	106.1379	0.324	0.8148936	ns	27	-0.41979662	108.76659	0.675	0.9	ns
28	-0.74116182	109.54256	0.46	0.8597015	ns	28	-0.40008011	114.68695	0.69	0.9	ns
29	-0.60452263	112.83332	0.547	0.8597015	ns	29	-0.38998348	117.57209	0.697	0.9	ns
30	-0.74172765	113.23627	0.46	0.8597015	ns	30	-0.32990158	118.42302	0.742	0.9160494	ns
31	-0.56392788	114.45785	0.574	0.8597015	ns	31	-0.15710174	118.55449	0.875	0.9210526	ns
32	-0.57609257	112.9664	0.566	0.8597015	ns	32	0.05924265	119.43158	0.953	0.9656566	ns
33	-0.59030443	115.61579	0.556	0.8597015	ns	33	0.16453527	120.66169	0.87	0.9210526	ns
34	-0.98403642	112.26084	0.327	0.8148936	ns	34	-0.18450573	120.64921	0.854	0.9210526	ns
35	-1.04741533	110.77427	0.297	0.8148936	ns	35	-0.68283718	120.00293	0.496	0.7873016	ns
36	-1.44027595	114.18873	0.153	0.6375	ns	36	-0.89864285	119.86404	0.371	0.6944444	ns
37	-1.54644851	116.5164	0.125	0.5952381	ns	37	-0.82121433	119.63337	0.413	0.6983333	ns
38	-1.74069166	118.16546	0.0843	0.4958824	ns	38	-0.84573214	115.81007	0.399	0.6983333	ns
39	-2.31024408	116.57968	0.0226	0.1738462	ns	39	-0.92474577	110.67362	0.357	0.6944444	ns
40	-2.65434827	113.1377	0.00909	0.1298571	ns	40	-0.89294148	109.56731	0.374	0.6944444	ns
41	-3.20134465	115.59475	0.00177	0.05875	ns	41	-0.89138492	108.11295	0.375	0.6944444	ns
42	-3.52001836	119.17046	0.000612	0.0306	*	42	-0.81135947	105.52462	0.419	0.6983333	ns
43	-3.55931485	122.62638	0.00053	0.0306	*	43	-0.54485421	108.41005	0.587	0.8893939	ns

44	-3.10601944	125.20548	0.00235	0.05875	ns	44	-0.27726202	114.40631	0.782	0.9210526	ns
45	-2.66620009	123.4095	0.0087	0.1298571	ns	45	0.02026737	119.54268	0.984	0.984	ns
46	-2.48370381	121.50607	0.0144	0.16	ns	46	0.51042107	123.85121	0.611	0.8985294	ns
47	-2.39727666	111.43262	0.0182	0.1666667	ns	47	1.07458508	125.0592	0.285	0.6477273	ns
48	-2.25903955	106.90426	0.0259	0.185	ns	48	1.64301321	124.91034	0.103	0.3787879	ns
49	-2.39041378	110.7073	0.0185	0.1666667	ns	49	1.98879508	123.60018	0.0489	0.3787879	ns
50	-2.512638	116.51511	0.0134	0.16	ns	50	1.94521372	119.95963	0.0541	0.3787879	ns
51	-2.70563185	113.92201	0.00787	0.1298571	ns	51	1.66326279	112.3189	0.099	0.3787879	ns
52	-2.36062798	111.04127	0.02	0.1666667	ns	52	1.35993168	105.41665	0.177	0.5057143	ns
53	-2.01723153	115.38614	0.046	0.305	ns	53	1.1957114	105.35691	0.234	0.6	ns
54	-1.99157655	116.54643	0.0488	0.305	ns	54	0.92606048	103.88696	0.357	0.6944444	ns
55	-1.55043	109.12528	0.124	0.5952381	ns	55	0.56839795	103.49385	0.571	0.8784615	ns
56	-1.63899304	102.40159	0.104	0.5777778	ns	56	0.16980794	107.45089	0.865	0.9210526	ns
57	-1.50166977	94.71958	0.137	0.6227273	ns	57	-0.19001378	113.28102	0.85	0.9210526	ns
58	-1.35093155	87.48126	0.18	0.6851852	ns	58	-0.51683816	114.25548	0.606	0.8985294	ns
59	-1.12078008	90.22548	0.265	0.8148936	ns	59	-0.90012698	109.07041	0.37	0.6944444	ns
60	-0.92462473	97.23795	0.357	0.8148936	ns	60	-1.32252684	101.45564	0.189	0.5108108	ns
61	-0.6778662	104.68111	0.499	0.8597015	ns	61	-1.62376687	99.79082	0.108	0.3787879	ns
62	-0.56099918	104.15549	0.576	0.8597015	ns	62	-1.54867477	100.87036	0.125	0.3787879	ns
63	-0.34068616	106.64607	0.734	0.9410256	ns	63	-1.34191477	101.12914	0.183	0.5083333	ns
64	-0.36547552	105.22752	0.715	0.9285714	ns	64	-1.12710287	100.08752	0.262	0.6238095	ns
65	-0.46251387	107.77156	0.645	0.8958333	ns	65	-1.04492134	99.92728	0.299	0.6644444	ns
66	-0.29593781	111.36058	0.768	0.9506173	ns	66	-1.08295665	99.57471	0.281	0.6477273	ns
67	-0.13696742	118.29221	0.891	0.975	ns	67	-1.13163651	100.1768	0.26	0.6238095	ns
68	-0.05913034	122.86527	0.953	0.9826531	ns	68	-1.02336912	104.53628	0.308	0.6695652	ns

69	0.13089757	124.16992	0.896	0.975	ns	69	-0.94328313	111.10809	0.348	0.6944444	ns
70	0.05296426	122.38288	0.958	0.9826531	ns	70	-1.00838311	114.97253	0.315	0.6702128	ns
71	0.01296886	121.01161	0.99	0.99	ns	71	-1.15696603	117.07749	0.25	0.6238095	ns
72	-0.04617819	115.85874	0.963	0.9826531	ns	72	-1.29500682	117.61213	0.198	0.5210526	ns
73	-0.29262431	109.69992	0.77	0.9506173	ns	73	-1.48157432	114.77334	0.141	0.4147059	ns
74	-0.09529884	110.35133	0.924	0.9826531	ns	74	-1.73234952	108.41443	0.0861	0.3787879	ns
75	-0.12930928	101.62943	0.897	0.975	ns	75	-1.83770369	105.05412	0.0689	0.3787879	ns
76	-0.22894186	93.64742	0.819	0.975	ns	76	-1.71369351	105.96237	0.0895	0.3787879	ns
77	-0.09373407	94.9308	0.926	0.9826531	ns	77	-1.60566417	105.54242	0.111	0.3787879	ns
78	-0.01314294	99.42148	0.99	0.99	ns	78	-1.57235916	104.39742	0.119	0.3787879	ns
79	0.1373119	112.5381	0.891	0.975	ns	79	-1.55617925	104.06397	0.123	0.3787879	ns
80	0.39635391	122.83789	0.693	0.924	ns	80	-1.55752649	103.70422	0.122	0.3787879	ns
81	0.50582991	126.82136	0.614	0.8929577	ns	81	-1.57027199	103.18422	0.119	0.3787879	ns
82	0.44635535	127.99224	0.656	0.8986301	ns	82	-1.59626424	103.6569	0.113	0.3787879	ns
83	0.58628201	127.54598	0.559	0.8597015	ns	83	-1.62868416	105.30319	0.106	0.3787879	ns
84	0.82193791	126.39893	0.413	0.8428571	ns	84	-1.69894112	106.15755	0.0923	0.3787879	ns
85	0.8507037	124.18313	0.397	0.8270833	ns	85	-1.78410334	105.79832	0.0773	0.3787879	ns
86	0.79449923	125.26963	0.428	0.8509804	ns	86	-1.83288719	104.40798	0.0697	0.3787879	ns
87	0.91507332	127.50836	0.362	0.8148936	ns	87	-1.84029427	102.77937	0.0686	0.3787879	ns
88	1.03866353	127.88864	0.301	0.8148936	ns	88	-1.79885984	101.64353	0.075	0.3787879	ns
89	1.00732138	127.69258	0.316	0.8148936	ns	89	-1.72597853	101.67878	0.0874	0.3787879	ns
90	0.91607905	126.09796	0.361	0.8148936	ns	90	-1.6869696	103.30757	0.0946	0.3787879	ns
91	0.70123084	123.59769	0.484	0.8597015	ns	91	-1.73996368	104.89265	0.0848	0.3787879	ns
92	0.47671124	118.71552	0.634	0.8929577	ns	92	-1.81836177	106.01344	0.0718	0.3787879	ns
93	0.48126152	118.43636	0.631	0.8929577	ns	93	-1.81501867	108.61603	0.0723	0.3787879	ns

94	0.73057046	123.13629	0.466	0.8597015	ns	94	-1.74267777	112.08787	0.0841	0.3787879	ns
95	0.89098809	126.88172	0.375	0.8148936	ns	95	-1.66708703	115.09944	0.0982	0.3787879	ns
96	0.93766571	124.48032	0.35	0.8148936	ns	96	-1.63972826	117.40055	0.104	0.3787879	ns
97	1.01813954	124.359	0.311	0.8148936	ns	97	-1.6883391	119.66382	0.094	0.3787879	ns
98	1.00133897	120.22387	0.319	0.8148936	ns	98	-1.87423125	121.79383	0.0633	0.3787879	ns
99	0.87551503	106.31474	0.383	0.8148936	ns	99	-2.07006471	123.58389	0.0405	0.3787879	ns
100	-0.08145633	97.03886	0.935	0.9826531	ns	100	-2.21648996	123.85254	0.0285	0.3787879	ns
11	statistic	df	p	p.adj	p.adj.signif	12	statistic	df	p	p.adj	p.adj.signif
1	2.55981115	104.9375	1.19E-02	0.0845	ns	1	0.73198135	127.13202	0.466	0.6955224	ns
2	2.45870361	109.8717	1.55E-02	0.0845	ns	2	0.86580538	127.30998	0.388	0.6772727	ns
3	2.43125531	112.835	1.66E-02	0.0845	ns	3	0.90947302	127.98763	0.365	0.6772727	ns
4	2.45377785	115.0258	1.56E-02	0.0845	ns	4	0.85798249	127.65545	0.393	0.6772727	ns
5	2.49750653	117.226	1.39E-02	0.0845	ns	5	0.80604548	127.54579	0.422	0.6772727	ns
6	2.42336377	118.325	1.69E-02	0.0845	ns	6	0.77607107	127.68555	0.439	0.6772727	ns
7	2.11381638	118.5663	3.66E-02	0.126206897	ns	7	0.87301219	127.99484	0.384	0.6772727	ns
8	1.6699451	120.9677	9.75E-02	0.25	ns	8	1.09052698	127.74001	0.278	0.6446809	ns
9	1.24904255	124.2138	2.14E-01	0.50952381	ns	9	1.03375823	127.99629	0.303	0.6446809	ns
10	0.95404499	127.2743	3.42E-01	0.697959184	ns	10	0.77459022	127.25174	0.44	0.6772727	ns
11	0.71152938	127.784	4.78E-01	0.775806452	ns	11	0.36245386	125.91091	0.718	0.8306818	ns
12	0.28782184	124.703	7.74E-01	0.914117647	ns	12	0.0581114	125.4813	0.954	0.986	ns
13	-0.30621581	120.1461	7.60E-01	0.914117647	ns	13	-0.01698795	121.94768	0.986	0.986	ns
14	-0.58567627	118.6585	5.59E-01	0.825352113	ns	14	0.03255581	113.43213	0.974	0.986	ns
15	-0.55322824	118.157	5.81E-01	0.825352113	ns	15	-0.12866549	103.67343	0.898	0.986	ns
16	-0.54546838	118.373	5.86E-01	0.825352113	ns	16	-0.40693827	100.72577	0.685	0.8190476	ns
17	-0.83270371	117.5088	4.07E-01	0.753703704	ns	17	-0.60498688	102.76956	0.547	0.7704225	ns

18	-1.30826596	117.4056	1.93E-01	0.470731707	ns	18	-0.81765671	104.33687	0.415	0.6772727	ns
19	-1.82743648	118.2385	7.02E-02	0.195	ns	19	-0.99185554	104.59996	0.324	0.648	ns
20	-2.23227605	120.4457	2.74E-02	0.1096	ns	20	-1.15248454	102.26946	0.252	0.6446809	ns
21	-2.34295889	120.7694	2.08E-02	0.094545455	ns	21	-1.40836915	99.13046	0.162	0.6433333	ns
22	-2.24163476	118.1377	2.69E-02	0.1096	ns	22	-1.75686197	98.48522	0.082	0.6433333	ns
23	-2.06077724	113.5797	4.16E-02	0.134193548	ns	23	-1.80847592	98.3995	0.0736	0.6433333	ns
24	-1.92408431	108.7523	5.70E-02	0.167647059	ns	24	-1.62942045	95.36756	0.107	0.6433333	ns
25	-1.80609868	106.7205	7.37E-02	0.195	ns	25	-1.41238099	94.23463	0.161	0.6433333	ns
26	-1.80388127	106.7426	7.41E-02	0.195	ns	26	-1.19144833	94.93129	0.236	0.6446809	ns
27	-1.86526357	110.0231	6.48E-02	0.185142857	ns	27	-1.03784351	97.61415	0.302	0.6446809	ns
28	-1.94666065	113.2452	5.41E-02	0.163939394	ns	28	-0.77238253	101.00512	0.442	0.6772727	ns
29	-2.01508722	117.7019	4.62E-02	0.144375	ns	29	-0.476248	105.44194	0.635	0.7839506	ns
30	-2.16807009	120.4692	3.21E-02	0.118888889	ns	30	-0.35313401	108.99771	0.725	0.8306818	ns
31	-2.14780916	120.0116	3.37E-02	0.120357143	ns	31	-0.42024829	110.52966	0.675	0.8190476	ns
32	-2.16942434	120.2836	3.20E-02	0.118888889	ns	32	-0.49173656	112.72119	0.624	0.7839506	ns
33	-2.31866787	123.0286	2.21E-02	0.096086957	ns	33	-0.4842593	112.85084	0.629	0.7839506	ns
34	-2.38212299	122.3387	1.88E-02	0.08952381	ns	34	-0.34401851	110.99961	0.731	0.8306818	ns
35	-2.45494996	119.7592	1.55E-02	0.0845	ns	35	-0.0517919	110.59027	0.959	0.986	ns
36	-2.6381224	119.7063	9.44E-03	0.072615385	ns	36	0.36222362	111.27559	0.718	0.8306818	ns
37	-2.85162742	121.8309	5.11E-03	0.046454545	*	37	0.76250534	114.09814	0.447	0.6772727	ns
38	-3.0449541	124.9356	2.84E-03	0.0284	*	38	1.04214603	114.02193	0.3	0.6446809	ns
39	-3.12130273	126.2547	2.23E-03	0.026444444	*	39	1.155891	111.54066	0.25	0.6446809	ns
40	-3.10230185	124.4403	2.38E-03	0.026444444	*	40	0.99167831	106.22563	0.324	0.648	ns
41	-3.12149215	120.6489	2.25E-03	0.026444444	*	41	1.05064465	105.86491	0.296	0.6446809	ns
42	-3.42528756	119.0871	8.43E-04	0.01686	*	42	1.38534868	109.8255	0.169	0.6433333	ns

43	-3.83130407	119.2471	2.05E-04	0.006833333	**	43	1.30944672	108.77397	0.193	0.6433333	ns
44	-4.21904545	117.5408	4.85E-05	0.00485	**	44	1.08700657	107.1012	0.279	0.6446809	ns
45	-4.0364694	115.0139	9.81E-05	0.004905	**	45	1.04683507	108.73	0.297	0.6446809	ns
46	-3.52487599	113.6709	6.12E-04	0.0153	*	46	1.12922978	113.00005	0.261	0.6446809	ns
47	-3.14924715	113.3696	2.09E-03	0.026444444	*	47	1.39038137	119.47324	0.167	0.6433333	ns
48	-2.6482645	111.3584	9.27E-03	0.072615385	ns	48	1.5867797	116.58294	0.115	0.6433333	ns
49	-2.06849747	107.7954	4.10E-02	0.134193548	ns	49	1.76436078	109.66224	0.0805	0.6433333	ns
50	-1.56845058	105.7403	1.20E-01	0.3	ns	50	1.9156981	101.3514	0.0582	0.6433333	ns
51	-1.08926079	103.1783	2.79E-01	0.634090909	ns	51	1.69784656	90.07282	0.093	0.6433333	ns
52	-0.84778679	102.5023	3.99E-01	0.752830189	ns	52	1.34287703	80.40092	0.183	0.6433333	ns
53	-0.72848608	103.599	4.68E-01	0.775806452	ns	53	0.80900137	72.52779	0.421	0.6772727	ns
54	-0.57351211	105.4065	5.68E-01	0.825352113	ns	54	0.50708255	68.45563	0.614	0.7839506	ns
55	-0.37568327	109.7003	7.08E-01	0.896202532	ns	55	0.61595544	69.41648	0.54	0.7704225	ns
56	-0.09673901	113.6317	9.23E-01	0.96969697	ns	56	0.83920153	72.37051	0.404	0.6772727	ns
57	0.03599389	115.8042	9.71E-01	0.971	ns	57	0.99513798	75.2856	0.323	0.648	ns
58	0.21519355	119.3429	8.30E-01	0.940449438	ns	58	1.13341454	75.66114	0.261	0.6446809	ns
59	0.45629265	121.0616	6.49E-01	0.877027027	ns	59	1.22447015	74.8748	0.225	0.6446809	ns
60	0.75603127	121.766	4.51E-01	0.775806452	ns	60	1.31234186	75.56838	0.193	0.6433333	ns
61	0.90097113	122.509	3.69E-01	0.709615385	ns	61	1.43955008	80.01562	0.154	0.6433333	ns
62	0.75084708	121.8615	4.54E-01	0.775806452	ns	62	1.49082938	84.67176	0.14	0.6433333	ns
63	0.40791231	121.5943	6.84E-01	0.896202532	ns	63	1.40433256	86.20752	0.164	0.6433333	ns
64	-0.0588541	123.5802	9.53E-01	0.96969697	ns	64	1.05503366	83.47875	0.294	0.6446809	ns
65	-0.33896262	124.5148	7.35E-01	0.911111111	ns	65	0.84376325	80.83858	0.401	0.6772727	ns
66	-0.17589096	124.0826	8.61E-01	0.956666667	ns	66	1.07338015	83.30479	0.286	0.6446809	ns
67	0.04985139	123.322	9.60E-01	0.96969697	ns	67	1.52891877	90.45497	0.13	0.6433333	ns

68	0.20579736	118.1008	8.37E-01	0.940449438	ns	68	1.79659755	96.94264	0.0755	0.6433333	ns
69	0.12021846	114.389	9.05E-01	0.96969697	ns	69	1.63965307	97.66674	0.104	0.6433333	ns
70	-0.23468604	113.7728	8.15E-01	0.936781609	ns	70	1.51723579	92.69569	0.133	0.6433333	ns
71	-0.37481187	115.3385	7.08E-01	0.896202532	ns	71	1.37770852	83.8565	0.172	0.6433333	ns
72	-0.07589123	114.1803	9.40E-01	0.96969697	ns	72	1.21066817	82.39548	0.229	0.6446809	ns
73	0.5648526	115.7941	5.73E-01	0.825352113	ns	73	0.97711251	82.91621	0.331	0.6490196	ns
74	0.98852272	118.4742	3.25E-01	0.697959184	ns	74	0.80450422	81.04425	0.423	0.6772727	ns
75	0.70643981	111.2895	4.81E-01	0.775806452	ns	75	0.55422122	76.49318	0.581	0.7839506	ns
76	0.62873908	104.1451	5.31E-01	0.806060606	ns	76	0.58470049	70.87364	0.561	0.7791667	ns
77	0.6363162	100.8375	5.26E-01	0.806060606	ns	77	0.62323622	67.68435	0.535	0.7704225	ns
78	0.79254406	104.308	4.30E-01	0.775806452	ns	78	0.40325249	64.613	0.688	0.8190476	ns
79	0.9613041	111.9076	3.38E-01	0.697959184	ns	79	0.08653225	62.43427	0.931	0.986	ns
80	0.74483347	119.1297	4.58E-01	0.775806452	ns	80	-0.06005	62.63838	0.952	0.986	ns
81	0.62750262	120.873	5.32E-01	0.806060606	ns	81	-0.0326054	65.44886	0.974	0.986	ns
82	0.91515383	119.2412	3.62E-01	0.709615385	ns	82	-0.02736718	66.10615	0.978	0.986	ns
83	1.0066387	116.3556	3.16E-01	0.697959184	ns	83	-0.0306213	67.61409	0.976	0.986	ns
84	0.91096651	114.628	3.64E-01	0.709615385	ns	84	-0.1098786	71.68155	0.913	0.986	ns
85	0.68945073	115.5279	4.92E-01	0.780952381	ns	85	-0.26939188	78.33879	0.788	0.8853933	ns
86	0.28336487	118.6681	7.77E-01	0.914117647	ns	86	-0.47610152	83.18654	0.635	0.7839506	ns
87	-0.1300663	118.511	8.97E-01	0.96969697	ns	87	-0.52538079	90.59697	0.601	0.7839506	ns
88	-0.51517668	114.701	6.07E-01	0.843055556	ns	88	-0.56346951	98.91235	0.574	0.7839506	ns
89	-0.95533566	110.8199	3.41E-01	0.697959184	ns	89	-0.52039523	107.29283	0.604	0.7839506	ns
90	-1.09863917	111.8841	2.74E-01	0.634090909	ns	90	-0.6304999	102.95909	0.53	0.7704225	ns
91	-0.71073402	116.7585	4.79E-01	0.775806452	ns	91	-1.16070145	97.70399	0.249	0.6446809	ns
92	-0.25521215	117.855	7.99E-01	0.929069767	ns	92	-1.81537481	92.94777	0.0727	0.6433333	ns

93	-0.07334756	114.783	9.42E-01	0.96969697	ns	93	-2.21396964	88.9859	0.0294	0.6433333	ns
94	0.05742665	114.4698	9.54E-01	0.96969697	ns	94	-2.52397619	83.2724	0.0135	0.6433333	ns
95	0.2998326	118.1211	7.65E-01	0.914117647	ns	95	-2.47750796	81.57629	0.0153	0.6433333	ns
96	0.40754743	121.4369	6.84E-01	0.896202532	ns	96	-2.14594846	87.2333	0.0347	0.6433333	ns
97	0.49204046	122.0798	6.24E-01	0.854794521	ns	97	-1.90854179	95.81973	0.0593	0.6433333	ns
98	0.38428469	122.6505	7.01E-01	0.896202532	ns	98	-1.700996	105.53227	0.0919	0.6433333	ns
99	-0.1041656	121.5227	9.17E-01	0.96969697	ns	99	-1.34528464	111.14615	0.181	0.6433333	ns
100	-0.33536076	119.0752	7.38E-01	0.911111111	ns	100	-0.93131703	113.34123	0.354	0.6772727	ns
13	statistic	df	p	p.adj	p.adj.signif	14	statistic	df	p	p.adj	p.adj.signif
1	-2.471792798	77.70017	1.56E-02	0.028363636	*	1	-1.33856404	98.30433	0.184	0.4173913	ns
2	-2.633907916	78.93607	1.02E-02	0.019615385	*	2	-1.42069883	97.0214	0.159	0.4170732	ns
3	-2.86319185	81.32142	5.33E-03	0.011844444	*	3	-1.46951701	95.65871	0.145	0.4054054	ns
4	-3.0767022	83.40235	2.83E-03	0.006738095	**	4	-1.4858401	93.95595	0.141	0.4054054	ns
5	-3.266009577	84.91788	1.57E-03	0.004361111	**	5	-1.50699436	92.3411	0.135	0.4054054	ns
6	-3.419949243	86.10198	9.59E-04	0.002820588	**	6	-1.57372148	91.70159	0.119	0.4054054	ns
7	-3.543982979	86.82502	6.37E-04	0.002054839	**	7	-1.62743045	91.92328	0.107	0.4054054	ns
8	-3.619524977	87.39051	4.94E-04	0.001646667	**	8	-1.61069961	92.0448	0.111	0.4054054	ns
9	-3.63692241	88.12658	4.64E-04	0.0016	**	9	-1.57196842	91.99276	0.119	0.4054054	ns
10	-3.641247584	89.91886	4.53E-04	0.0016	**	10	-1.51533042	91.71411	0.133	0.4054054	ns
11	-3.649920698	92.9854	4.33E-04	0.0016	**	11	-1.46118729	91.58422	0.147	0.4054054	ns
12	-3.691594689	96.69023	3.69E-04	0.0015625	**	12	-1.39959571	93.13835	0.165	0.4170732	ns
13	-3.72162105	100.70537	3.26E-04	0.001509091	**	13	-1.32523618	97.22634	0.188	0.4173913	ns
14	-3.710887594	104.93635	3.32E-04	0.001509091	**	14	-1.26974135	103.17485	0.207	0.4354167	ns
15	-3.63670252	108.12288	4.25E-04	0.0016	**	15	-1.22118918	108.88302	0.225	0.45	ns
16	-3.504933638	109.82018	6.62E-04	0.00206875	**	16	-1.16578191	113.75607	0.246	0.4696429	ns

17	-3.360647388	110.77971	1.07E-03	0.003057143	**	17	-1.12796735	116.76521	0.262	0.4696429	ns
18	-3.226379553	110.89337	1.65E-03	0.004368421	**	18	-1.0959226	118.05247	0.275	0.4741379	ns
19	-3.117410153	110.29514	2.33E-03	0.005825	**	19	-1.04090816	118.44988	0.3	0.5	ns
20	-3.037022971	110.10961	2.98E-03	0.006930233	**	20	-0.97152683	118.0851	0.333	0.5203125	ns
21	-3.016342707	111.92657	3.17E-03	0.007204546	**	21	-0.89925047	117.0987	0.37	0.5594203	ns
22	-3.064414301	114.6896	2.72E-03	0.006634146	**	22	-0.8208629	116.04173	0.413	0.5887324	ns
23	-3.218988832	117.40309	1.66E-03	0.004368421	**	23	-0.73267853	115.29999	0.465	0.60625	ns
24	-3.440268572	119.76562	8.01E-04	0.002427273	**	24	-0.63402084	115.50568	0.527	0.6127907	ns
25	-3.636022512	121.09519	4.08E-04	0.0016	**	25	-0.51215491	116.67411	0.61	0.6853933	ns
26	-3.843525641	122.65907	1.94E-04	0.00097	***	26	-0.36193849	117.02626	0.718	0.7804348	ns
27	-4.105643636	124.72938	7.24E-05	0.000402222	***	27	-0.1813142	116.0205	0.856	0.8916667	ns
28	-4.329694122	125.53227	3.02E-05	0.000215714	***	28	0.04292105	114.28588	0.966	0.966	ns
29	-4.490939448	125.57686	1.59E-05	0.000176667	***	29	0.28847915	113.68908	0.774	0.8322581	ns
30	-4.562911488	125.06754	1.19E-05	0.00014875	***	30	0.51521991	114.07723	0.607	0.6853933	ns
31	-4.635215989	124.3989	8.87E-06	0.000147833	***	31	0.6650741	113.42944	0.507	0.6119048	ns
32	-4.715756156	124.20822	6.36E-06	0.0001272	***	32	0.7155303	111.7909	0.476	0.60625	ns
33	-4.807363055	124.66315	4.32E-06	0.0001272	***	33	0.68737814	110.50205	0.493	0.608642	ns
34	-4.891751807	124.99021	3.01E-06	0.0001272	***	34	0.66226644	110.28124	0.509	0.6119048	ns
35	-4.884477384	125.44654	3.10E-06	0.0001272	***	35	0.65546496	110.56253	0.514	0.6119048	ns
36	-4.755020002	126.15251	5.33E-06	0.0001272	***	36	0.70120772	110.30961	0.485	0.60625	ns
37	-4.577437579	126.81291	1.11E-05	0.00014875	***	37	0.77551968	110.24026	0.44	0.60625	ns
38	-4.412213347	127.5016	2.15E-05	0.000215	***	38	0.86971077	110.69254	0.386	0.5594203	ns
39	-4.356321297	127.88978	2.69E-05	0.000215714	***	39	1.00159504	111.60407	0.319	0.5063492	ns
40	-4.35086168	127.99833	2.75E-05	0.000215714	***	40	1.14613861	113.03733	0.254	0.4696429	ns
41	-4.333319071	127.73494	2.95E-05	0.000215714	***	41	1.35291212	115.4014	0.179	0.4173913	ns

42	-4.305033513	127.3721	3.30E-05	0.00022	***	42	1.53753364	118.03431	0.127	0.4054054	ns
43	-4.210186767	127.11121	4.79E-05	0.000299375	***	43	1.66930109	119.8987	0.0977	0.4054054	ns
44	-4.120738112	126.88785	6.77E-05	0.000398235	***	44	1.76277485	120.50012	0.0805	0.4054054	ns
45	-3.977760134	126.58584	1.16E-04	0.000610526	***	45	1.67413017	120.3207	0.0967	0.4054054	ns
46	-3.654931306	127.176	3.75E-04	0.0015625	**	46	1.46770609	118.85606	0.145	0.4054054	ns
47	-3.18847195	127.87621	1.80E-03	0.004615385	**	47	1.26349881	117.22056	0.209	0.4354167	ns
48	-2.61649788	122.79723	1.00E-02	0.019607843	*	48	1.0000436	115.52582	0.319	0.5063492	ns
49	-2.104798632	113.01833	3.75E-02	0.060483871	ns	49	0.70159907	112.64743	0.484	0.60625	ns
50	-1.8930953	109.95233	6.10E-02	0.087142857	ns	50	0.44922063	107.39103	0.654	0.7266667	ns
51	-1.758444251	109.74052	8.15E-02	0.108666667	ns	51	0.25410188	102.01589	0.8	0.8510638	ns
52	-1.661462076	110.63617	9.95E-02	0.127564103	ns	52	0.14539896	98.55568	0.885	0.9123711	ns
53	-1.672675076	112.99361	9.72E-02	0.126233766	ns	53	0.10004166	96.27549	0.921	0.930303	ns
54	-1.775385414	115.77642	7.85E-02	0.106081081	ns	54	0.12387589	95.29167	0.902	0.9204082	ns
55	-1.874864216	117.37785	6.33E-02	0.08915493	ns	55	0.22263086	95.93379	0.824	0.8673684	ns
56	-1.981885105	117.97134	4.98E-02	0.0778125	ns	56	0.36785675	98.49663	0.714	0.7804348	ns
57	-2.145770475	118.34404	3.39E-02	0.0565	ns	57	0.51233269	102.15934	0.61	0.6853933	ns
58	-2.33100144	119.4053	2.14E-02	0.03754386	*	58	0.64022559	103.7739	0.523	0.6127907	ns
59	-2.478199846	120.55696	1.46E-02	0.027037037	*	59	0.73464342	102.03189	0.464	0.60625	ns
60	-2.620629816	121.01547	9.90E-03	0.019607843	*	60	0.81399813	98.1458	0.418	0.5887324	ns
61	-2.733930052	120.90727	7.20E-03	0.015	*	61	0.93838687	95.68082	0.35	0.5384615	ns
62	-2.800645917	121.67869	5.93E-03	0.012617021	*	62	1.04625224	93.84576	0.298	0.5	ns
63	-2.807078485	122.66014	5.82E-03	0.012617021	*	63	1.12669209	93.19444	0.263	0.4696429	ns
64	-2.689325523	122.73114	8.16E-03	0.016653061	*	64	1.15417396	94.16409	0.251	0.4696429	ns
65	-2.529023244	121.58365	1.27E-02	0.023962264	*	65	1.22981685	95.60992	0.222	0.45	ns
66	-2.396073925	119.72514	1.81E-02	0.032321429	*	66	1.37939964	96.74647	0.171	0.4170732	ns

67	-2.315395071	117.55225	2.23E-02	0.038448276	*	67	1.4983979	97.7638	0.137	0.4054054	ns
68	-2.251941245	115.18395	2.62E-02	0.04440678	*	68	1.59514282	98.69138	0.114	0.4054054	ns
69	-2.138020089	113.09368	3.47E-02	0.056885246	ns	69	1.65455454	99.9861	0.101	0.4054054	ns
70	-1.998454034	112.16043	4.81E-02	0.076349206	ns	70	1.60405506	101.91364	0.112	0.4054054	ns
71	-1.932099882	112.51274	5.59E-02	0.082205882	ns	71	1.50566271	104.10773	0.135	0.4054054	ns
72	-1.947978028	113.79122	5.39E-02	0.080447761	ns	72	1.37855212	105.77621	0.171	0.4170732	ns
73	-1.973846248	115.80933	5.08E-02	0.078153846	ns	73	1.19857872	106.61661	0.233	0.4568627	ns
74	-1.962305831	117.69169	5.21E-02	0.078939394	ns	74	1.02246503	107.27449	0.309	0.5063492	ns
75	-1.895082287	118.51006	6.05E-02	0.087142857	ns	75	0.87994791	107.80998	0.381	0.5594203	ns
76	-1.818632368	118.06601	7.15E-02	0.099305556	ns	76	0.76605452	108.31417	0.445	0.60625	ns
77	-1.7963135	118.18645	7.50E-02	0.102739726	ns	77	0.70136474	108.57335	0.485	0.60625	ns
78	-1.722659111	117.80089	8.76E-02	0.115263158	ns	78	0.74450005	107.93796	0.458	0.60625	ns
79	-1.561613116	116.83631	1.21E-01	0.153164557	ns	79	0.89092101	106.88404	0.375	0.5594203	ns
80	-1.365500726	116.19944	1.75E-01	0.21875	ns	80	1.10787647	105.65946	0.27	0.4736842	ns
81	-1.10174186	116.43701	2.73E-01	0.337037037	ns	81	1.31423076	105.44801	0.192	0.4173913	ns
82	-0.834594842	117.59371	4.06E-01	0.495121951	ns	82	1.44920063	105.99738	0.15	0.4054054	ns
83	-0.606315529	119.28742	5.45E-01	0.648809524	ns	83	1.53954413	106.9414	0.127	0.4054054	ns
84	-0.357206533	120.86248	7.22E-01	0.802222222	ns	84	1.61084917	107.11664	0.11	0.4054054	ns
85	-0.095135647	121.94154	9.24E-01	0.959793814	ns	85	1.68285878	105.12062	0.0954	0.4054054	ns
86	0.144913902	122.605	8.85E-01	0.931578947	ns	86	1.73252806	102.00523	0.0862	0.4054054	ns
87	0.320567769	122.50545	7.49E-01	0.814130435	ns	87	1.6971381	98.28721	0.0928	0.4054054	ns
88	0.470823487	122.07647	6.39E-01	0.726136364	ns	88	1.62205259	95.00352	0.108	0.4054054	ns
89	0.595046765	122.31481	5.53E-01	0.650588235	ns	89	1.53394805	93.23631	0.128	0.4054054	ns
90	0.628288712	123.16318	5.31E-01	0.639759036	ns	90	1.48187208	92.74413	0.142	0.4054054	ns
91	0.582520383	124.03686	5.61E-01	0.652325581	ns	91	1.53671456	93.25609	0.128	0.4054054	ns

92	0.496783987	124.13544	6.20E-01	0.712643678	ns	92	1.67835684	94.37941	0.0966	0.4054054	ns
93	0.402184102	122.97678	6.88E-01	0.773033708	ns	93	1.80784359	95.53833	0.0738	0.4054054	ns
94	0.269338895	120.82137	7.88E-01	0.847311828	ns	94	1.85470847	96.5551	0.0667	0.4054054	ns
95	0.168770068	118.5099	8.66E-01	0.921276596	ns	95	1.85437453	97.15381	0.0667	0.4054054	ns
96	0.086410838	116.73172	9.31E-01	0.959793814	ns	96	1.82648402	96.79174	0.0709	0.4054054	ns
97	0.032347798	115.06035	9.74E-01	0.983838384	ns	97	1.76435677	95.96651	0.0809	0.4054054	ns
98	-0.000579778	113.19046	1.00E+00	1	ns	98	1.63209643	94.66733	0.106	0.4054054	ns
99	0.068145099	109.28519	9.46E-01	0.965306122	ns	99	1.49242431	93.34298	0.139	0.4054054	ns
100	0.324262814	99.91839	7.46E-01	0.814130435	ns	100	1.35136861	91.71316	0.18	0.4173913	ns
15	statistic	df	p	p.adj	p.adj.signif	16	statistic	df	p	p.adj	p.adj.signif
1	-2.77702452	126.1685	0.00632	0.08571429	ns	1	1.24311034	76.90076	0.218	0.7397849	ns
2	-2.78396006	126.1111	0.0062	0.08571429	ns	2	1.21507333	76.73809	0.228	0.7397849	ns
3	-2.78415875	126.2185	0.00619	0.08571429	ns	3	1.17736152	76.68191	0.243	0.7397849	ns
4	-2.77032049	126.4893	0.00644	0.08571429	ns	4	1.12782487	76.7043	0.263	0.7397849	ns
5	-2.74543255	126.7081	0.00692	0.08571429	ns	5	1.06939176	76.79937	0.288	0.7397849	ns
6	-2.72040825	126.8719	0.00744	0.08571429	ns	6	1.00686395	76.97084	0.317	0.7397849	ns
7	-2.69591531	127.0006	0.00797	0.08571429	ns	7	0.94425306	77.18279	0.348	0.7397849	ns
8	-2.67263149	127.0944	0.00851	0.08571429	ns	8	0.88643178	77.37795	0.378	0.7397849	ns
9	-2.64757651	127.1627	0.00913	0.08571429	ns	9	0.8391922	77.52014	0.404	0.7397849	ns
10	-2.62018094	127.203	0.00986	0.08571429	ns	10	0.80076627	77.62033	0.426	0.7397849	ns
11	-2.59800383	127.2027	0.0105	0.08571429	ns	11	0.76828023	77.69442	0.445	0.7397849	ns
12	-2.58283765	127.1525	0.0109	0.08571429	ns	12	0.74280442	77.76376	0.46	0.7397849	ns
13	-2.56954974	127.0559	0.0113	0.08571429	ns	13	0.72326862	77.82212	0.472	0.7397849	ns
14	-2.54807334	126.9372	0.012	0.08571429	ns	14	0.70996521	77.93255	0.48	0.7397849	ns
15	-2.51980512	126.7991	0.013	0.08666667	ns	15	0.70300876	78.18128	0.484	0.7397849	ns

16	-2.49024351	126.6341	0.0141	0.088125	ns	16	0.70354204	78.58427	0.484	0.7397849	ns
17	-2.45831507	126.4079	0.0153	0.09	ns	17	0.71474803	79.20732	0.477	0.7397849	ns
18	-2.41851287	126.0676	0.017	0.09444444	ns	18	0.73080531	80.00015	0.467	0.7397849	ns
19	-2.37228728	125.6476	0.0192	0.10105263	ns	19	0.74411318	80.87684	0.459	0.7397849	ns
20	-2.32422922	125.1432	0.0217	0.1085	ns	20	0.75083053	81.79148	0.455	0.7397849	ns
21	-2.27609757	124.4722	0.0245	0.11666667	ns	21	0.74872296	82.6922	0.456	0.7397849	ns
22	-2.23024594	123.6644	0.0275	0.125	ns	22	0.74169205	83.51275	0.46	0.7397849	ns
23	-2.18374307	122.809	0.0309	0.13434783	ns	23	0.7348228	84.2283	0.464	0.7397849	ns
24	-2.13641811	121.984	0.0346	0.14416667	ns	24	0.72518159	84.80803	0.47	0.7397849	ns
25	-2.08066459	121.2121	0.0396	0.1584	ns	25	0.70952205	85.2319	0.48	0.7397849	ns
26	-2.01856454	120.5164	0.0458	0.17615385	ns	26	0.68692742	85.56584	0.494	0.7397849	ns
27	-1.95588527	119.955	0.0528	0.19555556	ns	27	0.65837555	85.85076	0.512	0.7397849	ns
28	-1.89553573	119.6045	0.0604	0.21571429	ns	28	0.63317137	86.14878	0.528	0.7397849	ns
29	-1.83906409	119.5041	0.0684	0.23586207	ns	29	0.61532933	86.49371	0.54	0.7397849	ns
30	-1.78147237	119.6768	0.0774	0.258	ns	30	0.60720724	86.92032	0.545	0.7397849	ns
31	-1.71927224	120.0748	0.0881	0.28419355	ns	31	0.60470487	87.42142	0.547	0.7397849	ns
32	-1.65166593	120.5867	0.101	0.315625	ns	32	0.59809564	87.98459	0.551	0.7397849	ns
33	-1.5761676	121.0559	0.118	0.35757576	ns	33	0.58004558	88.59681	0.563	0.7397849	ns
34	-1.49601789	121.435	0.137	0.40294118	ns	34	0.55288678	89.24028	0.582	0.7397849	ns
35	-1.41429249	121.7463	0.16	0.45714286	ns	35	0.51730565	89.91345	0.606	0.7397849	ns
36	-1.33233367	122.0009	0.185	0.51388889	ns	36	0.47967239	90.64719	0.633	0.7397849	ns
37	-1.25194013	122.1823	0.213	0.57567568	ns	37	0.44956407	91.55897	0.654	0.7397849	ns
38	-1.17705479	122.3054	0.241	0.63421053	ns	38	0.42718419	92.68686	0.67	0.7397849	ns
39	-1.10981504	122.4784	0.269	0.68974359	ns	39	0.41130725	94.07092	0.682	0.7397849	ns
40	-1.04704908	122.6166	0.297	0.7425	ns	40	0.40321746	95.66593	0.688	0.7397849	ns

41	-0.98980302	122.7957	0.324	0.7902439	ns	41	0.4028575	97.42091	0.688	0.7397849	ns
42	-0.94032289	123.0166	0.349	0.83095238	ns	42	0.41106551	99.23918	0.682	0.7397849	ns
43	-0.89648774	123.1723	0.372	0.86511628	ns	43	0.42803979	101.02823	0.67	0.7397849	ns
44	-0.85399916	123.1881	0.395	0.89772727	ns	44	0.45162793	102.75504	0.652	0.7397849	ns
45	-0.81189737	123.1119	0.418	0.92888889	ns	45	0.4757991	104.38867	0.635	0.7397849	ns
46	-0.77261924	122.9728	0.441	0.95869565	ns	46	0.49876145	105.94648	0.619	0.7397849	ns
47	-0.7396518	122.7354	0.461	0.98085106	ns	47	0.52468976	107.48662	0.601	0.7397849	ns
48	-0.70766207	122.3991	0.481	0.989	ns	48	0.55387475	109.08179	0.581	0.7397849	ns
49	-0.66861924	121.9258	0.505	0.989	ns	49	0.58406126	110.74841	0.56	0.7397849	ns
50	-0.61926708	121.2489	0.537	0.989	ns	50	0.61345693	112.5987	0.541	0.7397849	ns
51	-0.56488004	120.3714	0.573	0.989	ns	51	0.63765346	114.69939	0.525	0.7397849	ns
52	-0.5103262	119.4125	0.611	0.989	ns	52	0.65528429	117.0009	0.514	0.7397849	ns
53	-0.4609682	118.4312	0.646	0.989	ns	53	0.66515265	119.32458	0.507	0.7397849	ns
54	-0.41562315	117.4975	0.678	0.989	ns	54	0.67152984	121.54236	0.503	0.7397849	ns
55	-0.36864171	116.5561	0.713	0.989	ns	55	0.67401421	123.53499	0.502	0.7397849	ns
56	-0.31928184	115.7101	0.75	0.989	ns	56	0.66730476	125.16801	0.506	0.7397849	ns
57	-0.27043774	115.1017	0.787	0.989	ns	57	0.6471944	126.4039	0.519	0.7397849	ns
58	-0.22860001	114.8273	0.82	0.989	ns	58	0.61132961	127.22419	0.542	0.7397849	ns
59	-0.19737646	114.9591	0.844	0.989	ns	59	0.55700519	127.69053	0.578	0.7397849	ns
60	-0.16795611	115.2949	0.867	0.989	ns	60	0.47913885	127.90838	0.633	0.7397849	ns
61	-0.13856274	115.7411	0.89	0.989	ns	61	0.38102493	127.98435	0.704	0.7489362	ns
62	-0.10691351	116.2578	0.915	0.989	ns	62	0.27062226	127.99933	0.787	0.8197917	ns
63	-0.07516753	116.8348	0.94	0.989	ns	63	0.15160875	127.9995	0.88	0.8979592	ns
64	-0.04796462	117.5398	0.962	0.989	ns	64	0.02792029	127.98646	0.978	0.978	ns
65	-0.02806304	118.3678	0.978	0.989	ns	65	-0.10114495	127.90804	0.92	0.9292929	ns

66	-0.01710124	119.2367	0.986	0.989	ns	66	-0.23214272	127.68393	0.817	0.842268	ns
67	-0.01357548	120.1474	0.989	0.989	ns	67	-0.36356062	127.24369	0.717	0.7547368	ns
68	-0.01802865	121.1276	0.986	0.989	ns	68	-0.49079381	126.56173	0.624	0.7397849	ns
69	-0.03232727	122.1808	0.974	0.989	ns	69	-0.60759868	125.6414	0.545	0.7397849	ns
70	-0.05407588	123.2531	0.957	0.989	ns	70	-0.7139619	124.54688	0.477	0.7397849	ns
71	-0.08193119	124.2385	0.935	0.989	ns	71	-0.81542879	123.32764	0.416	0.7397849	ns
72	-0.11339566	125.067	0.91	0.989	ns	72	-0.91339675	122.04217	0.363	0.7397849	ns
73	-0.14108427	125.6994	0.888	0.989	ns	73	-1.01006563	120.73322	0.314	0.7397849	ns
74	-0.16501544	126.1492	0.869	0.989	ns	74	-1.10723087	119.48367	0.27	0.7397849	ns
75	-0.18906191	126.4535	0.85	0.989	ns	75	-1.20465807	118.39642	0.231	0.7397849	ns
76	-0.21422349	126.6494	0.831	0.989	ns	76	-1.29897661	117.48681	0.196	0.7397849	ns
77	-0.23880965	126.7517	0.812	0.989	ns	77	-1.38680524	116.77759	0.168	0.7397849	ns
78	-0.25960381	126.7728	0.796	0.989	ns	78	-1.46662293	116.25663	0.145	0.7397849	ns
79	-0.27412313	126.7397	0.784	0.989	ns	79	-1.5360088	115.91819	0.127	0.7397849	ns
80	-0.28077451	126.6911	0.779	0.989	ns	80	-1.59289866	115.72603	0.114	0.7397849	ns
81	-0.28225941	126.6463	0.778	0.989	ns	81	-1.63825312	115.57844	0.104	0.7397849	ns
82	-0.27787774	126.5944	0.782	0.989	ns	82	-1.67390995	115.38566	0.0969	0.7397849	ns
83	-0.26730814	126.5183	0.79	0.989	ns	83	-1.69526785	115.09341	0.0927	0.7397849	ns
84	-0.2518957	126.4296	0.802	0.989	ns	84	-1.69481588	114.71159	0.0928	0.7397849	ns
85	-0.23294914	126.3552	0.816	0.989	ns	85	-1.67443143	114.24235	0.0968	0.7397849	ns
86	-0.21336617	126.3157	0.831	0.989	ns	86	-1.63698672	113.80755	0.104	0.7397849	ns
87	-0.20026654	126.3011	0.842	0.989	ns	87	-1.59236069	113.51336	0.114	0.7397849	ns
88	-0.19620232	126.2804	0.845	0.989	ns	88	-1.54762001	113.46324	0.124	0.7397849	ns
89	-0.1998554	126.2571	0.842	0.989	ns	89	-1.50701469	113.64531	0.135	0.7397849	ns
90	-0.20825365	126.2313	0.835	0.989	ns	90	-1.46321397	114.01753	0.146	0.7397849	ns

91	-0.22225157	126.2178	0.824	0.989	ns	91	-1.41124935	114.50186	0.161	0.7397849	ns
92	-0.24012773	126.2296	0.811	0.989	ns	92	-1.35937443	115.08436	0.177	0.7397849	ns
93	-0.25806541	126.2872	0.797	0.989	ns	93	-1.32566477	115.78578	0.188	0.7397849	ns
94	-0.26972011	126.3937	0.788	0.989	ns	94	-1.31838102	116.47813	0.19	0.7397849	ns
95	-0.27597632	126.5433	0.783	0.989	ns	95	-1.33533194	117.01237	0.184	0.7397849	ns
96	-0.2742673	126.746	0.784	0.989	ns	96	-1.36503704	117.34698	0.175	0.7397849	ns
97	-0.25629899	126.9658	0.798	0.989	ns	97	-1.38990225	117.53725	0.167	0.7397849	ns
98	-0.21331197	127.1789	0.831	0.989	ns	98	-1.39519767	117.69027	0.166	0.7397849	ns
99	-0.14426533	127.3763	0.886	0.989	ns	99	-1.3776289	117.93956	0.171	0.7397849	ns
100	-0.05803906	127.547	0.954	0.989	ns	100	-1.34404817	118.4024	0.182	0.7397849	ns
17	statistic	df	p	p.adj	p.adj.signif	18	statistic	df	p	p.adj	p.adj.signif
1	1.109038635	81.15187	0.271	0.5211538	ns	1	1.4833149	103.6109	0.141	0.405	ns
2	1.029907394	84.42754	0.306	0.5563636	ns	2	1.4328757	104.0829	0.155	0.405	ns
3	0.951954019	89.66228	0.344	0.6051724	ns	3	1.3918048	104.32468	0.167	0.405	ns
4	0.869738977	96.71527	0.387	0.6344262	ns	4	1.3695082	104.33095	0.174	0.405	ns
5	0.781330084	104.24343	0.436	0.68125	ns	5	1.3498671	104.03399	0.18	0.405	ns
6	0.697632207	109.33436	0.487	0.7358209	ns	6	1.3356009	103.55285	0.185	0.405	ns
7	0.613038423	111.36985	0.541	0.784058	ns	7	1.3356068	102.87198	0.185	0.405	ns
8	0.554901936	111.12729	0.58	0.8055556	ns	8	1.3457579	101.8227	0.181	0.405	ns
9	0.508509393	110.72904	0.612	0.8092105	ns	9	1.3563813	100.48609	0.178	0.405	ns
10	0.454058952	110.97305	0.651	0.81375	ns	10	1.3575078	98.96814	0.178	0.405	ns
11	0.380538825	111.2153	0.704	0.8464286	ns	11	1.350507	97.37283	0.18	0.405	ns
12	0.296842814	111.4844	0.767	0.8715909	ns	12	1.3314646	95.74464	0.186	0.405	ns
13	0.204100165	111.49204	0.839	0.9119565	ns	13	1.2958431	94.13211	0.198	0.405	ns
14	0.102773358	111.35251	0.918	0.95625	ns	14	1.2455859	92.54875	0.216	0.405	ns

15	0.009439983	111.04112	0.992	0.992	ns	15	1.1846076	91.05605	0.239	0.405	ns
16	-0.068906077	110.4848	0.945	0.9642857	ns	16	1.1168118	89.81482	0.267	0.4171875	ns
17	-0.141085289	109.65266	0.888	0.9431579	ns	17	1.0482036	88.99352	0.297	0.43239437	ns
18	-0.205739092	109.00335	0.837	0.9119565	ns	18	0.9834498	88.61912	0.328	0.44931507	ns
19	-0.273320067	108.4669	0.785	0.8820225	ns	19	0.9289016	88.70192	0.355	0.46363636	ns
20	-0.357161182	107.75965	0.722	0.8494118	ns	20	0.8809497	89.14978	0.381	0.47160494	ns
21	-0.458447409	106.74826	0.648	0.81375	ns	21	0.8355199	89.85217	0.406	0.47291667	ns
22	-0.568441247	105.56927	0.571	0.8042254	ns	22	0.7981661	90.67941	0.427	0.47291667	ns
23	-0.687957155	104.2543	0.493	0.7358209	ns	23	0.7720896	91.44631	0.442	0.47291667	ns
24	-0.810896187	102.93253	0.419	0.6650794	ns	24	0.756573	91.94128	0.451	0.47291667	ns
25	-0.937166567	101.5054	0.351	0.6051724	ns	25	0.7519037	92.09477	0.454	0.47291667	ns
26	-1.056218595	100.2664	0.293	0.5528302	ns	26	0.7554675	91.91652	0.452	0.47291667	ns
27	-1.161023349	99.07865	0.248	0.496	ns	27	0.7586592	91.48215	0.45	0.47291667	ns
28	-1.250363525	97.68401	0.214	0.4652174	ns	28	0.7610358	90.91492	0.449	0.47291667	ns
29	-1.327907564	96.51811	0.187	0.425	ns	29	0.762474	90.30033	0.448	0.47291667	ns
30	-1.405046434	95.72901	0.163	0.3790698	ns	30	0.7642064	89.55309	0.447	0.47291667	ns
31	-1.481823882	95.28756	0.142	0.355	ns	31	0.7734971	88.68332	0.441	0.47291667	ns
32	-1.556392064	95.13576	0.123	0.3324324	ns	32	0.7995534	87.68909	0.426	0.47291667	ns
33	-1.630647265	95.70801	0.106	0.2944444	ns	33	0.8481252	86.5589	0.399	0.47291667	ns
34	-1.716152078	96.73829	0.0893	0.2706061	ns	34	0.9072034	85.35817	0.367	0.47051282	ns
35	-1.818670718	98.06475	0.072	0.24	ns	35	0.9687172	84.09659	0.335	0.4527027	ns
36	-1.9299904	99.39273	0.0565	0.2017857	ns	36	1.0288675	82.83124	0.307	0.43239437	ns
37	-2.036352708	100.41228	0.0443	0.1780769	ns	37	1.0862703	81.69455	0.281	0.42575758	ns
38	-2.143445165	100.776	0.0345	0.1568182	ns	38	1.1361461	80.82055	0.259	0.41269841	ns
39	-2.250780922	100.61169	0.0266	0.14	ns	39	1.1751757	80.32666	0.243	0.405	ns

40	-2.350512753	100.37569	0.0207	0.129375	ns	40	1.2069523	80.30418	0.231	0.405	ns
41	-2.436071217	100.36392	0.0166	0.129375	ns	41	1.2353869	80.71864	0.22	0.405	ns
42	-2.506591849	100.82957	0.0138	0.129375	ns	42	1.2700607	81.44036	0.208	0.405	ns
43	-2.568669776	101.69979	0.0117	0.129375	ns	43	1.3189283	82.37022	0.191	0.405	ns
44	-2.619976648	102.78228	0.0101	0.129375	ns	44	1.385797	83.40563	0.17	0.405	ns
45	-2.654501181	103.52479	0.0092	0.129375	ns	45	1.4623476	84.38454	0.147	0.405	ns
46	-2.662806786	103.71077	0.00898	0.129375	ns	46	1.5451536	85.17988	0.126	0.405	ns
47	-2.64080482	103.34143	0.00955	0.129375	ns	47	1.6252331	85.78437	0.108	0.37241379	ns
48	-2.590826457	102.5682	0.011	0.129375	ns	48	1.7077887	86.2316	0.0913	0.33814815	ns
49	-2.509937306	101.365	0.0137	0.129375	ns	49	1.7927975	86.56723	0.0765	0.29423077	ns
50	-2.382196225	100.38037	0.0191	0.129375	ns	50	1.8903075	86.71496	0.0621	0.2548	ns
51	-2.217350465	99.83995	0.0289	0.1445	ns	51	2.014285	86.66674	0.0471	0.20478261	ns
52	-2.017887282	99.55322	0.0463	0.1780769	ns	52	2.1614298	86.49266	0.0334	0.15904762	ns
53	-1.781470912	99.05851	0.0779	0.2434375	ns	53	2.3048849	86.35323	0.0236	0.12421053	ns
54	-1.501848892	98.49004	0.136	0.3487179	ns	54	2.4376764	86.1655	0.0168	0.09333333	ns
55	-1.197330669	98.24155	0.234	0.4875	ns	55	2.5607759	85.97498	0.0122	0.07625	ns
56	-0.884832713	98.47977	0.378	0.63	ns	56	2.6735464	85.74661	0.00898	0.06414286	ns
57	-0.588585952	98.87558	0.557	0.7957143	ns	57	2.7784419	85.49535	0.00671	0.05161538	ns
58	-0.319432084	99.64307	0.75	0.8715909	ns	58	2.8821245	85.42243	0.00499	0.04536364	*
59	-0.077785186	100.55187	0.938	0.9642857	ns	59	2.9859628	85.44835	0.00369	0.041	*
60	0.130618566	101.65467	0.896	0.9431579	ns	60	3.0751662	85.55238	0.00282	0.04085714	*
61	0.30238771	103.34065	0.763	0.8715909	ns	61	3.1352735	85.7997	0.00235	0.04085714	*
62	0.418787301	105.47068	0.676	0.8345679	ns	62	3.1610467	86.37543	0.00217	0.04085714	*
63	0.484320275	106.88873	0.629	0.81375	ns	63	3.1557359	87.4029	0.0022	0.04085714	*
64	0.512560359	107.1128	0.609	0.8092105	ns	64	3.1317389	88.7797	0.00235	0.04085714	*

65	0.505026112	107.17359	0.615	0.8092105	ns	65	3.0992645	90.14111	0.00259	0.04085714	*
66	0.463777748	107.36475	0.644	0.81375	ns	66	3.0647329	91.41715	0.00286	0.04085714	*
67	0.371406976	107.20522	0.711	0.8464286	ns	67	3.0076239	92.32359	0.00339	0.041	*
68	0.22725658	106.6529	0.821	0.9119565	ns	68	2.9153462	92.8861	0.00445	0.0445	*
69	0.044099672	106.02447	0.965	0.9747475	ns	69	2.7826132	93.21509	0.00653	0.05161538	ns
70	-0.156795681	105.33714	0.876	0.9419355	ns	70	2.6166634	93.39295	0.0104	0.06933333	ns
71	-0.381801644	104.975	0.703	0.8464286	ns	71	2.4450507	93.66331	0.0164	0.09333333	ns
72	-0.630791668	105.32967	0.53	0.7794118	ns	72	2.2678659	94.02312	0.0256	0.128	ns
73	-0.897649292	106.17989	0.371	0.6288136	ns	73	2.0776921	94.55583	0.0404	0.18363636	ns
74	-1.176426006	107.18213	0.242	0.4938776	ns	74	1.876181	95.36194	0.0637	0.2548	ns
75	-1.430298984	108.15823	0.156	0.3761905	ns	75	1.6563837	96.05965	0.101	0.36071429	ns
76	-1.64793232	108.84726	0.102	0.2944444	ns	76	1.4311199	96.70493	0.156	0.405	ns
77	-1.838782081	109.09583	0.0687	0.2368966	ns	77	1.218433	97.76672	0.226	0.405	ns
78	-2.016095971	108.72082	0.0463	0.1780769	ns	78	1.0475318	99.74461	0.297	0.43239437	ns
79	-2.163461816	108.12056	0.0327	0.1557143	ns	79	0.9253447	102.29428	0.357	0.46363636	ns
80	-2.272400356	107.52291	0.0251	0.14	ns	80	0.862443	104.97625	0.39	0.47291667	ns
81	-2.364145685	106.96034	0.0199	0.129375	ns	81	0.8501066	107.36997	0.397	0.47291667	ns
82	-2.444973371	106.26688	0.0161	0.129375	ns	82	0.8778204	109.21898	0.382	0.47160494	ns
83	-2.479684327	105.75421	0.0147	0.129375	ns	83	0.9320658	109.88292	0.353	0.46363636	ns
84	-2.450078312	105.72323	0.0159	0.129375	ns	84	0.9904957	108.97357	0.324	0.44931507	ns
85	-2.366596281	106.27047	0.0198	0.129375	ns	85	1.044383	106.76356	0.299	0.43239437	ns
86	-2.251683964	107.43896	0.0264	0.14	ns	86	1.0956459	103.53282	0.276	0.42461538	ns
87	-2.110502917	109.07405	0.0371	0.1613043	ns	87	1.1446459	99.76966	0.255	0.41269841	ns
88	-1.950557972	110.63368	0.0536	0.1985185	ns	88	1.1878579	96.06752	0.238	0.405	ns
89	-1.789804574	111.8894	0.0762	0.2434375	ns	89	1.2220577	92.75196	0.225	0.405	ns

90	-1.641232413	112.94861	0.104	0.2944444	ns	90	1.2414162	90.15348	0.218	0.405	ns
91	-1.526105835	113.7665	0.13	0.3421053	ns	91	1.2451466	88.48654	0.216	0.405	ns
92	-1.422515753	114.23886	0.158	0.3761905	ns	92	1.2323825	87.77755	0.221	0.405	ns
93	-1.313268578	114.24183	0.192	0.4266667	ns	93	1.1971291	88.75695	0.234	0.405	ns
94	-1.202159809	113.97415	0.232	0.4875	ns	94	1.1335657	91.88288	0.26	0.41269841	ns
95	-1.115858631	113.12928	0.267	0.5211538	ns	95	1.0307776	98.10379	0.305	0.43239437	ns
96	-1.038336749	111.96889	0.301	0.5563636	ns	96	0.8861811	106.23249	0.378	0.47160494	ns
97	-0.937672348	110.80679	0.35	0.6051724	ns	97	0.7197681	114.29163	0.473	0.48762887	ns
98	-0.826692648	110.54042	0.41	0.6612903	ns	98	0.5601707	119.95173	0.576	0.5877551	ns
99	-0.700167028	111.31393	0.485	0.7358209	ns	99	0.4457186	121.9867	0.657	0.66363636	ns
100	-0.540762135	112.44185	0.59	0.8082192	ns	100	0.3788327	120.99932	0.705	0.705	ns
19	statistic	df	p	p.adj	p.adj.signif	20	statistic	df	p	p.adj	p.adj.signif
1	-2.61193466	127.8091	0.0101	0.08416667	ns	1	-0.227203152	93.4249	0.821	0.91098901	ns
2	-2.68907643	127.991	0.00812	0.08416667	ns	2	-0.335133589	93.47812	0.738	0.85813953	ns
3	-2.69358964	127.8957	0.00802	0.08416667	ns	3	-0.41009218	93.97492	0.683	0.83529412	ns
4	-2.69475686	127.47392	0.00799	0.08416667	ns	4	-0.460804406	94.23849	0.646	0.83529412	ns
5	-2.71181925	126.93748	0.00762	0.08416667	ns	5	-0.578089151	94.49341	0.565	0.83529412	ns
6	-2.7072153	126.45264	0.00772	0.08416667	ns	6	-0.7372821	95.07662	0.463	0.83529412	ns
7	-2.67595687	126.38259	0.00844	0.08416667	ns	7	-0.866592513	95.35604	0.388	0.776	ns
8	-2.61420078	126.79249	0.01	0.08416667	ns	8	-0.939891994	94.95421	0.35	0.74468085	ns
9	-2.53794218	127.3279	0.0124	0.0875	ns	9	-0.944396072	93.62592	0.347	0.74468085	ns
10	-2.4334302	127.73979	0.0163	0.09588235	ns	10	-0.878017806	91.75538	0.382	0.776	ns
11	-2.28015453	127.97114	0.0243	0.135	ns	11	-0.739916211	89.86465	0.461	0.83529412	ns
12	-2.11758145	127.9599	0.0361	0.1805	ns	12	-0.583176142	88.50624	0.561	0.83529412	ns
13	-1.97425282	127.78673	0.0505	0.24047619	ns	13	-0.46436384	87.75234	0.644	0.83529412	ns

14	-1.819016	127.6447	0.0713	0.32409091	ns	14	-0.419789774	86.97334	0.676	0.83529412	ns
15	-1.65228396	127.54096	0.101	0.42083333	ns	15	-0.430479797	86.19951	0.668	0.83529412	ns
16	-1.50021724	127.36617	0.136	0.544	ns	16	-0.474333449	86.02204	0.636	0.83529412	ns
17	-1.36207644	127.17641	0.176	0.65185185	ns	17	-0.554918833	85.92225	0.58	0.83529412	ns
18	-1.23537795	127.05737	0.219	0.70645161	ns	18	-0.607647191	86.08763	0.545	0.83529412	ns
19	-1.12706637	127.05255	0.262	0.76363636	ns	19	-0.59843382	86.50138	0.551	0.83529412	ns
20	-1.02336982	127.11639	0.308	0.76363636	ns	20	-0.552120693	87.1121	0.582	0.83529412	ns
21	-0.91541356	127.12774	0.362	0.76363636	ns	21	-0.507905247	87.84812	0.613	0.83529412	ns
22	-0.82722021	127.10128	0.41	0.76363636	ns	22	-0.465493265	89.08636	0.643	0.83529412	ns
23	-0.80308241	126.79822	0.423	0.76363636	ns	23	-0.445604396	90.92039	0.657	0.83529412	ns
24	-0.83072522	126.28029	0.408	0.76363636	ns	24	-0.438207362	92.82307	0.662	0.83529412	ns
25	-0.89519017	125.94114	0.372	0.76363636	ns	25	-0.4132057	94.41368	0.68	0.83529412	ns
26	-0.9426956	125.70377	0.348	0.76363636	ns	26	-0.382862719	96.38807	0.703	0.83529412	ns
27	-0.92298732	125.42666	0.358	0.76363636	ns	27	-0.37232848	98.22968	0.71	0.83529412	ns
28	-0.82998034	124.82421	0.408	0.76363636	ns	28	-0.388817689	99.45768	0.698	0.83529412	ns
29	-0.66944408	123.99194	0.504	0.76363636	ns	29	-0.431616924	99.9553	0.667	0.83529412	ns
30	-0.47929236	123.30267	0.633	0.84210526	ns	30	-0.493252916	100.20586	0.623	0.83529412	ns
31	-0.27628833	122.94867	0.783	0.9	ns	31	-0.538673862	100.52096	0.591	0.83529412	ns
32	-0.07984581	123.04718	0.936	0.96969697	ns	32	-0.594112334	100.56932	0.554	0.83529412	ns
33	0.08720978	123.81354	0.931	0.96969697	ns	33	-0.682903578	100.998	0.496	0.83529412	ns
34	0.23344214	125.062	0.816	0.91685393	ns	34	-0.810681032	102.24699	0.419	0.82156863	ns
35	0.34736256	126.46657	0.729	0.87831325	ns	35	-1.047252761	104.23092	0.297	0.675	ns
36	0.42022489	127.49527	0.675	0.86538462	ns	36	-1.368541341	106.73074	0.174	0.42439024	ns
37	0.46836765	127.94254	0.64	0.84210526	ns	37	-1.663011482	108.81578	0.0992	0.26105263	ns
38	0.50113997	127.97788	0.617	0.84210526	ns	38	-1.863488387	111.23758	0.065	0.18055556	ns

39	0.50874874	127.81199	0.612	0.84210526	ns	39	-2.002873733	114.13493	0.0476	0.14411765	ns
40	0.4969827	127.65142	0.62	0.84210526	ns	40	-2.115955449	116.89738	0.0365	0.1140625	ns
41	0.47739195	127.54613	0.634	0.84210526	ns	41	-2.245207802	118.75176	0.0266	0.08866667	ns
42	0.43168165	127.4988	0.667	0.86538462	ns	42	-2.425739668	119.92548	0.0168	0.06461538	ns
43	0.37511204	127.59029	0.708	0.87195122	ns	43	-2.620749191	120.68082	0.0099	0.05210526	ns
44	0.33085581	127.69352	0.741	0.88214286	ns	44	-2.832387873	121.41746	0.00541	0.03864286	*
45	0.30183008	127.73699	0.763	0.88837209	ns	45	-3.01350597	121.95094	0.00314	0.0314	*
46	0.30148559	127.7444	0.764	0.88837209	ns	46	-3.092100533	122.33727	0.00246	0.0314	*
47	0.36663055	127.81765	0.715	0.87195122	ns	47	-3.079943903	122.12455	0.00256	0.0314	*
48	0.49754248	127.94548	0.62	0.84210526	ns	48	-3.036728287	120.63274	0.00293	0.0314	*
49	0.6305348	127.99775	0.529	0.77794118	ns	49	-2.958597013	117.73225	0.00374	0.03284615	*
50	0.72500157	127.98148	0.47	0.76363636	ns	50	-2.76509048	114.01081	0.00664	0.0415	*
51	0.79366605	127.89039	0.429	0.76363636	ns	51	-2.43835546	109.90261	0.0164	0.06461538	ns
52	0.79795448	127.7901	0.426	0.76363636	ns	52	-1.991239485	106.6783	0.049	0.14411765	ns
53	0.74202919	127.71346	0.459	0.76363636	ns	53	-1.465941235	107.59475	0.146	0.365	ns
54	0.68510249	127.57178	0.495	0.76363636	ns	54	-0.975906466	110.80253	0.331	0.73555556	ns
55	0.67639986	127.42447	0.5	0.76363636	ns	55	-0.654963406	113.88712	0.514	0.83529412	ns
56	0.70725841	127.36659	0.481	0.76363636	ns	56	-0.495087515	117.13587	0.621	0.83529412	ns
57	0.72910619	127.4537	0.467	0.76363636	ns	57	-0.394245056	121.00317	0.694	0.83529412	ns
58	0.70804259	127.65701	0.48	0.76363636	ns	58	-0.318744085	123.54699	0.75	0.86206897	ns
59	0.69973172	127.83615	0.485	0.76363636	ns	59	-0.272569412	123.65125	0.786	0.89318182	ns
60	0.73133568	127.9402	0.466	0.76363636	ns	60	-0.183167575	123.22489	0.855	0.91935484	ns
61	0.78158365	127.99799	0.436	0.76363636	ns	61	-0.125976049	122.96588	0.9	0.94736842	ns
62	0.78632779	127.8776	0.433	0.76363636	ns	62	-0.089093663	121.97679	0.929	0.95773196	ns
63	0.64053766	127.04978	0.523	0.77794118	ns	63	-0.057827175	119.95254	0.954	0.97346939	ns

64	0.37719051	124.75448	0.707	0.87195122	ns	64	-0.007468048	118.87946	0.994	0.994	ns
65	0.07687878	120.89748	0.939	0.96969697	ns	65	0.091496034	120.33174	0.927	0.95773196	ns
66	-0.15070684	117.00992	0.88	0.95652174	ns	66	0.197663157	123.19703	0.844	0.9173913	ns
67	-0.24049418	115.59649	0.81	0.91685393	ns	67	0.216011466	125.07717	0.829	0.91098901	ns
68	-0.19625995	116.36537	0.845	0.92857143	ns	68	0.146721974	125.92961	0.884	0.94042553	ns
69	-0.10121778	117.84667	0.92	0.96969697	ns	69	-0.027465619	126.33143	0.978	0.98787879	ns
70	-0.00863754	118.89243	0.993	0.993	ns	70	-0.246208495	126.93468	0.806	0.90561798	ns
71	0.06768278	119.49779	0.946	0.96969697	ns	71	-0.422372164	127.68289	0.673	0.83529412	ns
72	0.05069228	119.89967	0.96	0.96969697	ns	72	-0.532998057	127.99859	0.595	0.83529412	ns
73	-0.05816387	119.51611	0.954	0.96969697	ns	73	-0.622748948	127.7705	0.535	0.83529412	ns
74	-0.21654931	118.69231	0.829	0.92111111	ns	74	-0.748481897	127.89868	0.456	0.83529412	ns
75	-0.40033905	118.0669	0.69	0.87195122	ns	75	-0.898445115	127.98616	0.371	0.77291667	ns
76	-0.57589474	117.8281	0.566	0.82028986	ns	76	-1.102997395	127.94433	0.272	0.63255814	ns
77	-0.68826892	117.587	0.493	0.76363636	ns	77	-1.319922907	127.97489	0.189	0.45	ns
78	-0.76394269	116.48524	0.446	0.76363636	ns	78	-1.510285529	127.95801	0.133	0.34102564	ns
79	-0.81880546	113.19069	0.415	0.76363636	ns	79	-1.698836184	127.72701	0.0918	0.24810811	ns
80	-0.92698803	105.35819	0.356	0.76363636	ns	80	-1.933210681	127.61996	0.0554	0.15828571	ns
81	-1.09631297	95.26554	0.276	0.76363636	ns	81	-2.200602697	127.91987	0.0296	0.09548387	ns
82	-1.23980862	87.15896	0.218	0.70645161	ns	82	-2.435863383	127.83136	0.0162	0.06461538	ns
83	-1.29950622	83.17562	0.197	0.70357143	ns	83	-2.570027265	127.005	0.0113	0.05380952	ns
84	-1.26585568	82.66289	0.209	0.70645161	ns	84	-2.630077005	125.97041	0.0096	0.05210526	ns
85	-1.14082449	84.37021	0.257	0.76363636	ns	85	-2.57596598	125.64097	0.0112	0.05380952	ns
86	-0.98355578	85.8465	0.328	0.76363636	ns	86	-2.457471616	126.22913	0.0153	0.06461538	ns
87	-0.8549424	85.6816	0.395	0.76363636	ns	87	-2.386108146	126.85228	0.0185	0.06827586	ns
88	-0.82879699	85.08554	0.41	0.76363636	ns	88	-2.358986609	127.46766	0.0198	0.06827586	ns

89	-0.93644798	86.12455	0.352	0.76363636	ns	89	-2.372528986	127.88014	0.0192	0.06827586	ns
90	-1.14009263	90.6848	0.257	0.76363636	ns	90	-2.475315113	127.91585	0.0146	0.06461538	ns
91	-1.42685692	98.35072	0.157	0.60384615	ns	91	-2.662883893	127.68476	0.00874	0.05141176	ns
92	-1.80063575	105.99953	0.0746	0.32434783	ns	92	-2.909892301	127.17249	0.00427	0.03284615	*
93	-2.18629768	110.91214	0.0309	0.16263158	ns	93	-3.215033887	126.66954	0.00166	0.02766667	*
94	-2.49702155	112.81571	0.014	0.0875	ns	94	-3.485829317	125.64963	0.000676	0.018625	*
95	-2.69929125	112.54182	0.00802	0.08416667	ns	95	-3.654979032	125.11746	0.000377	0.018625	*
96	-2.75392934	110.96625	0.00688	0.08416667	ns	96	-3.635677517	124.95675	0.000404	0.018625	*
97	-2.71846837	110.07481	0.00762	0.08416667	ns	97	-3.458247655	124.38019	0.000745	0.018625	*
98	-2.62180592	110.92585	0.00997	0.08416667	ns	98	-3.217107242	123.31547	0.00165	0.02766667	*
99	-2.55628017	113.81843	0.0119	0.0875	ns	99	-2.929656351	121.77556	0.00405	0.03284615	*
100	-2.5054556	117.18649	0.0136	0.0875	ns	100	-2.781213778	119.07956	0.0063	0.0415	*

Table S5： Comparison of Mean AD Between prodromal PD carriers of dual LRRK2 and GBA variants and Healthy Controls Across 20 Fiber Tracts. Statistical significance was set at $p < 0.05$, corrected by FDR.

1	statistic	df	p	p.adj	p.adj.signif	2	statistic	df	p	p.adj	p.adj.signif
1	0.75536639	124.15063	0.451	0.7242424	ns	1	-0.01077561	127.67642	0.991	0.991	ns
2	0.83672949	124.04163	0.404	0.7242424	ns	2	0.13172806	127.91657	0.895	0.9132653	ns
3	0.90771982	123.70849	0.366	0.690566	ns	3	0.25799803	127.99679	0.797	0.8569892	ns
4	0.96542844	123.14301	0.336	0.6588235	ns	4	0.35121588	127.99314	0.726	0.8191011	ns
5	1.03461061	122.56543	0.303	0.6333333	ns	5	0.41808393	127.98236	0.677	0.8149425	ns
6	1.10095058	122.10019	0.273	0.6066667	ns	6	0.4473446	127.98791	0.655	0.8073171	ns
7	1.18022668	122.17457	0.24	0.5674419	ns	7	0.45979769	127.99348	0.646	0.8073171	ns
8	1.26330734	122.61703	0.209	0.535	ns	8	0.47466021	127.99572	0.636	0.8073171	ns

9	1.33531292	123.18895	0.184	0.4972973	ns	9	0.49283977	127.99618	0.623	0.8073171	ns
10	1.40137643	123.78566	0.164	0.4685714	ns	10	0.51472093	127.99911	0.608	0.8073171	ns
11	1.46124928	124.39551	0.146	0.4470588	ns	11	0.53787828	127.99408	0.592	0.8073171	ns
12	1.50909081	124.97284	0.134	0.4322581	ns	12	0.56930002	127.92905	0.57	0.8073171	ns
13	1.52605775	125.26974	0.13	0.4322581	ns	13	0.61220382	127.69176	0.541	0.8073171	ns
14	1.50750082	125.04097	0.134	0.4322581	ns	14	0.65382835	127.11719	0.514	0.8073171	ns
15	1.45446071	123.88664	0.148	0.4470588	ns	15	0.67176541	125.97609	0.503	0.8073171	ns
16	1.37975498	121.42752	0.17	0.4722222	ns	16	0.66032672	124.0289	0.51	0.8073171	ns
17	1.29886442	117.72041	0.197	0.5184211	ns	17	0.63386487	121.49009	0.527	0.8073171	ns
18	1.2237784	112.98521	0.224	0.5463415	ns	18	0.58530026	118.76324	0.559	0.8073171	ns
19	1.1707398	107.93669	0.244	0.5674419	ns	19	0.51542059	116.05471	0.607	0.8073171	ns
20	1.1335379	103.22199	0.26	0.5909091	ns	20	0.44137587	113.61188	0.66	0.8073171	ns
21	1.08600811	99.2889	0.28	0.6086957	ns	21	0.3736342	111.58965	0.709	0.8149425	ns
22	1.03280753	96.42381	0.304	0.6333333	ns	22	0.34768415	110.07216	0.729	0.8191011	ns
23	0.97607012	94.65044	0.332	0.6588235	ns	23	0.38045995	108.88521	0.704	0.8149425	ns
24	0.91163695	94.04999	0.364	0.690566	ns	24	0.47042255	107.82382	0.639	0.8073171	ns
25	0.83932989	94.43735	0.403	0.7242424	ns	25	0.59052954	106.83624	0.556	0.8073171	ns
26	0.77092261	95.69174	0.443	0.7242424	ns	26	0.70781574	106.23628	0.481	0.8073171	ns
27	0.71690653	97.22833	0.475	0.7242424	ns	27	0.79943556	106.2173	0.426	0.8073171	ns
28	0.66799129	98.50155	0.506	0.7305556	ns	28	0.86486068	106.3507	0.389	0.8073171	ns
29	0.63953183	99.42824	0.524	0.7305556	ns	29	0.90632398	105.96343	0.367	0.8073171	ns
30	0.63653639	100.16873	0.526	0.7305556	ns	30	0.93947822	105.07337	0.35	0.8073171	ns
31	0.65763323	100.83089	0.512	0.7305556	ns	31	0.98408073	103.98997	0.327	0.8073171	ns
32	0.68876734	101.17327	0.493	0.7305556	ns	32	1.05726395	102.8082	0.293	0.8054054	ns
33	0.71967033	100.91717	0.473	0.7242424	ns	33	1.15682229	101.57494	0.25	0.78125	ns

34	0.73738383	100.21342	0.463	0.7242424	ns	34	1.28491094	100.59019	0.202	0.7214286	ns
35	0.75032117	99.823	0.455	0.7242424	ns	35	1.45795043	100.28005	0.148	0.592	ns
36	0.76418654	100.13291	0.447	0.7242424	ns	36	1.66775312	100.46457	0.0985	0.4695652	ns
37	0.76731782	100.86652	0.445	0.7242424	ns	37	1.86061479	100.56201	0.0657	0.4695652	ns
38	0.74365008	101.9606	0.459	0.7242424	ns	38	1.98576691	100.30385	0.0498	0.4695652	ns
39	0.71175283	103.33545	0.478	0.7242424	ns	39	2.02304953	99.23648	0.0458	0.4695652	ns
40	0.66961012	104.83695	0.505	0.7305556	ns	40	1.99876229	97.41057	0.0484	0.4695652	ns
41	0.61080429	106.28549	0.543	0.7438356	ns	41	1.93995517	94.87037	0.0554	0.4695652	ns
42	0.54209048	107.52657	0.589	0.7959459	ns	42	1.89694546	92.29384	0.061	0.4695652	ns
43	0.47261416	107.88883	0.637	0.8063291	ns	43	1.86541543	90.19996	0.0654	0.4695652	ns
44	0.40598347	106.79322	0.686	0.8088889	ns	44	1.85555066	88.67501	0.0668	0.4695652	ns
45	0.36199987	104.64465	0.718	0.8088889	ns	45	1.88101628	87.20033	0.0633	0.4695652	ns
46	0.34858539	101.93566	0.728	0.8088889	ns	46	1.91092236	85.43861	0.0594	0.4695652	ns
47	0.37402443	99.15603	0.709	0.8088889	ns	47	1.90341278	83.57941	0.0604	0.4695652	ns
48	0.41965494	96.17838	0.676	0.8088889	ns	48	1.84154796	81.98017	0.0692	0.4695652	ns
49	0.47424052	92.5213	0.636	0.8063291	ns	49	1.75123935	80.9505	0.0837	0.4695652	ns
50	0.51164091	88.4247	0.61	0.8063291	ns	50	1.66993066	80.48275	0.0988	0.4695652	ns
51	0.50158828	84.33621	0.617	0.8063291	ns	51	1.62335755	80.55806	0.108	0.4695652	ns
52	0.44009324	81.08817	0.661	0.8088889	ns	52	1.62705081	81.26522	0.108	0.4695652	ns
53	0.35714885	79.06489	0.722	0.8088889	ns	53	1.66368121	82.49452	0.1	0.4695652	ns
54	0.27942169	78.1481	0.781	0.8397849	ns	54	1.69710583	83.85329	0.0934	0.4695652	ns
55	0.22583856	78.18649	0.822	0.8438776	ns	55	1.71497561	85.11546	0.09	0.4695652	ns
56	0.21889484	78.74114	0.827	0.8438776	ns	56	1.71236531	86.08508	0.0904	0.4695652	ns
57	0.25696923	79.71794	0.798	0.84	ns	57	1.69245872	86.8354	0.0941	0.4695652	ns
58	0.30508399	81.06412	0.761	0.8271739	ns	58	1.64493149	87.4225	0.104	0.4695652	ns

59	0.35281585	82.68754	0.725	0.8088889	ns	59	1.55602931	88.05299	0.123	0.5125	ns
60	0.38022884	84.51464	0.705	0.8088889	ns	60	1.43814464	88.73108	0.154	0.5923077	ns
61	0.38716294	86.60897	0.7	0.8088889	ns	61	1.30869489	89.5516	0.194	0.7185185	ns
62	0.3722029	88.9931	0.711	0.8088889	ns	62	1.17830653	90.71224	0.242	0.78125	ns
63	0.32158096	91.56193	0.749	0.8230769	ns	63	1.04569153	92.17314	0.298	0.8054054	ns
64	0.23157394	94.07308	0.817	0.8438776	ns	64	0.91657471	93.53607	0.362	0.8073171	ns
65	0.10842075	96.18698	0.914	0.9232323	ns	65	0.79630212	94.0756	0.428	0.8073171	ns
66	-0.05605341	98.01847	0.955	0.955	ns	66	0.68506824	93.1096	0.495	0.8073171	ns
67	-0.2561304	99.59857	0.798	0.84	ns	67	0.58247427	90.40478	0.562	0.8073171	ns
68	-0.49290576	100.89594	0.623	0.8063291	ns	68	0.50763113	86.83272	0.613	0.8073171	ns
69	-0.75864716	101.96209	0.45	0.7242424	ns	69	0.4737334	83.61344	0.637	0.8073171	ns
70	-1.01866259	102.59005	0.311	0.6346939	ns	70	0.48041109	81.54366	0.632	0.8073171	ns
71	-1.25090232	102.84587	0.214	0.535	ns	71	0.49470787	80.71587	0.622	0.8073171	ns
72	-1.44414107	103.10123	0.152	0.4470588	ns	72	0.51126152	80.90633	0.611	0.8073171	ns
73	-1.58030997	103.2218	0.117	0.4178571	ns	73	0.52248646	81.88533	0.603	0.8073171	ns
74	-1.65047061	102.93812	0.102	0.4115385	ns	74	0.52449476	83.3632	0.601	0.8073171	ns
75	-1.65936117	101.52665	0.1	0.4115385	ns	75	0.5075324	85.26406	0.613	0.8073171	ns
76	-1.66193294	99.87974	0.0997	0.4115385	ns	76	0.4610515	87.74617	0.646	0.8073171	ns
77	-1.6730905	98.59581	0.0975	0.4115385	ns	77	0.3806021	90.96516	0.704	0.8149425	ns
78	-1.67141577	97.52992	0.0978	0.4115385	ns	78	0.27496845	94.86815	0.784	0.8521739	ns
79	-1.66500485	96.76172	0.0991	0.4115385	ns	79	0.13826675	99.29387	0.89	0.9132653	ns
80	-1.67710149	96.69356	0.0968	0.4115385	ns	80	-0.01438979	103.69304	0.989	0.991	ns
81	-1.69019098	97.20142	0.0942	0.4115385	ns	81	-0.16850421	107.22462	0.867	0.903125	ns
82	-1.71988818	98.34458	0.0886	0.4115385	ns	82	-0.33424872	109.31628	0.739	0.8211111	ns
83	-1.76497681	99.98776	0.0806	0.4115385	ns	83	-0.5159656	110.44446	0.607	0.8073171	ns

84	-1.80591596	101.83654	0.0739	0.4115385	ns	84	-0.69140407	111.04884	0.491	0.8073171	ns
85	-1.84533784	103.81861	0.0678	0.4115385	ns	85	-0.83786104	111.39349	0.404	0.8073171	ns
86	-1.88624517	105.77575	0.062	0.4115385	ns	86	-0.93440283	111.07169	0.352	0.8073171	ns
87	-1.92581021	108.09792	0.0568	0.4115385	ns	87	-1.08837074	111.66242	0.279	0.8054054	ns
88	-1.941713	110.81186	0.0547	0.4115385	ns	88	-1.15703496	111.9822	0.25	0.78125	ns
89	-1.91982476	113.56268	0.0574	0.4115385	ns	89	-1.15837379	112.09387	0.249	0.78125	ns
90	-1.85210716	116.03961	0.0666	0.4115385	ns	90	-1.08190816	111.76512	0.282	0.8054054	ns
91	-1.74482704	118.26641	0.0836	0.4115385	ns	91	-0.90956265	111.36654	0.365	0.8073171	ns
92	-1.63775031	120.12672	0.104	0.4115385	ns	92	-0.66354434	110.46133	0.508	0.8073171	ns
93	-1.58420069	121.51869	0.116	0.4178571	ns	93	-0.40393955	108.85758	0.687	0.8149425	ns
94	-1.62242547	122.47186	0.107	0.4115385	ns	94	-0.22196949	106.88914	0.825	0.8776596	ns
95	-1.75317734	122.99163	0.0821	0.4115385	ns	95	-0.18748473	104.27319	0.852	0.8968421	ns
96	-1.94079683	123.08206	0.0546	0.4115385	ns	96	-0.27478621	101.38501	0.784	0.8521739	ns
97	-2.13709773	122.4742	0.0346	0.4115385	ns	97	-0.43893338	98.84717	0.662	0.8073171	ns
98	-2.3053617	120.63618	0.0229	0.4115385	ns	98	-0.67139111	97.68394	0.504	0.8073171	ns
99	-2.46739498	116.92398	0.0151	0.4115385	ns	99	-0.9185557	97.53724	0.361	0.8073171	ns
100	-2.64861705	111.67505	0.00925	0.4115385	ns	100	-1.0474416	97.57937	0.297	0.8054054	ns
3	statistic	df	p	p.adj	p.adj.signif	4	statistic	df	p	p.adj	p.adj.signif
1	-0.43554444	113.83289	0.664	0.77209302	ns	1	-0.6083305	121.10897	0.544	0.7157895	ns
2	-0.33206192	117.97835	0.74	0.84090909	ns	2	-0.66033445	120.43269	0.51	0.7083333	ns
3	-0.25189127	121.35646	0.802	0.89111111	ns	3	-0.58973468	120.75169	0.556	0.7220779	ns
4	-0.19999127	123.84111	0.842	0.896875	ns	4	-0.39531914	121.65575	0.693	0.805814	ns
5	-0.17882498	125.17002	0.858	0.896875	ns	5	-0.09063371	123.16692	0.928	0.9469388	ns
6	-0.19718363	125.83605	0.844	0.896875	ns	6	0.19123058	124.26914	0.849	0.9031915	ns
7	-0.20862289	126.27257	0.835	0.896875	ns	7	0.39664458	124.95165	0.692	0.805814	ns

8	-0.18299291	126.32006	0.855	0.896875	ns	8	0.55775529	124.00253	0.578	0.7275	ns
9	-0.08740236	125.56797	0.93	0.94242424	ns	9	0.69375976	121.05963	0.489	0.7083333	ns
10	0.02000024	123.7526	0.984	0.984	ns	10	0.82888047	115.46005	0.409	0.6924242	ns
11	0.10355248	120.31697	0.918	0.94242424	ns	11	0.93366324	109.24019	0.353	0.6924242	ns
12	0.17498915	115.91466	0.861	0.896875	ns	12	1.04296443	102.77184	0.299	0.6924242	ns
13	0.30638716	111.56645	0.76	0.85393258	ns	13	1.23865075	96.18283	0.218	0.6924242	ns
14	0.47573713	107.83913	0.635	0.75595238	ns	14	1.44915413	91.33062	0.151	0.5807692	ns
15	0.63011431	105.04055	0.53	0.64634146	ns	15	1.59061174	88.06773	0.115	0.547619	ns
16	0.75105748	103.63924	0.454	0.60533333	ns	16	1.69484162	85.67002	0.0937	0.4931579	ns
17	0.84014221	104.32539	0.403	0.60533333	ns	17	1.82785914	83.98469	0.0711	0.405	ns
18	0.86719039	106.33258	0.388	0.60533333	ns	18	1.98774637	83.91731	0.0501	0.313125	ns
19	0.80284476	108.75298	0.424	0.60533333	ns	19	2.11153539	83.96434	0.0377	0.2692857	ns
20	0.71563071	111.39584	0.476	0.61538462	ns	20	2.18473679	83.76222	0.0317	0.2438462	ns
21	0.6950843	113.95553	0.488	0.61772152	ns	21	2.21070442	83.9201	0.0298	0.2438462	ns
22	0.79295923	115.5366	0.429	0.60533333	ns	22	2.21964671	84.93204	0.0291	0.2438462	ns
23	0.94799475	115.83488	0.345	0.60533333	ns	23	2.27064302	86.51385	0.0257	0.2438462	ns
24	1.1401609	115.18677	0.257	0.55869565	ns	24	2.39215976	88.28272	0.0189	0.23625	ns
25	1.38034996	113.46659	0.17	0.4047619	ns	25	2.59444948	89.87019	0.0111	0.1728571	ns
26	1.62853767	109.7512	0.106	0.29444444	ns	26	2.81653622	91.14112	0.00595	0.119	ns
27	1.81371896	104.89657	0.0726	0.23419355	ns	27	2.99772414	91.57334	0.0035	0.1166667	ns
28	1.90279489	99.56066	0.06	0.21464286	ns	28	3.1134353	90.6422	0.00247	0.1166667	ns
29	1.93206685	95.04368	0.0563	0.21464286	ns	29	3.05207291	88.55044	0.003	0.1166667	ns
30	1.9559097	92.4035	0.0535	0.21464286	ns	30	2.83677727	86.20219	0.00568	0.119	ns
31	1.96591141	90.7063	0.0524	0.21464286	ns	31	2.5649357	83.88609	0.0121	0.1728571	ns
32	1.92250455	89.19425	0.0577	0.21464286	ns	32	2.27608037	81.25247	0.0255	0.2438462	ns

33	1.7739254	87.3507	0.0796	0.24818182	ns	33	2.02050249	79.03144	0.0467	0.3113333	ns
34	1.51503832	85.40328	0.133	0.35	ns	34	1.81780982	78.30533	0.0729	0.405	ns
35	1.19424075	83.98348	0.236	0.52444444	ns	35	1.61278908	78.6187	0.111	0.547619	ns
36	0.821797	83.66828	0.414	0.60533333	ns	36	1.45465529	79.23611	0.15	0.5807692	ns
37	0.3886555	85.09961	0.699	0.80344828	ns	37	1.32340692	79.78202	0.189	0.675	ns
38	-0.08378265	88.01042	0.933	0.94242424	ns	38	1.18135714	79.91664	0.241	0.6924242	ns
39	-0.58002287	90.82987	0.563	0.67831325	ns	39	0.98829975	79.36214	0.326	0.6924242	ns
40	-1.03463541	92.97727	0.304	0.59607843	ns	40	0.74826573	78.75035	0.457	0.6924242	ns
41	-1.38288555	95.00708	0.17	0.4047619	ns	41	0.47376241	78.67121	0.637	0.7674699	ns
42	-1.65120938	97.5558	0.102	0.29444444	ns	42	0.20214278	79.03669	0.84	0.9031915	ns
43	-1.85314135	99.82	0.0668	0.22266667	ns	43	-0.05196174	79.82489	0.959	0.959	ns
44	-1.98458216	101.16342	0.0499	0.21464286	ns	44	-0.30565321	80.93955	0.761	0.8384615	ns
45	-2.1038657	101.23205	0.0379	0.21464286	ns	45	-0.55289955	81.92803	0.582	0.7275	ns
46	-2.25282869	100.53905	0.0264	0.21464286	ns	46	-0.78051627	82.52215	0.437	0.6924242	ns
47	-2.41612813	99.63955	0.0175	0.16818182	ns	47	-0.93606616	82.88259	0.352	0.6924242	ns
48	-2.58194695	98.98948	0.0113	0.12555556	ns	48	-0.96484903	82.86033	0.337	0.6924242	ns
49	-2.75585687	99.03912	0.00697	0.09957143	ns	49	-0.91677095	82.55563	0.362	0.6924242	ns
50	-2.9455489	100.18056	0.00401	0.07783333	ns	50	-0.83801709	82.82101	0.404	0.6924242	ns
51	-3.10843685	101.89591	0.00244	0.0695	ns	51	-0.78704942	84.22675	0.433	0.6924242	ns
52	-3.18584098	103.86653	0.00191	0.0695	ns	52	-0.74781748	86.74591	0.457	0.6924242	ns
53	-3.17217419	105.88282	0.00198	0.0695	ns	53	-0.75996318	90.55581	0.449	0.6924242	ns
54	-3.06233176	107.25498	0.00278	0.0695	ns	54	-0.79249065	93.93075	0.43	0.6924242	ns
55	-2.88905548	107.80795	0.00467	0.07783333	ns	55	-0.80418568	96.47841	0.423	0.6924242	ns
56	-2.65815675	107.22229	0.00906	0.11325	ns	56	-0.76316133	98.51474	0.447	0.6924242	ns
57	-2.3926365	106.03473	0.0185	0.16818182	ns	57	-0.72445947	100.68331	0.47	0.7014925	ns

58	-2.11508798	104.65762	0.0368	0.21464286	ns	58	-0.70056612	102.65994	0.485	0.7083333	ns
59	-1.85970859	103.59759	0.0658	0.22266667	ns	59	-0.67852145	104.05771	0.499	0.7083333	ns
60	-1.63637217	103.63822	0.105	0.29444444	ns	60	-0.61289593	105.11954	0.541	0.7157895	ns
61	-1.45188966	105.32752	0.15	0.38461538	ns	61	-0.49151679	106.1349	0.624	0.7674699	ns
62	-1.2858679	109.006	0.201	0.46744186	ns	62	-0.32520585	106.80574	0.746	0.8384615	ns
63	-1.10971961	113.65558	0.269	0.57234043	ns	63	-0.14050766	107.26859	0.889	0.9357895	ns
64	-0.92789876	117.66747	0.355	0.60533333	ns	64	0.09481572	109.2365	0.925	0.9469388	ns
65	-0.76440916	120.36396	0.446	0.60533333	ns	65	0.30265013	110.97144	0.763	0.8384615	ns
66	-0.67274043	121.70796	0.502	0.61975309	ns	66	0.48225375	111.83366	0.631	0.7674699	ns
67	-0.67660265	121.89371	0.5	0.61975309	ns	67	0.6305411	111.65878	0.53	0.7157895	ns
68	-0.7543256	121.07436	0.452	0.60533333	ns	68	0.77821644	111.03106	0.438	0.6924242	ns
69	-0.83726225	119.24261	0.404	0.60533333	ns	69	0.90011967	110.19351	0.37	0.6924242	ns
70	-0.8974965	116.86677	0.371	0.60533333	ns	70	0.97026049	108.76489	0.334	0.6924242	ns
71	-0.91977044	114.81047	0.36	0.60533333	ns	71	0.9874165	106.6943	0.326	0.6924242	ns
72	-0.92581386	113.73734	0.357	0.60533333	ns	72	0.96497385	104.04302	0.337	0.6924242	ns
73	-0.95439971	113.14139	0.342	0.60533333	ns	73	0.94589019	101.51409	0.346	0.6924242	ns
74	-0.9963716	112.06348	0.321	0.59814815	ns	74	0.96259816	99.78481	0.338	0.6924242	ns
75	-1.04932814	110.57981	0.296	0.592	ns	75	1.00271167	98.93421	0.318	0.6924242	ns
76	-1.08676391	109.46616	0.28	0.58333333	ns	76	1.06322102	99.09568	0.29	0.6924242	ns
77	-1.07089815	109.3347	0.287	0.58571429	ns	77	1.12422444	100.22657	0.264	0.6924242	ns
78	-0.99794374	109.8143	0.321	0.59814815	ns	78	1.13539626	101.69627	0.259	0.6924242	ns
79	-0.89426996	110.50941	0.373	0.60533333	ns	79	1.09994842	102.94443	0.274	0.6924242	ns
80	-0.8162667	110.94522	0.416	0.60533333	ns	80	1.02991138	103.46869	0.305	0.6924242	ns
81	-0.78412376	110.61751	0.435	0.60533333	ns	81	0.94643128	102.9898	0.346	0.6924242	ns
82	-0.76803959	109.42507	0.444	0.60533333	ns	82	0.86471385	102.10814	0.389	0.6924242	ns

83	-0.75545972	107.66615	0.452	0.60533333	ns	83	0.77591549	101.13392	0.44	0.6924242	ns
84	-0.72870516	106.43183	0.468	0.61538462	ns	84	0.67286818	100.07261	0.503	0.7083333	ns
85	-0.70803092	105.93398	0.48	0.61538462	ns	85	0.57422003	98.93099	0.567	0.7269231	ns
86	-0.45431292	102.24555	0.651	0.76588235	ns	86	0.75273856	95.57959	0.453	0.6924242	ns
87	-0.82842324	106.10654	0.409	0.60533333	ns	87	0.40269728	98.45458	0.688	0.805814	ns
88	-0.99346313	105.85782	0.323	0.59814815	ns	88	0.31022404	99.86982	0.757	0.8384615	ns
89	-1.20183428	105.11923	0.232	0.52444444	ns	89	0.20833287	102.38844	0.835	0.9031915	ns
90	-1.405363	104.10524	0.163	0.4047619	ns	90	0.07363493	105.61996	0.941	0.9505051	ns
91	-1.59011077	103.20048	0.115	0.31081081	ns	91	-0.10535535	109.07587	0.916	0.9469388	ns
92	-1.7569664	102.91459	0.0819	0.24818182	ns	92	-0.33904354	111.66559	0.735	0.8384615	ns
93	-1.90060914	103.50502	0.0601	0.21464286	ns	93	-0.62180612	112.35473	0.535	0.7157895	ns
94	-2.01527371	104.90729	0.0464	0.21464286	ns	94	-0.87301265	111.23811	0.385	0.6924242	ns
95	-2.09251766	106.67739	0.0388	0.21464286	ns	95	-1.06995238	108.81308	0.287	0.6924242	ns
96	-2.11668403	108.38575	0.0366	0.21464286	ns	96	-1.22547374	106.04248	0.223	0.6924242	ns
97	-2.0690303	109.76674	0.0409	0.21464286	ns	97	-1.34692627	104.2316	0.181	0.6703704	ns
98	-1.98255851	110.81656	0.0499	0.21464286	ns	98	-1.44887298	103.49749	0.15	0.5807692	ns
99	-1.92687437	111.17935	0.0565	0.21464286	ns	99	-1.52063328	103.3231	0.131	0.5695652	ns
100	-1.94257599	110.86263	0.0546	0.21464286	ns	100	-1.53413232	102.69531	0.128	0.5695652	ns
5	statistic	df	p	p.adj	p.adj.signif	6	statistic	df	p	p.adj	p.adj.signif
1	-0.662592394	105.12095	0.509	0.999	ns	1	-0.733072376	112.07688	0.465	0.9908046	ns
2	-0.283427194	100.16991	0.777	0.999	ns	2	-0.601039083	111.89008	0.549	0.9908046	ns
3	0.000796666	98.51949	0.999	0.999	ns	3	-0.404073984	112.3344	0.687	0.9908046	ns
4	0.109322875	99.18659	0.913	0.999	ns	4	-0.149317792	114.91173	0.882	0.9978723	ns
5	-0.071221029	99.62326	0.943	0.999	ns	5	-0.122894946	115.78336	0.902	0.9978723	ns
6	-0.321457631	101.4162	0.749	0.999	ns	6	-0.231607705	112.93587	0.817	0.9908046	ns

7	-0.456905688	102.03581	0.649	0.999	ns	7	-0.309819163	111.18238	0.757	0.9908046	ns
8	-0.651386101	102.47373	0.516	0.999	ns	8	-0.396393629	108.16366	0.693	0.9908046	ns
9	-0.869467853	106.281	0.387	0.999	ns	9	-0.3478345	104.86432	0.729	0.9908046	ns
10	-0.963121652	112.03777	0.338	0.999	ns	10	-0.262043037	103.527	0.794	0.9908046	ns
11	-1.04932513	115.27621	0.296	0.999	ns	11	-0.20182797	103.32329	0.84	0.9908046	ns
12	-1.097978748	115.38236	0.274	0.999	ns	12	-0.24917608	105.07746	0.804	0.9908046	ns
13	-1.049291622	114.04592	0.296	0.999	ns	13	-0.369228128	110.17697	0.713	0.9908046	ns
14	-1.000634812	111.65533	0.319	0.999	ns	14	-0.434936076	115.19366	0.664	0.9908046	ns
15	-1.011338424	108.27913	0.314	0.999	ns	15	-0.450911432	118.02376	0.653	0.9908046	ns
16	-1.028485127	103.9873	0.306	0.999	ns	16	-0.313725046	116.47488	0.754	0.9908046	ns
17	-0.945383823	100.12707	0.347	0.999	ns	17	-0.200601943	111.06686	0.841	0.9908046	ns
18	-0.768430802	98.80506	0.444	0.999	ns	18	-0.04536993	106.28988	0.964	0.999	ns
19	-0.555116126	99.86485	0.58	0.999	ns	19	0.122019323	100.94105	0.903	0.9978723	ns
20	-0.285771733	101.48325	0.776	0.999	ns	20	0.263177689	95.99057	0.793	0.9908046	ns
21	-0.015452716	103.15175	0.988	0.999	ns	21	0.281343572	94.35072	0.779	0.9908046	ns
22	0.099598452	105.11273	0.921	0.999	ns	22	0.264012684	96.72453	0.792	0.9908046	ns
23	0.033622224	104.63719	0.973	0.999	ns	23	0.324662977	101.73055	0.746	0.9908046	ns
24	0.05960189	104.35511	0.953	0.999	ns	24	0.470426012	107.27095	0.639	0.9908046	ns
25	0.206148304	106.16917	0.837	0.999	ns	25	0.62325801	111.45856	0.534	0.9908046	ns
26	0.417204804	107.80566	0.677	0.999	ns	26	0.810453814	115.73188	0.419	0.9908046	ns
27	0.531123411	106.91089	0.596	0.999	ns	27	1.010009495	118.96987	0.315	0.8631579	ns
28	0.507910219	105.6691	0.613	0.999	ns	28	1.246085093	120.75609	0.215	0.7962963	ns
29	0.358843895	103.51784	0.72	0.999	ns	29	1.509957885	121.69032	0.134	0.5833333	ns
30	0.246015783	102.52106	0.806	0.999	ns	30	1.795699246	121.19039	0.075	0.46875	ns
31	0.225860469	104.72391	0.822	0.999	ns	31	2.028129033	119.37671	0.0448	0.4378571	ns

32	0.271228385	107.73274	0.787	0.999	ns	32	2.047386237	116.20623	0.0429	0.4378571	ns
33	0.433770128	108.29392	0.665	0.999	ns	33	1.897902114	113.16281	0.0603	0.4378571	ns
34	0.846290084	111.58385	0.399	0.999	ns	34	1.65605667	110.00835	0.101	0.505	ns
35	1.236005634	115.28361	0.219	0.999	ns	35	1.557768314	106.6914	0.122	0.5809524	ns
36	1.44967889	116.70013	0.15	0.999	ns	36	1.672848621	102.16432	0.0974	0.505	ns
37	1.546581832	118.04454	0.125	0.999	ns	37	1.949260988	99.09874	0.0541	0.4378571	ns
38	1.654656138	121.70904	0.101	0.999	ns	38	2.224502898	100.05348	0.0284	0.4057143	ns
39	1.685850953	124.55242	0.0943	0.999	ns	39	2.497897051	105.708	0.014	0.28	ns
40	1.63062444	125.18141	0.105	0.999	ns	40	2.619894389	110.89889	0.01	0.28	ns
41	1.548081972	124.17467	0.124	0.999	ns	41	2.662342244	113.41153	0.00889	0.28	ns
42	1.51315556	123.18609	0.133	0.999	ns	42	2.673768491	116.59379	0.00858	0.28	ns
43	1.323976342	122.00818	0.188	0.999	ns	43	2.537985569	120.58642	0.0124	0.28	ns
44	1.042981386	122.16387	0.299	0.999	ns	44	2.286372628	122.84929	0.0239	0.3983333	ns
45	0.573721321	123.00859	0.567	0.999	ns	45	2.101288251	124.211	0.0376	0.4378571	ns
46	0.005206716	121.6313	0.996	0.999	ns	46	1.99469149	125.01534	0.0483	0.4378571	ns
47	-0.40372977	115.40123	0.687	0.999	ns	47	1.823847916	123.94255	0.0706	0.46875	ns
48	-0.474521352	111.06048	0.636	0.999	ns	48	1.651416356	121.09866	0.101	0.505	ns
49	-0.2534948	112.2649	0.8	0.999	ns	49	1.484977841	118.31398	0.14	0.5833333	ns
50	0.087531074	116.95981	0.93	0.999	ns	50	1.259744805	117.4011	0.21	0.7962963	ns
51	0.443068033	122.33666	0.658	0.999	ns	51	1.009877356	116.62496	0.315	0.8631579	ns
52	0.710059968	125.82114	0.479	0.999	ns	52	0.796911654	114.82656	0.427	0.9908046	ns
53	0.909870973	127.20071	0.365	0.999	ns	53	0.695462751	113.87963	0.488	0.9908046	ns
54	1.162463601	127.60703	0.247	0.999	ns	54	0.741725375	114.49254	0.46	0.9908046	ns
55	1.469842331	127.43415	0.144	0.999	ns	55	0.757915043	115.02652	0.45	0.9908046	ns
56	1.784370546	126.13801	0.0768	0.999	ns	56	0.581171381	112.88893	0.562	0.9908046	ns

57	2.003533516	124.20214	0.0473	0.999	ns	57	0.276505025	108.52432	0.783	0.9908046	ns
58	2.04400564	121.97642	0.0431	0.999	ns	58	-0.036254453	103.02713	0.971	0.999	ns
59	1.912819923	120.16428	0.0582	0.999	ns	59	-0.251591242	98.34913	0.802	0.9908046	ns
60	1.614625196	119.43339	0.109	0.999	ns	60	-0.328110739	95.83225	0.744	0.9908046	ns
61	1.198118254	119.20227	0.233	0.999	ns	61	-0.312726226	95.42521	0.755	0.9908046	ns
62	0.732675234	119.86581	0.465	0.999	ns	62	-0.253353013	96.07089	0.801	0.9908046	ns
63	0.327695715	120.533	0.744	0.999	ns	63	-0.174654834	96.291	0.862	0.9908046	ns
64	0.073950201	119.54607	0.941	0.999	ns	64	-0.078219264	96.79175	0.938	0.9978723	ns
65	-0.06730427	117.92646	0.946	0.999	ns	65	-0.034245468	96.86026	0.973	0.999	ns
66	-0.043896979	115.7436	0.965	0.999	ns	66	0.001744659	97.6781	0.999	0.999	ns
67	0.121378937	114.31317	0.904	0.999	ns	67	0.101275898	98.59928	0.92	0.9978723	ns
68	0.354501165	113.13526	0.724	0.999	ns	68	0.199565359	99.3541	0.842	0.9908046	ns
69	0.537265131	111.17907	0.592	0.999	ns	69	0.333788041	100.77722	0.739	0.9908046	ns
70	0.691950177	110.97124	0.49	0.999	ns	70	0.455642427	101.46655	0.65	0.9908046	ns
71	0.800843441	113.95429	0.425	0.999	ns	71	0.519111407	100.30171	0.605	0.9908046	ns
72	0.908172101	118.15557	0.366	0.999	ns	72	0.443639593	98.75162	0.658	0.9908046	ns
73	0.934731951	118.42946	0.352	0.999	ns	73	0.214050144	97.62195	0.831	0.9908046	ns
74	0.852171259	114.40016	0.396	0.999	ns	74	-0.079574129	96.83477	0.937	0.9978723	ns
75	0.649693284	106.93176	0.517	0.999	ns	75	-0.355896748	96.60949	0.723	0.9908046	ns
76	0.469912876	102.80525	0.639	0.999	ns	76	-0.618670312	97.93468	0.538	0.9908046	ns
77	0.417614385	103.07401	0.677	0.999	ns	77	-0.883266182	99.25346	0.379	0.9717949	ns
78	0.405577931	107.06353	0.686	0.999	ns	78	-0.997287729	99.59598	0.321	0.8631579	ns
79	0.453719085	112.27766	0.651	0.999	ns	79	-0.805793226	101.94767	0.422	0.9908046	ns
80	0.515126646	116.91326	0.607	0.999	ns	80	-0.484141647	103.7183	0.629	0.9908046	ns
81	0.550515821	117.0217	0.583	0.999	ns	81	-0.17965578	105.57867	0.858	0.9908046	ns

82	0.442408928	112.77217	0.659	0.999	ns	82	0.013473328	106.00419	0.989	0.999	ns
83	0.307516995	106.31652	0.759	0.999	ns	83	0.083761732	104.80781	0.933	0.9978723	ns
84	0.231193699	100.61904	0.818	0.999	ns	84	-0.006370376	104.40408	0.995	0.999	ns
85	0.124289568	96.50223	0.901	0.999	ns	85	-0.200819472	104.30998	0.841	0.9908046	ns
86	0.157179801	94.30683	0.875	0.999	ns	86	-0.183591799	102.52208	0.855	0.9908046	ns
87	-0.138546177	97.65913	0.89	0.999	ns	87	-0.632507689	99.42057	0.529	0.9908046	ns
88	-0.144664796	98.66048	0.885	0.999	ns	88	-0.827186846	95.84668	0.41	0.9908046	ns
89	-0.10037694	99.12246	0.92	0.999	ns	89	-0.9839088	93.88754	0.328	0.8631579	ns
90	-0.099815199	97.91944	0.921	0.999	ns	90	-1.066750211	93.98443	0.289	0.8631579	ns
91	-0.149381998	96.81688	0.882	0.999	ns	91	-1.04527703	95.41474	0.299	0.8631579	ns
92	-0.234250867	99.18008	0.815	0.999	ns	92	-1.004173866	98.50815	0.318	0.8631579	ns
93	-0.361930885	102.65344	0.718	0.999	ns	93	-1.005023537	101.53997	0.317	0.8631579	ns
94	-0.414467462	105.09623	0.679	0.999	ns	94	-1.024256984	105.52755	0.308	0.8631579	ns
95	-0.352975332	106.85468	0.725	0.999	ns	95	-1.057435874	110.23327	0.293	0.8631579	ns
96	-0.361592853	106.34331	0.718	0.999	ns	96	-1.12667207	113.88224	0.262	0.8631579	ns
97	-0.388895098	104.14489	0.698	0.999	ns	97	-1.301420393	114.50233	0.196	0.784	ns
98	-0.359483028	103.63275	0.72	0.999	ns	98	-1.502491563	112.45632	0.136	0.5833333	ns
99	-0.27088046	105.42448	0.787	0.999	ns	99	-1.742446858	109.70935	0.0842	0.4952941	ns
100	-0.140609534	107.6478	0.888	0.999	ns	100	-1.891780586	105.72333	0.0613	0.4378571	ns
7	statistic	df	p	p.adj	p.adj.signif	8	statistic	df	p	p.adj	p.adj.signif
1	-3.97163521	121.37631	1.21E-04	0.000636842	***	1	-1.61077068	97.06867	0.11	0.5	ns
2	-3.972423196	122.35817	1.21E-04	0.000636842	***	2	-1.49700565	95.32526	0.138	0.5	ns
3	-3.910550744	123.48082	1.51E-04	0.000690909	***	3	-1.49739978	95.29232	0.138	0.5	ns
4	-3.832367008	124.65694	2.00E-04	0.000740741	***	4	-1.54185798	96.71795	0.126	0.5	ns
5	-3.779495006	125.83188	2.41E-04	0.000831035	***	5	-1.57538802	98.78533	0.118	0.5	ns

6	-3.792674428	126.84323	2.30E-04	0.000821429	***	6	-1.57604767	99.90764	0.118	0.5	ns
7	-3.842146881	127.52334	1.91E-04	0.000734615	***	7	-1.56137215	99.69085	0.122	0.5	ns
8	-3.86415456	127.84073	1.76E-04	0.000704	***	8	-1.54719084	98.66653	0.125	0.5	ns
9	-3.877452625	127.93479	1.68E-04	0.0007	***	9	-1.54458116	98.03682	0.126	0.5	ns
10	-3.884468408	127.91973	1.64E-04	0.0007	***	10	-1.55867577	98.40772	0.122	0.5	ns
11	-3.913661195	127.77996	1.47E-04	0.000690909	***	11	-1.57999942	98.86933	0.117	0.5	ns
12	-3.982742822	127.48619	1.14E-04	0.000636842	***	12	-1.5885756	99.03073	0.115	0.5	ns
13	-4.11779153	127.29434	6.83E-05	0.0004275	***	13	-1.60486387	99.62887	0.112	0.5	ns
14	-4.256938004	127.27385	3.99E-05	0.0004275	***	14	-1.6134694	101.06877	0.11	0.5	ns
15	-4.345135842	127.261	2.82E-05	0.0004275	***	15	-1.57090574	102.54179	0.119	0.5	ns
16	-4.343086473	127.06547	2.84E-05	0.0004275	***	16	-1.48742822	104.01702	0.14	0.5	ns
17	-4.288225732	126.87365	3.54E-05	0.0004275	***	17	-1.39024546	105.54419	0.167	0.53125	ns
18	-4.221191997	126.75496	4.60E-05	0.0004275	***	18	-1.34412911	107.72063	0.182	0.5459459	ns
19	-4.133917086	126.44497	6.45E-05	0.0004275	***	19	-1.30492715	109.37155	0.195	0.5459459	ns
20	-4.118922053	126.05259	6.84E-05	0.0004275	***	20	-1.22763975	109.53471	0.222	0.5560976	ns
21	-4.15049492	125.78704	6.07E-05	0.0004275	***	21	-1.07822802	108.09587	0.283	0.6148936	ns
22	-4.188781524	125.9016	5.23E-05	0.0004275	***	22	-0.8981771	106.53752	0.371	0.687037	ns
23	-4.255404236	126.09138	4.04E-05	0.0004275	***	23	-0.75296195	104.84023	0.453	0.7289855	ns
24	-4.337895666	126.37229	2.91E-05	0.0004275	***	24	-0.692779	102.90383	0.49	0.7289855	ns
25	-4.425534095	126.87103	2.05E-05	0.0004275	***	25	-0.70459037	101.53309	0.483	0.7289855	ns
26	-4.47980977	127.62502	1.64E-05	0.0004275	***	26	-0.79434146	102.22221	0.429	0.7289855	ns
27	-4.410431379	127.98443	2.16E-05	0.0004275	***	27	-0.93257692	103.59532	0.353	0.687037	ns
28	-4.193538619	127.9749	5.09E-05	0.0004275	***	28	-1.12453629	103.97102	0.263	0.5844444	ns
29	-3.905132799	127.98888	1.52E-04	0.000690909	***	29	-1.32637201	102.98108	0.188	0.5459459	ns
30	-3.618954131	127.94972	4.25E-04	0.001416667	**	30	-1.50596361	100.77392	0.135	0.5	ns

31	-3.333018823	127.67564	1.12E-03	0.003111111	**	31	-1.62316734	98.33376	0.108	0.5	ns
32	-3.083859886	127.16525	2.51E-03	0.006121951	**	32	-1.68924316	95.86527	0.0944	0.5	ns
33	-2.922479817	126.33591	4.12E-03	0.009155556	**	33	-1.68700749	94.03623	0.0949	0.5	ns
34	-2.888376758	125.15483	4.56E-03	0.009913044	**	34	-1.67305843	93.35409	0.0977	0.5	ns
35	-2.948327432	124.1542	3.82E-03	0.008681818	**	35	-1.65990586	93.17893	0.1	0.5	ns
36	-3.05007608	123.46159	2.80E-03	0.006666667	**	36	-1.58294628	92.36092	0.117	0.5	ns
37	-3.1723974	122.45393	1.91E-03	0.004897436	**	37	-1.44468284	90.93594	0.152	0.5066667	ns
38	-3.313077605	120.87627	1.22E-03	0.003297297	**	38	-1.28445359	89.19712	0.202	0.5459459	ns
39	-3.438482571	118.48673	8.08E-04	0.002376471	**	39	-1.15216512	87.92796	0.252	0.5795455	ns
40	-3.542538224	114.36602	5.75E-04	0.001796875	**	40	-1.14558626	88.13002	0.255	0.5795455	ns
41	-3.549336527	108.69464	5.72E-04	0.001796875	**	41	-1.233811	90.98252	0.22	0.5560976	ns
42	-3.465029752	103.26814	7.73E-04	0.002342424	**	42	-1.38339848	95.12696	0.17	0.53125	ns
43	-3.371632854	99.79879	1.06E-03	0.003028571	**	43	-1.55262223	98.64999	0.124	0.5	ns
44	-3.29184673	97.99342	1.38E-03	0.003631579	**	44	-1.65863444	101.05967	0.1	0.5	ns
45	-3.175771352	98.70742	1.99E-03	0.004975	**	45	-1.67172605	103.43993	0.0976	0.5	ns
46	-3.00809489	99.74444	3.33E-03	0.007744186	**	46	-1.6348837	105.65431	0.105	0.5	ns
47	-2.7772256	101.33984	6.53E-03	0.013893617	*	47	-1.58966986	107.09489	0.115	0.5	ns
48	-2.528298709	103.01173	1.30E-02	0.027083333	*	48	-1.46747137	107.63228	0.145	0.5	ns
49	-2.259214133	104.60393	2.59E-02	0.052857143	ns	49	-1.29694885	107.28647	0.197	0.5459459	ns
50	-2.033788381	105.39858	4.45E-02	0.089	ns	50	-1.22259873	107.72764	0.224	0.5560976	ns
51	-1.883734882	105.2614	6.24E-02	0.105932203	ns	51	-1.21169449	108.43842	0.228	0.5560976	ns
52	-1.835377572	104.03625	6.93E-02	0.113606557	ns	52	-1.16603975	108.41273	0.246	0.5795455	ns
53	-1.882861834	103.75596	6.25E-02	0.105932203	ns	53	-1.06450685	108.53665	0.289	0.6148936	ns
54	-1.939891527	105.24172	5.51E-02	0.098392857	ns	54	-0.92652169	109.03032	0.356	0.687037	ns
55	-1.980047943	107.04627	5.03E-02	0.092181818	ns	55	-0.76570722	110.64772	0.445	0.7289855	ns

56	-2.013868225	108.55757	4.65E-02	0.089423077	ns	56	-0.60393827	113.25262	0.547	0.7493151	ns
57	-2.021925012	109.42802	4.56E-02	0.089411765	ns	57	-0.46119172	116.46666	0.646	0.8125	ns
58	-2.001903151	109.92602	4.78E-02	0.090188679	ns	58	-0.32828102	119.44907	0.743	0.8674157	ns
59	-1.975371195	109.89381	5.07E-02	0.092181818	ns	59	-0.2316868	121.87474	0.817	0.893617	ns
60	-1.915303476	109.44813	5.81E-02	0.101929825	ns	60	-0.20262324	123.95179	0.84	0.893617	ns
61	-1.847611478	108.71915	6.74E-02	0.112333333	ns	61	-0.22270759	125.49523	0.824	0.893617	ns
62	-1.770819125	107.74309	7.94E-02	0.128064516	ns	62	-0.29199649	126.29546	0.771	0.8674157	ns
63	-1.686876805	106.29474	9.46E-02	0.15015873	ns	63	-0.40178249	126.5197	0.689	0.8402439	ns
64	-1.594071924	104.44768	1.14E-01	0.178125	ns	64	-0.511731	126.22854	0.61	0.8025641	ns
65	-1.443559606	102.73671	1.52E-01	0.233846154	ns	65	-0.64308861	125.35552	0.521	0.7338028	ns
66	-1.25500309	101.31015	2.12E-01	0.321212121	ns	66	-0.73453141	124.38242	0.464	0.7289855	ns
67	-1.010934349	99.97765	3.14E-01	0.468656716	ns	67	-0.76046926	123.5578	0.448	0.7289855	ns
68	-0.743499546	97.99241	4.59E-01	0.66	ns	68	-0.7219389	122.45142	0.472	0.7289855	ns
69	-0.52818908	95.22274	5.99E-01	0.831944444	ns	69	-0.62532018	121.44711	0.533	0.7402778	ns
70	-0.378281944	93.2266	7.06E-01	0.932098765	ns	70	-0.48945369	121.01379	0.625	0.8025641	ns
71	-0.273840887	92.58822	7.85E-01	0.934117647	ns	71	-0.31649932	120.39486	0.752	0.8674157	ns
72	-0.181346396	92.68888	8.56E-01	0.952747253	ns	72	-0.12235971	119.50218	0.903	0.9309278	ns
73	-0.049935781	92.91519	9.60E-01	0.979591837	ns	73	0.08316873	118.37208	0.934	0.9530612	ns
74	0.091516038	93.29618	9.27E-01	0.979591837	ns	74	0.29199514	117.00762	0.771	0.8674157	ns
75	0.218680626	93.44632	8.27E-01	0.952747253	ns	75	0.48866242	114.6273	0.626	0.8025641	ns
76	0.313191757	93.47885	7.55E-01	0.932098765	ns	76	0.67805149	111.75267	0.499	0.7289855	ns
77	0.35510392	94.13561	7.23E-01	0.932098765	ns	77	0.81413407	109.16184	0.417	0.7289855	ns
78	0.362767183	95.39783	7.18E-01	0.932098765	ns	78	0.9077954	107.08506	0.366	0.687037	ns
79	0.350918252	96.87533	7.26E-01	0.932098765	ns	79	0.94894522	105.19403	0.345	0.687037	ns
80	0.31848495	99.07506	7.51E-01	0.932098765	ns	80	0.94010072	103.7383	0.349	0.687037	ns

81	0.261864809	100.90548	7.94E-01	0.934117647	ns	81	0.90228005	102.96755	0.369	0.687037	ns
82	0.169900727	102.30501	8.65E-01	0.952747253	ns	82	0.84734994	103.47137	0.399	0.7254545	ns
83	0.080576432	103.2193	9.36E-01	0.979591837	ns	83	0.76722386	104.74423	0.445	0.7289855	ns
84	0.01585191	105.06025	9.87E-01	0.993	ns	84	0.6817444	105.93711	0.497	0.7289855	ns
85	-0.008689723	107.47762	9.93E-01	0.993	ns	85	0.58064801	107.09115	0.563	0.7608108	ns
86	0.168068352	109.70057	8.67E-01	0.952747253	ns	86	0.6434645	107.02161	0.521	0.7338028	ns
87	0.072134141	110.10102	9.43E-01	0.979591837	ns	87	0.33987153	107.57507	0.735	0.8674157	ns
88	0.13494517	109.8452	8.93E-01	0.960215054	ns	88	0.22690005	107.01359	0.821	0.893617	ns
89	0.190209342	107.7057	8.50E-01	0.952747253	ns	89	0.1307378	105.93839	0.896	0.9309278	ns
90	0.265255263	102.77833	7.91E-01	0.934117647	ns	90	0.0381153	105.19415	0.97	0.97	ns
91	0.330839915	98.74356	7.41E-01	0.932098765	ns	91	-0.05783095	105.26712	0.954	0.9636364	ns
92	0.33372514	97.58004	7.39E-01	0.932098765	ns	92	-0.1344308	105.55285	0.893	0.9309278	ns
93	0.283873724	97.51614	7.77E-01	0.934117647	ns	93	-0.20873683	106.16945	0.835	0.893617	ns
94	0.192193066	96.86573	8.48E-01	0.952747253	ns	94	-0.29030961	107.48147	0.772	0.8674157	ns
95	0.053743833	95.45428	9.57E-01	0.979591837	ns	95	-0.35849579	109.29828	0.721	0.8674157	ns
96	-0.135823249	93.77634	8.92E-01	0.960215054	ns	96	-0.40453457	111.5801	0.687	0.8402439	ns
97	-0.361473482	92.45074	7.19E-01	0.932098765	ns	97	-0.4546384	114.57343	0.65	0.8125	ns
98	-0.568414946	91.70531	5.71E-01	0.804225352	ns	98	-0.54494021	117.7287	0.587	0.7826667	ns
99	-0.739065555	92.04704	4.62E-01	0.66	ns	99	-0.67174175	120.25161	0.503	0.7289855	ns
100	-0.886311541	93.49012	3.78E-01	0.555882353	ns	100	-0.81108063	121.75286	0.419	0.7289855	ns
9	statistic	df	p	p.adj	p.adj.signif	10	statistic	df	p	p.adj	p.adj.signif
1	0.632983601	96.49888	0.528	0.9958763	ns	1	-0.09061399	89.87691	0.928	0.95	ns
2	1.01941917	98.22083	0.311	0.9352941	ns	2	-0.08855832	92.21354	0.93	0.95	ns
3	1.297875105	99.93675	0.197	0.8444444	ns	3	-0.13805795	96.01065	0.89	0.94680851	ns
4	1.003079057	99.10174	0.318	0.9352941	ns	4	-0.32317848	98.88799	0.747	0.87882353	ns

5	0.305225845	94.26788	0.761	0.9958763	ns
6	-0.225698907	89.0676	0.822	0.9958763	ns
7	-0.46232548	84.3943	0.645	0.9958763	ns
8	-0.285176603	83.1086	0.776	0.9958763	ns
9	0.043156269	84.08566	0.966	0.9958763	ns
10	0.46352388	86.92081	0.644	0.9958763	ns
11	0.777406728	89.43434	0.439	0.984	ns
12	0.761353901	91.69551	0.448	0.984	ns
13	0.611613255	92.9783	0.542	0.9958763	ns
14	0.522783402	92.99203	0.602	0.9958763	ns
15	0.551669285	91.0859	0.583	0.9958763	ns
16	0.609719964	86.77449	0.544	0.9958763	ns
17	0.53986355	83.67231	0.591	0.9958763	ns
18	0.283518814	81.49664	0.777	0.9958763	ns
19	0.159938531	84.51412	0.873	0.9958763	ns
20	0.113300298	90.31689	0.91	0.9958763	ns
21	-0.01971081	94.55568	0.984	0.997	ns
22	-0.065573907	101.36228	0.948	0.9958763	ns
23	-0.126698682	100.38305	0.899	0.9958763	ns
24	-0.124636821	97.97061	0.901	0.9958763	ns
25	-0.105278667	97.60629	0.916	0.9958763	ns
26	-0.193858639	98.91905	0.847	0.9958763	ns
27	-0.320433874	97.45441	0.749	0.9958763	ns
28	-0.223553017	101.44345	0.824	0.9958763	ns
29	-0.210279928	104.25033	0.834	0.9958763	ns

5	-0.66766442	99.51811	0.506	0.76268657	ns
6	-1.11327295	99.1885	0.268	0.48727273	ns
7	-1.4232808	97.17351	0.158	0.3375	ns
8	-1.51238662	94.87576	0.134	0.32682927	ns
9	-1.52507619	94.20825	0.131	0.32682927	ns
10	-1.61533317	94.12079	0.11	0.28947368	ns
11	-1.7707312	93.79008	0.0799	0.2496875	ns
12	-1.91653929	93.35117	0.0584	0.20857143	ns
13	-2.01105435	92.04048	0.0472	0.19666667	ns
14	-2.05912377	90.20967	0.0424	0.18826087	ns
15	-2.07861205	88.43081	0.0406	0.18826087	ns
16	-2.05021177	87.27209	0.0433	0.18826087	ns
17	-1.92867002	86.55988	0.057	0.20857143	ns
18	-1.67430569	85.83772	0.0977	0.27914286	ns
19	-1.30607419	85.34501	0.195	0.38235294	ns
20	-0.93446186	84.8663	0.353	0.59830508	ns
21	-0.65960179	84.39148	0.511	0.76268657	ns
22	-0.52508258	84.4796	0.601	0.79220779	ns
23	-0.51126048	85.46365	0.61	0.79220779	ns
24	-0.57354153	86.74554	0.568	0.77972973	ns
25	-0.63030983	88.35788	0.53	0.77941176	ns
26	-0.59349584	90.79851	0.554	0.77972973	ns
27	-0.45357751	94.31122	0.651	0.82405063	ns
28	-0.25419545	98.30485	0.8	0.8988764	ns
29	0.08636663	101.68652	0.931	0.95	ns

30	-0.305752376	104.99031	0.76	0.9958763	ns	30	0.61224496	105.44685	0.542	0.77972973	ns
31	-0.142965564	109.24525	0.887	0.9958763	ns	31	1.20499294	109.38235	0.231	0.44423077	ns
32	-0.043558806	111.95939	0.965	0.9958763	ns	32	1.68126487	112.68839	0.0955	0.27914286	ns
33	-0.0700602	113.74068	0.944	0.9958763	ns	33	1.93160199	114.10196	0.0559	0.20857143	ns
34	-0.291220094	110.88261	0.771	0.9958763	ns	34	1.80907223	112.00436	0.0731	0.24366667	ns
35	-0.283230342	108.9879	0.778	0.9958763	ns	35	1.48646023	108.12381	0.14	0.33333333	ns
36	-0.499965683	109.69266	0.618	0.9958763	ns	36	1.16795457	103.59616	0.246	0.45555556	ns
37	-0.524259058	109.40887	0.601	0.9958763	ns	37	0.95523007	98.83375	0.342	0.58965517	ns
38	-0.565998579	107.26375	0.573	0.9958763	ns	38	0.75967586	94.56163	0.449	0.71428571	ns
39	-0.866770221	100.68051	0.388	0.984	ns	39	0.55914589	92.09573	0.577	0.77972973	ns
40	-1.086641021	96.09679	0.28	0.9032258	ns	40	0.38585608	91.22649	0.701	0.85487805	ns
41	-1.591042504	94.9061	0.115	0.7666667	ns	41	0.27917923	89.0415	0.781	0.8988764	ns
42	-1.91360561	99.05325	0.0586	0.5554545	ns	42	0.26484483	85.06354	0.792	0.8988764	ns
43	-2.030754057	104.2696	0.0448	0.5554545	ns	43	0.35400328	81.57617	0.724	0.87228916	ns
44	-1.893753882	102.99858	0.0611	0.5554545	ns	44	0.49333799	80.14237	0.623	0.79871795	ns
45	-1.648140665	102.59719	0.102	0.75	ns	45	0.66303987	80.18889	0.509	0.76268657	ns
46	-1.482031781	105.80452	0.141	0.8055556	ns	46	0.96448188	81.23964	0.338	0.58965517	ns
47	-1.427115859	102.76044	0.157	0.8263158	ns	47	1.36127884	82.03574	0.177	0.356	ns
48	-1.287973704	101.31034	0.201	0.8444444	ns	48	1.79232664	81.91166	0.0768	0.24774194	ns
49	-1.213782862	101.70947	0.228	0.8444444	ns	49	2.09601435	81.97541	0.0392	0.18826087	ns
50	-1.141944527	100.88668	0.256	0.8827586	ns	50	2.17256606	82.50889	0.0327	0.18166667	ns
51	-1.255946798	93.7683	0.212	0.8444444	ns	51	1.98620418	80.71583	0.0504	0.2016	ns
52	-1.254327141	88.40333	0.213	0.8444444	ns	52	1.75174896	78.9618	0.0837	0.25363636	ns
53	-1.282346082	88.96972	0.203	0.8444444	ns	53	1.64291095	78.69868	0.104	0.28108108	ns
54	-1.285947418	91.62964	0.202	0.8444444	ns	54	1.35979313	75.06292	0.178	0.356	ns

55	-0.906058207	92.48193	0.367	0.984	ns	55	0.97671339	72.42675	0.332	0.58965517	ns
56	-0.817132026	91.91702	0.416	0.984	ns	56	0.56700049	72.47291	0.572	0.77972973	ns
57	-0.726955273	87.85239	0.469	0.984	ns	57	0.1613529	73.63324	0.872	0.93763441	ns
58	-0.642891219	83.32465	0.522	0.9958763	ns	58	-0.20005797	73.82499	0.842	0.93186813	ns
59	-0.47435939	84.9013	0.636	0.9958763	ns	59	-0.51190183	74.05614	0.61	0.79220779	ns
60	-0.306964982	93.35548	0.76	0.9958763	ns	60	-0.75921763	76.39735	0.45	0.71428571	ns
61	-0.126353758	103.14235	0.9	0.9958763	ns	61	-0.85669437	79.55419	0.394	0.65666667	ns
62	-0.084049204	103.69087	0.933	0.9958763	ns	62	-0.73523996	82.06057	0.464	0.725	ns
63	0.06872879	108.093	0.945	0.9958763	ns	63	-0.57645703	84.23718	0.566	0.77972973	ns
64	0.092035497	108.33727	0.927	0.9958763	ns	64	-0.43712582	86.09853	0.663	0.82875	ns
65	-0.003853691	108.98865	0.997	0.997	ns	65	-0.33998344	88.2391	0.735	0.875	ns
66	0.138406479	111.68345	0.89	0.9958763	ns	66	-0.25417789	89.98905	0.8	0.8988764	ns
67	0.265634932	115.95809	0.791	0.9958763	ns	67	-0.1799189	90.56592	0.858	0.9326087	ns
68	0.404596819	118.47368	0.687	0.9958763	ns	68	-0.08968624	91.70502	0.929	0.95	ns
69	0.691080101	117.31275	0.491	0.984	ns	69	-0.02750972	94.19161	0.978	0.978	ns
70	0.87619631	114.25526	0.383	0.984	ns	70	-0.05785125	95.96166	0.954	0.96363636	ns
71	1.010310344	114.57556	0.314	0.9352941	ns	71	-0.19251449	95.32108	0.848	0.93186813	ns
72	0.925515293	112.80437	0.357	0.984	ns	72	-0.42408789	93.56691	0.672	0.82962963	ns
73	0.733578606	113.17453	0.465	0.984	ns	73	-0.78305256	90.28615	0.436	0.71428571	ns
74	0.690011425	115.89155	0.492	0.984	ns	74	-1.17110002	85.90229	0.245	0.45555556	ns
75	0.332147241	112.67921	0.74	0.9958763	ns	75	-1.40933919	83.36001	0.162	0.3375	ns
76	-0.183544703	112.73281	0.855	0.9958763	ns	76	-1.44091878	84.54118	0.153	0.3375	ns
77	-0.529455641	116.46985	0.597	0.9958763	ns	77	-1.42186866	87.76804	0.159	0.3375	ns
78	-0.762958355	123.02214	0.447	0.984	ns	78	-1.42981744	91.7898	0.156	0.3375	ns
79	-0.696487714	127.12085	0.487	0.984	ns	79	-1.46694985	94.95388	0.146	0.3375	ns

80	-0.372321905	127.78915	0.71	0.9958763	ns	80	-1.53466339	96.39816	0.128	0.32682927	ns
81	0.00646665	127.88613	0.995	0.997	ns	81	-1.64370035	96.49736	0.103	0.28108108	ns
82	0.314763042	127.77821	0.753	0.9958763	ns	82	-1.82638836	96.40038	0.0709	0.24366667	ns
83	0.707845611	127.2788	0.48	0.984	ns	83	-2.07676608	96.50385	0.0405	0.18826087	ns
84	1.107519944	126.7399	0.27	0.9	ns	84	-2.41660045	95.77254	0.0176	0.12571429	ns
85	1.230111901	126.53249	0.221	0.8444444	ns	85	-2.78079903	93.86651	0.00655	0.0749	ns
86	1.466694237	123.46838	0.145	0.8055556	ns	86	-2.73967719	89.57241	0.00742	0.0749	ns
87	1.631627476	122.7164	0.105	0.75	ns	87	-3.27713695	91.83095	0.00148	0.04933333	*
88	1.995892633	121.27143	0.0482	0.5554545	ns	88	-3.34614068	93.54701	0.00118	0.04933333	*
89	2.360234837	120.11662	0.0199	0.4975	ns	89	-3.27558494	96.63309	0.00146	0.04933333	*
90	2.619030115	118.86387	0.00997	0.4975	ns	90	-3.10942879	100.37699	0.00244	0.0558	ns
91	2.611140053	117.8876	0.0102	0.4975	ns	91	-2.9171004	102.32244	0.00434	0.06557143	ns
92	2.393586226	117.60589	0.0183	0.4975	ns	92	-2.72890911	101.53981	0.00749	0.0749	ns
93	2.25292152	118.11387	0.0261	0.522	ns	93	-2.5243391	100.02275	0.0132	0.11	ns
94	2.130798262	118.13122	0.0352	0.5554545	ns	94	-2.32754118	97.92504	0.022	0.14666667	ns
95	1.915698059	118.65744	0.0578	0.5554545	ns	95	-2.21471767	94.64584	0.0292	0.17176471	ns
96	1.726699012	117.18695	0.0869	0.7241667	ns	96	-2.25688901	91.05209	0.0264	0.165	ns
97	1.517166021	117.48373	0.132	0.8055556	ns	97	-2.43331909	88.8596	0.017	0.12571429	ns
98	1.177894695	116.14769	0.241	0.8607143	ns	98	-2.68373872	88.07849	0.0087	0.07909091	ns
99	0.968120049	110.39994	0.335	0.9571429	ns	99	-2.9088109	87.93533	0.00459	0.06557143	ns
100	0.774910257	112.15763	0.44	0.984	ns	100	-3.07747647	87.34287	0.00279	0.0558	ns
11	statistic	df	p	p.adj	p.adj.signif	12	statistic	df	p	p.adj	p.adj.signif
1	3.25819253	119.97405	0.00146	0.01327273	*	1	1.58983798	125.53966	0.114	0.5181818	ns
2	3.062045885	123.93272	0.0027	0.0225	*	2	1.48494572	123.73808	0.14	0.53125	ns
3	2.942018177	125.35332	0.00389	0.02921429	*	3	1.37999825	119.31845	0.17	0.53125	ns

4	2.836381354	126.30889	0.00532	0.03325	*	4	1.27660755	115.95045	0.204	0.5439024	ns
5	2.715557657	127.05231	0.00754	0.04435294	*	5	1.22392533	115.37009	0.223	0.5439024	ns
6	2.509026902	127.34412	0.0134	0.067	ns	6	1.24493805	117.51549	0.216	0.5439024	ns
7	2.183796977	127.86632	0.0308	0.14	ns	7	1.34779448	120.72308	0.18	0.5323529	ns
8	1.890947195	127.98942	0.0609	0.23423077	ns	8	1.51743417	121.69927	0.132	0.528	ns
9	1.700747697	127.66682	0.0914	0.32642857	ns	9	1.45575144	119.16777	0.148	0.53125	ns
10	1.551096329	126.77956	0.123	0.42333333	ns	10	1.24812372	114.42296	0.215	0.5439024	ns
11	1.3596825	123.95114	0.176	0.50285714	ns	11	0.94524893	111.82763	0.347	0.6345455	ns
12	1.057620172	118.92329	0.292	0.66818182	ns	12	0.67768211	111.11536	0.499	0.7070423	ns
13	0.567604017	115.7623	0.571	0.90864198	ns	13	0.55981835	107.52202	0.577	0.7439024	ns
14	0.419126762	116.23277	0.676	0.90864198	ns	14	0.56551837	102.57747	0.573	0.7439024	ns
15	0.338336805	118.68772	0.736	0.90864198	ns	15	0.43615005	98.17196	0.664	0.7655172	ns
16	0.260820509	119.31463	0.795	0.93529412	ns	16	0.21926615	97.39514	0.827	0.8892473	ns
17	-0.007263194	117.51632	0.994	0.994	ns	17	-0.02480342	100.87699	0.98	0.98	ns
18	-0.355104961	115.23014	0.723	0.90864198	ns	18	-0.33081144	104.04841	0.741	0.8054348	ns
19	-0.647460555	113.078	0.519	0.87966102	ns	19	-0.59038074	105.18469	0.556	0.7413333	ns
20	-0.805623302	112.41059	0.422	0.82115385	ns	20	-0.81740744	101.36039	0.416	0.6666667	ns
21	-0.83487428	112.04574	0.406	0.812	ns	21	-1.15302929	95.19117	0.252	0.56	ns
22	-0.912998231	110.12045	0.363	0.77234043	ns	22	-1.70462293	92.49486	0.0916	0.4361905	ns
23	-1.073229111	107.94111	0.286	0.66818182	ns	23	-2.09087083	92.18818	0.0393	0.2211111	ns
24	-1.25710231	106.69333	0.211	0.57027027	ns	24	-2.30181546	91.58035	0.0236	0.1573333	ns
25	-1.395677583	106.85898	0.166	0.48823529	ns	25	-2.44665682	94.35138	0.0163	0.1573333	ns
26	-1.490632665	108.94896	0.139	0.434375	ns	26	-2.53884341	97.24429	0.0127	0.1573333	ns
27	-1.504825515	112.35835	0.135	0.434375	ns	27	-2.68307426	99.62176	0.00854	0.1573333	ns
28	-1.405357317	115.36909	0.163	0.48823529	ns	28	-2.69115299	102.7672	0.00831	0.1573333	ns

29	-1.190488802	116.79135	0.236	0.62105263	ns	29	-2.58440682	105.66372	0.0111	0.1573333	ns
30	-0.999120777	116.93887	0.32	0.71111111	ns	30	-2.46641985	108.90831	0.0152	0.1573333	ns
31	-0.648829266	116.38756	0.518	0.87966102	ns	31	-2.38805609	110.63497	0.0186	0.1573333	ns
32	-0.274365287	118.37684	0.784	0.93529412	ns	32	-2.33001693	112.94968	0.0216	0.1573333	ns
33	-0.096467061	121.74578	0.923	0.97263158	ns	33	-2.29480881	114.56207	0.0236	0.1573333	ns
34	-0.178391509	121.18264	0.859	0.95054945	ns	34	-2.2075223	114.78229	0.0293	0.183125	ns
35	-0.598590719	118.30123	0.551	0.90864198	ns	35	-1.92730294	115.29844	0.0564	0.282	ns
36	-1.274646279	116.54584	0.205	0.56944444	ns	36	-1.53623772	115.6311	0.127	0.528	ns
37	-2.125725653	116.54322	0.0356	0.15478261	ns	37	-1.23867166	117.7061	0.218	0.5439024	ns
38	-2.881964019	119.38969	0.00469	0.03126667	*	38	-1.01926368	117.69349	0.31	0.6326531	ns
39	-3.370936793	122.56521	0.001	0.01111111	*	39	-0.89208217	116.69991	0.374	0.6448276	ns
40	-3.563903085	122.52803	0.000522	0.0073125	**	40	-1.03619717	115.58355	0.302	0.6326531	ns
41	-3.532457651	120.26621	0.000585	0.0073125	**	41	-1.06830857	115.57117	0.288	0.626087	ns
42	-3.648797836	117.98318	0.000394	0.0073125	**	42	-0.98556465	117.45458	0.326	0.6345455	ns
43	-3.792178153	117.43789	0.000237	0.0073125	**	43	-1.17057316	115.09327	0.244	0.5545455	ns
44	-3.94428489	118.19424	0.000136	0.0073125	**	44	-1.38345269	110.91856	0.169	0.53125	ns
45	-3.858786885	118.71464	0.000186	0.0073125	**	45	-1.44497505	108.7529	0.151	0.53125	ns
46	-3.67211348	117.61848	0.000363	0.0073125	**	46	-1.30971231	108.5679	0.193	0.5439024	ns
47	-3.559384706	113.18752	0.000545	0.0073125	**	47	-1.02336959	112.19396	0.308	0.6326531	ns
48	-3.332090783	105.911	0.00119	0.0119	*	48	-0.81033104	108.78808	0.42	0.6666667	ns
49	-2.93917364	99.53446	0.00409	0.02921429	*	49	-0.674471	100.08833	0.502	0.7070423	ns
50	-2.552706963	96.36214	0.0123	0.06526316	ns	50	-0.60318145	90.06376	0.548	0.7413333	ns
51	-2.228054286	96.20987	0.0282	0.13428571	ns	51	-0.70806163	80.91003	0.481	0.6971014	ns
52	-2.052346211	97.40123	0.0428	0.17833333	ns	52	-0.89495379	76.03763	0.374	0.6448276	ns
53	-1.870845339	100.44853	0.0643	0.23814815	ns	53	-1.19061994	73.09102	0.238	0.5534884	ns

54	-1.539996543	103.10894	0.127	0.42333333	ns	54	-1.40845481	72.03484	0.163	0.53125	ns
55	-1.058915115	104.8001	0.292	0.66818182	ns	55	-1.41575941	75.38337	0.161	0.53125	ns
56	-0.530076818	106.70846	0.597	0.90864198	ns	56	-1.34869244	80.17102	0.181	0.5323529	ns
57	-0.17015146	106.36148	0.865	0.95054945	ns	57	-1.19646368	84.79076	0.235	0.5534884	ns
58	0.028410715	106.90326	0.977	0.994	ns	58	-0.8439037	85.59853	0.401	0.657377	ns
59	0.119692612	107.39146	0.905	0.97263158	ns	59	-0.38574598	82.55592	0.701	0.7866667	ns
60	0.276397931	111.22877	0.783	0.93529412	ns	60	0.05244733	80.01065	0.958	0.9676768	ns
61	0.407362625	116.37947	0.684	0.90864198	ns	61	0.51245023	82.75551	0.61	0.7439024	ns
62	0.386034294	117.57601	0.7	0.90864198	ns	62	0.86124901	86.08225	0.391	0.6516667	ns
63	0.405968844	115.75419	0.686	0.90864198	ns	63	0.94097006	85.16371	0.349	0.6345455	ns
64	0.397078121	113.84037	0.692	0.90864198	ns	64	0.75925689	80.95659	0.45	0.6808824	ns
65	0.52149293	114.99081	0.603	0.90864198	ns	65	0.51623423	77.93408	0.607	0.7439024	ns
66	0.736827194	120.397	0.463	0.84181818	ns	66	0.5129512	80.60518	0.609	0.7439024	ns
67	0.868811969	123.50781	0.387	0.79387755	ns	67	0.77491263	87.71544	0.44	0.68	ns
68	0.94828847	123.0698	0.345	0.75	ns	68	0.92537677	92.71766	0.357	0.6375	ns
69	0.747452686	123.12602	0.456	0.84181818	ns	69	0.74704308	91.22038	0.457	0.6808824	ns
70	0.3690167	121.92933	0.713	0.90864198	ns	70	0.59097902	88.14611	0.556	0.7413333	ns
71	0.134743686	121.3669	0.893	0.97065217	ns	71	0.43295403	85.45391	0.666	0.7655172	ns
72	0.261878551	122.42522	0.794	0.93529412	ns	72	0.18735127	85.9913	0.852	0.8968421	ns
73	0.545774537	124.06956	0.586	0.90864198	ns	73	-0.20251982	85.22981	0.84	0.893617	ns
74	0.738716076	123.18917	0.461	0.84181818	ns	74	-0.39516382	80.65587	0.694	0.7866667	ns
75	0.702835616	117.12423	0.484	0.84912281	ns	75	-0.34110793	74.84027	0.734	0.8054348	ns
76	0.715122551	109.58693	0.476	0.84912281	ns	76	-0.12058567	71.39789	0.904	0.9416667	ns
77	0.797255318	105.93416	0.427	0.82115385	ns	77	0.05308317	70.35093	0.958	0.9676768	ns
78	1.054027102	109.10502	0.294	0.66818182	ns	78	-0.05657604	67.2657	0.955	0.9676768	ns

79	1.143569415	112.2065	0.255	0.65	ns	79	-0.37666167	63.6075	0.708	0.7866667	ns
80	0.864890183	113.50522	0.389	0.79387755	ns	80	-0.49716678	62.86248	0.621	0.7440476	ns
81	0.54742863	115.04047	0.585	0.90864198	ns	81	-0.49054682	65.54053	0.625	0.7440476	ns
82	0.364415499	114.08905	0.716	0.90864198	ns	82	-0.54257408	67.79636	0.589	0.7439024	ns
83	0.201956016	108.76523	0.84	0.95054945	ns	83	-0.44775009	68.14636	0.656	0.7655172	ns
84	0.170070182	104.02961	0.865	0.95054945	ns	84	-0.54969482	69.47608	0.584	0.7439024	ns
85	0.201491009	103.74499	0.841	0.95054945	ns	85	-0.73783545	71.83883	0.463	0.6808824	ns
86	0.095955855	106.00601	0.924	0.97263158	ns	86	-0.65439722	73.03078	0.515	0.7152778	ns
87	-0.078705765	105.12315	0.937	0.97604167	ns	87	-0.96028237	75.76651	0.34	0.6345455	ns
88	-0.178796012	102.26991	0.858	0.95054945	ns	88	-0.97743029	79.1504	0.331	0.6345455	ns
89	-0.44952397	101.42392	0.654	0.90864198	ns	89	-0.86618321	85.39628	0.389	0.6516667	ns
90	-0.448561385	106.6003	0.655	0.90864198	ns	90	-0.77274024	86.86039	0.442	0.68	ns
91	-0.009765931	114.82804	0.992	0.994	ns	91	-0.9661915	88.79066	0.337	0.6345455	ns
92	0.464241104	118.63102	0.643	0.90864198	ns	92	-1.23736289	91.81954	0.219	0.5439024	ns
93	0.567222222	119.47711	0.572	0.90864198	ns	93	-1.56483898	95.75948	0.121	0.526087	ns
94	0.440670323	118.17508	0.66	0.90864198	ns	94	-1.9939043	98.20718	0.0489	0.2573684	ns
95	0.343939269	118.1418	0.732	0.90864198	ns	95	-2.35310968	95.55833	0.0207	0.1573333	ns
96	0.051227694	115.32668	0.959	0.98865979	ns	96	-2.51608937	92.69409	0.0136	0.1573333	ns
97	-0.485552347	107.89134	0.628	0.90864198	ns	97	-2.59982411	92.72064	0.0109	0.1573333	ns
98	-1.132508987	102.71237	0.26	0.65	ns	98	-2.70714346	98.82633	0.008	0.1573333	ns
99	-2.008288322	101.7348	0.0473	0.1892	ns	99	-2.51290712	104.73177	0.0135	0.1573333	ns
100	-2.545215627	101.93097	0.0124	0.06526316	ns	100	-2.08137197	108.03151	0.0398	0.2211111	ns
13	statistic	df	p	p.adj	p.adj.signif	14	statistic	df	p	p.adj	p.adj.signif
1	-2.0703628	87.89678	4.14E-02	5.52E-02	ns	1	-0.69610744	105.63485	0.488	0.6024096	ns
2	-2.1499056	90.04509	3.42E-02	4.89E-02	*	2	-0.75020161	104.99012	0.455	0.58875	ns

3	-2.2554297	92.64852	2.65E-02	4.02E-02	*	3	-0.75837368	105.04458	0.45	0.58875	ns
4	-2.3426611	95.36417	2.12E-02	3.53E-02	*	4	-0.73900924	105.42349	0.462	0.58875	ns
5	-2.3862462	97.04516	1.90E-02	3.46E-02	*	5	-0.72760749	106.11615	0.468	0.58875	ns
6	-2.3890203	97.9054	1.88E-02	3.46E-02	*	6	-0.74919951	107.8535	0.455	0.58875	ns
7	-2.3905103	98.33384	1.87E-02	3.46E-02	*	7	-0.76543414	110.36816	0.446	0.58875	ns
8	-2.3789112	98.42088	1.93E-02	3.46E-02	*	8	-0.7570936	112.8898	0.451	0.58875	ns
9	-2.3646731	98.05391	2.00E-02	3.46E-02	*	9	-0.72872965	114.80503	0.468	0.58875	ns
10	-2.3825532	97.68126	1.91E-02	3.46E-02	*	10	-0.67640463	115.94479	0.5	0.6024096	ns
11	-2.3950021	97.77065	1.85E-02	3.46E-02	*	11	-0.63577032	116.37713	0.526	0.6162791	ns
12	-2.4174104	98.92312	1.75E-02	3.46E-02	*	12	-0.60156691	117.0257	0.549	0.6238636	ns
13	-2.4173023	101.07504	1.74E-02	3.46E-02	*	13	-0.55159015	118.1662	0.582	0.6395604	ns
14	-2.3840394	103.57566	1.89E-02	3.46E-02	*	14	-0.56272496	119.45355	0.575	0.6388889	ns
15	-2.3067263	105.79143	2.30E-02	3.71E-02	*	15	-0.63230436	120.19976	0.528	0.6162791	ns
16	-2.1858879	107.33758	3.10E-02	4.56E-02	*	16	-0.74422861	120.81357	0.458	0.58875	ns
17	-2.0461367	108.53209	4.32E-02	5.61E-02	ns	17	-0.89682643	120.9621	0.372	0.58875	ns
18	-1.9543801	109.77616	5.32E-02	6.65E-02	ns	18	-1.0616865	120.53373	0.291	0.5705882	ns
19	-1.9328631	110.97118	5.58E-02	6.89E-02	ns	19	-1.21872276	119.50024	0.225	0.5217391	ns
20	-1.9612273	112.60534	5.23E-02	6.62E-02	ns	20	-1.35133721	117.9938	0.179	0.5217391	ns
21	-2.0180019	115.35594	4.59E-02	5.88E-02	ns	21	-1.44477579	117.11663	0.151	0.5217391	ns
22	-2.1249851	118.45141	3.57E-02	5.03E-02	ns	22	-1.50988045	116.71713	0.134	0.5217391	ns
23	-2.2731916	121.0377	2.48E-02	3.82E-02	*	23	-1.55209528	116.03084	0.123	0.5217391	ns
24	-2.4323735	122.80437	1.64E-02	3.46E-02	*	24	-1.56022178	115.46969	0.121	0.5217391	ns
25	-2.5541147	123.62976	1.19E-02	3.46E-02	*	25	-1.54727235	115.60363	0.125	0.5217391	ns
26	-2.6802808	124.17151	8.35E-03	3.46E-02	*	26	-1.45537174	115.57443	0.148	0.5217391	ns
27	-2.8500564	124.89331	5.12E-03	2.28E-02	*	27	-1.28770714	114.89144	0.2	0.5217391	ns

28	-3.0245361	125.2475	3.02E-03	1.44E-02	*	28	-1.11141219	113.38429	0.269	0.5604167	ns
29	-3.1904157	125.29727	1.80E-03	9.00E-03	**	29	-0.95756906	111.98551	0.34	0.58875	ns
30	-3.3615839	124.8839	1.03E-03	5.42E-03	**	30	-0.8268239	111.20397	0.41	0.58875	ns
31	-3.5701476	124.07173	5.08E-04	2.99E-03	**	31	-0.75410394	110.16243	0.452	0.58875	ns
32	-3.8209031	123.36086	2.10E-04	1.31E-03	**	32	-0.73399816	108.87051	0.465	0.58875	ns
33	-4.105803	123.10677	7.28E-05	5.20E-04	***	33	-0.72691731	107.66645	0.469	0.58875	ns
34	-4.4021781	123.11276	2.30E-05	1.92E-04	***	34	-0.72341702	106.94145	0.471	0.58875	ns
35	-4.6518088	123.14593	8.35E-06	7.59E-05	****	35	-0.72866252	106.80637	0.468	0.58875	ns
36	-4.7895457	123.0873	4.71E-06	5.23E-05	****	36	-0.73753276	106.3163	0.462	0.58875	ns
37	-4.8437562	122.79982	3.76E-06	4.70E-05	****	37	-0.77567567	105.65433	0.44	0.58875	ns
38	-4.9000188	122.73488	2.96E-06	4.70E-05	****	38	-0.83341579	105.42354	0.406	0.58875	ns
39	-5.0068918	122.83858	1.87E-06	4.58E-05	****	39	-0.88143202	105.65016	0.38	0.58875	ns
40	-5.0900164	122.84951	1.31E-06	4.58E-05	****	40	-0.92263778	105.7459	0.358	0.58875	ns
41	-5.1077606	123.01905	1.21E-06	4.58E-05	****	41	-0.92847497	106.01041	0.355	0.58875	ns
42	-5.0649626	122.62064	1.46E-06	4.58E-05	****	42	-0.96381814	106.87546	0.337	0.58875	ns
43	-4.9631612	121.43816	2.29E-06	4.58E-05	****	43	-1.02368421	108.3454	0.308	0.58875	ns
44	-4.8613825	120.0824	3.57E-06	4.70E-05	****	44	-1.07711225	109.80648	0.284	0.568	ns
45	-4.7138592	118.59033	6.68E-06	6.68E-05	****	45	-1.18058331	110.21946	0.24	0.5217391	ns
46	-4.3873984	115.82218	2.54E-05	1.95E-04	***	46	-1.33749836	109.02069	0.184	0.5217391	ns
47	-3.9396086	110.52292	1.43E-04	9.53E-04	***	47	-1.50982055	107.59084	0.134	0.5217391	ns
48	-3.3981668	102.44209	9.67E-04	5.37E-03	**	48	-1.70503216	105.9226	0.0911	0.4794737	ns
49	-2.8576589	94.46659	5.25E-03	2.28E-02	*	49	-1.88268427	103.20388	0.0626	0.3682353	ns
50	-2.5336252	90.76868	1.30E-02	3.46E-02	*	50	-2.02247775	99.20883	0.0458	0.28625	ns
51	-2.2943606	88.8265	2.41E-02	3.82E-02	*	51	-2.12389415	95.03156	0.0363	0.242	ns
52	-2.1216869	88.24692	3.67E-02	5.08E-02	ns	52	-2.18870251	92.1241	0.0311	0.2392308	ns

53	-2.0725477	89.72373	4.11E-02	5.52E-02	ns	53	-2.23036407	90.87668	0.0282	0.235	ns
54	-2.1152472	92.46782	3.71E-02	5.08E-02	ns	54	-2.26451236	91.25398	0.0259	0.235	ns
55	-2.1702747	95.11754	3.25E-02	4.71E-02	*	55	-2.30093865	92.89328	0.0236	0.235	ns
56	-2.2338602	97.10425	2.78E-02	4.15E-02	*	56	-2.36681514	95.08248	0.02	0.2222222	ns
57	-2.3285687	99.08829	2.19E-02	3.59E-02	*	57	-2.4804768	97.32571	0.0148	0.2114286	ns
58	-2.4256792	101.85357	1.70E-02	3.46E-02	*	58	-2.60577369	98.90811	0.0106	0.2114286	ns
59	-2.4900649	105.04892	1.43E-02	3.46E-02	*	59	-2.70553937	98.8595	0.00803	0.2114286	ns
60	-2.5302386	108.46681	1.28E-02	3.46E-02	*	60	-2.77522096	97.29533	0.00662	0.2114286	ns
61	-2.515993	111.585	1.33E-02	3.46E-02	*	61	-2.76452145	95.886	0.00684	0.2114286	ns
62	-2.4871747	115.01236	1.43E-02	3.46E-02	*	62	-2.68228147	93.87167	0.00864	0.2114286	ns
63	-2.4770027	118.05501	1.47E-02	3.46E-02	*	63	-2.53926759	92.399	0.0128	0.2114286	ns
64	-2.4405695	119.71279	1.61E-02	3.46E-02	*	64	-2.36818618	91.99926	0.02	0.2222222	ns
65	-2.3913765	120.32659	1.83E-02	3.46E-02	*	65	-2.13607931	92.01704	0.0353	0.242	ns
66	-2.3500466	120.37131	2.04E-02	3.46E-02	*	66	-1.85135484	91.34848	0.0673	0.3738889	ns
67	-2.3567744	119.6792	2.01E-02	3.46E-02	*	67	-1.61678234	91.06983	0.109	0.5217391	ns
68	-2.4023579	117.87465	1.79E-02	3.46E-02	*	68	-1.42704748	92.41195	0.157	0.5217391	ns
69	-2.4202221	115.99164	1.71E-02	3.46E-02	*	69	-1.28012013	94.42077	0.204	0.5217391	ns
70	-2.3901989	115.20417	1.85E-02	3.46E-02	*	70	-1.21691261	96.06017	0.227	0.5217391	ns
71	-2.3675822	115.89187	1.96E-02	3.46E-02	*	71	-1.19288276	96.82917	0.236	0.5217391	ns
72	-2.3793598	117.0064	1.90E-02	3.46E-02	*	72	-1.20483488	97.15942	0.231	0.5217391	ns
73	-2.4091471	118.04377	1.75E-02	3.46E-02	*	73	-1.28064135	97.71679	0.203	0.5217391	ns
74	-2.4394928	118.48636	1.62E-02	3.46E-02	*	74	-1.33404399	98.79952	0.185	0.5217391	ns
75	-2.4945318	118.20654	1.40E-02	3.46E-02	*	75	-1.32602434	100.15115	0.188	0.5217391	ns
76	-2.5614523	117.56024	1.17E-02	3.46E-02	*	76	-1.28992536	100.73524	0.2	0.5217391	ns
77	-2.6203524	117.28966	9.95E-03	3.46E-02	*	77	-1.22074484	100.4343	0.225	0.5217391	ns

78	-2.5849201	116.86087	1.10E-02	3.46E-02	*	78	-1.08698859	99.50598	0.28	0.568	ns
79	-2.4483791	116.1309	1.58E-02	3.46E-02	*	79	-0.87794767	98.59092	0.382	0.58875	ns
80	-2.2745552	115.87948	2.48E-02	3.82E-02	*	80	-0.63084189	98.17201	0.53	0.6162791	ns
81	-2.0523078	117.18375	4.24E-02	5.58E-02	ns	81	-0.39446926	98.86328	0.694	0.7229167	ns
82	-1.815129	119.63786	7.20E-02	8.78E-02	ns	82	-0.20716581	100.21392	0.836	0.8540816	ns
83	-1.5808894	121.57026	1.17E-01	1.41E-01	ns	83	-0.05494146	101.99586	0.956	0.956	ns
84	-1.3453824	122.64137	1.81E-01	2.15E-01	ns	84	0.06438785	103.33049	0.949	0.956	ns
85	-1.1341985	122.96758	2.59E-01	3.05E-01	ns	85	0.20684208	102.78787	0.837	0.8540816	ns
86	-0.7783729	121.9344	4.38E-01	4.81E-01	ns	86	0.43507677	99.64452	0.664	0.6989474	ns
87	-0.8602928	121.69388	3.91E-01	4.39E-01	ns	87	0.46413755	97.55275	0.644	0.6893617	ns
88	-0.7417259	120.4082	4.60E-01	5.00E-01	ns	88	0.53187896	95.28576	0.596	0.6478261	ns
89	-0.5945857	119.06769	5.53E-01	5.82E-01	ns	89	0.56671032	94.51406	0.572	0.6388889	ns
90	-0.4940168	117.69517	6.22E-01	6.41E-01	ns	90	0.61651366	94.93133	0.539	0.6195402	ns
91	-0.4113194	116.78559	6.82E-01	6.96E-01	ns	91	0.73946551	96.47386	0.461	0.58875	ns
92	-0.365352	115.694	7.16E-01	7.16E-01	ns	92	0.93948414	98.61172	0.35	0.58875	ns
93	-0.4008491	113.89917	6.89E-01	6.96E-01	ns	93	1.12047121	100.29312	0.265	0.5604167	ns
94	-0.551409	111.82848	5.82E-01	6.06E-01	ns	94	1.23651351	101.49877	0.219	0.5217391	ns
95	-0.6819142	110.50958	4.97E-01	5.29E-01	ns	95	1.31392891	102.2208	0.192	0.5217391	ns
96	-0.7786161	110.22178	4.38E-01	4.81E-01	ns	96	1.32348368	102.20874	0.189	0.5217391	ns
97	-0.9139651	110.40697	3.63E-01	4.13E-01	ns	97	1.20448401	102.31581	0.231	0.5217391	ns
98	-1.0414804	110.7754	3.00E-01	3.49E-01	ns	98	0.9503604	102.41569	0.344	0.58875	ns
99	-1.0059306	108.50335	3.17E-01	3.64E-01	ns	99	0.68145565	102.44459	0.497	0.6024096	ns
100	-0.7342552	100.06185	4.65E-01	5.00E-01	ns	100	0.45828866	102.12792	0.648	0.6893617	ns
15	statistic	df	p	p.adj	p.adj.signif	16	statistic	df	p	p.adj	p.adj.signif
1	-3.1357718	119.6649	0.00216	0.0135	*	1	0.22719125	80.05314	0.821	0.9021978	ns

2	-3.2107418	119.1411	0.0017	0.0135	*	2	0.13357205	80.51155	0.894	0.9463918	ns
3	-3.2459378	118.7397	0.00152	0.0135	*	3	0.01807022	81.02355	0.986	0.986	ns
4	-3.2449442	118.3589	0.00153	0.0135	*	4	-0.11047327	81.55498	0.912	0.9463918	ns
5	-3.2377215	117.9614	0.00156	0.0135	*	5	-0.2489976	82.0359	0.804	0.9021978	ns
6	-3.2386168	117.5807	0.00156	0.0135	*	6	-0.38961548	82.43258	0.698	0.8409639	ns
7	-3.2413302	117.2221	0.00155	0.0135	*	7	-0.52356454	82.73366	0.602	0.7717949	ns
8	-3.2398267	116.9297	0.00156	0.0135	*	8	-0.64625232	82.93737	0.52	0.7123288	ns
9	-3.2331009	116.6881	0.00159	0.0135	*	9	-0.75632715	83.01736	0.452	0.6647059	ns
10	-3.2239447	116.5425	0.00164	0.0135	*	10	-0.8590819	83.00501	0.393	0.633871	ns
11	-3.2190609	116.5917	0.00167	0.0135	*	11	-0.95427874	82.92744	0.343	0.6	ns
12	-3.2149135	116.8968	0.00169	0.0135	*	12	-1.03714577	82.83846	0.303	0.5924528	ns
13	-3.2066801	117.3537	0.00173	0.0135	*	13	-1.11117652	82.74672	0.27	0.5924528	ns
14	-3.1925652	117.8187	0.00181	0.0135	*	14	-1.17843902	82.69411	0.242	0.5860465	ns
15	-3.1718839	118.2009	0.00193	0.0135	*	15	-1.23995587	82.72347	0.218	0.545	ns
16	-3.1452859	118.5115	0.0021	0.0135	*	16	-1.2953172	82.87248	0.199	0.5378378	ns
17	-3.1040136	118.8068	0.00239	0.01405882	*	17	-1.33997965	83.22165	0.184	0.525	ns
18	-3.0547233	119.0868	0.00278	0.01544444	*	18	-1.37791205	83.66899	0.172	0.5212121	ns
19	-3.0006729	119.3596	0.00328	0.01726316	*	19	-1.41298395	84.12988	0.161	0.5212121	ns
20	-2.9390481	119.7092	0.00395	0.01975	*	20	-1.44314863	84.60757	0.153	0.5212121	ns
21	-2.8713861	120.1876	0.00483	0.023	*	21	-1.46936681	85.05817	0.145	0.5212121	ns
22	-2.7965796	120.7267	0.00601	0.02731818	*	22	-1.49296553	85.44077	0.139	0.5212121	ns
23	-2.7106789	121.3379	0.00769	0.03343478	*	23	-1.50866582	85.692	0.135	0.5212121	ns
24	-2.6093437	121.9433	0.0102	0.0425	*	24	-1.51673646	85.82138	0.133	0.5212121	ns
25	-2.4889393	122.5404	0.0142	0.05178571	ns	25	-1.51297203	85.88811	0.134	0.5212121	ns
26	-2.3531391	123.1327	0.0202	0.06121212	ns	26	-1.50155769	85.92764	0.137	0.5212121	ns

27	-2.2173763	123.6843	0.0284	0.07888889	ns	27	-1.48119447	85.90026	0.142	0.5212121	ns
28	-2.08585	124.1376	0.039	0.1	ns	28	-1.44599138	85.81509	0.152	0.5212121	ns
29	-1.9588182	124.4788	0.0524	0.1247619	ns	29	-1.39292187	85.68112	0.167	0.5212121	ns
30	-1.8331018	124.7071	0.0692	0.15377778	ns	30	-1.3252811	85.52742	0.189	0.525	ns
31	-1.7132252	124.8564	0.0892	0.18978723	ns	31	-1.24422036	85.361	0.217	0.545	ns
32	-1.5933103	124.984	0.114	0.228	ns	32	-1.15317985	85.2263	0.252	0.5860465	ns
33	-1.4690977	125.1281	0.144	0.26666667	ns	33	-1.05357001	85.17861	0.295	0.5924528	ns
34	-1.3406819	125.3217	0.182	0.31929825	ns	34	-0.94386264	85.2162	0.348	0.6	ns
35	-1.2118056	125.5579	0.228	0.38	ns	35	-0.82575808	85.35318	0.411	0.6421875	ns
36	-1.0811073	125.8189	0.282	0.44761905	ns	36	-0.70167212	85.52105	0.485	0.6928571	ns
37	-0.9565256	126.0778	0.341	0.51343284	ns	37	-0.56792353	85.75582	0.572	0.7526316	ns
38	-0.8443967	126.3035	0.4	0.57142857	ns	38	-0.42939723	86.0964	0.669	0.8259259	ns
39	-0.7517925	126.4638	0.454	0.62328767	ns	39	-0.29131719	86.56196	0.772	0.8931034	ns
40	-0.6751258	126.6084	0.501	0.668	ns	40	-0.15366287	87.12662	0.878	0.944086	ns
41	-0.6089471	126.7276	0.544	0.6974359	ns	41	-0.01729837	87.79764	0.986	0.986	ns
42	-0.5492406	126.8292	0.584	0.73	ns	42	0.11608963	88.56007	0.908	0.9463918	ns
43	-0.4914074	126.9688	0.624	0.76097561	ns	43	0.24484525	89.39642	0.807	0.9021978	ns
44	-0.4345742	127.1586	0.665	0.79166667	ns	44	0.36760688	90.27917	0.714	0.85	ns
45	-0.3829075	127.3524	0.702	0.80689655	ns	45	0.48017798	91.19838	0.632	0.79375	ns
46	-0.3411184	127.522	0.734	0.80909091	ns	46	0.58184431	92.13548	0.562	0.7493333	ns
47	-0.3137009	127.6731	0.754	0.80909091	ns	47	0.6736602	93.07905	0.502	0.7069444	ns
48	-0.2953431	127.793	0.768	0.80909091	ns	48	0.75582527	94.03226	0.452	0.6647059	ns
49	-0.2807989	127.8859	0.779	0.80909091	ns	49	0.82704246	95.00614	0.41	0.6421875	ns
50	-0.2672216	127.9581	0.79	0.80909091	ns	50	0.89179963	96.0978	0.375	0.6147541	ns
51	-0.2564176	127.9974	0.798	0.80909091	ns	51	0.94556136	97.39558	0.347	0.6	ns

52	-0.2525621	127.984	0.801	0.80909091	ns	52	0.98832551	98.84042	0.325	0.6	ns
53	-0.2596338	127.897	0.796	0.80909091	ns	53	1.01831575	100.34465	0.311	0.5924528	ns
54	-0.2790003	127.735	0.781	0.80909091	ns	54	1.03987999	101.88002	0.301	0.5924528	ns
55	-0.3072913	127.5211	0.759	0.80909091	ns	55	1.05249678	103.42276	0.295	0.5924528	ns
56	-0.3471995	127.3083	0.729	0.80909091	ns	56	1.05222682	104.92983	0.295	0.5924528	ns
57	-0.4004352	127.1475	0.69	0.80232558	ns	57	1.04001643	106.34801	0.301	0.5924528	ns
58	-0.4705796	127.0772	0.639	0.76987952	ns	58	1.01188065	107.62507	0.314	0.5924528	ns
59	-0.5583056	127.1264	0.578	0.73	ns	59	0.96303241	108.71507	0.338	0.6	ns
60	-0.6529239	127.2167	0.515	0.67763158	ns	60	0.89095544	109.5691	0.375	0.6147541	ns
61	-0.7500345	127.3199	0.455	0.62328767	ns	61	0.80104468	110.22769	0.425	0.6469697	ns
62	-0.8454878	127.4285	0.399	0.57142857	ns	62	0.70024283	110.60398	0.485	0.6928571	ns
63	-0.9371219	127.5348	0.35	0.51470588	ns	63	0.59113082	110.61911	0.556	0.7493333	ns
64	-1.0301515	127.6402	0.305	0.46923077	ns	64	0.47544257	110.20557	0.635	0.79375	ns
65	-1.1334598	127.7334	0.259	0.41774194	ns	65	0.35538591	109.40509	0.723	0.8505882	ns
66	-1.2453372	127.8005	0.215	0.36440678	ns	66	0.23180928	108.31658	0.817	0.9021978	ns
67	-1.3645414	127.8577	0.175	0.3125	ns	67	0.10306617	107.03153	0.918	0.9463918	ns
68	-1.4908619	127.9122	0.138	0.26037736	ns	68	-0.02818861	105.6911	0.978	0.986	ns
69	-1.6260114	127.9646	0.106	0.21632653	ns	69	-0.15856948	104.49286	0.874	0.944086	ns
70	-1.7662056	127.9983	0.0797	0.17326087	ns	70	-0.28402084	103.68662	0.777	0.8931034	ns
71	-1.9078966	127.9777	0.0586	0.13318182	ns	71	-0.40715164	103.36052	0.685	0.8353659	ns
72	-2.0486013	127.8498	0.0425	0.10365854	ns	72	-0.53090098	103.42186	0.597	0.7717949	ns
73	-2.173439	127.5641	0.0316	0.08315789	ns	73	-0.66250637	103.64193	0.509	0.7069444	ns
74	-2.278686	127.0798	0.0244	0.06971429	ns	74	-0.79773806	103.95394	0.427	0.6469697	ns
75	-2.3660147	126.4211	0.0195	0.0609375	ns	75	-0.92825326	104.3381	0.355	0.6016949	ns
76	-2.4352431	125.6837	0.0163	0.05433333	ns	76	-1.04764691	104.77282	0.297	0.5924528	ns

77	-2.4785831	124.943	0.0145	0.05178571	ns	77	-1.15479878	105.28008	0.251	0.5860465	ns
78	-2.4935403	124.2456	0.014	0.05178571	ns	78	-1.24638708	105.9088	0.215	0.545	ns
79	-2.4832828	123.5675	0.0144	0.05178571	ns	79	-1.32442595	106.72936	0.188	0.525	ns
80	-2.4487381	122.9349	0.0157	0.05413793	ns	80	-1.39089536	107.74505	0.167	0.5212121	ns
81	-2.3893396	122.3338	0.0184	0.05935484	ns	81	-1.44983726	108.78151	0.15	0.5212121	ns
82	-2.3021165	121.7369	0.023	0.06764706	ns	82	-1.50880203	109.73337	0.134	0.5212121	ns
83	-2.1932478	121.1325	0.0302	0.08162162	ns	83	-1.56426865	110.54944	0.121	0.5212121	ns
84	-2.0646561	120.4769	0.0411	0.10275	ns	84	-1.61105353	111.20031	0.11	0.5212121	ns
85	-1.9308171	119.7857	0.0559	0.13	ns	85	-1.64445085	111.78705	0.103	0.5212121	ns
86	-1.4999084	119.2901	0.136	0.26037736	ns	86	-1.52060342	110.46929	0.131	0.5212121	ns
87	-1.6671828	118.5321	0.0981	0.204375	ns	87	-1.674439	113.11173	0.0968	0.5212121	ns
88	-1.5358574	118.1268	0.127	0.24901961	ns	88	-1.67200273	113.86165	0.0973	0.5212121	ns
89	-1.403966	118.0133	0.163	0.29636364	ns	89	-1.66751439	114.60369	0.0981	0.5212121	ns
90	-1.280013	118.2072	0.203	0.35	ns	90	-1.66228622	115.29585	0.0992	0.5212121	ns
91	-1.166706	118.5609	0.246	0.40327869	ns	91	-1.66035911	115.90217	0.0995	0.5212121	ns
92	-1.059973	118.9833	0.291	0.4546875	ns	92	-1.66732111	116.45767	0.0981	0.5212121	ns
93	-0.9501131	119.4296	0.344	0.51343284	ns	93	-1.68513522	117.03278	0.0946	0.5212121	ns
94	-0.8341832	119.822	0.406	0.57183099	ns	94	-1.71307794	117.57755	0.0893	0.5212121	ns
95	-0.7283627	120.037	0.468	0.63243243	ns	95	-1.7502836	117.93501	0.0827	0.5212121	ns
96	-0.6298173	119.9574	0.53	0.68831169	ns	96	-1.79202183	117.98747	0.0757	0.5212121	ns
97	-0.5301246	119.5761	0.597	0.73703704	ns	97	-1.81618393	117.74924	0.0719	0.5212121	ns
98	-0.4205702	118.9802	0.675	0.79411765	ns	98	-1.80558281	117.26081	0.0735	0.5212121	ns
99	-0.3082691	118.2051	0.758	0.80909091	ns	99	-1.7525787	116.57117	0.0823	0.5212121	ns
100	-0.194798	117.3419	0.846	0.846	ns	100	-1.66819639	115.78626	0.098	0.5212121	ns
17	statistic	df	p	p.adj	p.adj.signif	18	statistic	df	p	p.adj	p.adj.signif

1	1.17752754	82.0265	0.242	0.50408163	ns	1	-0.019991537	106.00509	0.984	0.9979798	ns
2	1.04529323	85.65016	0.299	0.56415094	ns	2	0.019115238	105.67646	0.985	0.9979798	ns
3	0.89906747	91.15363	0.371	0.5875	ns	3	0.06698552	105.28899	0.947	0.9979798	ns
4	0.7320426	98.42351	0.466	0.64722222	ns	4	0.12581203	104.86513	0.9	0.9979798	ns
5	0.54217939	106.02818	0.589	0.74556962	ns	5	0.184068071	104.38304	0.854	0.9979798	ns
6	0.34244108	111.08776	0.733	0.80879121	ns	6	0.23798798	103.92419	0.812	0.9979798	ns
7	0.1357041	113.07789	0.892	0.91958763	ns	7	0.285510588	103.49081	0.776	0.9979798	ns
8	-0.0345237	112.78211	0.973	0.98282828	ns	8	0.326777119	103.00134	0.744	0.9979798	ns
9	-0.17130337	112.18392	0.864	0.90947368	ns	9	0.353889819	102.35404	0.724	0.9979798	ns
10	-0.30351103	112.14477	0.762	0.81808511	ns	10	0.360019241	101.50683	0.72	0.9979798	ns
11	-0.45803559	112.26967	0.648	0.77738095	ns	11	0.349706475	100.51371	0.727	0.9979798	ns
12	-0.65278194	112.39904	0.515	0.70547945	ns	12	0.317881372	99.42354	0.751	0.9979798	ns
13	-0.89202983	112.06248	0.374	0.5875	ns	13	0.265919747	98.23297	0.791	0.9979798	ns
14	-1.14859565	111.51307	0.253	0.506	ns	14	0.204951159	96.98088	0.838	0.9979798	ns
15	-1.3918397	110.91848	0.167	0.37111111	ns	15	0.138123007	95.70899	0.89	0.9979798	ns
16	-1.58737187	110.42537	0.115	0.2804878	ns	16	0.068643886	94.60387	0.945	0.9979798	ns
17	-1.73649976	109.96984	0.0853	0.23432432	ns	17	0.001118459	93.8183	0.999	0.999	ns
18	-1.845437	109.88604	0.0677	0.22090909	ns	18	-0.061740796	93.39932	0.951	0.9979798	ns
19	-1.93477081	109.93864	0.0556	0.19172414	ns	19	-0.113232289	93.36269	0.91	0.9979798	ns
20	-2.02724914	109.65675	0.0451	0.16703704	ns	20	-0.158683856	93.60139	0.874	0.9979798	ns
21	-2.13037161	108.95468	0.0354	0.14153846	ns	21	-0.201755823	94.00335	0.841	0.9979798	ns
22	-2.24849324	107.99209	0.0266	0.11083333	ns	22	-0.240247915	94.45867	0.811	0.9979798	ns
23	-2.3814206	106.81919	0.019	0.08636364	ns	23	-0.273756763	94.84063	0.785	0.9979798	ns
24	-2.52366102	105.53102	0.0131	0.0655	ns	24	-0.30511751	95.04428	0.761	0.9979798	ns
25	-2.67766019	104.03372	0.00862	0.04788889	*	25	-0.333624068	95.11111	0.739	0.9979798	ns

26	-2.84209422	102.55709	0.00541	0.0338125	*	26	-0.358787358	95.03193	0.721	0.9979798	ns
27	-3.01337181	101.02785	0.00327	0.02335714	*	27	-0.381796887	94.83985	0.703	0.9979798	ns
28	-3.17734091	99.24079	0.00198	0.0165	*	28	-0.401620227	94.57273	0.689	0.9979798	ns
29	-3.32219603	97.65838	0.00126	0.0126	*	29	-0.418297248	94.25048	0.677	0.9979798	ns
30	-3.44256517	96.54162	0.000853	0.01095	*	30	-0.430379655	93.76623	0.668	0.9979798	ns
31	-3.5262005	95.79397	0.000649	0.01083333	*	31	-0.432102161	93.08898	0.667	0.9979798	ns
32	-3.57609625	95.27366	0.00055	0.01083333	*	32	-0.419439238	92.25819	0.676	0.9979798	ns
33	-3.60320409	95.47815	0.000501	0.01083333	*	33	-0.387504997	91.30555	0.699	0.9979798	ns
34	-3.60293627	96.1583	0.0005	0.01083333	*	34	-0.338211765	90.3227	0.736	0.9979798	ns
35	-3.57929772	97.16256	0.00054	0.01083333	*	35	-0.280790172	89.30549	0.78	0.9979798	ns
36	-3.52262043	98.13416	0.00065	0.01083333	*	36	-0.225121978	88.27849	0.822	0.9979798	ns
37	-3.43211091	98.89204	0.000876	0.01095	*	37	-0.174836192	87.33012	0.862	0.9979798	ns
38	-3.32464685	99.13324	0.00124	0.0126	*	38	-0.131744105	86.56877	0.895	0.9979798	ns
39	-3.20966133	98.819	0.00179	0.01627273	*	39	-0.096080146	86.07911	0.924	0.9979798	ns
40	-3.08463166	98.13846	0.00265	0.02038462	*	40	-0.059876942	85.91626	0.952	0.9979798	ns
41	-2.94585972	97.26422	0.00403	0.02686667	*	41	-0.014571982	86.01064	0.988	0.9979798	ns
42	-2.79338523	96.51292	0.00629	0.037	*	42	0.045509252	86.2287	0.964	0.9979798	ns
43	-2.63341743	95.99151	0.00985	0.05184211	ns	43	0.124651328	86.50628	0.901	0.9979798	ns
44	-2.46735031	95.72056	0.0154	0.07333333	ns	44	0.224619026	86.78276	0.823	0.9979798	ns
45	-2.29657882	95.29932	0.0238	0.10347826	ns	45	0.334619519	86.93102	0.739	0.9979798	ns
46	-2.11797231	94.68888	0.0368	0.14153846	ns	46	0.450341356	86.98021	0.654	0.9979798	ns
47	-1.94213006	93.88614	0.0551	0.19172414	ns	47	0.570325771	86.94221	0.57	0.9979798	ns
48	-1.77654825	93.03793	0.0789	0.22542857	ns	48	0.695906599	86.89778	0.488	0.9207547	ns
49	-1.60736562	92.24285	0.111	0.2804878	ns	49	0.832294917	86.91463	0.408	0.7846154	ns
50	-1.40531191	91.81206	0.163	0.37045455	ns	50	0.984182627	86.93917	0.328	0.7288889	ns

51	-1.16580651	91.77255	0.247	0.50408163	ns	51	1.165060343	86.94557	0.247	0.625	ns
52	-0.89602479	92.27967	0.373	0.5875	ns	52	1.370503654	87.01645	0.174	0.54375	ns
53	-0.60747769	93.43602	0.545	0.72435897	ns	53	1.582951766	87.38807	0.117	0.5086957	ns
54	-0.30761464	95.00387	0.759	0.81808511	ns	54	1.795812462	88.07433	0.076	0.3619048	ns
55	-0.00456983	96.73925	0.996	0.996	ns	55	1.99776776	88.9406	0.0488	0.2568421	ns
56	0.29441573	98.3692	0.769	0.81808511	ns	56	2.168604503	89.83917	0.0328	0.1929412	ns
57	0.58120903	99.93306	0.562	0.72435897	ns	57	2.304777347	90.64112	0.0235	0.1566667	ns
58	0.85775644	101.97308	0.393	0.5969697	ns	58	2.423317975	91.31547	0.0174	0.1338462	ns
59	1.11466623	104.29627	0.268	0.5254902	ns	59	2.538809153	91.84891	0.0128	0.1125	ns
60	1.34103657	106.98104	0.183	0.39782609	ns	60	2.637129622	92.24248	0.00981	0.0981	ns
61	1.54299956	110.21705	0.126	0.3	ns	61	2.700599895	92.57452	0.00823	0.0981	ns
62	1.69251699	113.67365	0.0933	0.24552632	ns	62	2.734075438	93.00102	0.00749	0.0981	ns
63	1.77453012	116.08557	0.0786	0.22542857	ns	63	2.749691425	93.81422	0.00716	0.0981	ns
64	1.81878649	117.4564	0.0715	0.22090909	ns	64	2.746990595	94.91137	0.0072	0.0981	ns
65	1.83624217	118.70148	0.0688	0.22090909	ns	65	2.734397523	96.09877	0.00744	0.0981	ns
66	1.80911104	120.03182	0.0729	0.22090909	ns	66	2.731495236	97.77536	0.00748	0.0981	ns
67	1.72681696	121.26922	0.0867	0.23432432	ns	67	2.745364694	99.9851	0.00717	0.0981	ns
68	1.59225919	122.32387	0.114	0.2804878	ns	68	2.727256572	102.2933	0.00752	0.0981	ns
69	1.41876593	123.15431	0.158	0.36744186	ns	69	2.645509807	104.49255	0.00942	0.0981	ns
70	1.23385389	123.47758	0.22	0.46808511	ns	70	2.510857873	106.5247	0.0135	0.1125	ns
71	1.04321866	123.34852	0.299	0.56415094	ns	71	2.352634735	108.70443	0.0204	0.1457143	ns
72	0.83171338	123.14532	0.407	0.59852941	ns	72	2.184991568	110.86966	0.031	0.1929412	ns
73	0.59562777	123.26851	0.553	0.72435897	ns	73	2.008449828	112.83535	0.047	0.2568421	ns
74	0.33824354	123.88443	0.736	0.80879121	ns	74	1.817308533	114.54118	0.0718	0.359	ns
75	0.08681853	124.72424	0.931	0.95	ns	75	1.603050576	115.86917	0.112	0.5086957	ns

76	-0.15583285	125.37899	0.876	0.9125	ns	76	1.384537409	117.08102	0.169	0.54375	ns
77	-0.37449611	125.78042	0.709	0.79662921	ns	77	1.174853134	118.42055	0.242	0.625	ns
78	-0.57631783	125.74701	0.565	0.72435897	ns	78	1.007800366	120.22332	0.316	0.7181818	ns
79	-0.73508262	125.5337	0.464	0.64722222	ns	79	0.897453956	122.15547	0.371	0.7729167	ns
80	-0.83520473	125.25543	0.405	0.59852941	ns	80	0.858160283	123.9292	0.392	0.7823529	ns
81	-0.88902938	124.89007	0.376	0.5875	ns	81	0.880442863	125.26143	0.38	0.7755102	ns
82	-0.92133854	124.42093	0.359	0.5875	ns	82	0.948110173	126.10036	0.345	0.75	ns
83	-0.95057468	123.8876	0.344	0.58305085	ns	83	1.034194362	126.32739	0.303	0.7093023	ns
84	-0.97761691	123.32666	0.33	0.57894737	ns	84	1.112742668	125.92427	0.268	0.6536585	ns
85	-1.00059016	122.43879	0.319	0.56964286	ns	85	1.171345333	124.77426	0.244	0.625	ns
86	-0.78458962	117.3672	0.434	0.62898551	ns	86	1.327244775	120.73953	0.187	0.55	ns
87	-1.02279241	119.86479	0.308	0.56964286	ns	87	1.279771839	119.9479	0.203	0.5638889	ns
88	-1.00270626	118.92253	0.318	0.56964286	ns	88	1.343998304	116.52532	0.182	0.55	ns
89	-0.94951272	118.48217	0.344	0.58305085	ns	89	1.407608316	112.83684	0.162	0.54375	ns
90	-0.85459507	118.50153	0.394	0.5969697	ns	90	1.456804939	109.13407	0.148	0.5285714	ns
91	-0.7406091	118.87678	0.46	0.64722222	ns	91	1.489836301	105.65817	0.139	0.5285714	ns
92	-0.62529221	119.3409	0.533	0.72027027	ns	92	1.509585865	102.70041	0.134	0.5285714	ns
93	-0.52001278	119.51422	0.604	0.755	ns	93	1.506923223	100.91493	0.135	0.5285714	ns
94	-0.43420548	119.31387	0.665	0.78235294	ns	94	1.47184484	100.55465	0.144	0.5285714	ns
95	-0.3976529	118.03483	0.692	0.78977273	ns	95	1.396733948	101.69917	0.166	0.54375	ns
96	-0.39373427	116.00294	0.695	0.78977273	ns	96	1.286297162	103.74573	0.201	0.5638889	ns
97	-0.41415726	113.63668	0.68	0.78977273	ns	97	1.15753313	106.42268	0.25	0.625	ns
98	-0.45015651	111.69315	0.653	0.77738095	ns	98	1.031499611	109.59681	0.305	0.7093023	ns
99	-0.47815214	110.38672	0.633	0.77195122	ns	99	0.927673341	112.84384	0.356	0.7574468	ns
100	-0.48495551	109.70114	0.629	0.77195122	ns	100	0.845974315	115.80745	0.399	0.7823529	ns

19	statistic	df	p	p.adj	p.adj.signif	20	statistic	df	p	p.adj	p.adj.signif
1	-1.71825347	127.9994	0.0882	0.3528	ns	1	-0.5443753	108.66029	0.587	0.7623377	ns
2	-1.88047401	127.9716	0.0623	0.2743478	ns	2	-0.6933046	108.34062	0.49	0.7085714	ns
3	-1.96415399	127.8456	0.0517	0.2461905	ns	3	-0.8312871	109.16605	0.408	0.6688525	ns
4	-2.05235262	127.5872	0.0422	0.2357895	ns	4	-0.9700089	109.72611	0.334	0.6320755	ns
5	-2.13655817	127.1412	0.0345	0.2357895	ns	5	-1.1415691	109.39137	0.256	0.5333333	ns
6	-2.18988624	126.469	0.0304	0.2357895	ns	6	-1.3140705	108.91829	0.192	0.4682927	ns
7	-2.19777608	125.8902	0.0298	0.2357895	ns	7	-1.4636178	108.32793	0.146	0.3945946	ns
8	-2.18232387	125.6096	0.0309	0.2357895	ns	8	-1.5993989	107.44883	0.113	0.3424242	ns
9	-2.1608479	125.5488	0.0326	0.2357895	ns	9	-1.7020016	106.16675	0.0917	0.2865625	ns
10	-2.12409011	125.7876	0.0356	0.2357895	ns	10	-1.7671787	104.99876	0.0801	0.2803448	ns
11	-2.07343232	126.2964	0.0402	0.2357895	ns	11	-1.7973715	103.42641	0.0752	0.2785185	ns
12	-2.04850143	126.8158	0.0426	0.2357895	ns	12	-1.8006582	101.87831	0.0747	0.2785185	ns
13	-2.04868695	127.1079	0.0426	0.2357895	ns	13	-1.822171	100.32807	0.0714	0.2785185	ns
14	-2.02620396	127.1849	0.0448	0.2357895	ns	14	-1.8938379	98.1386	0.0612	0.2570833	ns
15	-1.96421151	127.1642	0.0517	0.2461905	ns	15	-2.0005455	95.94925	0.0483	0.2319048	ns
16	-1.8748489	127.1855	0.0631	0.2743478	ns	16	-2.1153997	94.1512	0.037	0.2319048	ns
17	-1.76188675	127.1168	0.0805	0.3354167	ns	17	-2.1969306	92.81158	0.0305	0.2319048	ns
18	-1.62738396	126.843	0.106	0.4076923	ns	18	-2.2049201	92.4445	0.0299	0.2319048	ns
19	-1.45953005	126.4184	0.147	0.525	ns	19	-2.1459378	92.83498	0.0345	0.2319048	ns
20	-1.23907907	125.9454	0.218	0.7283582	ns	20	-2.0334464	93.55275	0.0448	0.2319048	ns
21	-0.99120966	125.6591	0.323	0.7283582	ns	21	-1.8907875	94.44279	0.0617	0.2570833	ns
22	-0.75810999	125.6148	0.45	0.7283582	ns	22	-1.720924	95.77377	0.0885	0.2854839	ns
23	-0.59646002	125.9955	0.552	0.7594595	ns	23	-1.5413464	97.18499	0.126	0.3685714	ns
24	-0.51583149	126.3154	0.607	0.78625	ns	24	-1.3712598	98.41854	0.173	0.4435897	ns

25	-0.52160436	126.4061	0.603	0.78625	ns	25	-1.1931183	99.19149	0.236	0.5021277	ns
26	-0.58128139	126.5227	0.562	0.7594595	ns	26	-1.0138926	99.7713	0.313	0.6137255	ns
27	-0.64059077	126.7617	0.523	0.757971	ns	27	-0.875198	100.68216	0.384	0.662069	ns
28	-0.6801382	127.0896	0.498	0.7323529	ns	28	-0.7712812	101.92002	0.442	0.7015873	ns
29	-0.70190898	127.4051	0.484	0.7283582	ns	29	-0.6911398	103.01712	0.491	0.7085714	ns
30	-0.73303799	127.6721	0.465	0.7283582	ns	30	-0.6305619	103.82446	0.53	0.730137	ns
31	-0.76903358	127.858	0.443	0.7283582	ns	31	-0.5662377	104.2788	0.572	0.7618421	ns
32	-0.81194175	127.9491	0.418	0.7283582	ns	32	-0.5119191	103.97363	0.61	0.7721519	ns
33	-0.88005322	127.9522	0.38	0.7283582	ns	33	-0.4461637	103.34781	0.656	0.7809524	ns
34	-0.95166063	127.8724	0.343	0.7283582	ns	34	-0.3694608	102.76437	0.713	0.810989	ns
35	-1.02934525	127.5916	0.305	0.7283582	ns	35	-0.3119454	102.09032	0.756	0.8129032	ns
36	-1.10235147	127.1014	0.272	0.7283582	ns	36	-0.2785149	101.6187	0.781	0.815625	ns
37	-1.13980242	126.703	0.257	0.7283582	ns	37	-0.2400168	101.12863	0.811	0.8285714	ns
38	-1.1348265	126.4418	0.259	0.7283582	ns	38	-0.1965849	100.79063	0.845	0.845	ns
39	-1.11473502	126.1759	0.267	0.7283582	ns	39	-0.1957279	102.56655	0.845	0.845	ns
40	-1.0802444	125.8731	0.282	0.7283582	ns	40	-0.2382342	106.9478	0.812	0.8285714	ns
41	-1.04286211	125.5651	0.299	0.7283582	ns	41	-0.338261	112.61377	0.736	0.810989	ns
42	-1.0064454	125.3093	0.316	0.7283582	ns	42	-0.4977248	117.64962	0.62	0.7722892	ns
43	-0.94826837	125.3722	0.345	0.7283582	ns	43	-0.6984054	121.35656	0.486	0.7085714	ns
44	-0.86482299	125.628	0.389	0.7283582	ns	44	-0.9544542	124.25014	0.342	0.6333333	ns
45	-0.77317281	125.7327	0.441	0.7283582	ns	45	-1.2499582	126.16473	0.214	0.4755556	ns
46	-0.6975678	125.5712	0.487	0.7283582	ns	46	-1.5267964	127.16939	0.129	0.3685714	ns
47	-0.58448191	125.2297	0.56	0.7594595	ns	47	-1.7568666	127.51806	0.0813	0.2803448	ns
48	-0.42385008	124.3957	0.672	0.8096386	ns	48	-1.9620013	127.38762	0.0519	0.2359091	ns
49	-0.27089904	122.6341	0.787	0.8865169	ns	49	-2.1203136	126.77398	0.0359	0.2319048	ns

50	-0.14364361	120.4537	0.886	0.928125	ns	50	-2.1892981	126.04813	0.0304	0.2319048	ns
51	-0.03794598	118.3174	0.97	0.97	ns	51	-2.1997738	125.17144	0.0297	0.2319048	ns
52	0.04378836	115.8473	0.965	0.97	ns	52	-2.1592762	123.50939	0.0328	0.2319048	ns
53	0.10116983	112.9268	0.92	0.9484536	ns	53	-2.078077	121.00656	0.0398	0.2319048	ns
54	0.13672621	110.1284	0.891	0.928125	ns	54	-2.0143082	117.34215	0.0463	0.2319048	ns
55	0.18356256	107.5546	0.855	0.928125	ns	55	-2.0237987	113.48109	0.0453	0.2319048	ns
56	0.2533956	105.5011	0.8	0.8888889	ns	56	-2.0534826	110.90171	0.0424	0.2319048	ns
57	0.36387714	104.1647	0.717	0.8337209	ns	57	-2.0854187	111.14535	0.0393	0.2319048	ns
58	0.49020585	103.4625	0.625	0.78625	ns	58	-2.1607727	112.9018	0.0328	0.2319048	ns
59	0.62287284	103.4229	0.535	0.7594595	ns	59	-2.2713729	114.03958	0.025	0.2319048	ns
60	0.74085984	103.8183	0.46	0.7283582	ns	60	-2.3098707	114.76851	0.0227	0.2319048	ns
61	0.8284948	104.291	0.409	0.7283582	ns	61	-2.2963962	115.31317	0.0235	0.2319048	ns
62	0.87623873	104.6056	0.383	0.7283582	ns	62	-2.1754681	114.04998	0.0317	0.2319048	ns
63	0.83604461	105.0025	0.405	0.7283582	ns	63	-1.9932938	110.47119	0.0487	0.2319048	ns
64	0.6967071	104.589	0.488	0.7283582	ns	64	-1.7307101	106.56228	0.0864	0.2854839	ns
65	0.51938071	102.9155	0.605	0.78625	ns	65	-1.3929528	104.29756	0.167	0.4394737	ns
66	0.40382335	101.3826	0.687	0.8178571	ns	66	-1.1157742	104.21545	0.267	0.534	ns
67	0.38213241	101.9676	0.703	0.8270588	ns	67	-0.9677779	105.16439	0.335	0.6320755	ns
68	0.42486632	104.1763	0.672	0.8096386	ns	68	-0.8397089	106.07713	0.403	0.6688525	ns
69	0.49213015	106.8552	0.624	0.78625	ns	69	-0.73947	107.7057	0.461	0.7085714	ns
70	0.61006516	108.7097	0.543	0.7594595	ns	70	-0.6829901	110.27828	0.496	0.7085714	ns
71	0.77334488	109.9539	0.441	0.7283582	ns	71	-0.6009903	113.61789	0.549	0.7418919	ns
72	0.88382496	111.2206	0.379	0.7283582	ns	72	-0.4678991	116.47962	0.641	0.7722892	ns
73	0.91987758	111.872	0.36	0.7283582	ns	73	-0.3353844	117.42627	0.738	0.810989	ns
74	0.92793017	111.806	0.355	0.7283582	ns	74	-0.2765858	115.73192	0.783	0.815625	ns

75	0.93759622	112.1088	0.35	0.7283582	ns	75	-0.2883173	114.00039	0.774	0.815625	ns
76	0.93420355	113.4573	0.352	0.7283582	ns	76	-0.3791054	115.16408	0.705	0.810989	ns
77	0.93515573	115.8457	0.352	0.7283582	ns	77	-0.4841689	116.95548	0.629	0.7722892	ns
78	0.94149197	118.2841	0.348	0.7283582	ns	78	-0.5561378	118.48998	0.579	0.7618421	ns
79	0.94311789	119.7582	0.348	0.7283582	ns	79	-0.624696	119.62378	0.533	0.730137	ns
80	0.88351615	119.7474	0.379	0.7283582	ns	80	-0.693883	121.16567	0.489	0.7085714	ns
81	0.71469096	118.9051	0.476	0.7283582	ns	81	-0.78862	121.83177	0.432	0.6967742	ns
82	0.48403851	117.8138	0.629	0.78625	ns	82	-0.8797949	121.91985	0.381	0.662069	ns
83	0.28386243	117.2217	0.777	0.8865169	ns	83	-0.8846809	123.01997	0.378	0.662069	ns
84	0.1435007	117.4056	0.886	0.928125	ns	84	-0.8333676	124.05903	0.406	0.6688525	ns
85	0.04738326	118.1708	0.962	0.97	ns	85	-0.7132041	125.02379	0.477	0.7085714	ns
86	0.18450508	116.8878	0.854	0.928125	ns	86	-0.5242567	125.74161	0.601	0.7705128	ns
87	-0.1475262	119.6135	0.883	0.928125	ns	87	-0.4284221	126.91071	0.669	0.7870588	ns
88	-0.26786686	120.8331	0.789	0.8865169	ns	88	-0.3601376	127.61782	0.719	0.810989	ns
89	-0.46935482	122.8226	0.64	0.7901235	ns	89	-0.3231475	127.98606	0.747	0.8119565	ns
90	-0.75474476	125.1587	0.452	0.7283582	ns	90	-0.3446834	127.97145	0.731	0.810989	ns
91	-1.13393645	127.1167	0.259	0.7283582	ns	91	-0.4673114	127.9565	0.641	0.7722892	ns
92	-1.59389527	127.8775	0.113	0.4185185	ns	92	-0.6598865	127.99999	0.511	0.7197183	ns
93	-2.0301671	127.993	0.0444	0.2357895	ns	93	-0.9219964	127.87536	0.358	0.6509091	ns
94	-2.36543147	128	0.0195	0.2357895	ns	94	-1.1187739	127.32098	0.265	0.534	ns
95	-2.58758688	127.9984	0.0108	0.18	ns	95	-1.2116449	126.51328	0.228	0.4956522	ns
96	-2.70800564	127.9858	0.00769	0.1786	ns	96	-1.2572567	125.6831	0.211	0.4755556	ns
97	-2.75914513	127.9202	0.00665	0.1786	ns	97	-1.2824886	124.97789	0.202	0.4697674	ns
98	-2.73674383	127.7088	0.00709	0.1786	ns	98	-1.2879275	124.76899	0.2	0.4697674	ns
99	-2.68862466	127.1147	0.00814	0.1786	ns	99	-1.3451424	124.48439	0.181	0.4525	ns

100	-2.65585055	126.2144	0.00893	0.1786	ns	100	-1.4988517	123.46893	0.136	0.3777778	ns

Table S6： Comparison of Mean RD Between prodromal PD carriers of dual LRRK2 and GBA variants and Healthy Controls Across 20 Fiber Tracts. Statistical significance was set at $p < 0.05$, corrected by FDR.

1	statistic	df	p	p.adj	p.adj.signif	2	statistic	df	p	p.adj	p.adj.signif
1	2.14795721	126.8527	0.0336	0.0675	ns	1	0.460716972	126.2777	0.646	0.997	ns
2	2.16847246	126.47223	0.032	0.066666667	ns	2	0.470413428	126.79047	0.639	0.997	ns
3	2.18350829	126.07137	0.0308	0.065531915	ns	3	0.482118195	127.28155	0.631	0.997	ns
4	2.19220149	125.59896	0.0302	0.065531915	ns	4	0.476505265	127.58029	0.635	0.997	ns
5	2.20843231	125.12063	0.029	0.065531915	ns	5	0.451490915	127.71897	0.652	0.997	ns
6	2.22444206	124.71344	0.0279	0.065531915	ns	6	0.400495313	127.77872	0.689	0.997	ns
7	2.24334031	124.50921	0.0266	0.065531915	ns	7	0.334528667	127.82993	0.739	0.997	ns
8	2.2571458	124.43372	0.0257	0.065531915	ns	8	0.27368783	127.88221	0.785	0.997	ns
9	2.25737485	124.41162	0.0257	0.065531915	ns	9	0.215342999	127.93961	0.83	0.997	ns
10	2.24163131	124.43036	0.0268	0.065531915	ns	10	0.160252099	127.96855	0.873	0.997	ns
11	2.20734483	124.49551	0.0291	0.065531915	ns	11	0.1028016	127.9753	0.918	0.997	ns
12	2.14390015	124.70007	0.034	0.0675	ns	12	0.054212248	127.97695	0.957	0.997	ns
13	2.02844717	124.94179	0.0446	0.078245614	ns	13	0.027397887	127.97379	0.978	0.997	ns
14	1.84239423	125.0325	0.0678	0.111147541	ns	14	0.014434016	127.95855	0.989	0.997	ns
15	1.59551965	124.68695	0.113	0.166176471	ns	15	-0.004150664	127.88198	0.997	0.997	ns
16	1.31413248	123.63018	0.191	0.261643836	ns	16	-0.043864011	127.58586	0.965	0.997	ns
17	1.0199619	121.82808	0.31	0.407894737	ns	17	-0.098869815	126.82469	0.921	0.997	ns
18	0.7450871	119.34064	0.458	0.547619048	ns	18	-0.177510115	125.31165	0.859	0.997	ns

19	0.51616606	116.42418	0.607	0.652688172	ns	19	-0.264126216	122.89908	0.792	0.997	ns
20	0.33334413	113.25602	0.739	0.769791667	ns	20	-0.318979537	119.80841	0.75	0.997	ns
21	0.17343686	109.90974	0.863	0.880612245	ns	21	-0.343184264	116.34891	0.732	0.997	ns
22	0.03146429	106.89277	0.975	0.975	ns	22	-0.328159364	113.06353	0.743	0.997	ns
23	-0.09470084	104.42799	0.925	0.934343434	ns	23	-0.261858192	110.19376	0.794	0.997	ns
24	-0.22304842	102.96471	0.824	0.849484536	ns	24	-0.144053296	107.87211	0.886	0.997	ns
25	-0.35999539	102.68267	0.72	0.757894737	ns	25	-0.007264003	105.95568	0.994	0.997	ns
26	-0.48274867	103.2999	0.63	0.670212766	ns	26	0.095773342	104.66424	0.924	0.997	ns
27	-0.58051388	104.15034	0.563	0.611956522	ns	27	0.127896762	104.25228	0.898	0.997	ns
28	-0.67357504	104.81588	0.502	0.577011494	ns	28	0.101655644	104.30463	0.919	0.997	ns
29	-0.7416904	104.94573	0.46	0.547619048	ns	29	0.041571235	104.11596	0.967	0.997	ns
30	-0.76898475	104.63616	0.444	0.547619048	ns	30	-0.0327452	103.48127	0.974	0.997	ns
31	-0.74331594	104.33147	0.459	0.547619048	ns	31	-0.113883676	102.33551	0.91	0.997	ns
32	-0.69349356	103.77103	0.49	0.573255814	ns	32	-0.192268331	100.81048	0.848	0.997	ns
33	-0.63813193	102.82911	0.525	0.58988764	ns	33	-0.243124008	99.18005	0.808	0.997	ns
34	-0.61199479	101.76247	0.542	0.595604396	ns	34	-0.243371607	97.75814	0.808	0.997	ns
35	-0.61951993	101.21857	0.537	0.595604396	ns	35	-0.188809399	96.78062	0.851	0.997	ns
36	-0.64249642	101.33355	0.522	0.58988764	ns	36	-0.115761988	96.17227	0.908	0.997	ns
37	-0.6885999	101.575	0.493	0.573255814	ns	37	-0.057989292	96.23619	0.954	0.997	ns
38	-0.76466309	101.85687	0.446	0.547619048	ns	38	-0.06325882	97.11901	0.95	0.997	ns
39	-0.84085065	102.46024	0.402	0.508860759	ns	39	-0.120020937	98.38642	0.905	0.997	ns
40	-0.91169412	103.54042	0.364	0.466666667	ns	40	-0.179148904	99.30971	0.858	0.997	ns
41	-0.98740841	105.72334	0.326	0.423376623	ns	41	-0.197536504	99.46767	0.844	0.997	ns
42	-1.09930863	109.04386	0.274	0.365333333	ns	42	-0.13926993	98.95475	0.89	0.997	ns
43	-1.25144856	112.17851	0.213	0.287837838	ns	43	-0.035866598	98.49509	0.971	0.997	ns

44	-1.43855792	113.63677	0.153	0.2125	ns	44	0.077062469	98.304	0.939	0.997	ns
45	-1.64099415	113.46252	0.104	0.156716418	ns	45	0.184856847	97.832	0.854	0.997	ns
46	-1.83521926	112.57013	0.0691	0.111451613	ns	46	0.271805445	96.6033	0.786	0.997	ns
47	-1.99347495	111.85068	0.0486	0.083793103	ns	47	0.316081261	94.54786	0.753	0.997	ns
48	-2.1143533	110.81689	0.0367	0.069245283	ns	48	0.305577769	92.32479	0.761	0.997	ns
49	-2.21404558	108.66002	0.0289	0.065531915	ns	49	0.259779582	90.56462	0.796	0.997	ns
50	-2.33009545	104.44497	0.0217	0.062	ns	50	0.208244009	89.55211	0.836	0.997	ns
51	-2.48361989	98.06698	0.0147	0.049	*	51	0.17520396	89.41389	0.861	0.997	ns
52	-2.66139805	91.53749	0.00919	0.035346154	*	52	0.183280058	90.25867	0.855	0.997	ns
53	-2.81836847	86.31362	0.00598	0.027181818	*	53	0.206610816	91.78883	0.837	0.997	ns
54	-2.92827462	83.0688	0.0044	0.023947368	*	54	0.213299263	93.62268	0.832	0.997	ns
55	-2.96845404	81.55136	0.00393	0.023117647	*	55	0.190102955	96.03805	0.85	0.997	ns
56	-2.89868844	81.13395	0.00482	0.0241	*	56	0.127129047	98.76687	0.899	0.997	ns
57	-2.74512348	81.70744	0.00744	0.031	*	57	0.033237562	101.1167	0.974	0.997	ns
58	-2.55634621	83.01713	0.0124	0.044285714	*	58	-0.08324974	102.42134	0.934	0.997	ns
59	-2.35088265	84.37228	0.0211	0.062	ns	59	-0.228580152	102.77179	0.82	0.997	ns
60	-2.14069599	85.51799	0.0351	0.0675	ns	60	-0.386169717	102.75034	0.7	0.997	ns
61	-1.94017917	86.88381	0.0556	0.092666667	ns	61	-0.52377879	102.75046	0.602	0.997	ns
62	-1.76441412	88.55517	0.0811	0.12671875	ns	62	-0.625616744	103.0425	0.533	0.997	ns
63	-1.63658385	90.65745	0.105	0.156716418	ns	63	-0.684455745	104.04721	0.495	0.997	ns
64	-1.56926196	92.95473	0.12	0.171428571	ns	64	-0.706403631	105.52914	0.481	0.997	ns
65	-1.55624327	94.85867	0.123	0.173239437	ns	65	-0.699531212	106.50024	0.486	0.997	ns
66	-1.58890319	95.90531	0.115	0.166666667	ns	66	-0.669398212	106.24565	0.505	0.997	ns
67	-1.67136213	96.02698	0.0979	0.150615385	ns	67	-0.624069742	105.05635	0.534	0.997	ns
68	-1.79864438	95.9913	0.0752	0.119365079	ns	68	-0.550312945	103.42752	0.583	0.997	ns

69	-1.94260291	96.61866	0.055	0.092666667	ns	69	-0.446835675	101.88032	0.656	0.997	ns
70	-2.07386947	98.1307	0.0407	0.074	ns	70	-0.32703495	100.56802	0.744	0.997	ns
71	-2.20243159	100.85452	0.0299	0.065531915	ns	71	-0.210670356	99.34343	0.834	0.997	ns
72	-2.35479139	105.20949	0.0204	0.062	ns	72	-0.091898895	98.18516	0.927	0.997	ns
73	-2.50993351	110.42683	0.0135	0.046551724	*	73	0.019561626	97.30155	0.984	0.997	ns
74	-2.65021862	115.36777	0.00917	0.035346154	*	74	0.127670748	97.0218	0.899	0.997	ns
75	-2.76480981	119.29862	0.0066	0.028695652	*	75	0.221466685	97.30458	0.825	0.997	ns
76	-2.89097284	122.15223	0.00455	0.023947368	*	76	0.281141612	98.04439	0.779	0.997	ns
77	-3.0318559	123.86262	0.00296	0.0185	*	77	0.30332634	98.76918	0.762	0.997	ns
78	-3.15789465	124.34517	0.00199	0.013733333	*	78	0.289658059	99.01251	0.773	0.997	ns
79	-3.24517778	123.75125	0.00151	0.011615385	*	79	0.269768071	98.55467	0.788	0.997	ns
80	-3.28785395	122.3674	0.00132	0.011	*	80	0.262880863	97.52636	0.793	0.997	ns
81	-3.29357729	120.5679	0.0013	0.011	*	81	0.255944035	96.21891	0.799	0.997	ns
82	-3.30639479	118.60414	0.00125	0.011	*	82	0.236140308	94.82377	0.814	0.997	ns
83	-3.34692872	116.91777	0.0011	0.011	*	83	0.20680986	93.2513	0.837	0.997	ns
84	-3.42608174	115.90703	0.000848	0.0106	*	84	0.176859516	91.54376	0.86	0.997	ns
85	-3.55365916	115.89614	0.000551	0.009085714	**	85	0.149615784	90.12811	0.881	0.997	ns
86	-3.70781732	116.74879	0.000321	0.00642	**	86	0.101759414	89.08218	0.919	0.997	ns
87	-3.84636737	118.50656	0.000195	0.0061	**	87	0.039566193	88.37739	0.969	0.997	ns
88	-3.93047352	121.01463	0.000142	0.0061	**	88	0.01243899	88.19791	0.99	0.997	ns
89	-3.920083	123.52084	0.000146	0.0061	**	89	0.065146167	88.93369	0.948	0.997	ns
90	-3.7774784	125.33219	0.000244	0.0061	**	90	0.219822643	90.59702	0.827	0.997	ns
91	-3.50351119	126.25005	0.000636	0.009085714	**	91	0.472438554	92.68177	0.638	0.997	ns
92	-3.14730124	126.55038	0.00206	0.013733333	*	92	0.764359209	93.7786	0.447	0.997	ns
93	-2.81814075	126.42431	0.00561	0.026714286	*	93	1.017622103	93.86393	0.311	0.997	ns

94	-2.58330044	125.84038	0.0109	0.04037037	*	94	1.17326859	94.64176	0.244	0.997	ns
95	-2.43905026	124.88269	0.0161	0.051935484	ns	95	1.208170693	96.19078	0.23	0.997	ns
96	-2.34406515	123.66648	0.0207	0.062	ns	96	1.167181634	97.67959	0.246	0.997	ns
97	-2.25466411	122.04801	0.0259	0.065531915	ns	97	1.089523899	98.55331	0.279	0.997	ns
98	-2.13391818	119.62795	0.0349	0.0675	ns	98	0.994240805	99.70139	0.323	0.997	ns
99	-2.06174066	116.76761	0.0415	0.074107143	ns	99	0.913194336	101.64127	0.363	0.997	ns
100	-2.08252633	114.70507	0.0395	0.073148148	ns	100	0.912151268	103.34243	0.364	0.997	ns
3	statistic	df	p	p.adj	p.adj.signif	4	statistic	df	p	p.adj	p.adj.signif
1	0.66625092	126.782	5.06E-01	5.62E-01	ns	1	0.976357521	127.96035	0.331	0.999	ns
2	0.67931089	127.3265	4.98E-01	5.60E-01	ns	2	0.806517359	127.93685	0.421	0.999	ns
3	0.6231407	127.4326	5.34E-01	5.80E-01	ns	3	0.729427958	127.85143	0.467	0.999	ns
4	0.54056146	127.6054	5.90E-01	6.34E-01	ns	4	0.780842285	127.88299	0.436	0.999	ns
5	0.41997653	127.6801	6.75E-01	7.11E-01	ns	5	0.946710269	127.99732	0.346	0.999	ns
6	0.27378151	127.6261	7.85E-01	8.09E-01	ns	6	1.07490103	127.82038	0.284	0.999	ns
7	0.12728781	127.6498	8.99E-01	9.08E-01	ns	7	1.069490065	127.7916	0.287	0.999	ns
8	-0.02046111	127.5778	9.84E-01	9.84E-01	ns	8	1.019146771	127.99992	0.31	0.999	ns
9	-0.17451958	127.2907	8.62E-01	8.80E-01	ns	9	0.896233022	127.46722	0.372	0.999	ns
10	-0.33009258	126.8782	7.42E-01	7.73E-01	ns	10	0.736541901	125.6385	0.463	0.999	ns
11	-0.4823326	126.251	6.30E-01	6.70E-01	ns	11	0.553496159	122.96591	0.581	0.999	ns
12	-0.64167401	125.5718	5.22E-01	5.74E-01	ns	12	0.387841032	120.69742	0.699	0.999	ns
13	-0.75933366	124.8232	4.49E-01	5.10E-01	ns	13	0.253902726	119.54985	0.8	0.999	ns
14	-0.79348077	123.7511	4.29E-01	4.93E-01	ns	14	0.127491706	119.34904	0.899	0.999	ns
15	-0.81758457	122.3678	4.15E-01	4.83E-01	ns	15	-0.009827175	119.77375	0.992	0.999	ns
16	-0.90004573	120.5177	3.70E-01	4.35E-01	ns	16	-0.110217702	120.5873	0.912	0.999	ns
17	-1.08286104	119.1638	2.81E-01	3.35E-01	ns	17	-0.176861449	121.72535	0.86	0.999	ns

18	-1.38397653	119.551	1.69E-01	2.04E-01	ns	18	-0.250793348	122.93785	0.802	0.999	ns
19	-1.80631668	121.0205	7.34E-02	8.95E-02	ns	19	-0.433602341	123.75009	0.665	0.999	ns
20	-2.27434408	122.6599	2.47E-02	3.09E-02	*	20	-0.584789247	123.92316	0.56	0.999	ns
21	-2.68760914	124.3421	8.18E-03	1.11E-02	*	21	-0.690862489	123.77911	0.491	0.999	ns
22	-2.964885	125.5944	3.62E-03	5.10E-03	**	22	-0.791877734	123.99587	0.43	0.999	ns
23	-3.13862635	126.253	2.11E-03	3.10E-03	**	23	-0.827367061	124.33073	0.41	0.999	ns
24	-3.22109858	126.2951	1.62E-03	2.57E-03	**	24	-0.719881622	124.3613	0.473	0.999	ns
25	-3.19623277	125.7132	1.76E-03	2.71E-03	**	25	-0.490776543	122.90545	0.624	0.999	ns
26	-3.20880121	125.0281	1.69E-03	2.64E-03	**	26	-0.209932808	120.84319	0.834	0.999	ns
27	-3.32020771	124.2168	1.18E-03	2.05E-03	**	27	0.021875337	119.11865	0.983	0.999	ns
28	-3.4992124	122.9165	6.51E-04	1.23E-03	**	28	0.266862119	119.39495	0.79	0.999	ns
29	-3.68634179	121.0934	3.42E-04	6.98E-04	***	29	0.410042263	119.08008	0.683	0.999	ns
30	-3.81787272	118.7762	2.15E-04	4.89E-04	***	30	0.350032369	118.08146	0.727	0.999	ns
31	-3.87286176	116.1548	1.78E-04	4.45E-04	***	31	0.139392188	116.88334	0.889	0.999	ns
32	-3.86362553	113.7541	1.86E-04	4.54E-04	***	32	-0.06609791	114.36804	0.947	0.999	ns
33	-3.84770789	111.1018	1.99E-04	4.74E-04	***	33	-0.180353328	110.15273	0.857	0.999	ns
34	-3.89058967	108.4615	1.73E-04	4.45E-04	***	34	-0.220928902	107.19277	0.826	0.999	ns
35	-4.04250125	106.9577	1.00E-04	3.23E-04	***	35	-0.238616522	107.09379	0.812	0.999	ns
36	-4.33589294	107.3041	3.29E-05	1.61E-04	***	36	-0.21683937	108.8856	0.829	0.999	ns
37	-4.7243643	109.3872	6.90E-06	7.67E-05	****	37	-0.148207999	110.69617	0.882	0.999	ns
38	-5.08450961	112.5369	1.48E-06	2.47E-05	****	38	-0.077428519	111.1099	0.938	0.999	ns
39	-5.31442216	114.6736	5.33E-07	1.33E-05	****	39	-0.04112954	109.9727	0.967	0.999	ns
40	-5.38411965	115.5546	3.87E-07	1.33E-05	****	40	-0.010120259	109.67336	0.992	0.999	ns
41	-5.36180236	117.7927	4.16E-07	1.33E-05	****	41	-0.011427003	110.31133	0.991	0.999	ns
42	-5.32501191	122.2286	4.66E-07	1.33E-05	****	42	-0.076754634	110.97841	0.939	0.999	ns

43	-5.19623064	125.4687	7.99E-07	1.60E-05	****	43	-0.163192384	111.8631	0.871	0.999	ns
44	-4.95648273	126.8305	2.25E-06	3.21E-05	****	44	-0.184488644	112.31287	0.854	0.999	ns
45	-4.70126209	127.1354	6.62E-06	7.67E-05	****	45	-0.090631005	112.15618	0.928	0.999	ns
46	-4.48005695	126.9291	1.65E-05	1.58E-04	***	46	0.032192021	110.80226	0.974	0.999	ns
47	-4.30341799	126.0647	3.34E-05	1.61E-04	***	47	0.165805012	108.71623	0.869	0.999	ns
48	-4.20978466	124.3956	4.86E-05	1.79E-04	***	48	0.289237746	105.94083	0.773	0.999	ns
49	-4.20566327	122.7884	4.97E-05	1.79E-04	***	49	0.389710276	101.98161	0.698	0.999	ns
50	-4.27089501	122.5136	3.87E-05	1.61E-04	***	50	0.45725609	97.96863	0.648	0.999	ns
51	-4.31560602	123.261	3.23E-05	1.61E-04	***	51	0.473784462	94.87227	0.637	0.999	ns
52	-4.24565091	124.5756	4.22E-05	1.69E-04	***	52	0.453849777	93.00822	0.651	0.999	ns
53	-4.04675167	125.5301	9.02E-05	3.01E-04	***	53	0.423477611	92.20394	0.673	0.999	ns
54	-3.77999017	125.8754	2.41E-04	5.36E-04	***	54	0.386941129	92.56044	0.7	0.999	ns
55	-3.54465688	125.7708	5.53E-04	1.06E-03	**	55	0.391428176	94.96998	0.696	0.999	ns
56	-3.38271602	124.8925	9.59E-04	1.74E-03	**	56	0.442902587	98.81461	0.659	0.999	ns
57	-3.31332575	123.3535	1.21E-03	2.05E-03	**	57	0.530308242	102.59787	0.597	0.999	ns
58	-3.30498447	121.3263	1.25E-03	2.08E-03	**	58	0.629385996	104.42485	0.53	0.999	ns
59	-3.31538879	119.1422	1.21E-03	2.05E-03	**	59	0.705810464	102.95993	0.482	0.999	ns
60	-3.30200866	117.7766	1.27E-03	2.08E-03	**	60	0.785190457	98.33557	0.434	0.999	ns
61	-3.25963286	117.6043	1.46E-03	2.35E-03	**	61	0.828576889	93.46583	0.409	0.999	ns
62	-3.15674296	117.2975	2.03E-03	3.03E-03	**	62	0.823417897	90.0652	0.412	0.999	ns
63	-2.97448145	116.0694	3.57E-03	5.10E-03	**	63	0.793704665	88.50729	0.429	0.999	ns
64	-2.77737264	114.2582	6.41E-03	8.78E-03	**	64	0.757154633	88.67517	0.451	0.999	ns
65	-2.62626453	112.7553	9.83E-03	1.29E-02	*	65	0.704459367	89.6312	0.483	0.999	ns
66	-2.57440527	112.3353	1.13E-02	1.45E-02	*	66	0.645006647	90.80758	0.521	0.999	ns
67	-2.6682409	112.8735	8.75E-03	1.17E-02	*	67	0.584819315	92.0196	0.56	0.999	ns

68	-2.90894237	113.568	4.36E-03	6.06E-03	**	68	0.577317344	92.34402	0.565	0.999	ns
69	-3.19328441	113.3722	1.82E-03	2.76E-03	**	69	0.608462731	92.62039	0.544	0.999	ns
70	-3.45129669	112.845	7.86E-04	1.46E-03	**	70	0.602819487	94.49515	0.548	0.999	ns
71	-3.63241569	112.3989	4.25E-04	8.50E-04	***	71	0.541237092	98.45	0.59	0.999	ns
72	-3.76394033	112.449	2.68E-04	5.70E-04	***	72	0.437478394	101.87619	0.663	0.999	ns
73	-3.93101633	113.1341	1.46E-04	4.17E-04	***	73	0.320012443	102.9136	0.75	0.999	ns
74	-4.11030407	113.4618	7.50E-05	2.59E-04	***	74	0.202471791	102.3538	0.84	0.999	ns
75	-4.28703783	113.7653	3.81E-05	1.61E-04	***	75	0.072145074	101.8691	0.943	0.999	ns
76	-4.41898399	114.5855	2.26E-05	1.61E-04	***	76	0.001020839	101.48431	0.999	0.999	ns
77	-4.44078499	116.1654	2.05E-05	1.58E-04	***	77	-0.003104362	101.19602	0.998	0.999	ns
78	-4.36488545	118.2106	2.74E-05	1.61E-04	***	78	-0.002078135	100.85039	0.998	0.999	ns
79	-4.27570598	120.1817	3.84E-05	1.61E-04	***	79	-0.037419014	100.82255	0.97	0.999	ns
80	-4.27787379	121.8967	3.78E-05	1.61E-04	***	80	-0.084831573	100.84066	0.933	0.999	ns
81	-4.36382291	122.7044	2.68E-05	1.61E-04	***	81	-0.106392119	100.69116	0.915	0.999	ns
82	-4.44678595	121.8944	1.94E-05	1.58E-04	***	82	-0.071548841	100.9872	0.943	0.999	ns
83	-4.47487822	120.0843	1.75E-05	1.58E-04	***	83	-0.00984009	101.81498	0.992	0.999	ns
84	-4.39302247	118.988	2.44E-05	1.61E-04	***	84	0.060222533	102.10718	0.952	0.999	ns
85	-4.20834298	118.7396	5.02E-05	1.79E-04	***	85	0.145501137	101.04647	0.885	0.999	ns
86	-4.00736573	118.6342	1.08E-04	3.38E-04	***	86	0.255312338	99.54179	0.799	0.999	ns
87	-3.8902974	118.661	1.66E-04	4.45E-04	***	87	0.399503323	98.60761	0.69	0.999	ns
88	-3.87628478	118.9232	1.74E-04	4.45E-04	***	88	0.602311969	98.72197	0.548	0.999	ns
89	-3.92922782	118.2814	1.44E-04	4.17E-04	***	89	0.859550963	99.95017	0.392	0.999	ns
90	-3.95448899	115.7176	1.33E-04	4.03E-04	***	90	1.074545401	101.5121	0.285	0.999	ns
91	-3.90650649	112.1311	1.60E-04	4.44E-04	***	91	1.178629587	103.35002	0.241	0.999	ns
92	-3.8376154	109.7481	2.08E-04	4.84E-04	***	92	1.150591716	104.60852	0.253	0.999	ns

93	-3.77522288	108.8982	2.61E-04	5.67E-04	***	93	0.99157798	103.89451	0.324	0.999	ns
94	-3.70494892	108.8075	3.34E-04	6.96E-04	***	94	0.766124256	101.69149	0.445	0.999	ns
95	-3.58987869	108.8523	4.98E-04	9.76E-04	***	95	0.559288077	99.1602	0.577	0.999	ns
96	-3.36532663	108.953	1.06E-03	1.89E-03	**	96	0.436458963	97.17472	0.663	0.999	ns
97	-3.00549034	109.3243	3.29E-03	4.77E-03	**	97	0.431127872	97.31037	0.667	0.999	ns
98	-2.59477923	109.753	1.08E-02	1.40E-02	*	98	0.487571193	100.04606	0.627	0.999	ns
99	-2.28070517	110.0078	2.45E-02	3.09E-02	*	99	0.616353007	104.11694	0.539	0.999	ns
100	-2.14243096	110.3672	3.44E-02	4.25E-02	*	100	0.814609601	107.07693	0.417	0.999	ns
5	statistic	df	p	p.adj	p.adj.signif	6	statistic	df	p	p.adj	p.adj.signif
1	0.42797726	100.6177	6.70E-01	0.85	ns	1	-0.31122137	105.34093	0.756	0.991	ns
2	0.34943536	100.78105	7.27E-01	0.85	ns	2	0.03174757	107.29841	0.975	0.991	ns
3	-0.14285885	99.65068	8.87E-01	0.908163265	ns	3	0.49352013	105.0419	0.623	0.991	ns
4	-0.63503573	98.89699	5.27E-01	0.802941177	ns	4	0.78845523	105.96434	0.432	0.9644444	ns
5	-0.89049982	96.86639	3.75E-01	0.638983051	ns	5	0.79518171	102.90141	0.428	0.9644444	ns
6	-0.99286343	96.54108	3.23E-01	0.622641509	ns	6	0.54414657	99.38632	0.588	0.991	ns
7	-0.98442887	99.77722	3.27E-01	0.622641509	ns	7	0.37298877	98.85289	0.71	0.991	ns
8	-0.8366357	103.97109	4.05E-01	0.663934426	ns	8	0.37959241	101.92293	0.705	0.991	ns
9	-0.67397339	105.38056	5.02E-01	0.784375	ns	9	0.38027068	104.17382	0.705	0.991	ns
10	-0.62666138	104.2724	5.32E-01	0.802941177	ns	10	0.05932342	105.61338	0.953	0.991	ns
11	-0.61035076	102.18734	5.43E-01	0.802941177	ns	11	-0.36625642	107.58348	0.715	0.991	ns
12	-0.46340418	100.15404	6.44E-01	0.848684211	ns	12	-0.70578683	109.51053	0.482	0.988	ns
13	-0.37560357	100.15589	7.08E-01	0.85	ns	13	-0.9009745	110.54544	0.37	0.9644444	ns
14	-0.40859512	103.09118	6.84E-01	0.85	ns	14	-0.83047622	110.83571	0.408	0.9644444	ns
15	-0.47812395	106.84551	6.34E-01	0.848684211	ns	15	-0.57920204	111.06114	0.564	0.991	ns
16	-0.52272643	108.81436	6.02E-01	0.848684211	ns	16	-0.30824207	111.58601	0.758	0.991	ns

17	-0.60609496	107.79483	5.46E-01	0.802941177	ns	17	0.02904842	110.28203	0.977	0.991	ns
18	-0.72983501	103.72533	4.67E-01	0.753225807	ns	18	0.48204311	107.53682	0.631	0.991	ns
19	-0.89684119	98.94203	3.72E-01	0.638983051	ns	19	0.99161907	105.74954	0.324	0.9644444	ns
20	-1.01369908	96.92654	3.13E-01	0.622641509	ns	20	1.35445631	103.33448	0.179	0.8136364	ns
21	-1.12137299	97.10206	2.65E-01	0.552083333	ns	21	1.49475336	105.98504	0.138	0.69	ns
22	-1.23680825	100.71704	2.19E-01	0.465957447	ns	22	1.37690927	109.77613	0.171	0.8136364	ns
23	-1.43872405	105.36261	1.53E-01	0.373170732	ns	23	0.94416421	111.52404	0.347	0.9644444	ns
24	-1.73348564	108.5832	8.58E-02	0.225789474	ns	24	0.44676337	110.30823	0.656	0.991	ns
25	-2.05014349	109.65008	4.27E-02	0.125588235	ns	25	0.04137274	108.20017	0.967	0.991	ns
26	-2.33559847	110.30406	2.13E-02	0.068709677	ns	26	-0.11077486	107.36796	0.912	0.991	ns
27	-2.6768365	111.0039	8.56E-03	0.035666667	*	27	-0.16425399	107.60748	0.87	0.991	ns
28	-2.94547333	112.29151	3.92E-03	0.021777778	*	28	-0.1481135	109.62932	0.883	0.991	ns
29	-3.05114417	114.58044	2.83E-03	0.01875	*	29	0.01944804	111.6807	0.985	0.991	ns
30	-2.83296549	116.61078	5.44E-03	0.025904762	*	30	0.27617889	111.89535	0.783	0.991	ns
31	-2.43854513	114.53409	1.63E-02	0.056206897	ns	31	0.37329591	111.78416	0.71	0.991	ns
32	-1.9495977	111.01218	5.37E-02	0.149166667	ns	32	0.25365267	112.65135	0.8	0.991	ns
33	-1.62274689	109.7502	1.08E-01	0.27	ns	33	0.04287629	114.14371	0.966	0.991	ns
34	-1.40521624	111.39718	1.63E-01	0.388095238	ns	34	-0.07351493	116.3471	0.942	0.991	ns
35	-1.2758362	114.44928	2.05E-01	0.455555556	ns	35	-0.11077207	118.83526	0.912	0.991	ns
36	-1.25804973	116.93685	2.11E-01	0.458695652	ns	36	-0.17909289	121.20423	0.858	0.991	ns
37	-1.39171842	118.72969	1.67E-01	0.388372093	ns	37	-0.4218977	121.30352	0.674	0.991	ns
38	-1.68323027	118.34816	9.50E-02	0.243589744	ns	38	-0.69290273	119.95591	0.49	0.988	ns
39	-1.9752449	115.87126	5.06E-02	0.144571429	ns	39	-0.85700547	117.17157	0.393	0.9644444	ns
40	-2.19093155	113.27993	3.05E-02	0.092424242	ns	40	-0.88200157	112.80464	0.38	0.9644444	ns
41	-2.36508608	114.27912	1.97E-02	0.065666667	ns	41	-0.8198524	109.94657	0.414	0.9644444	ns

42	-2.53601513	118.99126	1.25E-02	0.046296296	*	42	-0.80115441	111.22094	0.425	0.9644444	ns
43	-2.67719338	123.27456	8.44E-03	0.035666667	*	43	-0.85305392	115.23963	0.395	0.9644444	ns
44	-2.9053877	125.85434	4.33E-03	0.022789474	*	44	-1.00895099	118.711	0.315	0.9644444	ns
45	-3.3393813	126.54965	1.10E-03	0.012	*	45	-1.27906977	121.68653	0.203	0.8826087	ns
46	-3.88084827	125.66934	1.67E-04	0.002933333	**	46	-1.52691048	123.93544	0.129	0.6789474	ns
47	-4.35338291	122.26165	2.80E-05	0.000933333	***	47	-1.62102829	124.2781	0.108	0.6529412	ns
48	-4.54731667	116.60108	1.34E-05	0.000933333	***	48	-1.64682939	123.36924	0.102	0.6529412	ns
49	-4.44831502	112.60782	2.04E-05	0.000933333	***	49	-1.60583327	119.7649	0.111	0.6529412	ns
50	-4.1721774	111.13242	6.01E-05	0.0015025	**	50	-1.56364177	112.33329	0.121	0.6722222	ns
51	-3.8847717	109.74387	1.76E-04	0.002933333	**	51	-1.64227439	104.10208	0.104	0.6529412	ns
52	-3.627738	107.97972	4.38E-04	0.006257143	**	52	-1.76027287	99.64943	0.0814	0.6529412	ns
53	-3.41840979	108.29189	8.89E-04	0.0111125	*	53	-1.84981097	99.97265	0.0673	0.6118182	ns
54	-3.23541798	110.98524	1.60E-03	0.013666667	*	54	-1.89841385	102.90068	0.0604	0.604	ns
55	-3.03283133	114.43684	3.00E-03	0.01875	*	55	-1.96601316	106.71649	0.0519	0.5766667	ns
56	-2.94752358	117.94615	3.86E-03	0.021777778	*	56	-2.08308045	109.32155	0.0396	0.53875	ns
57	-3.03037433	121.52961	2.98E-03	0.01875	*	57	-2.25085957	111.2411	0.0264	0.44	ns
58	-3.21879556	123.71245	1.64E-03	0.013666667	*	58	-2.4529774	111.78053	0.0157	0.3925	ns
59	-3.31466406	124.54803	1.20E-03	0.012	*	59	-2.55224283	109.61135	0.0121	0.3925	ns
60	-3.15711486	123.58826	2.00E-03	0.015384615	*	60	-2.5583926	105.16451	0.0119	0.3925	ns
61	-2.88725024	120.26065	4.61E-03	0.02305	*	61	-2.51356096	100.30376	0.0135	0.3925	ns
62	-2.67902524	114.62995	8.47E-03	0.035666667	*	62	-2.34899543	96.48274	0.0209	0.418	ns
63	-2.62843338	108.32642	9.82E-03	0.03928	*	63	-2.05062118	92.9917	0.0431	0.53875	ns
64	-2.61128049	103.04884	1.04E-02	0.04	*	64	-1.65265485	90.67527	0.102	0.6529412	ns
65	-2.49387315	97.41563	1.43E-02	0.051071429	ns	65	-1.18440589	89.11621	0.239	0.9644444	ns
66	-2.21816945	92.43837	2.90E-02	0.090625	ns	66	-0.66594046	88.32534	0.507	0.991	ns

67	-1.83563191	90.54473	6.97E-02	0.188378378	ns	67	-0.16939563	88.21195	0.866	0.991	ns
68	-1.33542849	93.34026	1.85E-01	0.420454546	ns	68	0.17394038	90.27868	0.862	0.991	ns
69	-0.85497985	98.30017	3.95E-01	0.658333333	ns	69	0.39446182	93.09252	0.694	0.991	ns
70	-0.51453756	102.27125	6.08E-01	0.848684211	ns	70	0.49431325	93.77951	0.622	0.991	ns
71	-0.37576466	104.06559	7.08E-01	0.85	ns	71	0.55029995	92.02519	0.583	0.991	ns
72	-0.37404805	101.97188	7.09E-01	0.85	ns	72	0.42067082	91.08231	0.675	0.991	ns
73	-0.41677248	96.1778	6.78E-01	0.85	ns	73	0.15611749	92.55434	0.876	0.991	ns
74	-0.34492283	91.2521	7.31E-01	0.85	ns	74	-0.06761925	94.16638	0.946	0.991	ns
75	-0.13919869	89.04702	8.90E-01	0.908163265	ns	75	-0.1787557	96.59919	0.859	0.991	ns
76	-0.06291476	89.12059	9.50E-01	0.95	ns	76	-0.28838922	100.39566	0.774	0.991	ns
77	-0.15659643	90.05949	8.76E-01	0.908163265	ns	77	-0.42024829	102.96499	0.675	0.991	ns
78	-0.26076345	92.97038	7.95E-01	0.869565217	ns	78	-0.44515444	102.89007	0.657	0.991	ns
79	-0.13893851	97.56304	8.90E-01	0.908163265	ns	79	-0.30170679	102.40731	0.763	0.991	ns
80	0.09056611	102.51287	9.28E-01	0.937373737	ns	80	-0.01170565	102.53174	0.991	0.991	ns
81	0.3144826	104.03283	7.54E-01	0.856179775	ns	81	0.29173399	106.11171	0.771	0.991	ns
82	0.46187051	102.81474	6.45E-01	0.848684211	ns	82	0.51212793	109.53447	0.61	0.991	ns
83	0.48076325	101.5506	6.32E-01	0.848684211	ns	83	0.68674653	111.71647	0.494	0.988	ns
84	0.48280008	100.47077	6.30E-01	0.848684211	ns	84	0.78473076	113.25516	0.434	0.9644444	ns
85	0.40885485	99.34244	6.84E-01	0.85	ns	85	0.69774205	111.61404	0.487	0.988	ns
86	0.25347675	99.45576	8.00E-01	0.869565217	ns	86	0.49842052	108.28197	0.619	0.991	ns
87	0.17926031	101.80127	8.58E-01	0.908163265	ns	87	0.27527591	103.85475	0.784	0.991	ns
88	0.21774602	104.42956	8.28E-01	0.890322581	ns	88	0.05257449	99.77716	0.958	0.991	ns
89	0.27602342	106.93867	7.83E-01	0.869565217	ns	89	-0.20152293	96.54429	0.841	0.991	ns
90	0.30325856	108.70778	7.62E-01	0.856179775	ns	90	-0.38933449	96.03438	0.698	0.991	ns
91	0.32295659	108.01728	7.47E-01	0.856179775	ns	91	-0.54117651	98.56281	0.59	0.991	ns

92	0.35199847	106.83176	7.26E-01	0.85	ns	92	-0.72005588	101.85634	0.473	0.988	ns
93	0.47061348	106.63413	6.39E-01	0.848684211	ns	93	-0.92282146	103.10746	0.358	0.9644444	ns
94	0.71394288	109.59514	4.77E-01	0.757142857	ns	94	-1.05270102	104.21913	0.295	0.9644444	ns
95	0.93333503	114.88376	3.53E-01	0.635714286	ns	95	-1.12157787	106.04283	0.265	0.9644444	ns
96	0.97742845	117.47277	3.30E-01	0.622641509	ns	96	-1.10905536	108.17904	0.27	0.9644444	ns
97	0.92695074	116.42939	3.56E-01	0.635714286	ns	97	-1.08275494	109.24248	0.281	0.9644444	ns
98	0.88657864	113.19688	3.77E-01	0.638983051	ns	98	-1.05964751	107.95574	0.292	0.9644444	ns
99	0.94509507	109.80885	3.47E-01	0.635714286	ns	99	-1.02133958	105.69258	0.309	0.9644444	ns
100	1.00461782	107.62361	3.17E-01	0.622641509	ns	100	-0.95593037	103.93617	0.341	0.9644444	ns
7	statistic	df	p	p.adj	p.adj.signif	8	statistic	df	p	p.adj	p.adj.signif
1	-4.06561875	113.79721	8.85E-05	0.000966667	***	1	-0.422291582	106.13414	0.674	0.98556701	ns
2	-3.99369451	112.57485	1.16E-04	0.000966667	***	2	-0.365674542	104.13731	0.715	0.98556701	ns
3	-3.93331671	112.4383	1.45E-04	0.001035714	**	3	-0.484633433	101.76358	0.629	0.98556701	ns
4	-3.88567532	112.94781	1.73E-04	0.001136842	**	4	-0.679121046	98.94508	0.499	0.98556701	ns
5	-3.84566065	114.17903	1.98E-04	0.001136842	**	5	-0.798093443	97.00919	0.427	0.98556701	ns
6	-3.82625173	116.12616	2.11E-04	0.001136842	**	6	-0.799588112	95.96375	0.426	0.98556701	ns
7	-3.81847493	117.7887	2.16E-04	0.001136842	**	7	-0.705791735	95.30642	0.482	0.98556701	ns
8	-3.76477583	119.18901	2.60E-04	0.001238095	**	8	-0.531067785	95.14969	0.597	0.98556701	ns
9	-3.71074104	119.53517	3.15E-04	0.001369565	**	9	-0.32613478	95.88184	0.745	0.98556701	ns
10	-3.69162544	118.92652	3.38E-04	0.001408333	**	10	-0.175662316	97.85265	0.861	0.98556701	ns
11	-3.74133322	117.47174	2.85E-04	0.001295455	**	11	-0.103048088	100.71528	0.918	0.98556701	ns
12	-3.84135242	115.54431	2.00E-04	0.001136842	**	12	-0.089999302	103.40215	0.928	0.98556701	ns
13	-4.00719937	114.18829	1.10E-04	0.000966667	***	13	-0.139514407	107.02932	0.889	0.98556701	ns
14	-4.17003482	112.92246	6.00E-05	0.000922857	***	14	-0.231697876	110.68723	0.817	0.98556701	ns
15	-4.26844229	111.39552	4.15E-05	0.000922857	***	15	-0.300039523	112.78698	0.765	0.98556701	ns

16	-4.29198747	109.82897	3.83E-05	0.000922857	***	16	-0.320068417	113.64207	0.75	0.98556701	ns
17	-4.30031755	109.39071	3.72E-05	0.000922857	***	17	-0.283241342	114.38557	0.778	0.98556701	ns
18	-4.28522297	109.06278	3.95E-05	0.000922857	***	18	-0.236476086	114.87786	0.813	0.98556701	ns
19	-4.20437991	108.44834	5.40E-05	0.000922857	***	19	-0.178285271	114.65172	0.859	0.98556701	ns
20	-4.15754066	108.2574	6.46E-05	0.000922857	***	20	-0.123429153	114.89722	0.902	0.98556701	ns
21	-4.11535194	108.72463	7.55E-05	0.00094375	***	21	-0.055169538	115.23337	0.956	0.98556701	ns
22	-4.04625118	110.13647	9.69E-05	0.000966667	***	22	0.032576859	115.34612	0.974	0.993	ns
23	-3.9459482	111.4922	1.39E-04	0.001035714	**	23	0.112200445	115.09578	0.911	0.98556701	ns
24	-3.77804355	113.21951	2.54E-04	0.001238095	**	24	0.133974406	115.1303	0.894	0.98556701	ns
25	-3.59137127	116.18119	4.83E-04	0.001932	**	25	0.117410955	114.97487	0.907	0.98556701	ns
26	-3.42762053	120.28686	8.34E-04	0.003207692	**	26	0.07923048	115.49149	0.937	0.98556701	ns
27	-3.23656467	123.78777	1.55E-03	0.005740741	**	27	0.008197205	116.04725	0.993	0.993	ns
28	-3.00090056	126.09637	3.25E-03	0.011607143	*	28	-0.159527325	116.42868	0.874	0.98556701	ns
29	-2.76481281	127.05446	6.54E-03	0.017394737	*	29	-0.324446785	116.16265	0.746	0.98556701	ns
30	-2.54740168	127.24595	1.20E-02	0.027906977	*	30	-0.448907124	113.96586	0.654	0.98556701	ns
31	-2.35705979	126.97209	1.99E-02	0.041458333	*	31	-0.511477644	110.31211	0.61	0.98556701	ns
32	-2.20803536	126.0912	2.90E-02	0.054716981	ns	32	-0.586707265	106.55322	0.559	0.98556701	ns
33	-2.14750249	124.44327	3.37E-02	0.060178571	ns	33	-0.599490339	103.79044	0.55	0.98556701	ns
34	-2.18977628	121.74281	3.04E-02	0.055272727	ns	34	-0.60593881	101.47829	0.546	0.98556701	ns
35	-2.3129107	118.61097	2.25E-02	0.044615385	*	35	-0.663360116	100.73098	0.509	0.98556701	ns
36	-2.47255015	115.90526	1.49E-02	0.032391304	*	36	-0.684837786	101.28148	0.495	0.98556701	ns
37	-2.63003127	112.60879	9.73E-03	0.024390244	*	37	-0.61597964	102.12701	0.539	0.98556701	ns
38	-2.78081831	108.68199	6.39E-03	0.017394737	*	38	-0.523156487	101.81118	0.602	0.98556701	ns
39	-2.88661792	105.10046	4.73E-03	0.01478125	*	39	-0.467110518	99.85448	0.641	0.98556701	ns
40	-2.91112953	101.81141	4.42E-03	0.014258065	*	40	-0.515886051	98.05387	0.607	0.98556701	ns

41	-2.79621071	98.4725	6.22E-03	0.017394737	*	41	-0.644998387	97.35611	0.52	0.98556701	ns
42	-2.62670182	95.4732	1.00E-02	0.024390244	*	42	-0.809110883	97.95502	0.42	0.98556701	ns
43	-2.51296139	94.50421	1.37E-02	0.031136364	*	43	-0.955837782	98.66239	0.341	0.94722222	ns
44	-2.43056889	94.54055	1.70E-02	0.036170213	*	44	-1.047898727	99.37809	0.297	0.9	ns
45	-2.33042937	94.04743	2.19E-02	0.044615385	*	45	-1.088344808	100.47633	0.279	0.871875	ns
46	-2.20874883	93.21536	2.96E-02	0.054814815	ns	46	-1.107662524	101.30237	0.271	0.871875	ns
47	-2.08471313	92.95105	3.98E-02	0.06862069	ns	47	-1.106518079	100.45627	0.271	0.871875	ns
48	-1.99964851	92.91055	4.85E-02	0.080833333	ns	48	-1.026362621	98.47091	0.307	0.90294118	ns
49	-1.92355902	92.99163	5.75E-02	0.093064516	ns	49	-0.898047735	95.77934	0.371	0.98556701	ns
50	-1.88404839	92.92353	6.27E-02	0.09796875	ns	50	-0.843486152	94.81567	0.401	0.98556701	ns
51	-1.92176287	92.44751	5.77E-02	0.093064516	ns	51	-0.84473377	94.51918	0.4	0.98556701	ns
52	-2.06808529	92.33236	4.14E-02	0.070169492	ns	52	-0.835938605	93.29107	0.405	0.98556701	ns
53	-2.31464133	93.94244	2.28E-02	0.044615385	*	53	-0.785813631	91.75209	0.434	0.98556701	ns
54	-2.57945305	96.48222	1.14E-02	0.027142857	*	54	-0.705531323	90.36287	0.482	0.98556701	ns
55	-2.78863849	98.74976	6.35E-03	0.017394737	*	55	-0.594756976	90.19167	0.553	0.98556701	ns
56	-2.92608945	100.63641	4.24E-03	0.014133333	*	56	-0.485609619	91.85177	0.628	0.98556701	ns
57	-2.93980462	101.9747	4.06E-03	0.014	*	57	-0.363722631	94.32492	0.717	0.98556701	ns
58	-2.87184324	102.73334	4.96E-03	0.015030303	*	58	-0.214661211	96.23893	0.83	0.98556701	ns
59	-2.77219539	103.24757	6.61E-03	0.017394737	*	59	-0.06015731	97.97727	0.952	0.98556701	ns
60	-2.62981567	103.21719	9.85E-03	0.024390244	*	60	0.06634622	99.35719	0.947	0.98556701	ns
61	-2.47854568	102.13029	1.48E-02	0.032391304	*	61	0.143418142	101.25519	0.886	0.98556701	ns
62	-2.3060687	99.63381	2.32E-02	0.044615385	*	62	0.152046881	104.09097	0.879	0.98556701	ns
63	-2.11928878	95.98076	3.66E-02	0.064210526	ns	63	0.086163961	107.16621	0.931	0.98556701	ns
64	-1.8911063	92.11167	6.18E-02	0.09796875	ns	64	-0.016844007	109.7627	0.987	0.993	ns
65	-1.60110027	88.59717	1.13E-01	0.173846154	ns	65	-0.1009737	112.1559	0.92	0.98556701	ns

66	-1.27262291	85.74426	2.07E-01	0.313636364	ns	66	-0.148520386	114.36599	0.882	0.98556701	ns
67	-0.92537248	83.24419	3.57E-01	0.525	ns	67	-0.110772084	116.22696	0.912	0.98556701	ns
68	-0.60355411	80.92282	5.48E-01	0.685	ns	68	0.060438611	117.36551	0.952	0.98556701	ns
69	-0.3361967	78.96793	7.38E-01	0.829213483	ns	69	0.367208565	118.20093	0.714	0.98556701	ns
70	-0.11799744	77.83059	9.06E-01	0.933673469	ns	70	0.737517692	118.58151	0.462	0.98556701	ns
71	0.04335695	77.65645	9.66E-01	0.967	ns	71	1.095614557	118.1818	0.275	0.871875	ns
72	0.21177485	78.11673	8.33E-01	0.876842105	ns	72	1.440541881	117.60641	0.152	0.608	ns
73	0.40195209	78.47999	6.89E-01	0.810588235	ns	73	1.791569612	117.24358	0.0758	0.35818182	ns
74	0.60690809	78.16689	5.46E-01	0.685	ns	74	2.14933276	117.24375	0.0337	0.18473684	ns
75	0.77753112	77.37645	4.39E-01	0.590666667	ns	75	2.452974344	117.97084	0.0156	0.0975	ns
76	0.86888034	76.69336	3.88E-01	0.546478873	ns	76	2.719200534	119.87645	0.00752	0.05371429	ns
77	0.88888444	76.80587	3.77E-01	0.538571429	ns	77	2.968183067	122.93435	0.0036	0.03272727	*
78	0.8410059	77.85029	4.03E-01	0.559722222	ns	78	3.197776296	125.66219	0.00175	0.021875	*
79	0.77015803	79.71202	4.43E-01	0.590666667	ns	79	3.413421393	127.04859	0.000861	0.01475	*
80	0.70477945	83.53515	4.83E-01	0.635526316	ns	80	3.585272544	127.64881	0.000478	0.0134	*
81	0.6382211	89.021	5.25E-01	0.673076923	ns	81	3.673065883	127.91386	0.000351	0.0134	*
82	0.5575203	94.8791	5.78E-01	0.713580247	ns	82	3.65183089	127.98244	0.000378	0.0134	*
83	0.4757646	100.72557	6.35E-01	0.774390244	ns	83	3.552079073	127.996	0.000536	0.0134	*
84	0.41269433	107.13505	6.81E-01	0.810588235	ns	84	3.404595287	127.97801	0.000885	0.01475	*
85	0.38566556	112.64446	7.00E-01	0.813953488	ns	85	3.245639247	127.84438	0.0015	0.02142857	*
86	0.37251863	116.15448	7.10E-01	0.816091954	ns	86	3.12449485	127.32934	0.00221	0.02455556	*
87	0.34453698	118.44223	7.31E-01	0.829213483	ns	87	3.027163577	126.22541	0.00299	0.0299	*
88	0.29938118	119.60872	7.65E-01	0.85	ns	88	2.903107314	124.765	0.00437	0.03641667	*
89	0.25183552	118.98435	8.02E-01	0.865591398	ns	89	2.746712204	122.95249	0.00692	0.05323077	ns
90	0.24707391	115.7101	8.05E-01	0.865591398	ns	90	2.558782361	121.32628	0.0117	0.078	ns

91	0.23474681	113.07888	8.15E-01	0.867021277	ns	91	2.334511984	120.00198	0.0212	0.12470588	ns
92	0.16046799	112.54906	8.73E-01	0.909375	ns	92	2.131051052	118.66672	0.0351	0.18473684	ns
93	0.04169334	112.26713	9.67E-01	0.967	ns	93	1.947720667	117.21781	0.0538	0.269	ns
94	-0.10636744	110.72243	9.15E-01	0.933673469	ns	94	1.773330959	116.12534	0.0788	0.35818182	ns
95	-0.27423062	108.11069	7.84E-01	0.861538462	ns	95	1.618485141	115.5748	0.108	0.46956522	ns
96	-0.46130663	104.79035	6.46E-01	0.778313253	ns	96	1.493249831	115.6597	0.138	0.575	ns
97	-0.64915084	101.504	5.18E-01	0.672727273	ns	97	1.360609672	116.41148	0.176	0.67692308	ns
98	-0.79826193	99.34076	4.27E-01	0.584931507	ns	98	1.22106028	117.67559	0.225	0.83333333	ns
99	-0.8998534	98.69318	3.70E-01	0.536231884	ns	99	1.093844326	119.11882	0.276	0.871875	ns
100	-0.96627041	99.21766	3.36E-01	0.501492537	ns	100	0.968598946	120.18602	0.335	0.94722222	ns
9	statistic	df	p	p.adj	p.adj.signif	10	statistic	df	p	p.adj	p.adj.signif
1	-0.187644826	117.77833	0.851	0.9053191	ns	1	0.16574054	104.6138	0.869	0.8958763	ns
2	-0.217409697	122.28437	0.828	0.9	ns	2	0.23496004	106.5208	0.815	0.8747368	ns
3	-0.713386343	121.82026	0.477	0.7227273	ns	3	0.451887	111.4428	0.652	0.795122	ns
4	-1.019875777	118.22928	0.31	0.6245283	ns	4	0.7168397	117.3789	0.475	0.7307692	ns
5	-1.653969036	105.77976	0.101	0.3566667	ns	5	0.87393903	120.7618	0.384	0.6736842	ns
6	-2.009007695	96.97783	0.0473	0.2489474	ns	6	0.92873167	120.9876	0.355	0.6698113	ns
7	-2.211731796	92.3223	0.0295	0.2275	ns	7	1.13540017	118.0964	0.259	0.6166667	ns
8	-2.200223941	93.14382	0.0303	0.2275	ns	8	1.50432818	114.5319	0.135	0.5615385	ns
9	-2.200554652	90.90054	0.0303	0.2275	ns	9	1.88190991	112.9091	0.0624	0.5615385	ns
10	-1.769177252	95.99349	0.08	0.3354167	ns	10	2.10416541	111.2393	0.0376	0.5615385	ns
11	-1.559495895	101.20274	0.122	0.369697	ns	11	2.2100754	108.7272	0.0292	0.5615385	ns
12	-1.474780045	102.33491	0.143	0.3972222	ns	12	2.2899118	106.9787	0.024	0.5615385	ns
13	-1.163034728	104.63821	0.247	0.5369565	ns	13	2.27901769	106.0513	0.0247	0.5615385	ns
14	-0.665283591	109.15044	0.507	0.7347826	ns	14	2.1508928	105.7809	0.0338	0.5615385	ns

15	0.004465966	113.39315	0.996	0.998	ns	15	1.95234188	107.0317	0.0535	0.5615385	ns
16	0.436715652	120.04869	0.663	0.82875	ns	16	1.73668777	110.6401	0.0852	0.5615385	ns
17	0.943951975	125.77452	0.347	0.6309091	ns	17	1.5703054	114.5654	0.119	0.5615385	ns
18	1.335593283	127.76709	0.184	0.4487805	ns	18	1.47091519	116.7704	0.144	0.5615385	ns
19	1.505254155	127.99748	0.135	0.3970588	ns	19	1.39254004	116.9979	0.166	0.5615385	ns
20	1.367726763	127.92751	0.174	0.435	ns	20	1.25580105	115.9436	0.212	0.5615385	ns
21	0.75777601	126.84301	0.45	0.70625	ns	21	1.06101043	113.9352	0.291	0.6191489	ns
22	0.267376297	125.28593	0.79	0.8777778	ns	22	0.82397292	111.433	0.412	0.6983051	ns
23	-0.35625373	122.22601	0.722	0.8333333	ns	23	0.54457177	109.8793	0.587	0.7826667	ns
24	-0.758211425	119.49918	0.45	0.70625	ns	24	0.24710521	109.6914	0.805	0.8747368	ns
25	-1.185782379	116.18639	0.238	0.5369565	ns	25	-0.01346811	110.743	0.989	0.989	ns
26	-1.298706532	113.40636	0.197	0.4690476	ns	26	-0.22123115	112.7625	0.825	0.8747368	ns
27	-1.477822297	109.09326	0.142	0.3972222	ns	27	-0.33879662	116.3873	0.735	0.8448276	ns
28	-1.173732701	111.74973	0.243	0.5369565	ns	28	-0.43351589	120.348	0.665	0.8012048	ns
29	-0.928538714	115.54882	0.355	0.6339286	ns	29	-0.62437061	122.6948	0.534	0.7521127	ns
30	-1.087748402	115.5917	0.279	0.58125	ns	30	-0.87657001	122.8977	0.382	0.6736842	ns
31	-0.901873955	115.17971	0.369	0.6473684	ns	31	-1.03142173	121.6167	0.304	0.632	ns
32	-0.997951941	110.62323	0.32	0.6245283	ns	32	-1.06559134	119.9861	0.289	0.6191489	ns
33	-1.008641605	112.604	0.315	0.6245283	ns	33	-1.10042317	118.9704	0.273	0.6191489	ns
34	-1.446162997	108.46179	0.151	0.3973684	ns	34	-1.47837127	118.8272	0.142	0.5615385	ns
35	-1.562749236	106.63166	0.121	0.369697	ns	35	-1.91639935	118.2227	0.0577	0.5615385	ns
36	-1.950111939	111.59132	0.0537	0.2642857	ns	36	-1.98578916	118.3955	0.0494	0.5615385	ns
37	-2.07068071	113.13847	0.0407	0.2361111	ns	37	-1.73192538	118.6462	0.0859	0.5615385	ns
38	-2.239204357	113.97467	0.0271	0.2275	ns	38	-1.64555335	115.452	0.103	0.5615385	ns
39	-2.687892596	112.92352	0.00828	0.1656	ns	39	-1.61185018	110.8466	0.11	0.5615385	ns

40	-2.869207908	110.28988	0.00493	0.12325	ns	40	-1.41374645	108.9659	0.16	0.5615385	ns
41	-3.057498539	113.01994	0.00279	0.1066667	ns	41	-1.29451356	107.3307	0.198	0.5615385	ns
42	-3.098124779	114.78405	0.00245	0.1066667	ns	42	-1.18219325	106.0014	0.24	0.6	ns
43	-3.011312816	115.51375	0.0032	0.1066667	ns	43	-0.94779435	108.9966	0.345	0.6634615	ns
44	-2.587769763	119.03428	0.0109	0.1816667	ns	44	-0.7280605	110.5006	0.468	0.7307692	ns
45	-2.25860398	117.37775	0.0258	0.2275	ns	45	-0.47676696	112.114	0.634	0.79375	ns
46	-2.138619842	114.50476	0.0346	0.2275	ns	46	-0.06940281	116.4031	0.945	0.9545455	ns
47	-2.052765829	106.83052	0.0425	0.2361111	ns	47	0.39863193	119.3068	0.691	0.8186047	ns
48	-1.936237123	105.42365	0.0555	0.2642857	ns	48	0.89733864	121.7553	0.371	0.6736842	ns
49	-2.11813703	110.16261	0.0364	0.2275	ns	49	1.24703031	123.2091	0.215	0.5615385	ns
50	-2.322729966	115.48145	0.0219	0.2275	ns	50	1.25046333	121.5339	0.214	0.5615385	ns
51	-2.506819841	113.84999	0.0136	0.1942857	ns	51	1.06625496	116.3907	0.289	0.6191489	ns
52	-2.170701311	112.42339	0.0321	0.2275	ns	52	0.83246068	111.1025	0.407	0.6983051	ns
53	-1.763010832	115.61511	0.0805	0.3354167	ns	53	0.66877208	111.1872	0.505	0.7521127	ns
54	-1.720944392	116.43856	0.0879	0.3516	ns	54	0.45328192	111.2568	0.651	0.795122	ns
55	-1.453048113	111.025	0.149	0.3973684	ns	55	0.17080663	111.7028	0.865	0.8958763	ns
56	-1.654490415	106.71201	0.101	0.3566667	ns	56	-0.14284953	114.9482	0.887	0.905102	ns
57	-1.632360821	101.76188	0.106	0.3566667	ns	57	-0.38085055	119.3445	0.704	0.8186047	ns
58	-1.5696661	95.24458	0.12	0.369697	ns	58	-0.55036041	120.2296	0.583	0.7826667	ns
59	-1.410724176	96.6928	0.162	0.4153846	ns	59	-0.74593948	115.7105	0.457	0.7307692	ns
60	-1.238433184	99.97405	0.218	0.5069767	ns	60	-1.00817555	108.4515	0.316	0.632	ns
61	-0.987851365	103.98267	0.326	0.6245283	ns	61	-1.28425066	105.9724	0.202	0.5615385	ns
62	-0.819246376	103.42163	0.415	0.70625	ns	62	-1.3368932	106.4658	0.184	0.5615385	ns
63	-0.601016887	104.1577	0.549	0.7547945	ns	63	-1.29735593	107.4368	0.197	0.5615385	ns
64	-0.646347738	102.70086	0.519	0.7414286	ns	64	-1.23756094	106.7086	0.219	0.5615385	ns

65	-0.759457916	106.3666	0.449	0.70625	ns	65	-1.26649751	104.892	0.208	0.5615385	ns
66	-0.597478102	110.32155	0.551	0.7547945	ns	66	-1.39734467	103.7445	0.165	0.5615385	ns
67	-0.453713494	118.1443	0.651	0.82875	ns	67	-1.51130728	105.17	0.134	0.5615385	ns
68	-0.410233597	123.42708	0.682	0.8317073	ns	68	-1.45148294	108.7496	0.15	0.5615385	ns
69	-0.331542461	125.02909	0.741	0.8420455	ns	69	-1.40127955	113.485	0.164	0.5615385	ns
70	-0.558732328	123.30688	0.577	0.7693333	ns	70	-1.46823477	117.1607	0.145	0.5615385	ns
71	-0.734211316	121.72464	0.464	0.7138462	ns	71	-1.57347426	121.0844	0.118	0.5615385	ns
72	-0.754370423	117.03719	0.452	0.70625	ns	72	-1.60277994	123.862	0.112	0.5615385	ns
73	-0.975455623	110.70595	0.331	0.6245283	ns	73	-1.60750233	123.7618	0.11	0.5615385	ns
74	-0.673214252	111.40015	0.502	0.7347826	ns	74	-1.66331705	121.0318	0.0988	0.5615385	ns
75	-0.477054313	106.31348	0.634	0.82875	ns	75	-1.59203853	119.6081	0.114	0.5615385	ns
76	-0.246960893	100.23722	0.805	0.8846154	ns	76	-1.34507779	119.9457	0.181	0.5615385	ns
77	0.170502768	100.58704	0.865	0.9105263	ns	77	-1.15870994	118.8249	0.249	0.6073171	ns
78	0.421192452	102.29822	0.674	0.8317073	ns	78	-1.07086533	116.949	0.286	0.6191489	ns
79	0.597493843	109.13278	0.551	0.7547945	ns	79	-1.0094873	116.2333	0.315	0.632	ns
80	0.766343706	117.27187	0.445	0.70625	ns	80	-0.95646241	116.0785	0.341	0.6634615	ns
81	0.69980335	123.61902	0.485	0.7238806	ns	81	-0.88656805	115.9401	0.377	0.6736842	ns
82	0.441349602	127.06507	0.66	0.82875	ns	82	-0.78240929	115.9521	0.436	0.7266667	ns
83	0.353146846	127.95966	0.725	0.8333333	ns	83	-0.64332561	115.8726	0.521	0.7521127	ns
84	0.372605383	126.61728	0.71	0.8333333	ns	84	-0.50561188	114.9797	0.614	0.79375	ns
85	0.289473699	125.58023	0.773	0.8685393	ns	85	-0.39154949	113.4375	0.696	0.8186047	ns
86	0.108011649	125.93591	0.914	0.942268	ns	86	-0.30024311	111.2362	0.765	0.8606742	ns
87	0.002254764	126.31544	0.998	0.998	ns	87	-0.24435738	108.868	0.807	0.8747368	ns
88	-0.14110255	126.36808	0.888	0.925	ns	88	-0.21441519	107.1494	0.831	0.8747368	ns
89	-0.559329992	127.80135	0.577	0.7693333	ns	89	-0.21907471	106.7247	0.827	0.8747368	ns

90	-1.105933215	127.99973	0.271	0.5765957	ns	90	-0.29793752	108.0399	0.766	0.8606742	ns
91	-1.645799587	127.74073	0.102	0.3566667	ns	91	-0.47745299	110.2351	0.634	0.79375	ns
92	-1.840100585	125.36988	0.0681	0.3095455	ns	92	-0.65763679	113.1336	0.512	0.7521127	ns
93	-1.624608543	123.86451	0.107	0.3566667	ns	93	-0.72852501	117.0842	0.468	0.7307692	ns
94	-0.955609917	126.1845	0.341	0.6309091	ns	94	-0.71586755	120.6507	0.475	0.7307692	ns
95	-0.436183634	127.98354	0.663	0.82875	ns	95	-0.66204284	123.1439	0.509	0.7521127	ns
96	-0.189751513	127.29559	0.85	0.9053191	ns	96	-0.56943832	124.6728	0.57	0.7826667	ns
97	0.087841655	127.24587	0.93	0.9489796	ns	97	-0.47523835	125.654	0.635	0.79375	ns
98	0.36026419	126.39023	0.719	0.8333333	ns	98	-0.50118804	126.4083	0.617	0.79375	ns
99	0.378860098	120.00341	0.705	0.8333333	ns	99	-0.55953171	127.2457	0.577	0.7826667	ns
100	-0.773832425	108.43809	0.441	0.70625	ns	100	-0.62533976	127.6576	0.533	0.7521127	ns
11	statistic	df	p	p.adj	p.adj.signif	12	statistic	df	p	p.adj	p.adj.signif
1	1.756327403	113.0946	0.0817	0.30206897	ns	1	-0.051941479	127.46739	0.959	0.9886598	ns
2	1.637016019	117.2818	0.104	0.325	ns	2	0.242163542	126.88898	0.809	0.9043956	ns
3	1.59692118	120.1126	0.113	0.34242424	ns	3	0.415549907	127.66057	0.678	0.86375	ns
4	1.661598487	122.4304	0.0992	0.32	ns	4	0.430069116	127.95875	0.668	0.86375	ns
5	1.788255886	123.7703	0.0762	0.29307692	ns	5	0.399152933	127.99912	0.69	0.86375	ns
6	1.792448552	124.5948	0.0755	0.29307692	ns	6	0.335272703	127.95555	0.738	0.8770115	ns
7	1.527129068	124.438	0.129	0.36857143	ns	7	0.400297698	127.99564	0.69	0.86375	ns
8	1.118017368	125.2418	0.266	0.57826087	ns	8	0.601879503	127.80482	0.548	0.8058824	ns
9	0.705450956	126.6642	0.482	0.75384615	ns	9	0.540488896	127.90174	0.59	0.8428571	ns
10	0.431037769	127.9821	0.667	0.91234568	ns	10	0.273428028	127.94087	0.785	0.8920455	ns
11	0.191399047	127.1675	0.849	0.94333333	ns	11	-0.148019801	127.54861	0.883	0.9494624	ns
12	-0.271166413	123.9882	0.787	0.93370787	ns	12	-0.420775689	127.39667	0.675	0.86375	ns
13	-0.790837898	119.8708	0.431	0.75384615	ns	13	-0.437696699	125.63618	0.662	0.86375	ns

14	-1.021893686	117.3871	0.309	0.64375	ns	14	-0.345337518	119.604	0.73	0.8770115	ns
15	-0.852526209	116.4277	0.396	0.70714286	ns	15	-0.480297747	110.71188	0.632	0.86375	ns
16	-0.740787735	117.6711	0.46	0.75384615	ns	16	-0.731375667	107.32394	0.466	0.728125	ns
17	-0.942445285	119.5082	0.348	0.67307692	ns	17	-0.848316214	106.91781	0.398	0.6419355	ns
18	-1.372598685	121.1304	0.172	0.4195122	ns	18	-0.930497459	106.26313	0.354	0.59	ns
19	-1.912144529	122.8318	0.0582	0.2425	ns	19	-0.986398161	104.84996	0.326	0.59	ns
20	-2.36220853	124.7774	0.0197	0.13133333	ns	20	-1.058260455	103.77965	0.292	0.584	ns
21	-2.487298079	124.8368	0.0142	0.10923077	ns	21	-1.199643998	104.47558	0.233	0.5086957	ns
22	-2.303432244	122.4958	0.0229	0.13470588	ns	22	-1.353854234	106.18609	0.179	0.4589744	ns
23	-1.985824196	118.6958	0.0494	0.22130435	ns	23	-1.196543607	107.65854	0.234	0.5086957	ns
24	-1.7226571	115.3769	0.0876	0.30206897	ns	24	-0.820455781	106.79889	0.414	0.6571429	ns
25	-1.51377003	114.8943	0.133	0.36944444	ns	25	-0.413248051	107.01747	0.68	0.86375	ns
26	-1.470769264	114.9108	0.144	0.37435897	ns	26	-0.013678811	108.51558	0.989	0.993	ns
27	-1.559207834	116.9171	0.122	0.35882353	ns	27	0.311386102	111.43086	0.756	0.8770115	ns
28	-1.738151437	118.235	0.0848	0.30206897	ns	28	0.699909422	113.55981	0.485	0.7409091	ns
29	-1.972015228	120.9055	0.0509	0.22130435	ns	29	1.044163956	116.05501	0.299	0.5862745	ns
30	-2.316409194	122.0895	0.0222	0.13470588	ns	30	1.131362657	116.84397	0.26	0.5416667	ns
31	-2.552847866	120.1113	0.0119	0.1025	ns	31	1.028850216	115.93538	0.306	0.5884615	ns
32	-2.849756005	118.285	0.00516	0.069875	ns	32	0.93748445	115.57141	0.35	0.59	ns
33	-3.163075133	118.7611	0.00198	0.0495	*	33	0.972528139	114.57974	0.333	0.59	ns
34	-3.198212582	117.0303	0.00178	0.0495	*	34	1.103584153	114.9424	0.272	0.555102	ns
35	-3.037021501	114.7683	0.00296	0.05283333	ns	35	1.293989734	115.97062	0.198	0.4785714	ns
36	-2.823573834	115.5946	0.00559	0.069875	ns	36	1.61740044	114.83771	0.109	0.3257143	ns
37	-2.551243359	118.3952	0.012	0.1025	ns	37	1.975427759	112.62156	0.0507	0.24	ns
38	-2.368826394	121.4276	0.0194	0.13133333	ns	38	2.203149696	107.37689	0.0297	0.185625	ns

39	-2.227401474	122.1997	0.0278	0.153	ns	39	2.216330215	100.2449	0.0289	0.185625	ns
40	-2.122051206	120.758	0.0359	0.17095238	ns	40	2.035489086	94.85445	0.0446	0.223	ns
41	-2.189044136	119.3895	0.0305	0.153	ns	41	2.139746197	95.36065	0.0349	0.2021053	ns
42	-2.541929884	119.3395	0.0123	0.1025	ns	42	2.56367352	98.40191	0.0119	0.14875	ns
43	-3.012359343	119.0144	0.00317	0.05283333	ns	43	2.601819109	98.97022	0.0107	0.14875	ns
44	-3.423625671	116.1583	0.000854	0.0495	*	44	2.430526934	100.63722	0.0168	0.1663636	ns
45	-3.215796787	111.9839	0.0017	0.0495	*	45	2.398122188	104.38216	0.0183	0.1663636	ns
46	-2.629336368	109.0747	0.00979	0.1025	ns	46	2.445321778	111.08911	0.016	0.1663636	ns
47	-2.190639097	110.5811	0.0306	0.153	ns	47	2.644491534	118.36994	0.00929	0.14875	ns
48	-1.691902295	112.682	0.0934	0.31133333	ns	48	2.781159635	117.22458	0.00631	0.14875	ns
49	-1.150010759	111.921	0.253	0.575	ns	49	2.956372844	113.3483	0.00379	0.1263333	ns
50	-0.693332038	110.7875	0.49	0.75384615	ns	50	3.160824476	108.17223	0.00204	0.1263333	ns
51	-0.223406564	107.163	0.824	0.93370787	ns	51	2.994844112	100.41867	0.00346	0.1263333	ns
52	0.005522586	105.4564	0.996	0.996	ns	52	2.698075984	91.5573	0.0083	0.14875	ns
53	0.014101495	105.0652	0.989	0.996	ns	53	2.124698226	80.93664	0.0367	0.2021053	ns
54	-0.024949018	106.2848	0.98	0.996	ns	54	1.794104371	74.29659	0.0769	0.2848148	ns
55	-0.040053786	110.8475	0.968	0.996	ns	55	1.948671301	73.47155	0.0552	0.24	ns
56	0.063186641	114.718	0.95	0.996	ns	56	2.217306276	75.86476	0.0296	0.185625	ns
57	0.093007774	117.8388	0.926	0.98510638	ns	57	2.317316229	78.68084	0.0231	0.185625	ns
58	0.283295058	121.9001	0.777	0.93370787	ns	58	2.279811907	78.51221	0.0253	0.185625	ns
59	0.587066654	124.0083	0.558	0.83283582	ns	59	2.105684257	78.73097	0.0384	0.2021053	ns
60	0.8921024	124.609	0.374	0.68727273	ns	60	1.945948368	81.04734	0.0551	0.24	ns
61	0.974060598	124.8574	0.332	0.668	ns	61	1.850516945	85.94235	0.0677	0.2688462	ns
62	0.712219949	123.4826	0.478	0.75384615	ns	62	1.721939997	90.89482	0.0885	0.295	ns
63	0.214077781	123.5732	0.831	0.93370787	ns	63	1.544796101	93.46452	0.126	0.35	ns

64	-0.357628527	126.0344	0.721	0.91234568	ns	64	1.13930147	91.76078	0.258	0.5416667	ns
65	-0.758485865	126.7619	0.45	0.75384615	ns	65	0.95986041	91.18733	0.34	0.59	ns
66	-0.693317715	125.2314	0.489	0.75384615	ns	66	1.217710069	95.14436	0.226	0.5086957	ns
67	-0.493630806	122.3721	0.622	0.88857143	ns	67	1.596116924	101.70184	0.114	0.3257143	ns
68	-0.380776692	115.502	0.704	0.91234568	ns	68	1.831196856	107.08632	0.0699	0.2688462	ns
69	-0.375346151	109.7146	0.708	0.91234568	ns	69	1.744008203	108.22569	0.084	0.295	ns
70	-0.609223061	107.9832	0.544	0.82424242	ns	70	1.723095529	103.74669	0.0879	0.295	ns
71	-0.697643119	111.5961	0.487	0.75384615	ns	71	1.626660356	94.23424	0.107	0.3257143	ns
72	-0.398384039	114.1362	0.691	0.91234568	ns	72	1.498990558	89.59646	0.137	0.3702703	ns
73	0.242690006	116.6454	0.809	0.93370787	ns	73	1.405492787	88.55883	0.163	0.4289474	ns
74	0.716712015	119.7963	0.475	0.75384615	ns	74	1.326527341	87.13967	0.188	0.47	ns
75	0.373148766	112.965	0.71	0.91234568	ns	75	0.991526465	82.26543	0.324	0.59	ns
76	0.22711077	107.3996	0.821	0.93370787	ns	76	0.965153401	74.19142	0.338	0.59	ns
77	0.14785125	104.9797	0.883	0.95978261	ns	77	0.952473412	68.98268	0.344	0.59	ns
78	0.16899515	109.9392	0.866	0.95164835	ns	78	0.696425186	65.80653	0.489	0.7409091	ns
79	0.345771996	117.9609	0.73	0.91234568	ns	79	0.427473932	64.49511	0.67	0.86375	ns
80	0.236985958	124.3426	0.813	0.93370787	ns	80	0.314323289	65.36507	0.754	0.8770115	ns
81	0.334552356	125.6625	0.739	0.91234568	ns	81	0.369008454	68.61751	0.713	0.8770115	ns
82	0.969014802	124.8458	0.334	0.668	ns	82	0.399143332	69.76739	0.691	0.86375	ns
83	1.300261911	125.8932	0.196	0.46666667	ns	83	0.339855415	74.00394	0.735	0.8770115	ns
84	1.243145551	125.7928	0.216	0.50232558	ns	84	0.303055664	82.30147	0.763	0.8770115	ns
85	0.937862796	123.5586	0.35	0.67307692	ns	85	0.224842996	94.13756	0.823	0.9043956	ns
86	0.428689833	122.4373	0.669	0.91234568	ns	86	-0.008247569	102.70329	0.993	0.993	ns
87	-0.025462141	121.13	0.98	0.996	ns	87	0.094613295	110.20766	0.925	0.9635417	ns
88	-0.462158377	118.6833	0.645	0.9084507	ns	88	0.153007617	110.91739	0.879	0.9494624	ns

89	-0.903540576	117.1522	0.368	0.68727273	ns	89	0.124703962	106.22913	0.901	0.9536842	ns
90	-1.126327702	118.618	0.262	0.57826087	ns	90	-0.118736107	101.70847	0.906	0.9536842	ns
91	-0.884299328	122.46	0.378	0.68727273	ns	91	-0.641328148	98.18969	0.523	0.780597	ns
92	-0.544790198	123.1931	0.587	0.85072464	ns	92	-1.286721001	97.03428	0.201	0.4785714	ns
93	-0.365667837	119.9471	0.715	0.91234568	ns	93	-1.601095597	94.3868	0.113	0.3257143	ns
94	-0.132002638	119.3778	0.895	0.96236559	ns	94	-1.83526988	89.59543	0.0698	0.2688462	ns
95	0.243892952	120.2896	0.808	0.93370787	ns	95	-1.679033507	89.92295	0.0966	0.3116129	ns
96	0.572989508	123.3217	0.568	0.83529412	ns	96	-1.238402254	95.00702	0.219	0.5086957	ns
97	1.097499411	125.762	0.275	0.58510638	ns	97	-0.905035466	101.27814	0.368	0.6032787	ns
98	1.475169601	126.3111	0.143	0.37435897	ns	98	-0.572400064	106.8019	0.568	0.8231884	ns
99	1.462823231	123.7312	0.146	0.37435897	ns	99	-0.233069862	108.59531	0.816	0.9043956	ns
100	1.417187299	119.2055	0.159	0.3975	ns	100	0.030726446	107.89955	0.976	0.993	ns
13	statistic	df	p	p.adj	p.adj.signif	14	statistic	df	p	p.adj	p.adj.signif
1	-2.24614416	87.23804	0.0272	0.055510204	ns	1	-1.41104968	105.76749	0.161	0.20641026	ns
2	-2.40141145	89.24282	0.0184	0.039574468	*	2	-1.47888418	106.35146	0.142	0.18933333	ns
3	-2.63970992	92.34095	0.00974	0.027055556	*	3	-1.52036633	107.20329	0.131	0.17702703	ns
4	-2.8708506	94.36012	0.00505	0.016833333	*	4	-1.54539526	107.42265	0.125	0.17605634	ns
5	-3.09535028	95.8493	0.00258	0.009923077	**	5	-1.5750115	107.44029	0.118	0.16857143	ns
6	-3.28462249	97.17363	0.00142	0.006454545	**	6	-1.6445994	107.37453	0.103	0.15373134	ns
7	-3.42632144	98.04466	0.000895	0.004475	**	7	-1.70343207	107.08737	0.0914	0.15142857	ns
8	-3.51349113	98.96145	0.000668	0.004082353	**	8	-1.68907657	105.95356	0.0941	0.15142857	ns
9	-3.52974715	100.48046	0.000629	0.004082353	**	9	-1.66326259	104.24941	0.0993	0.15276923	ns
10	-3.50878598	103.05128	0.000669	0.004082353	**	10	-1.6329057	101.72135	0.106	0.15588235	ns
11	-3.49376907	106.81052	0.000694	0.004082353	**	11	-1.59687802	99.01162	0.113	0.16376812	ns
12	-3.52892788	110.55638	0.000609	0.004082353	**	12	-1.53873907	98.13092	0.127	0.17638889	ns

13	-3.58085467	113.9377	0.000505	0.004082353	**	13	-1.46260788	100.38481	0.147	0.19342105	ns
14	-3.60058443	116.98925	0.000467	0.004082353	**	14	-1.36723943	104.70078	0.174	0.21463415	ns
15	-3.5524567	118.61369	0.000549	0.004082353	**	15	-1.24129317	108.87473	0.217	0.25529412	ns
16	-3.44222489	118.8919	0.000797	0.004427778	**	16	-1.07626719	112.35141	0.284	0.32643678	ns
17	-3.31797654	118.94195	0.0012	0.005714286	**	17	-0.9107347	114.62137	0.364	0.40898876	ns
18	-3.16994294	118.43892	0.00194	0.00776	**	18	-0.75183935	115.92445	0.454	0.5032967	ns
19	-3.00263087	117.53542	0.00327	0.011678571	*	19	-0.58055512	116.8868	0.563	0.61195652	ns
20	-2.83958325	116.75606	0.00533	0.017193548	*	20	-0.4200672	117.53422	0.675	0.71808511	ns
21	-2.74591944	117.20964	0.00699	0.020970588	*	21	-0.29449024	117.29742	0.769	0.80104167	ns
22	-2.72108534	118.04239	0.00749	0.0214	*	22	-0.1795364	116.91146	0.858	0.8755102	ns
23	-2.82222541	118.81216	0.00559	0.01746875	*	23	-0.05942906	116.9614	0.953	0.953	ns
24	-3.0161586	119.77865	0.00313	0.011592593	*	24	0.07175173	117.82905	0.943	0.95252525	ns
25	-3.20724166	120.4619	0.00172	0.007166667	**	25	0.23941305	119.47702	0.811	0.83608247	ns
26	-3.41475531	121.53898	0.000868	0.004475	**	26	0.39635626	120.49895	0.693	0.72947368	ns
27	-3.64803063	123.0429	0.000389	0.004082353	**	27	0.54267882	120.41355	0.588	0.63225806	ns
28	-3.80838742	123.6684	0.000219	0.004082353	**	28	0.74385822	120.13613	0.458	0.5032967	ns
29	-3.89837618	123.66019	0.000158	0.004082353	**	29	0.98719333	120.94559	0.326	0.37045455	ns
30	-3.88386846	123.44765	0.000167	0.004082353	**	30	1.21025915	122.23573	0.229	0.26627907	ns
31	-3.85715816	123.41126	0.000184	0.004082353	**	31	1.36088421	122.52395	0.176	0.21463415	ns
32	-3.81209955	124.18984	0.000216	0.004082353	**	32	1.40982132	121.86435	0.161	0.20641026	ns
33	-3.7601685	125.35889	0.000259	0.004082353	**	33	1.36331143	121.44403	0.175	0.21463415	ns
34	-3.68326459	126.0281	0.00034	0.004082353	**	34	1.32533438	121.68942	0.188	0.225	ns
35	-3.50943666	126.66439	0.000622	0.004082353	**	35	1.32130389	121.93415	0.189	0.225	ns
36	-3.23713409	127.31888	0.00154	0.006695652	**	36	1.39288892	121.81619	0.166	0.21012658	ns
37	-2.97245223	127.76491	0.00353	0.012172414	*	37	1.52577371	121.76904	0.13	0.17702703	ns

38	-2.73474115	127.99534	0.00713	0.020970588	*		38	1.69676469	121.68381	0.0923	0.15142857	ns
39	-2.60384542	127.91074	0.0103	0.027837838	*		39	1.9102948	121.4428	0.0585	0.14404762	ns
40	-2.55801726	127.62706	0.0117	0.0295	*		40	2.14344995	121.5966	0.0341	0.1065625	ns
41	-2.55349169	127.14856	0.0118	0.0295	*		41	2.42643639	122.41746	0.0167	0.0726087	ns
42	-2.57352073	126.95639	0.0112	0.029473684	*		42	2.692261	123.1786	0.00808	0.047529	*
43	-2.53843165	126.97297	0.0123	0.03	*		43	2.914536	123.5196	0.00423	0.03975	*
44	-2.50205966	127.2132	0.0136	0.031627907	*		44	3.082985	122.8648	0.00253	0.03975	*
45	-2.39408695	127.38444	0.0181	0.039574468	*		45	3.073605	122.6215	0.00261	0.03975	*
46	-2.16020579	127.70197	0.0326	0.063921569	ns		46	2.949661	122.2327	0.00381	0.03975	*
47	-1.85421997	127.87061	0.066	0.117857143	ns		47	2.804752	121.4293	0.00587	0.03975	*
48	-1.48948447	124.84732	0.139	0.227868852	ns		48	2.58599541	120.86748	0.0109	0.0545	ns
49	-1.18202497	119.01213	0.24	0.347826087	ns		49	2.30757663	120.69143	0.0227	0.0908	ns
50	-1.07655435	118.03108	0.284	0.383783784	ns		50	2.04491758	118.81684	0.0431	0.11756757	ns
51	-1.01434185	119.38945	0.312	0.4	ns		51	1.83005291	116.10599	0.0698	0.15	ns
52	-0.97729473	121.35838	0.33	0.417721519	ns		52	1.71260561	114.17806	0.0895	0.15142857	ns
53	-1.01892255	123.48187	0.31	0.4	ns		53	1.6506736	112.07351	0.102	0.15373134	ns
54	-1.13286573	125.00877	0.259	0.359722222	ns		54	1.67209254	110.22353	0.0973	0.15203125	ns
55	-1.24677617	125.49547	0.215	0.316176471	ns		55	1.79054826	109.65001	0.0761	0.15142857	ns
56	-1.37219936	125.25787	0.172	0.273015873	ns		56	1.98689854	110.84127	0.0494	0.13	ns
57	-1.54982077	124.72045	0.124	0.206666667	ns		57	2.19631491	112.54801	0.0301	0.1037931	ns
58	-1.75333968	124.39187	0.082	0.14137931	ns		58	2.38524663	112.01865	0.0187	0.07791667	ns
59	-1.93181939	124.13287	0.0557	0.101272727	ns		59	2.53012514	108.52529	0.0128	0.06095238	ns
60	-2.12549068	123.21596	0.0355	0.068269231	ns		60	2.65116	103.1597	0.00929	0.048895	*
61	-2.31473746	121.62826	0.0223	0.046458333	*		61	2.78795	98.88241	0.00636	0.03975	*
62	-2.45452277	120.67816	0.0155	0.035227273	*		62	2.882801	95.75683	0.00487	0.03975	*

63	-2.50354826	120.42299	0.0136	0.031627907	*	63	2.913446	94.03363	0.00447	0.03975	*
64	-2.38649631	119.96996	0.0186	0.039574468	*	64	2.865547	93.83081	0.00514	0.03975	*
65	-2.20772404	118.36664	0.0292	0.0584	ns	65	2.860045	94.4021	0.00521	0.03975	*
66	-2.05278937	115.91762	0.0423	0.079811321	ns	66	2.942504	95.51158	0.00409	0.03975	*
67	-1.93392643	113.52249	0.0556	0.101272727	ns	67	3.003694	96.50579	0.0034	0.03975	*
68	-1.80172609	111.78527	0.0743	0.130350877	ns	68	3.039915	96.96907	0.00304	0.03975	*
69	-1.60339244	110.43531	0.112	0.189830508	ns	69	3.027124	97.37181	0.00316	0.03975	*
70	-1.39915031	109.71608	0.165	0.266129032	ns	70	2.923737	98.34629	0.00429	0.03975	*
71	-1.30773392	109.48903	0.194	0.293939394	ns	71	2.798607	99.86441	0.00616	0.03975	*
72	-1.32451589	110.39269	0.188	0.289230769	ns	72	2.660195	101.1902	0.00908	0.048895	*
73	-1.33891543	112.67217	0.183	0.2859375	ns	73	2.47080509	101.79826	0.0151	0.06863636	ns
74	-1.28247832	115.49905	0.202	0.301492537	ns	74	2.26012928	102.91694	0.0259	0.09961538	ns
75	-1.12489899	117.56458	0.263	0.360273973	ns	75	2.05162726	104.52559	0.0427	0.11756757	ns
76	-0.95465177	118.2388	0.342	0.422222222	ns	76	1.86233638	106.47192	0.0653	0.14840909	ns
77	-0.87610397	119.51964	0.383	0.461445783	ns	77	1.72010717	108.30756	0.0883	0.15142857	ns
78	-0.80503619	120.28065	0.422	0.496470588	ns	78	1.68939387	109.29037	0.094	0.15142857	ns
79	-0.69040892	120.23017	0.491	0.551685393	ns	79	1.75746749	109.61321	0.0816	0.15142857	ns
80	-0.54710658	119.9081	0.585	0.623404255	ns	80	1.90217436	109.10676	0.0598	0.14404762	ns
81	-0.33202163	119.20987	0.74	0.762886598	ns	81	2.0421764	108.98721	0.0435	0.11756757	ns
82	-0.11521091	118.77609	0.908	0.917171717	ns	82	2.11268063	109.447	0.0369	0.11181818	ns
83	0.05624743	119.52062	0.955	0.955	ns	83	2.14640457	110.31812	0.034	0.1065625	ns
84	0.264858	120.54244	0.792	0.808163265	ns	84	2.18003385	110.93533	0.0314	0.10466667	ns
85	0.50782779	121.24869	0.612	0.6375	ns	85	2.20810393	110.09054	0.0293	0.1037931	ns
86	0.75097194	121.8292	0.454	0.527906977	ns	86	2.20075594	108.50701	0.0299	0.1037931	ns
87	0.92286058	121.833	0.358	0.436585366	ns	87	2.09741761	106.08851	0.0383	0.11264706	ns

88	1.05929756	121.53455	0.292	0.389333333	ns	88	1.9636077	103.53849	0.0523	0.13410256	ns
89	1.14763663	121.75495	0.253	0.359722222	ns	89	1.82913956	101.98204	0.0703	0.15	ns
90	1.1340074	122.56581	0.259	0.359722222	ns	90	1.73686813	101.56392	0.0854	0.15142857	ns
91	1.01445523	123.38235	0.312	0.4	ns	91	1.74582297	101.8352	0.0839	0.15142857	ns
92	0.85764013	123.85869	0.393	0.467857143	ns	92	1.82784122	102.44379	0.0705	0.15	ns
93	0.74050467	123.5721	0.46	0.528735632	ns	93	1.89764699	103.18762	0.0605	0.14404762	ns
94	0.63717959	122.30218	0.525	0.583333333	ns	94	1.87871944	103.947	0.0631	0.14674419	ns
95	0.57102002	120.37908	0.569	0.618478261	ns	95	1.81544833	104.19195	0.0723	0.150625	ns
96	0.5181059	118.35827	0.605	0.636842105	ns	96	1.77524608	103.62591	0.0788	0.15142857	ns
97	0.54567386	116.04256	0.586	0.623404255	ns	97	1.78446866	102.85641	0.0773	0.15142857	ns
98	0.60095717	113.45154	0.549	0.603296703	ns	98	1.77303812	101.7771	0.0792	0.15142857	ns
99	0.70626442	109.77747	0.482	0.547727273	ns	99	1.74750456	101.33995	0.0836	0.15142857	ns
100	0.95558809	102.56736	0.342	0.422222222	ns	100	1.68317066	100.73075	0.0954	0.15142857	ns
15	statistic	df	p	p.adj	p.adj.signif	16	statistic	df	p	p.adj	p.adj.signif
1	-1.81722346	127.2994	0.0715	0.7588235	ns	1	1.750188675	87.87042	0.0836	0.26125	ns
2	-1.75772423	127.3495	0.0812	0.7588235	ns	2	1.782237462	87.17515	0.0782	0.2522581	ns
3	-1.71145757	127.5088	0.0894	0.7588235	ns	3	1.812324389	86.55885	0.0734	0.2446667	ns
4	-1.66803489	127.7304	0.0978	0.7588235	ns	4	1.832660455	86.04633	0.0703	0.2424138	ns
5	-1.60988389	127.8952	0.11	0.7588235	ns	5	1.846077421	85.62912	0.0683	0.2424138	ns
6	-1.5460606	127.9834	0.125	0.7588235	ns	6	1.854880644	85.36287	0.0671	0.2424138	ns
7	-1.4831955	127.9962	0.14	0.7588235	ns	7	1.860794327	85.24615	0.0662	0.2424138	ns
8	-1.42942591	127.946	0.155	0.7588235	ns	8	1.866863614	85.2331	0.0654	0.2424138	ns
9	-1.38159091	127.8564	0.17	0.7588235	ns	9	1.882291338	85.25628	0.0632	0.2424138	ns
10	-1.33689604	127.7592	0.184	0.7588235	ns	10	1.908123761	85.33916	0.0597	0.2424138	ns
11	-1.30370978	127.6958	0.195	0.7588235	ns	11	1.939124854	85.50265	0.0558	0.2424138	ns

12	-1.28523243	127.6968	0.201	0.7588235	ns	12	1.973881096	85.77268	0.0516	0.2424138	ns
13	-1.27451294	127.743	0.205	0.7588235	ns	13	2.011230298	86.11444	0.0474	0.2424138	ns
14	-1.25823554	127.8043	0.211	0.7588235	ns	14	2.053831694	86.58876	0.043	0.2424138	ns
15	-1.23704045	127.8722	0.218	0.7588235	ns	15	2.101145967	87.31907	0.0385	0.2424138	ns
16	-1.21941591	127.9407	0.225	0.7588235	ns	16	2.153520709	88.31064	0.034	0.2424138	ns
17	-1.20857481	127.9919	0.229	0.7588235	ns	17	2.209840391	89.58378	0.0297	0.2424138	ns
18	-1.19192687	127.9885	0.235	0.7588235	ns	18	2.265364015	91.03841	0.0259	0.2424138	ns
19	-1.17012291	127.8894	0.244	0.7588235	ns	19	2.311652083	92.53006	0.023	0.2424138	ns
20	-1.15037183	127.6532	0.252	0.7588235	ns	20	2.343756721	93.98652	0.0212	0.2424138	ns
21	-1.13349763	127.2063	0.259	0.7588235	ns	21	2.359608774	95.31372	0.0203	0.2424138	ns
22	-1.12119901	126.5389	0.264	0.7588235	ns	22	2.365964266	96.45484	0.02	0.2424138	ns
23	-1.11322781	125.7449	0.268	0.7588235	ns	23	2.369546698	97.46436	0.0198	0.2424138	ns
24	-1.11123362	124.9301	0.269	0.7588235	ns	24	2.36379417	98.32663	0.0201	0.2424138	ns
25	-1.1101074	124.1315	0.269	0.7588235	ns	25	2.340657351	98.98179	0.0213	0.2424138	ns
26	-1.1084521	123.4102	0.27	0.7588235	ns	26	2.297304574	99.42425	0.0237	0.2424138	ns
27	-1.10566904	122.8439	0.271	0.7588235	ns	27	2.235716705	99.68513	0.0276	0.2424138	ns
28	-1.1044752	122.5025	0.272	0.7588235	ns	28	2.16490405	99.81375	0.0328	0.2424138	ns
29	-1.10531381	122.3976	0.271	0.7588235	ns	29	2.089274021	99.83349	0.0392	0.2424138	ns
30	-1.10382144	122.4924	0.272	0.7588235	ns	30	2.012999104	99.7648	0.0468	0.2424138	ns
31	-1.09466113	122.734	0.276	0.7588235	ns	31	1.93256402	99.61717	0.0561	0.2424138	ns
32	-1.07905404	123.0542	0.283	0.7588235	ns	32	1.836508777	99.41782	0.0693	0.2424138	ns
33	-1.05631733	123.3511	0.293	0.7588235	ns	33	1.715115544	99.16415	0.0894	0.2709091	ns
34	-1.03122813	123.5679	0.304	0.7588235	ns	34	1.570657186	98.84431	0.119	0.35	ns
35	-1.00410552	123.7206	0.317	0.7588235	ns	35	1.40851009	98.49248	0.162	0.4512195	ns
36	-0.97277918	123.8679	0.333	0.7588235	ns	36	1.238780706	98.22826	0.218	0.4659574	ns

37	-0.9390051	124.0098	0.35	0.7588235	ns	37	1.071891765	98.2387	0.286	0.5296296	ns
38	-0.90281404	124.1651	0.368	0.7588235	ns	38	0.910677668	98.58718	0.365	0.6032258	ns
39	-0.86405281	124.3757	0.389	0.7588235	ns	39	0.757557778	99.31655	0.451	0.6219178	ns
40	-0.82002775	124.5772	0.414	0.7588235	ns	40	0.616654875	100.43261	0.539	0.7092105	ns
41	-0.77535443	124.7807	0.44	0.7588235	ns	41	0.488305105	101.88351	0.626	0.8025641	ns
42	-0.74097297	124.9379	0.46	0.7588235	ns	42	0.376661921	103.54908	0.707	0.88375	ns
43	-0.7204463	124.955	0.473	0.7588235	ns	43	0.284036658	105.30535	0.777	0.947561	ns
44	-0.70538179	124.8137	0.482	0.7588235	ns	44	0.209593049	107.09094	0.834	0.9928571	ns
45	-0.68988381	124.5835	0.492	0.7588235	ns	45	0.14748103	108.90145	0.883	0.997	ns
46	-0.6727114	124.3016	0.502	0.7588235	ns	46	0.09623643	110.72848	0.924	0.997	ns
47	-0.65532511	123.9813	0.513	0.7588235	ns	47	0.06053834	112.62743	0.952	0.997	ns
48	-0.63411271	123.623	0.527	0.7637681	ns	48	0.039672107	114.64944	0.968	0.997	ns
49	-0.60018897	123.1761	0.549	0.7732394	ns	49	0.029642295	116.78258	0.976	0.997	ns
50	-0.55172183	122.6123	0.582	0.7972603	ns	50	0.02459545	118.96682	0.98	0.997	ns
51	-0.49672593	121.9588	0.62	0.8157895	ns	51	0.019281138	121.10789	0.985	0.997	ns
52	-0.43898016	121.3175	0.661	0.8367089	ns	52	0.012584175	123.12034	0.99	0.997	ns
53	-0.38184744	120.7682	0.703	0.8765432	ns	53	0.003779065	124.85831	0.997	0.997	ns
54	-0.32354851	120.3268	0.747	0.9	ns	54	-0.004981325	126.22668	0.996	0.997	ns
55	-0.25674659	119.8436	0.798	0.927907	ns	55	-0.012969679	127.17904	0.99	0.997	ns
56	-0.18002351	119.4243	0.857	0.9674157	ns	56	-0.025177151	127.73578	0.98	0.997	ns
57	-0.09591432	119.2263	0.924	0.991	ns	57	-0.046485272	127.97448	0.963	0.997	ns
58	-0.01097503	119.3355	0.991	0.991	ns	58	-0.079170275	127.98075	0.937	0.997	ns
59	0.06887034	119.8089	0.945	0.991	ns	59	-0.123996147	127.86425	0.902	0.997	ns
60	0.14567363	120.4698	0.884	0.9822222	ns	60	-0.185276669	127.7264	0.853	0.997	ns
61	0.22025783	121.2449	0.826	0.9494253	ns	61	-0.261259697	127.63475	0.794	0.9566265	ns

62	0.29682791	122.0638	0.767	0.9130952	ns	62	-0.346478009	127.6217	0.73	0.9012346	ns
63	0.37324651	122.895	0.71	0.8765432	ns	63	-0.435883437	127.70178	0.664	0.8405063	ns
64	0.44801346	123.7368	0.655	0.8367089	ns	64	-0.527373767	127.84475	0.599	0.7779221	ns
65	0.52035913	124.5636	0.604	0.8053333	ns	65	-0.622123824	127.97169	0.535	0.7092105	ns
66	0.58859621	125.3324	0.557	0.7736111	ns	66	-0.717099494	127.98894	0.475	0.6418919	ns
67	0.65211334	126.0401	0.516	0.7588235	ns	67	-0.808332497	127.84127	0.42	0.6219178	ns
68	0.71040786	126.6814	0.479	0.7588235	ns	68	-0.89126854	127.51252	0.374	0.6032258	ns
69	0.76160686	127.232	0.448	0.7588235	ns	69	-0.960800687	127.01108	0.338	0.5779661	ns
70	0.80889277	127.6397	0.42	0.7588235	ns	70	-1.020514199	126.3475	0.309	0.5517857	ns
71	0.85289634	127.8778	0.395	0.7588235	ns	71	-1.074575237	125.54813	0.285	0.5296296	ns
72	0.89453166	127.9814	0.373	0.7588235	ns	72	-1.122327111	124.62606	0.264	0.5176471	ns
73	0.93520542	127.9989	0.351	0.7588235	ns	73	-1.162537793	123.57565	0.247	0.494	ns
74	0.9728779	127.9731	0.332	0.7588235	ns	74	-1.19895701	122.43325	0.233	0.4854167	ns
75	1.00277813	127.9315	0.318	0.7588235	ns	75	-1.23517608	121.24648	0.219	0.4659574	ns
76	1.0219434	127.8918	0.309	0.7588235	ns	76	-1.271337013	120.03151	0.206	0.4659574	ns
77	1.02855113	127.8711	0.306	0.7588235	ns	77	-1.304034787	118.78788	0.195	0.4581395	ns
78	1.02232916	127.8808	0.309	0.7588235	ns	78	-1.333845284	117.54198	0.185	0.4512195	ns
79	1.00920646	127.9141	0.315	0.7588235	ns	79	-1.358193981	116.30417	0.177	0.4512195	ns
80	0.98934895	127.9495	0.324	0.7588235	ns	80	-1.373516153	115.07994	0.172	0.4512195	ns
81	0.96099391	127.9776	0.338	0.7588235	ns	81	-1.377636727	113.90076	0.171	0.4512195	ns
82	0.92418248	127.996	0.357	0.7588235	ns	82	-1.36840378	112.79303	0.174	0.4512195	ns
83	0.88127663	127.9979	0.38	0.7588235	ns	83	-1.342399886	111.71485	0.182	0.4512195	ns
84	0.83359816	127.9691	0.406	0.7588235	ns	84	-1.297280099	110.73062	0.197	0.4581395	ns
85	0.78686645	127.9015	0.433	0.7588235	ns	85	-1.23914302	109.83518	0.218	0.4659574	ns
86	0.73966299	127.7853	0.461	0.7588235	ns	86	-1.173347484	109.17022	0.243	0.494	ns

87	0.68341101	127.5941	0.496	0.7588235	ns	87	-1.109406891	108.89148	0.27	0.5192308	ns
88	0.61200394	127.2976	0.542	0.7732394	ns	88	-1.054309922	109.10557	0.294	0.5345455	ns
89	0.52870371	126.9216	0.598	0.8053333	ns	89	-1.007101917	109.70594	0.316	0.554386	ns
90	0.44175259	126.5114	0.659	0.8367089	ns	90	-0.955499382	110.52315	0.341	0.5779661	ns
91	0.35385321	126.11	0.724	0.8829268	ns	91	-0.892661498	111.36309	0.374	0.6032258	ns
92	0.26464159	125.7402	0.792	0.927907	ns	92	-0.825366868	112.1865	0.411	0.6219178	ns
93	0.17488305	125.448	0.861	0.9674157	ns	93	-0.774494452	113.08016	0.44	0.6219178	ns
94	0.09198142	125.2483	0.927	0.991	ns	94	-0.752080469	114.01867	0.454	0.6219178	ns
95	0.02265902	125.1455	0.982	0.991	ns	95	-0.753858584	114.89367	0.452	0.6219178	ns
96	-0.02959407	125.1447	0.976	0.991	ns	96	-0.768614991	115.55573	0.444	0.6219178	ns
97	-0.06005496	125.2116	0.952	0.991	ns	97	-0.788290893	116.01352	0.432	0.6219178	ns
98	-0.06160005	125.3478	0.951	0.991	ns	98	-0.803499458	116.34125	0.423	0.6219178	ns
99	-0.03066984	125.5502	0.976	0.991	ns	99	-0.812404222	116.63279	0.418	0.6219178	ns
100	0.0228932	125.808	0.982	0.991	ns	100	-0.817664103	116.89155	0.415	0.6219178	ns
17	statistic	df	p	p.adj	p.adj.signif	18	statistic	df	p	p.adj	p.adj.signif
1	1.20249495	79.89014	0.233	0.5065217	ns	1	2.12591287	110.91349	0.0357	0.1785	ns
2	1.14003413	83.96948	0.258	0.5326531	ns	2	2.02605171	111.42159	0.0451	0.1883333	ns
3	1.06691811	90.04925	0.289	0.5666667	ns	3	1.93179858	111.67199	0.0559	0.1996429	ns
4	0.97340606	97.89563	0.333	0.6259259	ns	4	1.85512845	111.63956	0.0662	0.2	ns
5	0.85147978	105.79091	0.396	0.665	ns	5	1.78119106	111.2215	0.0776	0.2	ns
6	0.72158211	110.21087	0.472	0.6913043	ns	6	1.71888354	110.55076	0.0884	0.2	ns
7	0.60105865	110.31267	0.549	0.7418919	ns	7	1.68340891	109.58442	0.0951	0.2	ns
8	0.53163587	107.92901	0.596	0.7440476	ns	8	1.6696229	108.13874	0.0979	0.2	ns
9	0.49850187	106.0018	0.619	0.7440476	ns	9	1.66911753	106.4023	0.098	0.2	ns
10	0.49033531	105.69304	0.625	0.7440476	ns	10	1.67073286	104.61188	0.0978	0.2	ns

11	0.51149467	106.67883	0.61	0.7440476	ns	11	1.67356367	102.8799	0.0973	0.2	ns
12	0.57753295	108.51665	0.565	0.7440476	ns	12	1.67334424	101.24316	0.0973	0.2	ns
13	0.67274757	110.21416	0.503	0.7185714	ns	13	1.6615169	99.75802	0.0997	0.2	ns
14	0.75651131	111.08059	0.451	0.6833333	ns	14	1.63434789	98.42571	0.105	0.2058824	ns
15	0.8270163	111.19253	0.41	0.6721311	ns	15	1.59435062	97.24219	0.114	0.2150943	ns
16	0.87058076	110.69361	0.386	0.665	ns	16	1.54489443	96.32129	0.126	0.2290909	ns
17	0.87281928	109.86423	0.385	0.665	ns	17	1.49152299	95.77442	0.139	0.2431034	ns
18	0.84597004	109.18746	0.399	0.665	ns	18	1.44008964	95.6264	0.153	0.259322	ns
19	0.7934365	108.7525	0.429	0.6809524	ns	19	1.39641015	95.87406	0.166	0.2677419	ns
20	0.71383677	108.37807	0.477	0.6913043	ns	20	1.35818979	96.47448	0.178	0.269697	ns
21	0.6122486	107.87677	0.542	0.7418919	ns	21	1.32226232	97.35259	0.189	0.2742857	ns
22	0.50661338	107.33542	0.613	0.7440476	ns	22	1.29525644	98.401	0.198	0.2743243	ns
23	0.39724519	106.8136	0.692	0.7604396	ns	23	1.28102747	99.41946	0.203	0.2743243	ns
24	0.28758178	106.43577	0.774	0.8322581	ns	24	1.28096284	100.20918	0.203	0.2743243	ns
25	0.17721426	106.0917	0.86	0.9052632	ns	25	1.29479856	100.65699	0.198	0.2743243	ns
26	0.08022013	105.99409	0.936	0.955102	ns	26	1.31809865	100.73139	0.19	0.2742857	ns
27	0.00648317	105.9403	0.995	0.995	ns	27	1.33878039	100.47197	0.184	0.2742857	ns
28	-0.04989359	105.62386	0.96	0.969697	ns	28	1.35650928	100.01455	0.178	0.269697	ns
29	-0.10035448	105.32725	0.92	0.9484536	ns	29	1.37030676	99.45914	0.174	0.269697	ns
30	-0.16469015	105.13172	0.87	0.90625	ns	30	1.38181191	98.76402	0.17	0.269697	ns
31	-0.24765981	105.06563	0.805	0.856383	ns	31	1.39856644	97.91541	0.165	0.2677419	ns
32	-0.34361449	105.129	0.732	0.7956522	ns	32	1.43149832	96.90853	0.156	0.26	ns
33	-0.4435534	105.74448	0.658	0.7563218	ns	33	1.48572075	95.69426	0.141	0.2431034	ns
34	-0.56562898	106.61951	0.573	0.7440476	ns	34	1.54473825	94.39751	0.126	0.2290909	ns
35	-0.71779939	107.57976	0.474	0.6913043	ns	35	1.60311507	93.02539	0.112	0.2150943	ns

36	-0.89438891	108.421	0.373	0.665	ns	36	1.65960205	91.61375	0.1	0.2	ns
37	-1.08169585	108.88035	0.282	0.564	ns	37	1.71441691	90.28544	0.0899	0.2	ns
38	-1.27948275	108.71166	0.203	0.472093	ns	38	1.7601596	89.16257	0.0818	0.2	ns
39	-1.48473636	108.08744	0.141	0.3810811	ns	39	1.79065244	88.38723	0.0768	0.2	ns
40	-1.68803487	107.46063	0.0943	0.3041935	ns	40	1.80843303	88.10992	0.074	0.2	ns
41	-1.87961757	107.19184	0.0629	0.232963	ns	41	1.81513811	88.3647	0.0729	0.2	ns
42	-2.05669409	107.50661	0.0421	0.1830435	ns	42	1.82245139	89.01302	0.0717	0.2	ns
43	-2.22415243	108.22769	0.0282	0.1484211	ns	43	1.83859433	89.90069	0.0693	0.2	ns
44	-2.37704685	109.13509	0.0192	0.1371429	ns	44	1.86904481	90.90934	0.0648	0.2	ns
45	-2.50718998	109.75131	0.0136	0.1346154	ns	45	1.90824853	91.84339	0.0595	0.2	ns
46	-2.60421657	109.81255	0.0105	0.1346154	ns	46	1.95372546	92.52967	0.0538	0.1992593	ns
47	-2.65847653	109.44159	0.00903	0.1346154	ns	47	1.99286923	92.91983	0.0492	0.1926923	ns
48	-2.66827147	108.79413	0.00879	0.1346154	ns	48	2.03025531	93.01736	0.0452	0.1883333	ns
49	-2.63540654	107.65777	0.00964	0.1346154	ns	49	2.06339212	92.88735	0.0419	0.1883333	ns
50	-2.55326883	106.60112	0.0121	0.1346154	ns	50	2.10442242	92.52846	0.0381	0.1814286	ns
51	-2.43948782	105.9934	0.0164	0.1346154	ns	51	2.16713375	91.9931	0.0328	0.1784211	ns
52	-2.3010003	105.61581	0.0234	0.1452941	ns	52	2.24877828	91.33739	0.0269	0.1582353	ns
53	-2.13348815	104.87567	0.0352	0.167619	ns	53	2.31694503	90.57383	0.0228	0.145	ns
54	-1.92775404	103.97851	0.0566	0.2264	ns	54	2.36720082	89.62291	0.0201	0.1435714	ns
55	-1.69464117	103.18925	0.0932	0.3041935	ns	55	2.41061768	88.6634	0.018	0.1384615	ns
56	-1.44623597	102.76247	0.151	0.3871795	ns	56	2.46387731	87.88401	0.0157	0.1375	ns
57	-1.20563392	102.36543	0.231	0.5065217	ns	57	2.53086147	87.36476	0.0132	0.132	ns
58	-0.98974535	102.19587	0.325	0.625	ns	58	2.60434367	87.24784	0.0108	0.132	ns
59	-0.79826357	102.3012	0.427	0.6809524	ns	59	2.67498498	87.39091	0.00892	0.132	ns
60	-0.63536278	102.73794	0.527	0.7347222	ns	60	2.73017181	87.7469	0.00765	0.132	ns

61	-0.51381384	103.66419	0.608	0.7440476	ns	61	2.76143748	88.36996	0.007	0.132	ns
62	-0.44606627	104.8756	0.656	0.7563218	ns	62	2.75894335	89.35103	0.00703	0.132	ns
63	-0.41212003	105.43921	0.681	0.7604396	ns	63	2.72184324	90.67989	0.00778	0.132	ns
64	-0.4052231	104.92033	0.686	0.7604396	ns	64	2.66862801	92.1733	0.009	0.132	ns
65	-0.4249132	104.28397	0.672	0.7604396	ns	65	2.60973469	93.33013	0.0106	0.132	ns
66	-0.45882834	103.84693	0.647	0.7563218	ns	66	2.54308192	93.93398	0.0126	0.132	ns
67	-0.52429529	103.05042	0.601	0.7440476	ns	67	2.44090402	93.81352	0.0165	0.1375	ns
68	-0.63173439	101.78653	0.529	0.7347222	ns	68	2.30793806	93.38486	0.0232	0.145	ns
69	-0.77542764	100.4847	0.44	0.6833333	ns	69	2.15343254	92.90769	0.0339	0.1784211	ns
70	-0.93849833	99.32227	0.35	0.6363636	ns	70	1.98514215	92.49198	0.0501	0.1926923	ns
71	-1.13129447	98.78581	0.261	0.5326531	ns	71	1.82223026	92.22759	0.0717	0.2	ns
72	-1.34409333	99.2601	0.182	0.4333333	ns	72	1.66034094	92.04982	0.1	0.2	ns
73	-1.56535508	100.44842	0.121	0.3666667	ns	73	1.48901067	92.01505	0.14	0.2431034	ns
74	-1.79061162	101.78513	0.0763	0.2725	ns	74	1.3150352	92.17697	0.192	0.2742857	ns
75	-1.98843828	102.88116	0.0494	0.2058333	ns	75	1.13523705	92.15849	0.259	0.3453333	ns
76	-2.13860897	103.51453	0.0348	0.167619	ns	76	0.95462051	92.0343	0.342	0.45	ns
77	-2.25492057	103.83256	0.0262	0.1455556	ns	77	0.78602217	92.28377	0.434	0.5636364	ns
78	-2.34730977	104.19155	0.0208	0.1386667	ns	78	0.64715865	93.16521	0.519	0.6653846	ns
79	-2.41528381	104.63085	0.0175	0.1346154	ns	79	0.53851689	94.51809	0.591	0.7481013	ns
80	-2.46059878	105.07668	0.0155	0.1346154	ns	80	0.4644747	96.01252	0.643	0.7608696	ns
81	-2.50556964	105.60471	0.0138	0.1346154	ns	81	0.41735757	97.34346	0.677	0.7608696	ns
82	-2.54873029	106.01753	0.0122	0.1346154	ns	82	0.39135463	98.36811	0.696	0.7608696	ns
83	-2.53497961	106.22057	0.0127	0.1346154	ns	83	0.38611551	98.59096	0.7	0.7608696	ns
84	-2.43954004	106.24116	0.0164	0.1346154	ns	84	0.38923403	97.78846	0.698	0.7608696	ns
85	-2.27797514	106.41772	0.0247	0.1452941	ns	85	0.39925033	96.32654	0.691	0.7608696	ns

86	-2.08301236	107.0292	0.0396	0.18	ns	86	0.41314802	94.54629	0.68	0.7608696	ns
87	-1.88669904	108.01849	0.0619	0.232963	ns	87	0.42537912	92.74034	0.672	0.7608696	ns
88	-1.71642301	109.1675	0.0889	0.3041935	ns	88	0.43245677	91.16284	0.666	0.7608696	ns
89	-1.58916125	110.41523	0.115	0.359375	ns	89	0.43614514	89.82353	0.664	0.7608696	ns
90	-1.51479732	111.85551	0.133	0.3810811	ns	90	0.43324278	88.75437	0.666	0.7608696	ns
91	-1.496446	113.19303	0.137	0.3810811	ns	91	0.41913392	88.00918	0.676	0.7608696	ns
92	-1.49077996	113.89386	0.139	0.3810811	ns	92	0.38822527	87.58333	0.699	0.7608696	ns
93	-1.46309567	114.0529	0.146	0.3842105	ns	93	0.32786768	87.99594	0.744	0.7770833	ns
94	-1.40603198	113.95023	0.162	0.405	ns	94	0.23450569	89.62992	0.815	0.8402062	ns
95	-1.34238827	113.57322	0.182	0.4333333	ns	95	0.0991402	93.04804	0.921	0.930303	ns
96	-1.26473251	112.92743	0.209	0.475	ns	96	-0.06079054	97.18358	0.952	0.952	ns
97	-1.13160144	112.04286	0.26	0.5326531	ns	97	-0.21344259	100.61245	0.831	0.8479592	ns
98	-0.96261024	112.08465	0.338	0.6259259	ns	98	-0.32519854	101.75615	0.746	0.7770833	ns
99	-0.76501171	113.46142	0.446	0.6833333	ns	99	-0.36576007	99.67331	0.715	0.7688172	ns
100	-0.52155756	115.3739	0.603	0.7440476	ns	100	-0.34665953	95.27582	0.73	0.7765957	ns
19	statistic	df	p	p.adj	p.adj.signif	20	statistic	df	p	p.adj	p.adj.signif
1	-2.54991384	127.53107	0.012	0.2657143	ns	1	0.03701423	102.26075	0.971	0.99	ns
2	-2.56349427	126.95571	0.0115	0.2657143	ns	2	-0.01263793	101.16824	0.99	0.99	ns
3	-2.53057662	126.12021	0.0126	0.2657143	ns	3	-0.014291	100.71709	0.989	0.99	ns
4	-2.48875192	125.18114	0.0141	0.2657143	ns	4	0.0428648	100.47807	0.966	0.99	ns
5	-2.4670869	124.57987	0.015	0.2657143	ns	5	0.07381094	101.72114	0.941	0.99	ns
6	-2.43342256	124.32564	0.0164	0.2657143	ns	6	0.08018265	103.85943	0.936	0.99	ns
7	-2.38478544	124.61189	0.0186	0.2657143	ns	7	0.11468866	105.74686	0.909	0.98064516	ns
8	-2.30438331	125.31982	0.0228	0.285	ns	8	0.19572081	106.50017	0.845	0.95955056	ns
9	-2.20692171	126.12566	0.0291	0.3233333	ns	9	0.33017988	105.65837	0.742	0.8939759	ns

10	-2.08242163	126.77952	0.0393	0.393	ns	10	0.52754889	103.12734	0.599	0.77307692	ns
11	-1.89834078	127.29268	0.0599	0.4233333	ns	11	0.77978753	100.04601	0.437	0.64057971	ns
12	-1.68328527	127.67768	0.0948	0.4291667	ns	12	1.01048645	97.41538	0.315	0.55438596	ns
13	-1.47737878	127.84477	0.142	0.4580645	ns	13	1.17322624	95.90207	0.244	0.52708333	ns
14	-1.26874297	127.90126	0.207	0.5594595	ns	14	1.23598989	95.73808	0.219	0.52708333	ns
15	-1.0677734	127.92212	0.288	0.5857143	ns	15	1.22056358	96.03659	0.225	0.52708333	ns
16	-0.90216325	127.94417	0.369	0.604918	ns	16	1.15267149	97.24002	0.252	0.52708333	ns
17	-0.77405407	127.96915	0.44	0.6197183	ns	17	1.01590957	98.60779	0.312	0.55438596	ns
18	-0.67971265	127.99297	0.498	0.6379747	ns	18	0.90100238	99.90405	0.37	0.61666667	ns
19	-0.63257728	127.99987	0.528	0.6392857	ns	19	0.84834747	101.28505	0.398	0.63809524	ns
20	-0.62839815	127.99967	0.531	0.6392857	ns	20	0.8259241	102.70444	0.411	0.64	ns
21	-0.64415652	127.99844	0.521	0.6392857	ns	21	0.78473377	103.82361	0.434	0.64057971	ns
22	-0.68672431	127.99411	0.493	0.6379747	ns	22	0.72844503	104.82432	0.468	0.65915493	ns
23	-0.77860719	127.95004	0.438	0.6197183	ns	23	0.63574407	106.08398	0.526	0.73055556	ns
24	-0.88894569	127.78182	0.376	0.6064516	ns	24	0.52570382	107.33263	0.6	0.77307692	ns
25	-0.99013267	127.55187	0.324	0.5857143	ns	25	0.43348867	108.41957	0.666	0.84303797	ns
26	-1.0326808	127.29905	0.304	0.5857143	ns	26	0.34857479	110.03662	0.728	0.88780488	ns
27	-0.98182632	127.00095	0.328	0.5857143	ns	27	0.25818602	111.8602	0.797	0.93764706	ns
28	-0.84259413	126.47459	0.401	0.6169231	ns	28	0.14978596	113.32193	0.881	0.97888889	ns
29	-0.61876598	125.70154	0.537	0.6392857	ns	29	0.02377243	114.54879	0.981	0.99	ns
30	-0.34432783	124.88636	0.731	0.7726316	ns	30	-0.11022629	115.98441	0.912	0.98064516	ns
31	-0.0433463	124.24264	0.965	0.965	ns	31	-0.22770741	117.70094	0.82	0.95348837	ns
32	0.25659709	123.99288	0.798	0.8226804	ns	32	-0.35027914	119.26329	0.727	0.88780488	ns
33	0.53235402	124.44339	0.595	0.6685393	ns	33	-0.5240471	120.62216	0.601	0.77307692	ns
34	0.78182724	125.38306	0.436	0.6197183	ns	34	-0.77071992	121.58968	0.442	0.64057971	ns

35	0.9904727	126.45334	0.324	0.5857143	ns	35	-1.15806523	122.48168	0.249	0.52708333	ns
36	1.13961164	127.33948	0.257	0.58	ns	36	-1.63123586	123.31121	0.105	0.28378378	ns
37	1.23297252	127.86597	0.22	0.5641026	ns	37	-2.04496274	123.40646	0.043	0.13030303	ns
38	1.2822837	127.99741	0.202	0.5594595	ns	38	-2.30572269	123.33034	0.0228	0.076	ns
39	1.28569679	127.86999	0.201	0.5594595	ns	39	-2.44985118	122.58349	0.0157	0.05888889	ns
40	1.25193958	127.71863	0.213	0.5605263	ns	40	-2.53893371	121.66342	0.0124	0.0496	*
41	1.20779026	127.58568	0.229	0.5725	ns	41	-2.64429152	120.27329	0.00928	0.04833333	*
42	1.12912155	127.47259	0.261	0.58	ns	42	-2.80146399	118.38148	0.00594	0.04569231	*
43	1.02199566	127.48616	0.309	0.5857143	ns	43	-2.95314144	116.54204	0.00381	0.03463636	*
44	0.9168475	127.49807	0.361	0.6016667	ns	44	-3.0645955	115.23405	0.00271	0.033875	*
45	0.82676539	127.39696	0.41	0.6197183	ns	45	-3.09235632	115.49903	0.00249	0.033875	*
46	0.78277669	127.19489	0.435	0.6197183	ns	46	-2.97208062	117.44839	0.00359	0.03463636	*
47	0.80105133	127.03799	0.425	0.6197183	ns	47	-2.73760408	119.23346	0.00714	0.04764706	*
48	0.87304887	127.06603	0.384	0.6095238	ns	48	-2.44594718	119.82923	0.0159	0.05888889	ns
49	0.94684588	127.05927	0.346	0.5965517	ns	49	-2.13609522	119.14982	0.0347	0.1084375	ns
50	0.98378248	127.01745	0.327	0.5857143	ns	50	-1.76857773	117.62994	0.0796	0.22742857	ns
51	1.00348083	127.01442	0.318	0.5857143	ns	51	-1.32949282	114.83759	0.186	0.47692308	ns
52	0.95988109	127.0377	0.339	0.5947368	ns	52	-0.84129265	112.08357	0.402	0.63809524	ns
53	0.85428929	127.1237	0.395	0.6169231	ns	53	-0.3097813	112.65464	0.757	0.90119048	ns
54	0.75985067	127.38883	0.449	0.6236111	ns	54	0.1959518	115.43674	0.845	0.95955056	ns
55	0.71356182	127.76014	0.477	0.6379747	ns	55	0.55844768	117.72551	0.578	0.77307692	ns
56	0.69513694	127.98656	0.488	0.6379747	ns	56	0.75516562	119.62091	0.452	0.64571429	ns
57	0.63519671	127.93705	0.526	0.6392857	ns	57	0.88348181	121.45453	0.379	0.62131148	ns
58	0.51139424	127.72312	0.61	0.6777778	ns	58	1.00663409	122.23981	0.316	0.55438596	ns
59	0.39836119	127.39116	0.691	0.7593407	ns	59	1.10707023	120.98776	0.27	0.53921569	ns

60	0.34521489	126.88518	0.731	0.7726316	ns	60	1.18752684	119.86239	0.237	0.52708333	ns
61	0.34083386	126.30033	0.734	0.7726316	ns	61	1.19528472	119.70185	0.234	0.52708333	ns
62	0.31239565	126.09683	0.755	0.7864583	ns	62	1.14759888	119.83501	0.253	0.52708333	ns
63	0.16099143	126.67614	0.872	0.8897959	ns	63	1.09637334	119.90065	0.275	0.53921569	ns
64	-0.07445556	127.45394	0.941	0.9505051	ns	64	1.0503982	120.8268	0.296	0.55438596	ns
65	-0.34725211	127.87685	0.729	0.7726316	ns	65	1.02556838	123.22941	0.307	0.55438596	ns
66	-0.57868745	127.97593	0.564	0.6477273	ns	66	1.02479379	126.05998	0.307	0.55438596	ns
67	-0.69365947	127.81437	0.489	0.6379747	ns	67	0.97473059	127.53436	0.332	0.56779661	ns
68	-0.66968988	126.83945	0.504	0.6379747	ns	68	0.81520574	127.96822	0.416	0.64	ns
69	-0.59575356	124.98171	0.552	0.6418605	ns	69	0.52164027	127.98964	0.603	0.77307692	ns
70	-0.57024862	123.56152	0.57	0.6477273	ns	70	0.18406347	127.90811	0.854	0.95955056	ns
71	-0.6044023	123.17478	0.547	0.6418605	ns	71	-0.11398079	127.76378	0.909	0.98064516	ns
72	-0.72798038	123.07812	0.468	0.6379747	ns	72	-0.35592273	127.45781	0.722	0.88780488	ns
73	-0.91828149	123.57146	0.36	0.6016667	ns	73	-0.56908394	127.09637	0.57	0.77307692	ns
74	-1.14279649	124.75646	0.255	0.58	ns	74	-0.78152864	127.41978	0.436	0.64057971	ns
75	-1.3861099	125.99905	0.168	0.525	ns	75	-0.96851314	127.91185	0.335	0.56779661	ns
76	-1.598494	126.97609	0.112	0.4384615	ns	76	-1.12731735	127.99513	0.262	0.53469388	ns
77	-1.72293734	127.82364	0.0873	0.4233333	ns	77	-1.28175283	127.98381	0.202	0.505	ns
78	-1.78665189	127.74287	0.0764	0.4233333	ns	78	-1.45327465	127.9087	0.149	0.39210526	ns
79	-1.81206976	125.16876	0.0724	0.4233333	ns	79	-1.6413453	127.48418	0.103	0.28378378	ns
80	-1.86513898	118.65431	0.0646	0.4233333	ns	80	-1.8838682	127.34589	0.0619	0.18205882	ns
81	-1.93060687	108.81514	0.0561	0.4233333	ns	81	-2.15951604	127.57516	0.0327	0.10548387	ns
82	-1.94438489	99.43699	0.0547	0.4233333	ns	82	-2.40030653	127.90928	0.0178	0.06357143	ns
83	-1.89524145	94.12564	0.0611	0.4233333	ns	83	-2.57328784	127.9993	0.0112	0.04833333	*
84	-1.78390376	92.63391	0.0777	0.4233333	ns	84	-2.6901142	127.87288	0.0081	0.04764706	*

85	-1.59549689	93.3566	0.114	0.4384615	ns	85	-2.71269401	127.44229	0.0076	0.04764706	*
86	-1.36057325	93.68599	0.177	0.5363636	ns	86	-2.65614726	126.50966	0.00892	0.04833333	*
87	-1.15452951	92.48499	0.251	0.58	ns	87	-2.59690406	124.88642	0.0105	0.04833333	*
88	-1.04563986	90.59873	0.299	0.5857143	ns	88	-2.56330993	123.43685	0.0116	0.04833333	*
89	-1.04191346	90.09999	0.3	0.5857143	ns	89	-2.59143887	122.53479	0.0107	0.04833333	*
90	-1.09636296	92.99644	0.276	0.5857143	ns	90	-2.71338319	121.18314	0.00763	0.04764706	*
91	-1.18866637	98.57953	0.237	0.5780488	ns	91	-2.88764812	119.57311	0.00461	0.03841667	*
92	-1.33461096	104.47454	0.185	0.5441176	ns	92	-3.07959501	119.23577	0.00257	0.033875	*
93	-1.50616274	109.07655	0.135	0.4580645	ns	93	-3.2675639	120.22446	0.00141	0.0324	*
94	-1.64712717	110.99346	0.102	0.4291667	ns	94	-3.39145458	120.99378	0.00094	0.03133333	*
95	-1.73234097	110.41076	0.086	0.4233333	ns	95	-3.46187659	122.42685	0.000739	0.03133333	*
96	-1.7166252	107.85766	0.0889	0.4233333	ns	96	-3.40839841	123.95133	0.000882	0.03133333	*
97	-1.64589556	105.80022	0.103	0.4291667	ns	97	-3.22238035	124.52248	0.00162	0.0324	*
98	-1.54960379	105.43889	0.124	0.4580645	ns	98	-2.96713009	123.54651	0.00361	0.03463636	*
99	-1.5078547	106.92863	0.135	0.4580645	ns	99	-2.59085181	121.52291	0.0107	0.04833333	*
100	-1.47789756	109.01006	0.142	0.4580645	ns	100	-2.31314607	118.06775	0.0224	0.076	ns
